# Constructing chiral bicyclo[3.2.1]octanes via palladium-catalyzed asymmetric tandem Heck/carbonylation desymmetrization of cyclopentenes

**DOI:** 10.1038/s41467-020-16221-9

**Published:** 2020-05-21

**Authors:** Zhenbo Yuan, Yuye Zeng, Ziwen Feng, Zhe Guan, Aijun Lin, Hequan Yao

**Affiliations:** State Key Laboratory of Natural Medicines (SKLNM) and Department of Medicinal Chemistry, School of Pharmacy, China Pharmaceutical University, Nanjing, 210009 P. R. China

**Keywords:** Asymmetric catalysis, Asymmetric synthesis, Synthetic chemistry methodology

## Abstract

Transition-metal-catalyzed tandem Heck/carbonylation reaction has emerged as a powerful tool for the synthesis of structurally diverse carbonyl molecules, as well as natural products and pharmaceuticals. However, the asymmetric version was rarely reported, and remains a challenging topic. Herein, we describe a palladium-catalyzed asymmetric tandem Heck/carbonylation desymmetrization of cyclopentenes. Alcohols, phenols and amines are employed as versatile coupling reagents for the construction of multifunctional chiral bicyclo[3.2.1]octanes with one all-carbon quaternary and two tertiary carbon stereogenic centers in high diastereo- and enantioselectivities. This study represents an important progress in both the asymmetric tandem Heck/carbonylation reactions and enantioselective difunctionalization of internal alkenes.

## Abstract (web summary)

Tandem Heck/carbonylation reaction gives access to ubiquitous carbonyl molecules, however the asymmetric version is rarely studied. Here, the authors synthesize chiral bicyclo[3.2.1]octanes with a palladium-catalyzed asymmetric tandem Heck/carbonylation desymmetrization of cyclopentenes with alcohols, phenols and amines.

## Introduction

**Fig. 1:**
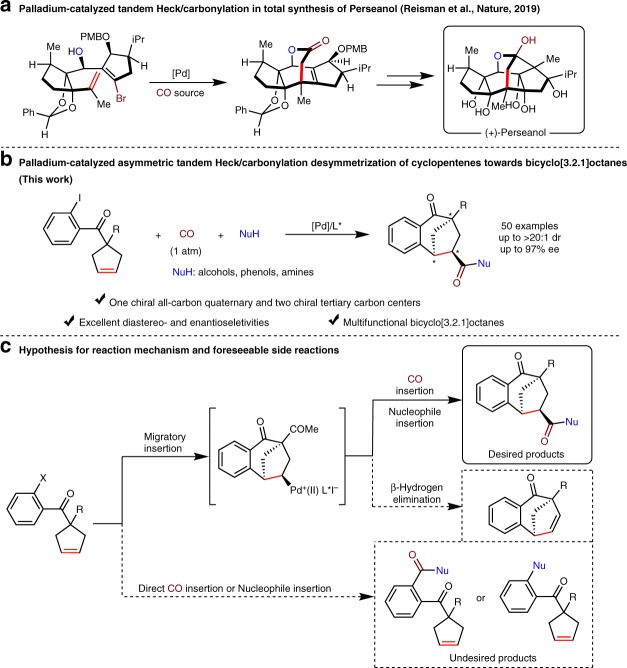
Palladium-catalyzed tandem Heck/carbonylation reactions of alkenes. **a** Pd-catalyzed tandem Heck/carbonylation as key step to the total synthesis of (+)-Perseanol. **b** Palladium-catalyzed asymmetric tandem Heck/carbonylation of internal alkenes towards bicyclo[3.2.1]octanes (this work). **c** Hypothesis of mechanism for the tandem Heck/carbonylation reaction and foreseeable side reactions.

Transition-metal (TM)-catalyzed carbonylation reaction^1–16^, especially palladium-catalyzed tandem Heck/carbonylation reaction, presents an efficient method to construct a variety of synthetically versatile carbonyl compounds from readily accessible organic halides and alkenes^17–21^. Moreover, these methods have been applied as key steps in the total synthesis of natural products and bioactive molecules^22–28^. Very recently, Reisman and co-workers realized the total synthesis of (+)-Perseanol employing palladium-catalyzed tandem Heck/carbonylation to assemble the vital tetracyclic core (Fig. 1a)^29^. However, the asymmetric version of tandem Heck/carbonylation reactions was rare, and remains a challenging topic. Some inherent issues, such as the strong π-acidity and coordination ability of CO, would hamper the oxidative addition of organohalides towards low-valent metal species^30^. In addition, the harsh reaction conditions (high temperature and high CO pressure), the incidental racemization^31^, the β-hydrogen elimination of alkylpalladium intermediates, the direct carbonylation of organohalides, and other competitive side-reactions make the asymmetric progress more difficult and complicated. Recently, three representative works on palladium-catalyzed asymmetric tandem Heck/carbonylation reaction of 1,1-disubstitueted alkenes to synthesize dihydrobenzofurans, oxindole derivatives, and 3,4-dihydroisoquinolines have been realized by Correia’s group^32^, Zhu and Luo’s group^33^, and Zhang’s group^34^, respectively. In contrast to the success of 1,1-disubstituted alkenes (the alkylpalladium intermediates lack eliminable β-hydrogen), the TM-catalyzed asymmetric tandem Heck/carbonylation reaction of unactivated internal alkenes has not been developed until now.

On the other hand, bicyclo[3.2.1]octanes are found in several natural products with antibacterial, antioxidant, antithrombosis, and antitumor activities^35,36^. However, constructing such intricate polycyclic bridge ring compounds with multiple chiral centers simultaneous implementation remains a challenging project^37–46^. Based on our research interest in Heck reactions^47–50^, herein, we describe a palladium-catalyzed asymmetric tandem Heck/carbonylation desymmetrization of cyclopentenes to construct multifunctional chiral bicyclo[3.2.1]octanes bearing one all-carbon quaternary and two tertiary carbon stereogenic centers in excellent diastereoselectivities and enantioselectivities (Fig. 1b). Suppressing the foreseeable side reactions, such as β-hydrogen elimination of alkylpalladium intermediates, and the direct CO insertion or nucleophiles insertion reaction (Fig. 1c), is the key to the success of this reaction.

## Results

### Reaction optimization

After systematic evaluation of the reaction conditions, the desired chiral bicyclo[3.2.1]octane product **3aa** was achieved in 81% yield and 96% ee employing Pd_2_dba_3_·CHCl_3_ (5 mol%) as the catalyst, (*S*)-Difluorphos **L1** (20 mol%) as the ligand, K_2_CO_3_ (2 equiv) as the base, and mixed 1,2-dichloroethane (DCE)/dichloromethane (DCM) (10/1) as the solvent at 100 °C (Table 1, entry 1). Other catalysts, such as Pd(OAc)_2_ and Pd_2_(dba)_3_ were less effective (entries 2 and 3). BINAP **L2**, SEGPHOS **L3**, DM-SEGPHOS **L4** offered **3aa** in 52–80% ee (entries 4–6), while BOX-type ligand **L5**, PHOX-type ligand **L6**, bis(phosphine-amide) ligand **L7** and phosphoramidite ligand **L8** caused the reaction inactivation (entries 7–10). Decreasing the amount of ligand resulted **3aa** in diminished yield, diastereo- and enantioselectivity (entry 11). Screening the additives revealed that K_2_CO_3_ was optimal, and AgOAc delivered racemic **3a′** in 83% yield, which was formed via β-hydrogen elimination (entries 1 and 12–14). The choice of solvent, also the ratio of the mixed solvent, was crucial to the reaction efficiency (entries 15–20). Adjusting the reaction temperature was inconducive to improve the outcome of the reaction (entries 21–22). The structure and absolute configuration of **3aa** were confirmed by single-crystal X-ray diffraction analysis (see the Supplementary Note 3 for details).

**Table 1:** Optimization of reaction conditions^a^.


Entry	Deviation of standard conditions	Yield of 3aa (%)^b^	Dr of 3aa^c^	Ee of 3aa (%)^d^	Yield of 3a′ (%)^b^
1	None	81	>20:1	96	<2
2	Pd(OAc)_2_ instead of Pd_2_dba_3_·CHCl_3_	71	>20:1	66	6
3	Pd_2_dba_3_ instead of Pd_2_dba_3_·CHCl_3_	31	>20:1	85	7
4	**L2** instead of **L1**	82	>20:1	52	<2
5	**L3** instead of **L1**	75	15:1	79	9
6	**L4** instead of **L1**	60	>20:1	80	<2
7	**L5** instead of **L1**	59	>20:1	−5	<2
8	**L6** instead of **L1**	21	>20:1	5	<2
9	**L7** instead of **L1**	<2	-	-	13
10	**L8** instead of **L1**	<2	-	-	21
11	10 mol% of **L1** instead of 20 mol% of **L1**	63	11:1	36	24
12	KHCO_3_ instead of K_2_CO_3_	57	4:1	80	<2
13	Na_2_CO_3_ instead of K_2_CO_3_	32	7:1	53	<2
14	AgOAc instead of K_2_CO_3_	<2	–	–	83
15	DCE instead of DCE/DCM	78	4:1	80	<2
16	DCM instead of DCE/DCM	75	>20:1	88	<2
17	Toluene instead of DCE/DCM	69	5:1	53	<2
18	CH_3_CN instead of DCE/DCM	90	8:1	77	<2
19	DCE/DCM = 1/1 instead of 10/1	71	10:1	96	8
20	DCE/DCM = 1/10 instead of 10/1	38	12:1	93	38
21	80 °C instead of 100 °C	71	>20:1	77	<2
22	120 °C instead of 100 °C	38	13:1	94	<2

^a^Reaction conditions: **1a** (0.1 mmol), **2a** (1 mmol), [Pd] (10 mol%), ligand (20 mol%), base (0.2 mmol) in 1 mL solvent, 100 °C, 36 h, under CO (1 atm).

^b^Isolated yield.

^c^Determined by ^1^H NMR analysis.

^d^Determined by HPLC analysis on a chiral stationary phase.

### Substrate scope

**Fig. 2:**
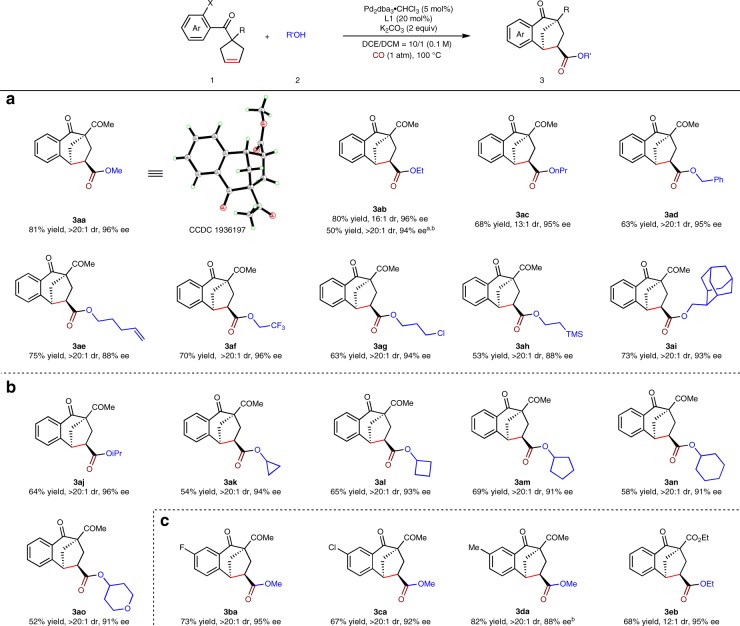
Substrate scope. **a** Substrate scope of primary alcohols. **b** Substrate scope of secondary alcohols. **c** Substrate scope of the benzoylcyclopentenes. Reaction conditions: *X* = I, **1** (0.1 mmol), **2** (1 mmol), Pd_2_dba_3_•CHCl_3_ (5 mol%), **L1** (20 mol%), K_2_CO_3_ (0.2 mmol) in 1 mL solvent, 100 °C, 36 h, under CO (1 atm). Yields of isolated products are given. The dr values were determined by ^1^H NMR analysis. The ee values were determined by HPLC analysis on a chiral stationary phase. ^a^X = Br. ^b^48 h.

With the optimized reaction conditions in hand, we then tested the substrate scope of alcohols in this asymmetric Heck/carbonylation reaction, and the results were summarized in Fig. 2. Simple primary alcohols, such as ethanol, *n*-propanol and benzyl alcohol afforded the products **3aa**–**ad** in moderate to good yields with high enantioselectivities. It is noted that aryl bromine derivative was also a good candidate, delivering **3ab** in 50% yield with 94% ee after prolonging the time to 48 h. Besides, other primary alcohols with various functional groups, such as alkenyl, trifluoromethyl, halogen, trimethylsilyl, even highly sterically hindered adamantly group, all performed well, offering **3ae**–**ai** in 88–96% ee. Cyclic and acyclic secondary alcohols delivered the corresponding products **3aj**–**ao** in good efficiency. Products **3ba**–**da** with different substituents on the benzene ring were produced in good yields with high enantioselectivities. Product **3eb** with two ester groups was achieved in 68% yield with 95% ee.

**Fig. 3:**
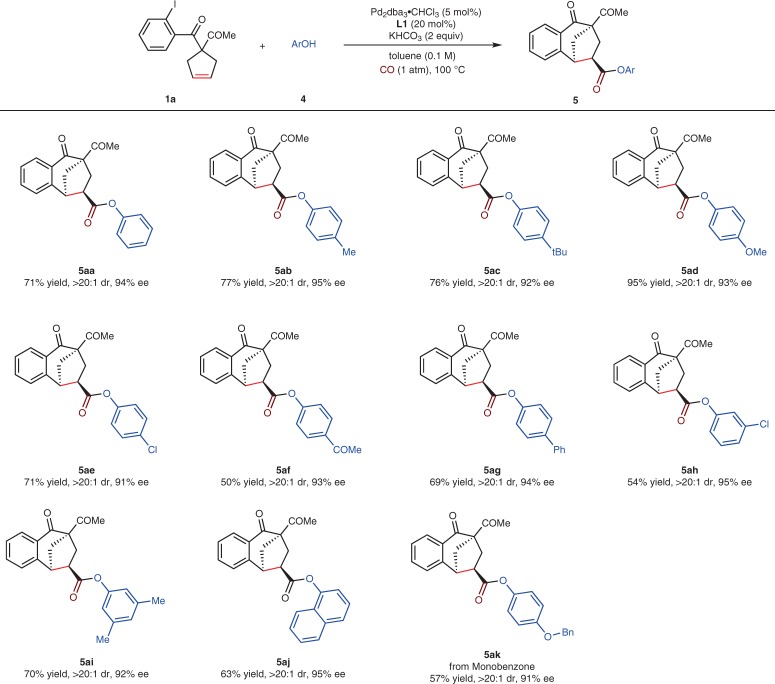
Substrate scope of phenols. Reaction conditions: **1a** (0.1 mmol), **4** (0.25 mmol), Pd_2_dba_3_•CHCl_3_ (5 mol%), **L1** (20 mol%), KHCO_3_ (0.2 mmol) in toluene (1 mL), 100 °C, 36 h, under CO (1 atm). Yields of isolated products are given. The dr values were determined by ^1^H NMR analysis. The ee values were determined by HPLC analysis on a chiral stationary phase.

Phenol esters are important skeletons in pharmaceuticals and bioactive compounds. Although phenols as nucleophilic reagents have been employed in some carbonylation reactions^51,52^, they have not met with the success in asymmetric tandem Heck/carbonylation reactions, because the two potential nucleophilic sites at O and C of phenols would increase the complexity of the reaction. Herein, phenols as versatile components were performed in our asymmetric Heck/carbonylation reactions with KHCO_3_ as the base and toluene as the solvent (Fig. 3). Electron donating groups (−Me, −*t*Bu, and −OMe), a halogen group (−Cl), an electron withdrawing group (−COMe), as well as a phenyl group at the *para*-position of phenols offered the corresponding products **5aa**–**ag** in 91–95% ee. *meta*-Chlorine substituted phenol **4h** and 3,5-dimethylphenol **4i** could also fulfill the reaction well, and no significant steric hindrance effect was observed. 1-Naphthol delivered **5aj** in 95% ee. Moreover, monobenzone, as a potent skin lightener drug, could give the adduct **5ak** in 91% ee.

**Fig. 4:**
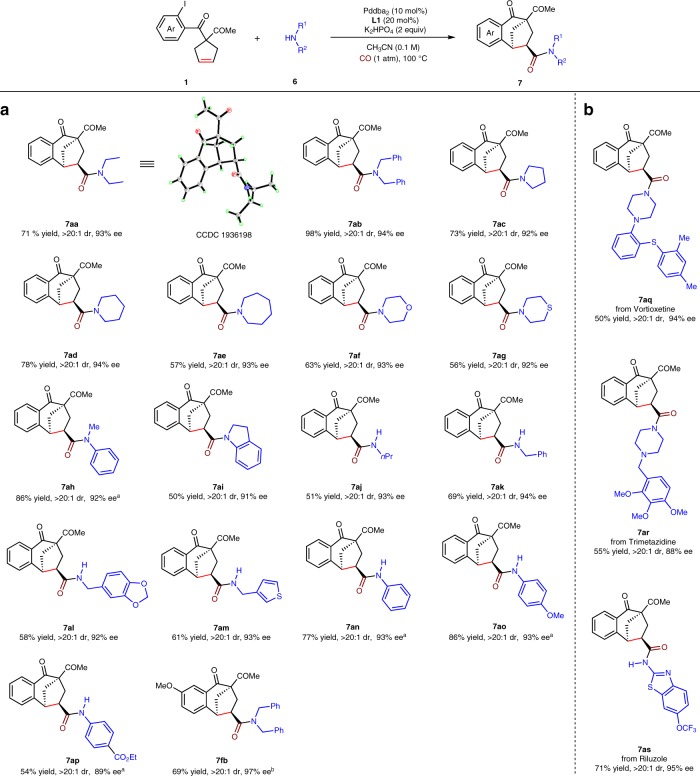
Substrate scope. **a** Substrate scope of amines. **b** Application to asymmetric late-stage functionalization of pharmaceuticals. Reaction conditions: **1a** (0.1 mmol), **6** (0.2 mmol), Pddba_2_ (10 mol%), **L1** (20 mol%), K_2_HPO_4_ (0.2 mmol) in CH_3_CN (1 mL), 100 °C, 36 h, under CO (1 atm). Yields of isolated products are given. The dr values were determined by ^1^H NMR analysis. The ee values were determined by HPLC analysis on a chiral stationary phase. ^a^K_2_HPO_4_ was replaced by KHCO_3_, 48 h. ^b^48 h.

To further exhibit the robustness and generality of this reaction, scope of nitrogen nucleophiles was investigated with Pddba_2_ (10 mol%) as the catalyst, **L1** (20 mol%) as the ligand, K_2_HPO_4_ (0.2 mmol) as the base in CH_3_CN (1 mL) at 100 °C (Fig. 4). Acyclic secondary alkylamines, such as diethylamine and dibenzylamine delivered products **7aa** (see the Supplementary Note 4 for details on the X-ray crystal structure) and **7ab** in 93 and 94% ee. Cyclic secondary alkylamines furnished products **7ac**–**ag** in 92–94% ee. Alkylarylamines, such as *N*-methylaniline and indoline, provided **7ah** and **7ai** in 92 and 91% ee. Primary alkylamines, such as *n*-propylamine, benzylamines, and thiophenylmethanamine offered products **7aj**–**am** in 92**–**94% ee. Primary arylamines were also qualified to work in this reaction, delivering products **7an**–**ap** in good enantioselectivities with KHCO_3_ as the base after prolonging the reaction time to 48 h. 5-OMe-substituted cyclopentene **1f** performed smoothly to give **7fb** in 97% ee. Finally, pharmaceuticals including Vortioxetine, Trimetazidine and Riluzole were all well late-stage functionalized with bicyclo[3.2.1]octanes to offer **7aq**–**as** in 88–95% ee.

### Further study

**Fig. 5:**
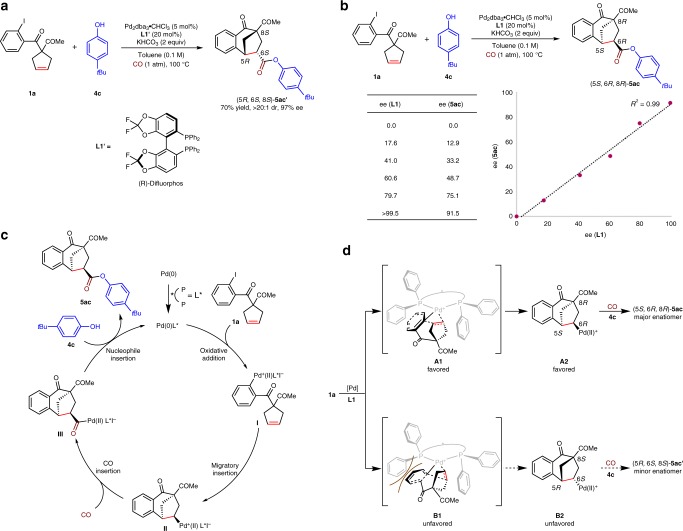
Further study on the reaction. **a** Enantiodivergent synthesis of (5*R*, 6*S*, 8*S*)-**5ac′**. **b** Linear correlation between the ee values of **5ac** and **L1**. **c** Proposed mechanism. **d** Model for enantioselectivity.

The enantiodivergent synthesis of (5*R*, 6*S*, 8*S*)-**5ac′** was also realized in 70% yield and 97% ee employing (*R*)-Difluorphos as the ligand (Fig. 5a). To demonstrate the mechanism of this reaction, study on nonlinear effect of the enantioselectivity of **5ac** was carried out (Fig. 5b). The linear correlation (*R*^2^ = 0.99) between the enantioselectivities of the product **5ac** and the enantiopurities of the ligand **L1** revealed the involvement of one active catalyst species in the stereo-determining transition states of the migratory insertion process. On the basis of the above-mentioned results and previous literatures^31,32,47^, a proposed mechanism of this reaction is figured in Fig. 5c. Firstly, oxidative addition of the active palladium catalyst with **1a** delivers the cationic Pd(II) intermediate **I**. Intramolecular *syn*-migratory insertion of **I** results in the alkylpalladium intermediate **II**, which followed by the insertion of CO delivers the intermediate **III**. Finally, the nucleophile insertion of the phenol **4c** to the intermediate **III** produces the product **5ac**. It is noted that the high diastereoselectivity was arisen from the stereospecific *syn*-migratory insertion step, which has been confirmed in our previous work by the deuterium-labeling experiments^47^. The observed stereochemical outcome of the reaction with the C2-symmetric, (*S*)-configured ligand **L1** can be rationalized based on the two diastereomeric intermediates **A1** and **B1** (Fig. 5d). The transition state **B1** is notable for the severe steric repulsion between the benzoyl moiety of the cyclopentene **1a** and the benzene ring of the ligand **L1**, a factor which is not present in the transition state **A1**; this may account for the predominance of the (5*S*, 6*R*, 8*R*) enantiomer of **5ac** in the product.

## Discussion

In summary, we have developed a Pd-catalyzed asymmetric tandem Heck/carbonylation desymmetrization of cyclopentenes. Various bicyclo[3.2.1]octanes bearing one chiral all-carbon quaternary and two tertiary carbon stereogenic centers were obtained in moderate to good yields with excellent diastereoselectivities and enantiomeric excess. This method provided a general and practical route for the enantioselective difunctionalization of unactivated internal alkenes and chiral bicyclo[3.2.1]octanes.

## Methods

### General procedure for the catalytic reactions with alchohols

A sealed tube was charged with the cyclopentenes **1** (0.1 mmol, 1 equiv), Pd_2_dba_3_•CHCl_3_ (5 mol%), **L1** (20 mol%), and K_2_CO_3_ (0.2 mmol, 2 equiv). The vial was thoroughly flushed with CO, and alchohols **2** (1 mmol, 10 equiv), as well as DCE/DCM (10/1, 1 mL) were added under CO atmosphere. The reaction mixture was stirred at 100 °C for 36 h. After the reaction vessel was cooled to room temperature, the solution was concentrated in vacuo and purified by careful chromatography on silica gel (200–300 mesh) (PE/EA = 4/1) to afford the desired products **3**.

### General procedure for the catalytic reactions with phenols

A sealed tube was charged with the cyclopentenes **1** (0.1 mmol, 1 equiv), phenols **4** (0.25 mmol, 2.5 equiv), Pd_2_dba_3_•CHCl_3_ (5 mol%), **L1** (20 mol%), and KHCO_3_ (0.2 mmol, 2 equiv). The vial was thoroughly flushed with CO, and toluene (1 mL) was added under CO atmosphere. The reaction mixture was stirred at 100 °C for 36 h. After the reaction vessel was cooled to room temperature, the solution was concentrated in vacuo and purified by careful chromatography on silica gel (200–300 mesh) (PE/EA = 4/1) to afford the desired products **5**.

### General procedure for the catalytic reactions with amines

A sealed tube was charged with the cyclopentenes **1** (0.1 mmol, 1 equiv), Pddba_2_ (10 mol%), **L1** (20 mol%), and K_2_HPO_4_ (0.2 mmol, 2 equiv). The vial was thoroughly flushed with CO, and amines **6** (0.2 mmol, 2 equiv), as well as CH_3_CN (1 mL) were added under CO atmosphere. The reaction mixture was stirred at 100 °C for 36 h. After the reaction vessel was cooled to room temperature, the solution was concentrated in vacuo and purified by careful chromatography on silica gel (200–300 mesh) (PE/EA = 2/1) to afford the desired products **7**.

## Supplementary information

Supplementary Information

## Data Availability

The authors declare that all the data supporting the findings of this study are available within the article and Supplementary Information files, and are also available from the corresponding author upon reasonable request. The X-ray crystallographic coordinates for structures reported in this article have been deposited at the Cambridge Crystallographic Data Center (CCDC) under deposition numbers 1936197 (**3aa**) and 1936198 (**7aa**). These data could be obtained free of charge from The Cambridge Crystallographic Data Center via http://www.ccdc.cam.ac.uk/data_request/cif.
