# Supplementary material for: Constructing chiral bicyclo[3.2.1]octanes via palladium-catalyzed asymmetric tandem Heck/carbonylation desymmetrization of cyclopentenes
Source: Nat Commun. 2020 May 21;11:2544. doi: 10.1038/s41467-020-16221-9 (PMC7242361; doi:10.1038/s41467-020-16221-9)
Supplement: Supplementary file 1 — Supplementary Information [file 41467_2020_16221_MOESM1_ESM.pdf]

Supplementary Information

Constructing Chiral Bicyclo[3.2.1]octanes via Palladium-Catalyzed Asymmetric Tandem Heck/Carbonylation Desymmetrization of Cyclopentenes

Yuan et al.

Contents

Supplementary Methods* *3
Supplementary Notes* *63
Supplementary References* *127

Supplementary Methods

General Information

^{1}H, ^{13}C and ^{19}F NMR spectra were collected on a 300 or 400 MHz spectrometer using $CDCl_3$ or acetone-d_6 as solvent. Chemical shifts of ^{1}H NMR were recorded in parts per million (ppm, δ) relative to tetramethylsilane (δ = 0.00). Data are reported as follows: chemical shift in (δ), multiplicity (s = singlet, d = doublet, t = triplet, q = quartet, brs = broad singlet, m = multiplet), coupling constant (Hz), and integration. High Resolution Mass measurement was performed with Electron Spray Ionization (ESI) method on a Q-TOF mass spectrometer operating in positive-ion mode. Melting point (m.p.) was measured on a microscopic melting point apparatus. Enantiomeric ratio (ee) values were determined by chiral HPLC with chiral OD, AD, OJ, AS, IA, IB columns with hexane and *i*-PrOH as solvents. Optical rotations were measured on an automatic polarimeter with $[\alpha]_D^{20}$ values reported in degrees; concentration (*c*) is in g/100 mL. PE refers to petroleum ether (b.p. 60–90 °C) and EA refers to ethyl acetate. Flash column chromatography was carried out using commercially available 200–300 mesh under pressure unless otherwise indicated. Gradient flash chromatography was conducted eluting with PE/EA. All other starting materials and solvents were commercially available and were used without further purification unless otherwise stated.

Preparation of Substrates 1

Structures of Substrates 1:

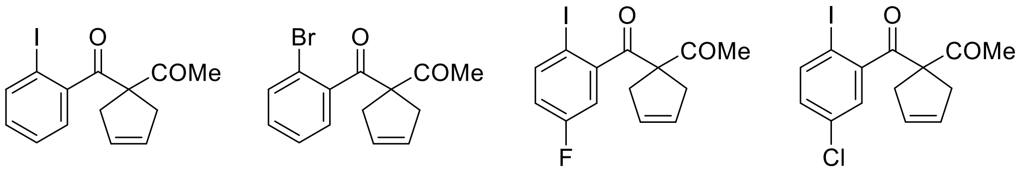

1a **1a'** **1b** **1c**

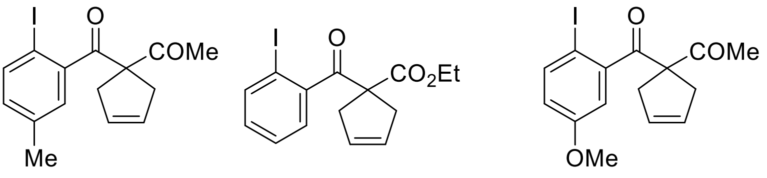

1d **1e** **1f**

General procedure for the synthesis of 1a–1f:

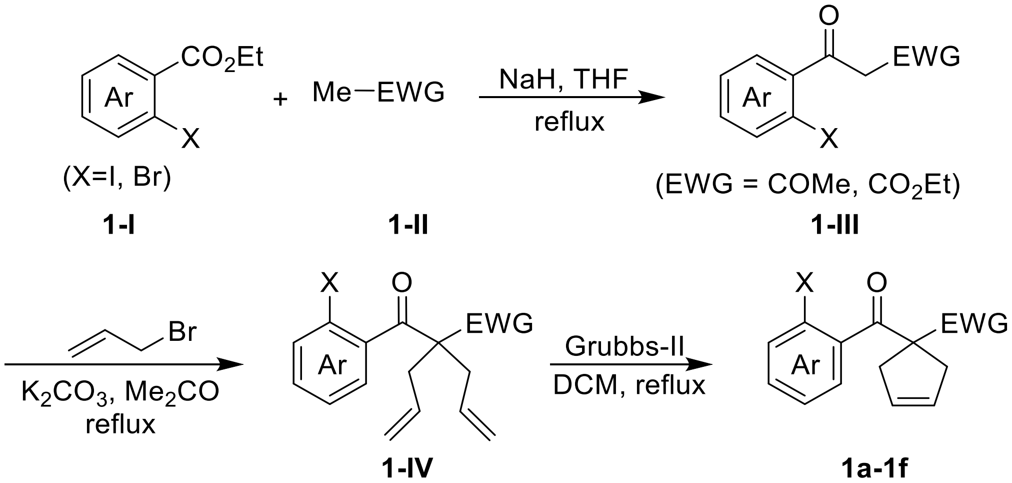

General procedure for the synthesis of 1-III[1]:

1-II (10 mmol, 1 equiv) in dry THF (10 mL) was added dropwise to a suspension of **1-I** (2 equiv) and sodium hydride (2 equiv) in dry THF (10 mL). The reaction mixture was stirred for 2 h at room temperature and then heated under reflux conditions for 24 h. After hydrolysis with saturated ammonium chloride solution, the water phase was extracted with ethyl acetate for 3 times. The combined organic layers were washed with water and brine sequentially, dried over Na_2SO_4, filtered and concentrated. The crude product was purified by flash chromatography on silica gel to afford the corresponding products **1-III**.

General procedure for the synthesis of 1-IV[2]:

To a 100 mL round-bottle flask was added **1-III** (1 equiv), acetone (30 mL), 3-bromoprop-1-ene (5 equiv) and K_2CO_3 (3 equiv). The reaction mixture was refluxed overnight, then filtered and evaporated. H_2O (10 mL) was added and the mixture was extracted with ethyl acetate for 3 times. The combined organic layer was washed with brine, then dried over Na_2SO_4, filtered and concentrated. The crude product was purified by flash chromatography on silica gel to afford the corresponding products **1-IV**.

General procedure for the synthesis of 1a–1f[3]:

1-IV (1 equiv) was placed in a dry flask, degassed and diluted with anhydrous DCM (c=0.2 M). Grubbs-II catalyst (1 mol%) was added and reaction was heated at 40 °C for 2 h. Solvent was removed under reduced pressure and the crude product was purified by flash chromatography on silica gel to afford the corresponding products.

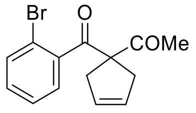

1-(1-(2-bromobenzoyl)cyclopent-3-en-1-yl)ethan-1-one (1a')

478.6 mg, 16% yield (69%, 26%, 91% yields for 3 individual steps).

^{1}H NMR (400 MHz, $CDCl_3$) δ 7.62 (d, J = 7.4 Hz, 1H), 7.33 – 7.26 (m, 2H), 7.19 (dd, J = 7.4, 1.8 Hz, 1H), 5.59 (s, 2H), 3.04 (s, 4H), 2.26 (s, 3H).

^{13}C NMR (101 MHz, $CDCl_3$) δ 203.8, 202.6, 139.9, 134.0, 131.5, 127.9, 127.4, 127.1, 119.7, 73.3, 39.9, 26.5.

HRMS (ESI) calcd. for $[C_{14}H_{13}BrO_2+H]^+$ 293.0172, found 293.0173.

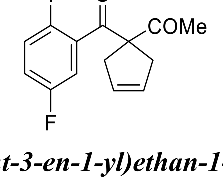

1-(1-(5-fluoro-2-iodobenzoyl)cyclopent-3-en-1-yl)ethan-1-one (1b)

499.6 mg, 14% yield (50%, 30%, 93% yields for 3 individual steps).

^{1}H NMR (300 MHz, $CDCl_3$) δ 7.88 (dd, J = 8.6, 5.3 Hz, 1H), 6.95 – 6.85 (m, 2H), 5.63 (s, 2H), 3.04 (s, 4H), 2.26 (s, 3H).

^{13}C NMR (75 MHz, $CDCl_3$) δ 204.1, 202.1, 162.1 (d, J = 251.0 Hz), 144.7 (d, J = 5.5 Hz), 142.2 (d, J = 7.4 Hz), 128.0, 119.0 (d, J = 21.6 Hz), 114.8 (d, J = 24.1 Hz), 85.5 (d, J = 3.7 Hz), 72.8, 40.0, 26.2.
^{19}F NMR (282 MHz, $CDCl_3$) δ -111.96 (d, J = 1.6 Hz).
HRMS (ESI) calcd. for $[C_{14}H_{12}FIO_2+H]^+$ 358.9939, found 358.9943.

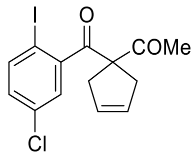

1-(1-(5-chloro-2-iodobenzoyl)cyclopent-3-en-1-yl)ethan-1-one (1c)
855.3 mg, 23% yield (73%, 34%, 92% yields for 3 individual steps).
^{1}H NMR (300 MHz, $CDCl_3$) δ 7.84 (d, J = 8.4 Hz, 1H), 7.14 (d, J = 2.3 Hz, 1H), 7.10 (ddd, J = 8.4, 2.4, 0.7 Hz, 1H), 5.63 (s, 2H), 3.04 (s, 4H), 2.27 (s, 3H).
^{13}C NMR (75 MHz, $CDCl_3$) δ 204.1, 202.4, 144.6, 141.8, 134.6, 131.7, 128.1, 127.0, 89.7, 72.8, 40.1, 26.3.
HRMS (ESI) calcd. for $[C_{14}H_{12}ClIO_2+H]^+$ 374.9643, found 374.9640.

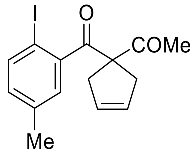

1-(1-(2-iodo-5-methylbenzoyl)cyclopent-3-en-1-yl)ethan-1-one (1d)
697.0 mg, 20% yield (67%, 33%, 89% yields for 3 individual steps).
^{1}H NMR (300 MHz, $CDCl_3$) δ 7.80 (d, J = 7.9 Hz, 1H), 6.96 – 6.91 (m, 2H), 5.60 (s, 2H), 3.05 (s, 4H), 2.29 (s, 3H), 2.24 (s, 3H).
^{13}C NMR (75 MHz, $CDCl_3$) δ 204.1, 202.8, 142.5, 140.9, 138.1, 132.8, 127.9, 127.8, 88.7, 72.9, 40.1, 26.5, 21.1.
HRMS (ESI) calcd. for $[C_{15}H_{15}IO_2+H]^+$ 355.0190, found 355.0185.

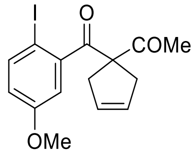

1-(1-(2-iodo-5-methoxybenzoyl)cyclopent-3-en-1-yl)ethan-1-one (1f)
1.154 g, 31% yield (79%, 42%, 94% yields for 3 individual steps).
^{1}H NMR (400 MHz, $CDCl_3$) δ 7.79 (d, J = 8.6 Hz, 1H), 6.75 (d, J = 2.9 Hz, 1H), 6.71 (dd, J = 8.7, 2.9 Hz, 1H), 5.61 (s, 2H), 3.77 (s, 3H), 3.06 (s, 4H), 2.24 (s, 3H).
^{13}C NMR (101 MHz, $CDCl_3$) δ 204.1, 202.3, 159.3, 143.1, 141.7, 127.9, 117.9, 113.5, 81.0, 72.8, 55.5, 40.1, 26.6.
HRMS (ESI) calcd. for $[C_{15}H_{15}IO_3+H]^+$ 371.0138, found 371.0139.

General Procedure for the Palladium-Catalyzed Asymmetric Tandem Heck/Carbonylation Desymmetrization of Cyclopentenes with Alcohols Exemplified by the Synthesis of 3aa

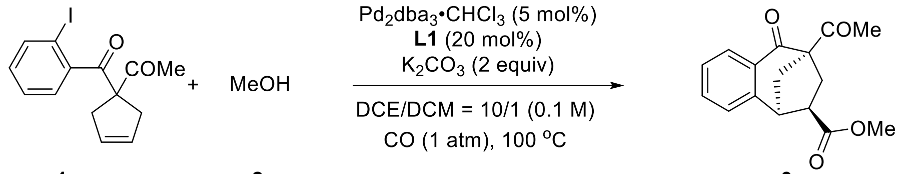

1a **2a** **3aa**

A sealed tube was charged with the substrate **1a** (34 mg, 0.1 mmol, 1 equiv), Pd_2dba_3•$CHCl_3$ (5.2 mg, 5 mol%), **L1** (13.4 mg, 20 mol%), and K_2CO_3 (27.6 mg, 0.2 mmol, 2 equiv). The vial is thoroughly flushed with CO, and **2a** (32 mg, 1 mmol, 10 equiv), as well as DCE/DCM (10/1, 1 mL) was added under CO atmosphere. Then the reaction mixture was stirred at 100 °C for 36h. After the reaction vessel was cooled to room temperature, the solution was concentrated in *vacuo* and purified by careful chromatography on silica gel (200–300 mesh) (PE/EA = 4/1) to afford the desired product **3aa**.

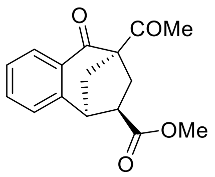

methyl (5S,6R,8R)-8-acetyl-9-oxo-6,7,8,9-tetrahydro-5H-5,8-methanobenzo[7]annulene-6-carboxylate (3aa)

22.1 mg, 81% yield, >20:1 dr, 96% *ee*; R_f = 0.3 (PE/EA = 4/1); White solid, m.p. 106 – 108 ºC; $[\alpha]^{20}_{D}$ = +75 (*c* = 0.1, EA).

^{1}H NMR (300 MHz, $CDCl_3$) δ 8.03 (d, *J* = 7.7 Hz, 1H), 7.50 (td, *J* = 7.4, 1.5 Hz, 1H), 7.39 (td, *J* = 7.6, 1.4 Hz, 1H), 7.20 (d, *J* = 7.6 Hz, 1H), 3.79 (t, *J* = 5.1 Hz, 1H), 3.53 – 3.45 (m, 4H), 2.73 (dd, *J* = 14.8, 10.7 Hz, 1H), 2.48 (d, *J* = 11.9 Hz, 1H), 2.35 (s, 3H), 2.32 – 2.19 (m, 2H).

^{13}C NMR (75 MHz, $CDCl_3$) δ 206.0, 197.3, 171.9, 144.7, 133.9, 130.4, 128.2, 127.9, 68.7, 51.8, 48.4, 45.7, 42.4, 28.6, 28.5.

HRMS (ESI) calcd. for $[C_{16}H_{16}O_4+H]^+$ 273.1121, found 273.1126.

HPLC: Daicel Chiralcel OD-H, *n*-hexane/isopropanol 98/2, flow rate = 1.0 mL/min, uv-vis λ = 254 nm, t_{R1} = 32.8 min (minor), t_{R2} = 34.3 min (major).

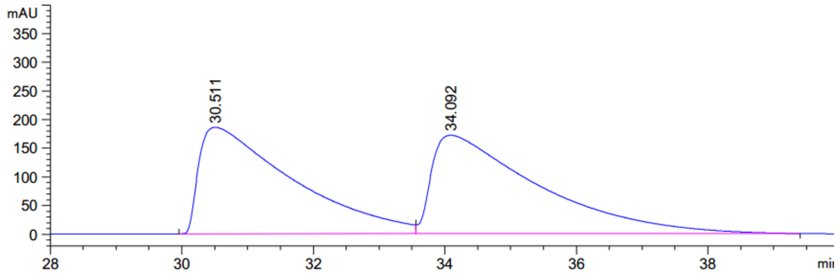

Peak #	RetTime [min]	Type	Width [min]	Area [mAU*s]	Height [mAU]	Area %
1	30.511	BV	1.3383	1.84118e4	185.76540	48.7794
2	34.092	VB	1.5134	1.93332e4	171.73088	51.2206

Supplementary Figure 1. HPLC Chromatograph of racemic **3aa**

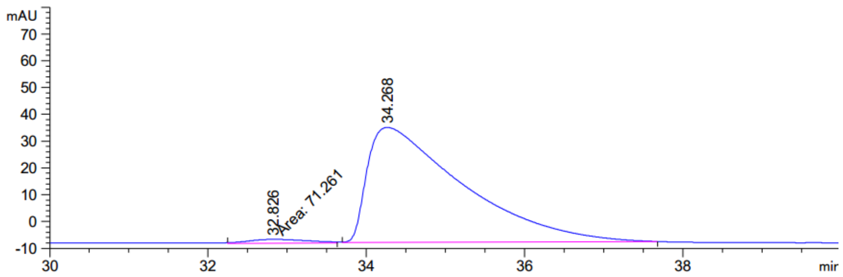

Peak #	RetTime [min]	Type	Width [min]	Area [mAU*s]	Height [mAU]	Area %
1	32.826	MM	0.8626	71.26096	1.37691	1.9404
2	34.268	VB	1.1488	3601.25073	42.92413	98.0596

Supplementary Figure 2. HPLC Chromatograph of chiral **3aa**

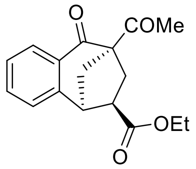

ethyl (5S,6R,8R)-8-acetyl-9-oxo-6,7,8,9-tetrahydro-5H-5,8-methanobenzo[7]annulene-6-carboxylate (3ab)

23.0 mg, 80% yield, >20:1 dr, 96% *ee*; R_f = 0.4 (PE/EA = 4/1); Colorless oil; $[\alpha]_D^{20}$ = +44 (*c* = 0.1, EA).

^{1}H NMR (300 MHz, $CDCl_3$) δ 8.03 (d, *J* = 7.6 Hz, 1H), 7.49 (td, *J* = 7.4, 1.5 Hz, 1H), 7.39 (dt, *J* = 7.5, 3.8 Hz, 1H), 7.22 (d, *J* = 7.5 Hz, 1H), 3.95 – 3.84 (m, 2H), 3.80 (t, *J* = 4.7, 4.2 Hz, 1H), 3.47 (dt, *J* = 10.7, 6.3 Hz, 1H), 2.72 (dd, *J* = 14.8, 10.7 Hz, 1H), 2.48 (d, *J* = 11.9 Hz, 1H), 2.35 – 2.19 (m, 5H), 1.10 (t, *J* = 7.1 Hz, 3H).

^{13}C NMR (75 MHz, $CDCl_3$) δ 206.1, 197.4, 171.4, 144.7, 133.8, 130.4, 128.1, 128.1, 127.8, 68.7, 60.7, 48.5, 45.7, 42.4, 28.6, 14.1.

HRMS (ESI) calcd. for $[C_{17}H_{18}O_4+H]^+$ 287.1278, found 287.1281.

HPLC: Daicel Chiralcel OD-H, *n*-hexane/isopropanol 90/10, flow rate = 1.0 mL/min, uv-vis λ = 254 nm, t_{R1} = 12.6 min (minor), t_{R2} = 13.7 min (major).

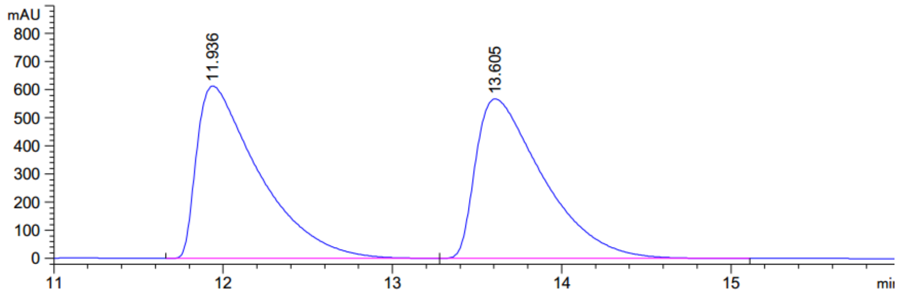

Peak #	RetTime [min]	Type	Width [min]	Area [mAU*s]	Height [mAU]	Area %
1	11.936	BV	0.3739	1.57760e4	613.65405	50.1472
2	13.605	VB	0.4102	1.56834e4	567.88745	49.8528

Supplementary Figure 3. HPLC Chromatograph of racemic **3ab**

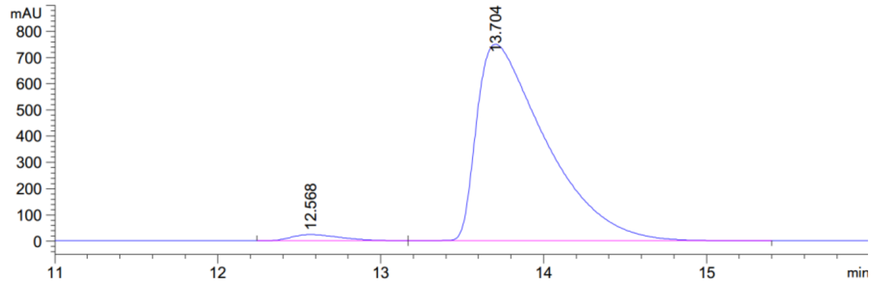

Peak #	RetTime [min]	Type	Width [min]	Area [mAU*s]	Height [mAU]	Area %
1	12.568	VV	0.3297	504.74411	23.58572	2.2334
2	13.704	VB	0.4360	2.20949e4	749.23541	97.7666

Supplementary Figure 4. HPLC Chromatograph of chiral **3ab**

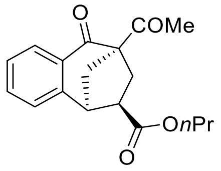

propyl (5S,6R,8R)-8-acetyl-9-oxo-6,7,8,9-tetrahydro-5H-5,8-methanobenzo[7]annulene-6-carboxylate (3ac)

20.4 mg, 68% yield, 13:1 dr, 95% *ee*; R_f = 0.4 (PE/EA = 4/1); White solid, m.p. 58 – 60 ºC; $[\alpha]_D^{20}$ = +112 (*c* = 0.1, EA).

^{1}H NMR (300 MHz, $CDCl_3$) δ 8.03 (dd, *J* = 7.7, 1.4 Hz, 1H), 7.49 (td, *J* = 7.5, 1.5 Hz, 1H), 7.38 (td, *J* = 7.6, 1.3 Hz, 1H), 7.22 (dd, *J* = 7.5, 1.2 Hz, 1H), 3.86 – 3.70 (m, 3H), 3.52 – 3.44 (m, 1H), 2.73 (dd, *J* = 14.8, 10.6 Hz, 1H), 2.48 (d, *J* = 11.9 Hz, 1H), 2.35 (s, 3H), 2.31 – 2.19 (m, 2H), 1.56 – 1.43 (m, 2H), 0.84 (t, *J* = 7.4 Hz, 3H).

^{13}C NMR (75 MHz, $CDCl_3$) δ 206.1, 197.4, 171.5, 144.7, 133.8, 130.4, 128.1, 127.8, 68.7, 66.4, 48.6, 45.8, 42.4, 28.6, 28.6, 21.8, 10.4.

HRMS (ESI) calcd. for $[C_{18}H_{20}O_4+H]^+$ 301.1434, found 301.1433.

HPLC: Daicel Chiralcel OD-H, *n*-hexane/isopropanol 96/4, flow rate = 1.0 mL/min, uv-vis λ = 254 nm, t_{R1} = 20.6 min (minor), t_{R2} = 22.0 min (major).

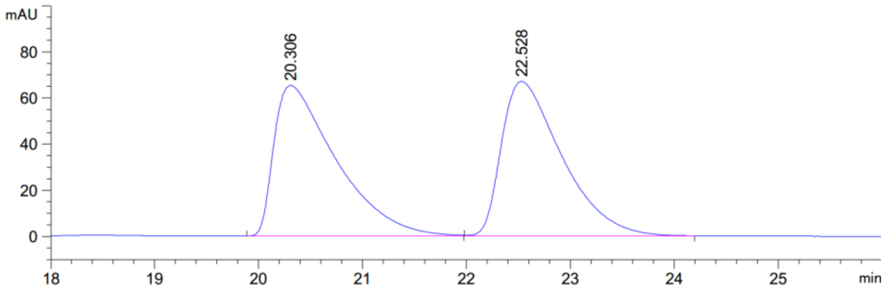

Peak #	RetTime [min]	Type	Width [min]	Area [mAU*s]	Height [mAU]	Area %
1	20.306	BB	0.6019	2717.42676	65.24121	50.0301
2	22.528	BB	0.6089	2714.16113	66.95555	49.9699

Supplementary Figure 5. HPLC Chromatograph of racemic **3ac**

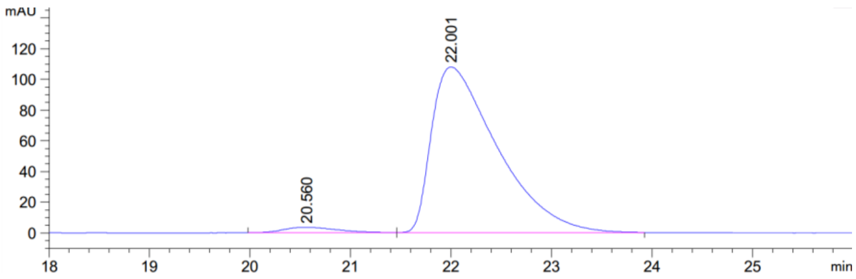

Peak #	RetTime [min]	Type	Width [min]	Area [mAU*s]	Height [mAU]	Area %
1	20.560	BV	0.4631	132.65659	3.58981	2.6244
2	22.001	VB	0.6723	4922.02490	107.87014	97.3756

Supplementary Figure 6. HPLC Chromatograph of chiral **3ac**

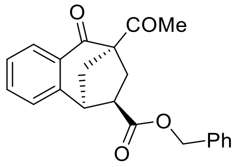

benzyl (5S,6R,8R)-8-acetyl-9-oxo-6,7,8,9-tetrahydro-5H-5,8-methanobenzo[7]annulene-6-carboxylate (3ad)

21.8 mg, 63% yield, >20:1 dr, 95% *ee*; R_f = 0.4 (PE/EA = 4/1); White solid, m.p. 70 – 72 ºC; $[\alpha]_D^{20}$ = +97 (*c* = 0.1, EA).

^{1}H NMR (300 MHz, $CDCl_3$) δ 8.06 – 7.98 (m, 1H), 7.38 – 7.31 (m, 5H), 7.21 (d, *J* = 1.3 Hz, 2H), 7.05 – 6.96 (m, 1H), 4.88 (s, 2H), 3.78 (t, *J* = 4.8 Hz, 1H), 3.55 – 3.47 (m, 1H), 2.74 (dd, *J* = 14.8, 10.7 Hz, 1H), 2.46 (d, *J* = 11.5 Hz, 1H), 2.34 (s, 3H), 2.28 (dd, *J* = 7.6, 6.1 Hz, 2H), 2.20 (dd, *J* = 11.9, 4.2 Hz, 1H).

^{13}C NMR (75 MHz, $CDCl_3$) δ 206.0, 197.3, 171.3, 144.4, 135.4, 133.9, 130.3, 128.7, 128.6, 128.5, 128.1, 127.8, 68.6, 66.7, 48.5, 45.7, 42.4, 28.6, 28.5.

HRMS (ESI) calcd. for $[C_{22}H_{20}O_4+H]^+$ 349.1434, found 349.1442.

HPLC: Daicel Chiralcel IB-3, *n*-hexane/isopropanol 96/4, flow rate = 1.0 mL/min, uv-vis λ = 254 nm, t_{R1} = 23.5 min (minor), t_{R2} = 25.0 min (major).

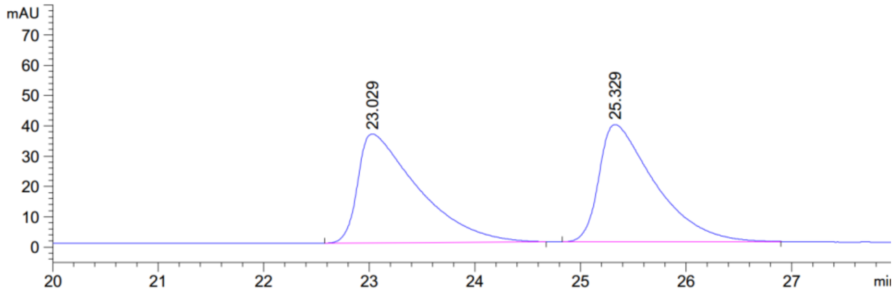

Peak #	RetTime [min]	Type	Width [min]	Area [mAU*s]	Height [mAU]	Area %
1	23.029	BB	0.5577	1430.27234	35.98888	50.0810
2	25.329	BB	0.5297	1425.64465	38.70908	49.9190

Supplementary Figure 7. HPLC Chromatograph of racemic **3ad**

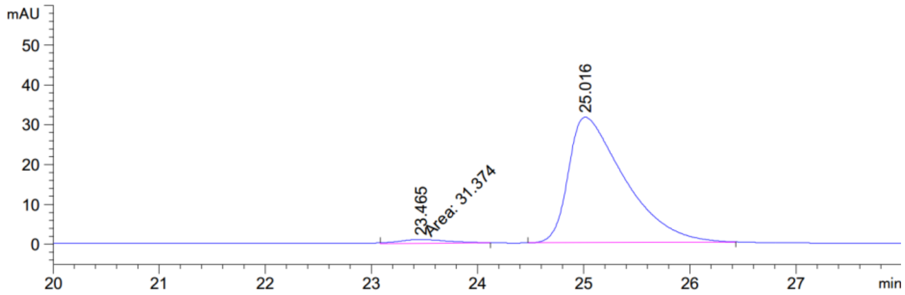

Peak #	RetTime [min]	Type	Width [min]	Area [mAU*s]	Height [mAU]	Area %
1	23.465	MM	0.5408	31.37397	9.66963e-1	2.6259
2	25.016	BB	0.5327	1163.41479	31.51668	97.3741

Supplementary Figure 8. HPLC Chromatograph of chiral **3ad**

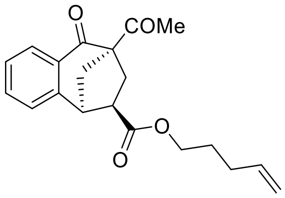

pent-4-en-1-yl (5S,6R,8R)-8-acetyl-9-oxo-6,7,8,9-tetrahydro-5H-5,8-methanobenzo[7]annulene-6-carboxylate (3ae)

24.4 mg, 75% yield, >20:1 dr, 88% ee; R_f = 0.4 (PE/EA = 4/1); Colorless oil; $[\alpha]_D^{20}$ = +112 (*c* = 0.1, EA).

^{1}H NMR (300 MHz, $CDCl_3$) δ 8.04 (dd, *J* = 7.7, 1.5 Hz, 1H), 7.49 (td, *J* = 7.4, 1.5 Hz, 1H), 7.38 (td, *J* = 7.5, 1.3 Hz, 1H), 7.21 (dd, *J* = 7.5, 1.3 Hz, 1H), 5.74 (ddt, *J* = 17.0, 10.4, 6.6 Hz, 1H), 5.04 – 4.99 (m, 1H), 4.97 (t, *J* = 1.5 Hz, 1H), 3.93 – 3.76 (m, 3H), 3.48 (ddd, *J* = 10.6, 6.8, 5.7 Hz, 1H), 2.73 (dd, *J* = 14.8, 10.6 Hz, 1H), 2.49 (d, *J* = 11.8 Hz, 1H), 2.35 (s, 3H), 2.30 – 2.19 (m, 2H), 2.03 – 1.95 (m, 2H), 1.61 – 1.51 (m, 2H).

^{13}C NMR (75 MHz, $CDCl_3$) δ 206.0, 197.4, 171.5, 144.7, 137.3, 133.9, 130.4, 128.1, 128.1, 127.8, 115.4, 68.7, 64.3, 48.6, 45.8, 42.4, 29.9, 28.6, 27.6.

HRMS (ESI) calcd. for $[C_{20}H_{22}O_4+H]^+$ 327.1591, found 327.1596.

HPLC: Daicel Chiralcel AS-H, *n*-hexane/isopropanol 95/5, flow rate = 1.0 mL/min, uv-vis λ = 254 nm, t_{R1} = 23.8 min (minor), t_{R2} = 25.8 min (major).

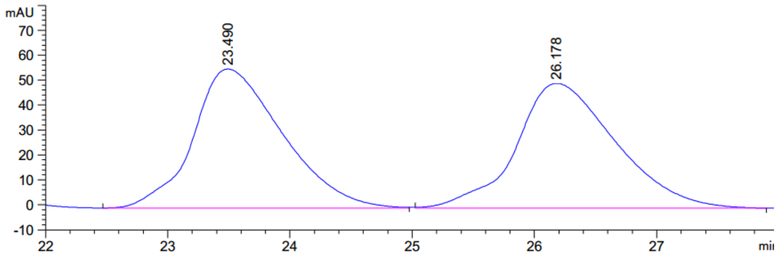

Peak #	RetTime [min]	Type	Width [min]	Area [mAU*s]	Height [mAU]	Area %
1	23.490	VB	0.7609	2841.68945	55.73225	50.0886
2	26.178	BB	0.8339	2831.63989	50.06274	49.9114

Supplementary Figure 9. HPLC Chromatograph of racemic **3ae**

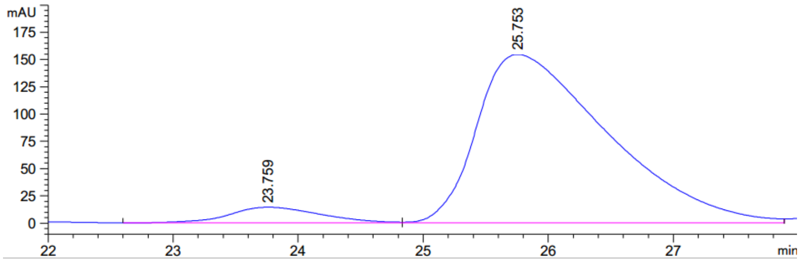

Peak #	RetTime [min]	Type	Width [min]	Area [mAU*s]	Height [mAU]	Area %
1	23.759	VV	0.7184	729.22327	14.36050	6.0884
2	25.753	VV	1.0677	1.12479e4	154.44687	93.9116

Supplementary Figure 10. HPLC Chromatograph of chiral **3ae**

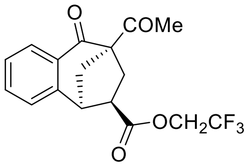

2,2,2-trifluoroethyl (5S,6R,8R)-8-acetyl-9-oxo-6,7,8,9-tetrahydro-5H-5,8-methanobenzo[7]annulene-6-carboxylate (3af)

23.8 mg, 70% yield, >20:1 dr, 96% *ee*; R_f = 0.4 (PE/EA = 4/1); White solid, m.p. 58 – 60 °C; $[\alpha]_D^{20}$ = +86 (*c* = 0.1, EA).

^{1}H NMR (400 MHz, $CDCl_3$) δ 8.05 (dd, *J* = 7.8, 1.4 Hz, 1H), 7.51 (td, *J* = 7.5, 1.5 Hz, 1H), 7.41 (td, *J* = 7.6, 1.2 Hz, 1H), 7.21 (d, *J* = 7.5 Hz, 1H), 4.22 (qd, *J* = 8.5, 0.9 Hz, 2H), 3.86 (t, *J* = 4.9 Hz, 1H), 3.62 – 3.56 (m, 1H), 2.80 (dd, *J* = 14.9, 10.7 Hz, 1H), 2.53 (d, *J* = 11.9 Hz, 1H), 2.35 (s, 3H), 2.30 – 2.21 (m, 2H).

^{13}C NMR (101 MHz, $CDCl_3$) δ 205.6, 197.0, 170.1, 144.0, 134.1, 130.3, 128.5, 128.1, 127.8, 122.8 (d, *J* = 276.5 Hz), 68.6, 60.7 (q, *J* = 36.7 Hz), 48.1, 45.7, 42.5, 28.5 (d, *J* = 4.2 Hz).

^{19}F NMR (282 MHz, $CDCl_3$) δ -58.11.

HRMS (ESI) calcd. for $[C_{17}H_{15}F_3O_4+H]^+$ 341.0995, found 341.1002.

HPLC: Daicel Chiralcel IB-3, *n*-hexane/isopropanol 96/4, flow rate = 0.5 mL/min, uv-vis λ = 254 nm, t_{R1} = 30.4 min (major), t_{R2} = 33.6 min (minor).

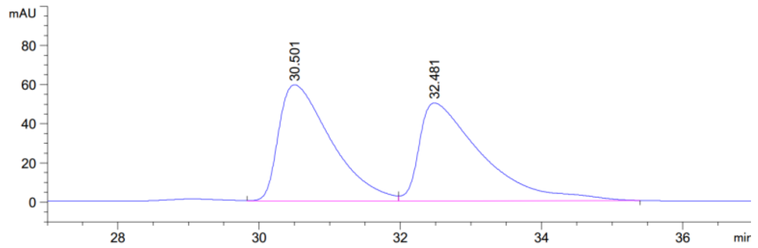

Peak #	RetTime [min]	Type	Width [min]	Area [mAU*s]	Height [mAU]	Area %
1	30.501	VV	0.7684	3056.34155	59.19141	49.9782
2	32.481	VB	0.8611	3059.01318	49.90491	50.0218

Supplementary Figure 11. HPLC Chromatograph of racemic **3af**

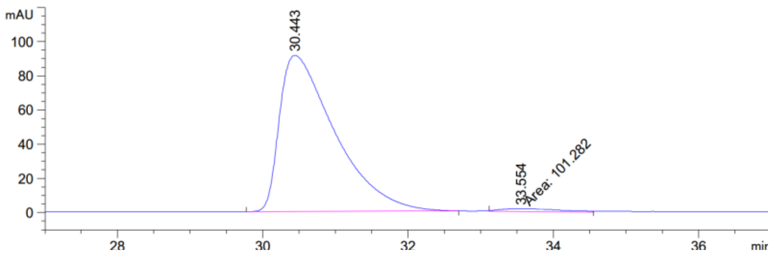

Peak #	RetTime [min]	Type	Width [min]	Area [mAU*s]	Height [mAU]	Area %
1	30.443	BB	0.7809	4843.04590	91.29739	97.9516
2	33.554	MM	0.9250	101.28172	1.82491	2.0484

Supplementary Figure 12. HPLC Chromatograph of chiral **3af**

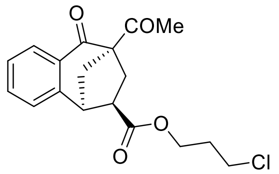

3-chloropropyl (5S,6R,8R)-8-acetyl-9-oxo-6,7,8,9-tetrahydro-5H-5,8-methanobenzo[7]annulene-6-carboxylate (3ag)

21.2 mg, 63% yield, >20:1 dr, 94% ee; R_f = 0.3 (PE/EA = 4/1); Colorless oil; $[\alpha]_D^{20}$ = +68 (*c* = 0.1, EA).

^{1}H NMR (400 MHz, $CDCl_3$) δ 8.05 (dd, *J* = 7.73, 1.42 Hz, 1H), 7.51 (td, *J* = 7.49, 1.37 Hz, 1H), 7.40 (td, *J* = 7.56, 1.27 Hz, 2H), 7.21 (dd, *J* = 7.52, 1.25 Hz, 1H), 4.04 (dt, *J* = 11.67, 5.84 Hz, 1H), 3.93 (ddd, *J* = 11.14, 6.86, 5.59 Hz, 1H), 3.80 (t, *J* = 5.06 Hz, 1H), 3.54 – 3.38 (m, 3H), 2.74 (dd, *J* = 14.85, 10.62 Hz, 1H), 2.50 (d, *J* = 11.83 Hz, 1H), 2.35 (s, 2H), 2.29 – 2.20 (m, 2H), 1.94 – 1.89 (m, 2H).

^{13}C NMR (101 MHz, $CDCl_3$) δ 205.9, 197.3, 171.3, 144.5, 134.0, 130.4, 128.3, 128.0, 127.9, 68.7, 61.5, 48.6, 45.9, 42.4, 41.0, 31.4, 28.6.

HRMS (ESI) calcd. for $[C_{18}H_{19}ClO_4+H]^+$ 335.1045, found 335.1049.

HPLC: Daicel Chiralcel OD-H, *n*-hexane/isopropanol 95/5, flow rate = 1.0 mL/min, uv-vis λ = 254 nm, t_{R1} = 32.3 min (minor), t_{R2} = 34.5 min (major).

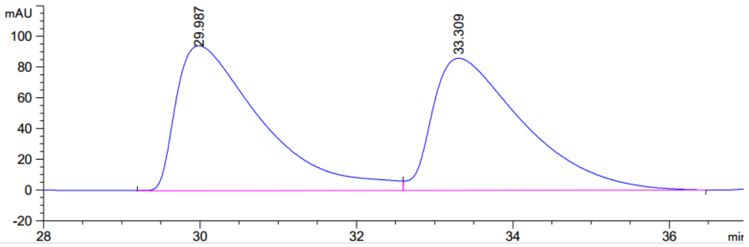

Peak #	RetTime [min]	Type	Width [min]	Area [mAU*s]	Height [mAU]	Area %
1	29.987	BV	1.0742	7131.60547	94.31711	50.6947
2	33.309	VB	1.1167	6936.14697	86.02297	49.3053

Supplementary Figure 13. HPLC Chromatograph of racemic **3ag**

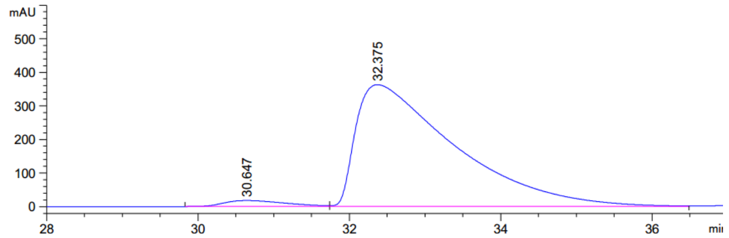

Peak #	RetTime [min]	Type	Width [min]	Area [mAU*s]	Height [mAU]	Area %
1	30.647	BV	0.7823	1016.98590	17.96932	2.9846
2	32.375	VB	1.2943	3.30571e4	363.03918	97.0154

Supplementary Figure 14. HPLC Chromatograph of chiral **3ag**

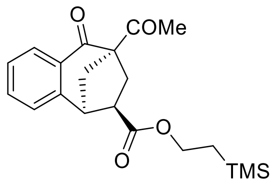

2-(trimethylsilyl)ethyl (5S,6R,8R)-8-acetyl-9-oxo-6,7,8,9-tetrahydro-5H-5,8-methanobenzo[7]annulene-6-carboxylate (3ah)

19.0 mg, 53% yield, >20:1 dr, 88% ee; R_f = 0.7 (PE/EA = 4/1); White solid, m.p. 38 – 40 ºC; $[\alpha]_D^{20}$ = +96 (*c* = 0.1, EA).

^{1}H NMR (300 MHz, $CDCl_3$) δ 8.23 (dd, *J* = 7.7, 1.5 Hz, 1H), 7.69 (td, *J* = 7.5, 1.5 Hz, 1H), 7.58 (td, *J* = 7.5, 1.3 Hz, 1H), 7.43 (d, *J* = 7.7 Hz, 1H), 4.19 – 4.05 (m, 2H), 3.99 (t, *J* = 4.8 Hz, 1H), 3.65 (ddd, *J* = 10.6, 6.8, 5.7 Hz, 1H), 2.92 (dd, *J* = 14.8, 10.6 Hz, 1H), 2.68 (d, *J* = 11.8 Hz, 1H), 2.55 (s, 3H), 2.48 (td, *J* = 8.1, 6.9, 1.3 Hz, 1H), 2.41 (dd, *J* = 11.8, 4.1 Hz, 1H), 1.01 (t, *J* = 8.8 Hz, 2H), 0.20 (s, 9H).

^{13}C NMR (75 MHz, $CDCl_3$) δ 206.1, 197.4, 171.6, 144.7, 133.7, 130.4, 128.2, 128.1, 127.8, 68.7, 63.0, 48.6, 45.8, 42.4, 28.6, 28.6, 17.4, -1.6.

HRMS (ESI) calcd. for $[C_{20}H_{26}O_4Si+H]^+$ 359.1673 , found 359.1679.

HPLC: Daicel Chiralcel AD-H, *n*-hexane/isopropanol 95/5, flow rate = 1.0 mL/min, uv-vis λ = 254 nm, t_{R1} = 8.7 min (minor), t_{R2} = 10.1 min (major).

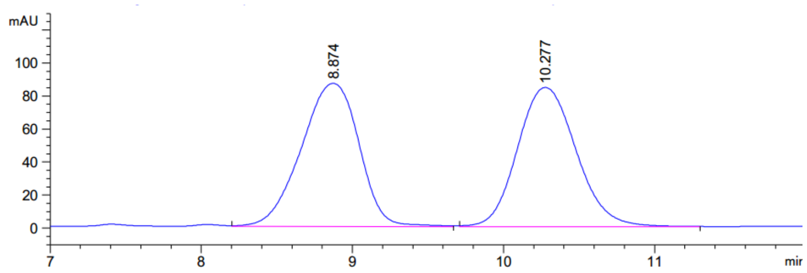

Peak #	RetTime [min]	Type	Width [min]	Area [mAU*s]	Height [mAU]	Area %
1	8.874	VB	0.4170	2310.67871	86.67575	50.4410
2	10.277	BB	0.4244	2270.27344	84.23508	49.5590

Supplementary Figure 15. HPLC Chromatograph of racemic **3ah**

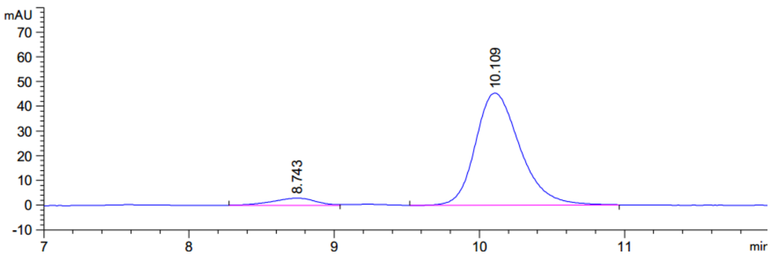

Peak #	RetTime [min]	Type	Width [min]	Area [mAU*s]	Height [mAU]	Area %
1	8.743	BV	0.3079	61.66529	2.94993	6.0147
2	10.109	VB	0.3257	963.57318	45.37537	93.9853

Supplementary Figure 16. HPLC Chromatograph of chiral **3ah**

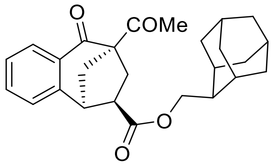

adamantan-2-yl)methyl (5S,6R,8R)-8-acetyl-9-oxo-6,7,8,9-tetrahydro-5H-5,8-methanobenzo[7]annulene-6-carboxylate (3ai)

29.5 mg, 73% yield, >20:1 dr, 93% *ee*; R_f = 0.5 (PE/EA = 4/1); White solid, m.p. 84 – 86 ºC; $[\alpha]_D^{20}$ = +92 (*c* = 0.1, EA).

^{1}H NMR (400 MHz, $CDCl_3$) δ 8.04 (dd, *J* = 7.7, 1.4 Hz, 1H), 7.50 (td, *J* = 7.5, 1.5 Hz, 1H), 7.38 (td, *J* = 7.6, 1.2 Hz, 1H), 7.22 (d, *J* = 7.5 Hz, 1H), 3.82 (t, *J* = 4.9 Hz, 1H), 3.54 – 3.49 (m, 2H), 3.26 (d, *J* = 10.7 Hz, 1H), 2.75 (dd, *J* = 14.9, 10.6 Hz, 1H), 2.49 (d, *J* = 11.8 Hz, 1H), 2.35 (s, 3H), 2.29 – 2.20 (m, 2H), 1.94 (s, 3H), 1.70 (d, *J* = 12.7 Hz, 3H), 1.59 (d, *J* = 12.0 Hz, 3H), 1.39 (d, *J* = 12.1 Hz, 3H), 1.32 (d, *J* = 11.3 Hz, 3H).

^{13}C NMR (101 MHz, $CDCl_3$) δ 206.2, 197.5, 171.6, 144.7, 134.0, 130.3, 128.2, 128.1, 127.8, 74.5, 68.8, 48.8, 45.9, 42.4, 39.2, 36.8, 32.8, 28.6, 28.4, 27.9.

HRMS (ESI) calcd. for $[C_{26}H_{30}O_4+H]^+$ 407.2217, found 407.2216.

HPLC: Daicel Chiralcel AD-H, *n*-hexane/isopropanol 96/4, flow rate = 1.0 mL/min, uv-vis λ = 254 nm, t_{R1} = 15.0 min (minor), t_{R2} = 17.3 min (major).

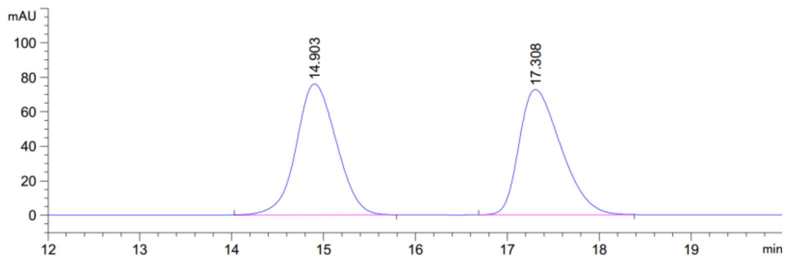

Peak #	RetTime [min]	Type	Width [min]	Area [mAU*s]	Height [mAU]	Area %
1	14.903	BB	0.4733	2311.38135	75.88922	50.0660
2	17.308	BB	0.4941	2305.28296	72.67100	49.9340

Supplementary Figure 17. HPLC Chromatograph of racemic **3ai**

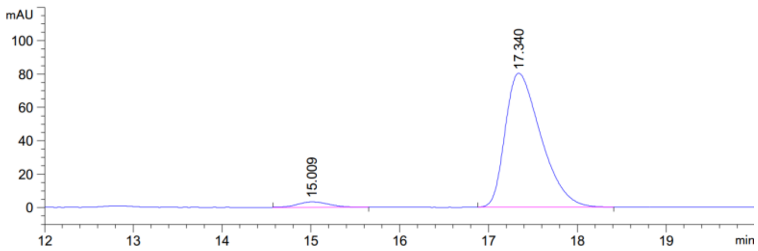

Peak #	RetTime [min]	Type	Width [min]	Area [mAU*s]	Height [mAU]	Area %
1	15.009	BB	0.3552	79.88810	3.26754	3.4342
2	17.340	BB	0.4284	2246.35889	80.28847	96.5658

Supplementary Figure 18. HPLC Chromatograph of chiral **3ai**

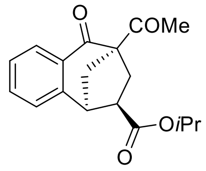

isopropyl (5S,6R,8R)-8-acetyl-9-oxo-6,7,8,9-tetrahydro-5H-5,8-methanobenzo[7]annulene-6-carboxylate (3aj)

19.2 mg, 64% yield, >20:1 dr, 96% *ee*; R_f = 0.5 (PE/EA = 4/1); White solid, m.p. 81 – 83 ºC; $[\alpha]_D^{20}$ = +128 (*c* = 0.1, EA).

^{1}H NMR (300 MHz, $CDCl_3$) δ 8.04 (d, *J* = 7.7 Hz, 1H), 7.49 (td, *J* = 7.4, 1.0 Hz, 1H), 7.38 (t, *J* = 7.3 Hz, 1H), 7.24 (d, *J* = 7.5 Hz, 1H), 4.73 (p, *J* = 6.3 Hz, 1H), 3.79 (t, *J* = 5.0 Hz, 1H), 3.43 (dt, *J* = 10.7, 6.3 Hz, 1H), 2.71 (dd, *J* = 14.8, 10.6 Hz, 1H), 2.47 (d, *J* = 11.7 Hz, 1H), 2.35 (s, 3H), 2.26 – 2.18 (m, 2H), 1.11 (d, *J* = 6.3 Hz, 3H), 0.98 (d, *J* = 6.3 Hz, 3H).

^{13}C NMR (75 MHz, $CDCl_3$) δ 206.2, 197.5, 170.9, 144.6, 133.7, 130.4, 128.5, 128.1, 127.8, 68.7, 68.4, 48.6, 45.7, 42.6, 28.7, 28.6, 21.9, 21.7.

HRMS (ESI) calcd. for $[C_{18}H_{20}O_4+H]^+$ 301.1434, found 301.1437.

HPLC: Daicel Chiralcel OD-H, *n*-hexane/isopropanol 98/2, flow rate = 0.5 mL/min, uv-vis λ = 254 nm, t_{R1} = 40.7 min (minor), t_{R2} = 42.2 min (major).

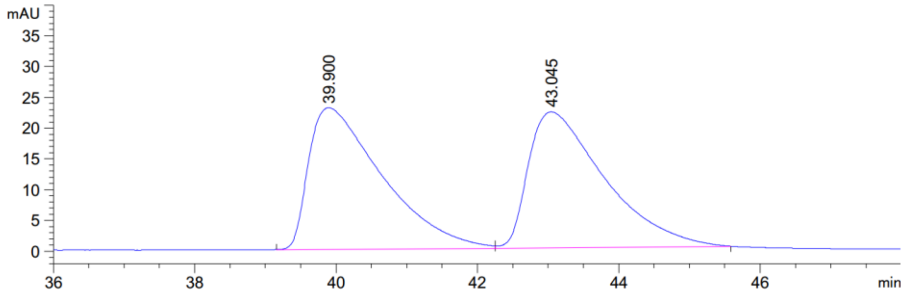

Peak #	RetTime [min]	Type	Width [min]	Area [mAU*s]	Height [mAU]	Area %
1	39.900	BB	0.9762	1664.37781	23.00264	50.0794
2	43.045	BB	1.0463	1659.09985	22.08738	49.9206

Supplementary Figure 19. HPLC Chromatograph of racemic **3aj**

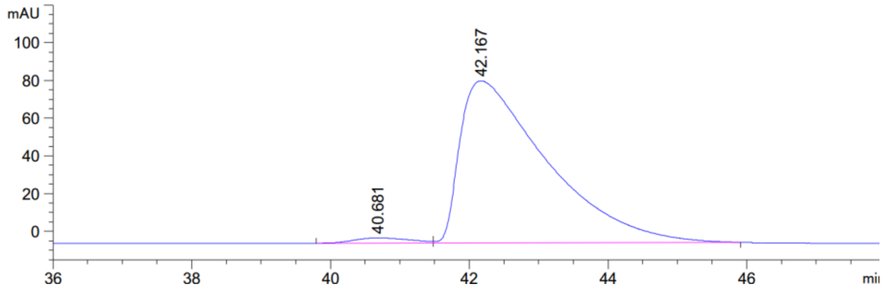

Peak #	RetTime [min]	Type	Width [min]	Area [mAU*s]	Height [mAU]	Area %
1	40.681	BV	0.6532	150.07953	2.73054	1.9632
2	42.167	VB	1.2155	7494.48096	85.92975	98.0368

Supplementary Figure 20. HPLC Chromatograph of chiral **3aj**

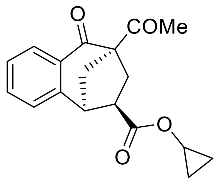

cyclopropyl (5S,6R,8R)-8-acetyl-9-oxo-6,7,8,9-tetrahydro-5H-5,8-methanobenzo[7]annulene-6-carboxylate (3ak)

16.0 mg, 54% yield, >20:1 dr, 94% *ee*; R_f = 0.5 (PE/EA = 4/1); White solid, m.p. 84 – 86 ºC; $[\alpha]_D^{20}$ = +60 (*c* = 0.1, EA).

^{1}H NMR (400 MHz, $CDCl_3$) δ 8.03 (dd, *J* = 7.8, 1.4 Hz, 1H), 7.51 (td, *J* = 7.5, 1.5 Hz, 1H), 7.40 (td, *J* = 7.6, 1.3 Hz, 1H), 7.20 (d, *J* = 7.5 Hz, 1H), 3.86 – 3.81 (m, 1H), 3.76 (t, *J* = 5.0 Hz, 1H), 3.46 – 3.40 (m, 1H), 2.72 (dd, *J* = 14.9, 10.7 Hz, 1H), 2.46 (dd, *J* = 12.0, 1.4 Hz, 1H), 2.34 (s, 3H), 2.27 – 2.23 (m, 1H), 2.20 (dd, *J* = 11.9, 4.2 Hz, 1H), 0.60 – 0.54 (m, 2H), 0.52 – 0.47 (m, 1H), 0.45 – 0.40 (m, 1H).

^{13}C NMR (101 MHz, $CDCl_3$) δ 205.9, 197.3, 172.2, 144.5, 133.8, 130.4, 128.2, 128.2, 127.8, 68.7, 49.2, 48.3, 45.7, 42.5, 28.6, 28.6, 4.9, 4.8.

HRMS (ESI) calcd. for $[C_{18}H_{20}O_4+H]^+$ 299.1278, found 299.1280.

HPLC: Daicel Chiralcel OD-H, *n*-hexane/isopropanol 97/3, flow rate = 1.0 mL/min, uv-vis λ = 254 nm, t_{R1} = 29.8 min (minor), t_{R2} = 37.4 min (major).

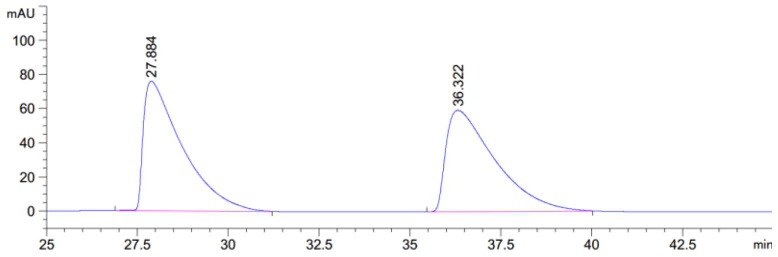

Peak #	RetTime [min]	Type	Width [min]	Area [mAU*s]	Height [mAU]	Area %
1	27.884	BB	1.0422	5665.87012	75.96545	50.4414
2	36.322	BB	1.2721	5566.69873	59.35973	49.5586

Supplementary Figure 21. HPLC Chromatograph of racemic **3ak**

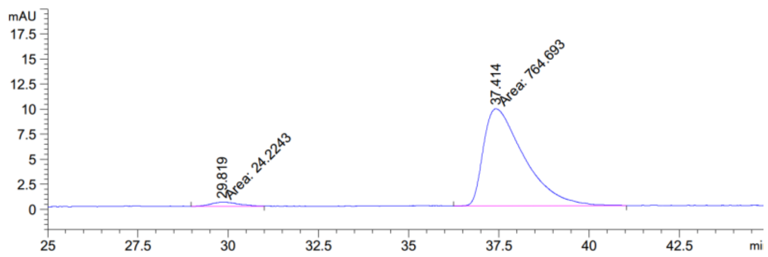

Peak #	RetTime [min]	Type	Width [min]	Area [mAU*s]	Height [mAU]	Area %
1	29.819	MM	0.9501	24.22427	4.24952e-1	3.0706
2	37.414	MM	1.3117	764.69250	9.71647	96.9294

Supplementary Figure 22. HPLC Chromatograph of chiral **3ak**

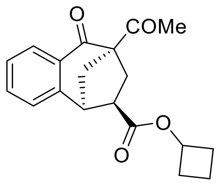

cyclobutyl (5S,6R,8R)-8-acetyl-9-oxo-6,7,8,9-tetrahydro-5H-5,8-methanobenzo[7]annulene-6-carboxylate (3al)

20.2 mg, 65% yield, >20:1 dr, 93% *ee*; R_f = 0.5 (PE/EA = 4/1); White solid, m.p. 104 – 106 ºC; $[\alpha]^{20}_{D}$ = +146 (c = 0.1, EA).

^{1}H NMR (300 MHz, $CDCl_3$) δ 8.03 (d, J = 7.7 Hz, 1H), 7.50 (td, J = 7.5, 1.5 Hz, 1H), 7.39 (td, J = 7.6, 1.3 Hz, 1H), 7.23 (d, J = 7.6 Hz, 1H), 4.70 (p, J = 7.5 Hz, 1H), 3.79 (t, J = 5.0 Hz, 1H), 3.48 – 3.40 (m, 1H), 2.71 (dd, J = 14.8, 10.6 Hz, 1H), 2.48 (d, J = 11.8 Hz, 1H), 2.35 (s, 3H), 2.29 – 2.13 (m, 4H), 1.97 – 1.88 (m, 1H), 1.84 – 1.69 (m, 2H), 1.56 – 1.47 (m, 1H).

^{13}C NMR (75 MHz, $CDCl_3$) δ 206.1, 197.4, 170.8, 144.6, 133.7, 130.4, 128.3, 128.1, 127.8, 69.1, 68.7, 48.4, 45.7, 42.5, 30.3, 30.1, 28.6, 28.6, 13.5.

HRMS (ESI) calcd. for $[C_{19}H_{20}O_4+H]^+$ 313.1434, found 313.1439.

HPLC: Daicel Chiralcel AD-H, *n*-hexane/isopropanol 98.5/1.5, flow rate = 1.0 mL/min, uv-vis λ = 254 nm, t_{R1} = 24.5 min (major), t_{R2} = 26.7 min (minor).

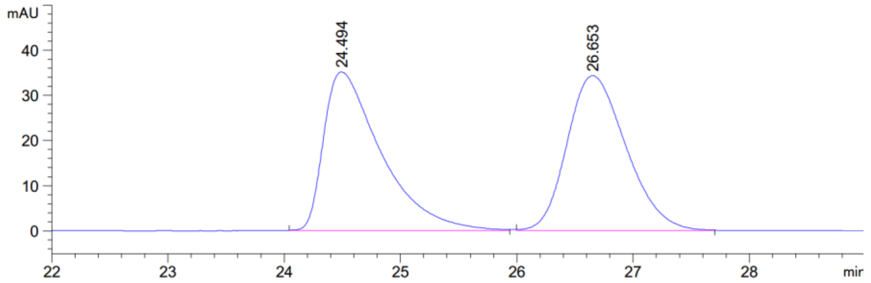

Peak #	RetTime [min]	Type	Width [min]	Area [mAU*s]	Height [mAU]	Area %
1	24.494	BB	0.5074	1199.91272	35.04594	49.8880
2	26.653	BB	0.5486	1205.29968	34.24546	50.1120

Supplementary Figure 23. HPLC Chromatograph of racemic **3al**

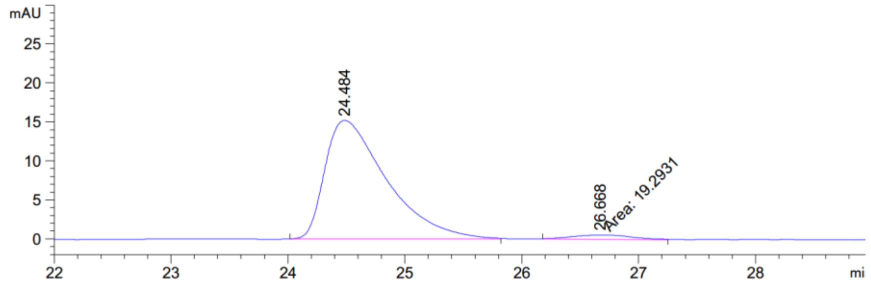

Peak #	RetTime [min]	Type	Width [min]	Area [mAU*s]	Height [mAU]	Area %
1	24.484	BB	0.5367	556.93372	15.22622	96.6518
2	26.668	MM	0.5806	19.29308	5.53808e-1	3.3482

Supplementary Figure 24. HPLC Chromatograph of chiral **3al**

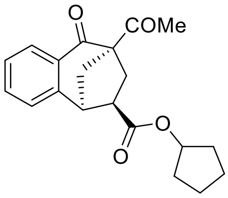

cyclopentyl (5S,6R,8R)-8-acetyl-9-oxo-6,7,8,9-tetrahydro-5H-5,8-methanobenzo[7]annulene-6-carboxylate (3am)

22.6 mg, 69% yield, >20:1 dr, 91% *ee*; R_f = 0.6 (PE/EA = 4/1); White solid, m.p. 68 – 70 ºC; $[\alpha]_D^{20}$ = +144 (*c* = 0.1, EA).

^{1}H NMR (300 MHz, $CDCl_3$) δ 8.03 (d, *J* = 7.7 Hz, 1H), 7.49 (t, *J* = 7.4 Hz, 1H), 7.38 (t, *J* = 7.5 Hz, 1H), 7.22 (d, *J* = 7.5 Hz, 2H), 4.87 (t, *J* = 3.0 Hz, 1H), 3.78 (t, *J* = 5.0 Hz, 1H), 3.43 (dt, *J* = 10.6, 6.3 Hz, 1H), 2.71 (dd, *J* = 14.8, 10.7 Hz, 1H), 2.47 (d, *J* = 11.8 Hz, 1H), 2.34 (s, 3H), 2.29 – 2.17 (m, 2H), 1.73 – 1.50 (m, 6H), 1.31 – 1.27 (m, 1H).

^{13}C NMR (75 MHz, $CDCl_3$) δ 205.9, 197.4, 171.1, 144.7, 133.6, 130.4, 128.3, 128.1, 127.7, 68.7, 48.6, 45.8, 42.5, 32.5, 32.3, 28.7, 28.5, 23.6, 23.6.

HRMS (ESI) calcd. for $[C_{20}H_{22}O_4+H]^+$ 327.1591, found 327.1596.

HPLC: Daicel Chiralcel IA-3, *n*-hexane/isopropanol 98/2, flow rate = 1.0 mL/min, uv-vis λ = 254 nm, t_{R1} = 22.4 min (minor), t_{R2} = 26.3 min (major).

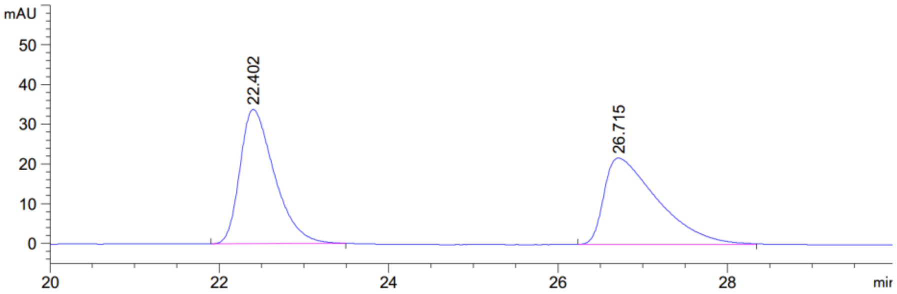

Peak #	RetTime [min]	Type	Width [min]	Area [mAU*s]	Height [mAU]	Area %
1	22.402	BB	0.4334	961.23169	33.84056	50.2986
2	26.715	BB	0.6132	949.81738	21.67833	49.7014

Supplementary Figure 25. HPLC Chromatograph of racemic **3am**

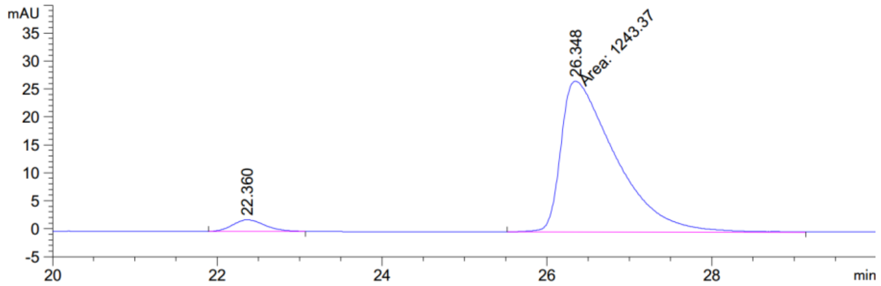

Peak #	RetTime [min]	Type	Width [min]	Area [mAU*s]	Height [mAU]	Area %
1	22.360	BB	0.4045	56.55873	2.07173	4.3509
2	26.348	MM	0.7687	1243.36755	26.95893	95.6491

Supplementary Figure 26. HPLC Chromatograph of chiral **3am**

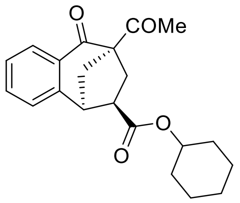

cyclohexyl (5S,6R,8R)-8-acetyl-9-oxo-6,7,8,9-tetrahydro-5H-5,8-methanobenzo[7]annulene-6-carboxylate (3an)

19.8 mg, 58% yield, >20:1 dr, 91% *ee*; R_f = 0.6 (PE/EA = 4/1); White solid, m.p. 60 – 62 ºC; $[\alpha]_D^{20}$ = +44 (*c* = 0.1, EA).

^{1}H NMR (300 MHz, $CDCl_3$) δ 8.03 (d, *J* = 7.7 Hz, 1H), 7.48 (td, *J* = 7.4, 1.5 Hz, 1H), 7.38 (td, *J* = 7.6, 1.3 Hz, 1H), 7.23 (d, *J* = 7.6 Hz, 1H), 4.50 (t, *J* = 8.6 Hz, 1H), 3.79 (t, *J* = 5.0 Hz, 1H), 3.44 (dt, *J* = 10.6, 6.3 Hz, 1H), 2.72 (dd, *J* = 14.8, 10.6 Hz, 1H), 2.47 (d, *J* = 11.8 Hz, 1H), 2.34 (s, 3H), 2.31 – 2.18 (m, 2H), 1.69 – 1.66 (m, 2H), 1.53 – 1.51 (m, 3H), 1.34 – 1.19 (m, 4H), 1.13 – 1.07 (m, 1H).

^{13}C NMR (75 MHz, $CDCl_3$) δ 205.9, 197.4, 170.7, 144.6, 133.7, 130.4, 128.4, 128.0, 127.7, 73.1, 68.7, 48.7, 45.8, 42.5, 31.6, 31.4, 31.4, 28.7, 28.5, 25.2, 23.7, 23.6.

HRMS (ESI) calcd. for $[C_{21}H_{24}O_4+H]^+$ 341.1747, found 341.1752.

HPLC: Daicel Chiralcel AD-H, *n*-hexane/isopropanol 99/1, flow rate = 1.0 mL/min, uv-vis λ = 254 nm, t_{R1} = 27.4 min (minor), t_{R2} = 36.7 min (major).

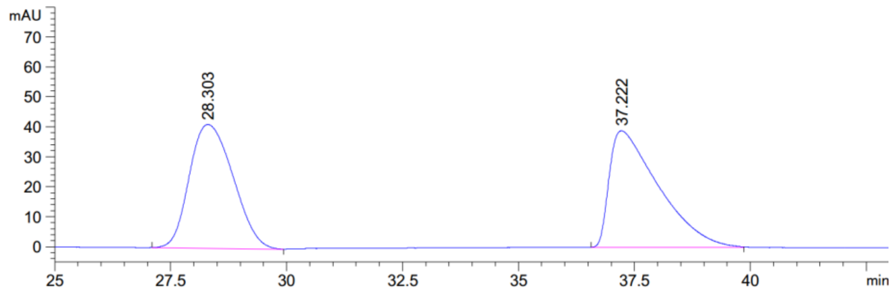

Peak #	RetTime [min]	Type	Width [min]	Area [mAU*s]	Height [mAU]	Area %
1	28.303	BV	1.0360	2705.32593	41.31407	48.5781
2	37.222	BB	1.0018	2863.69727	38.85911	51.4219

Supplementary Figure 27. HPLC Chromatograph of racemic **3an**

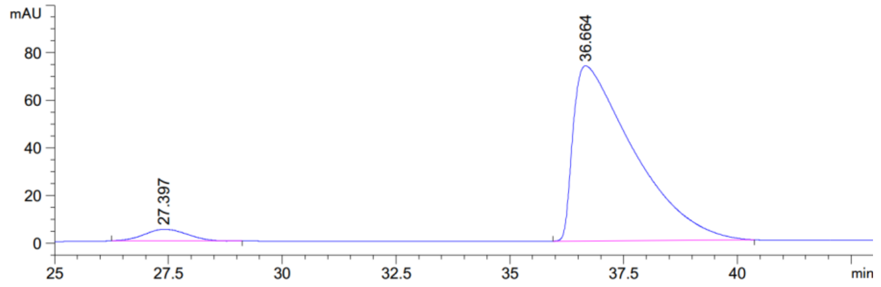

Peak #	RetTime [min]	Type	Width [min]	Area [mAU*s]	Height [mAU]	Area %
1	27.397	BB	0.7855	322.81082	4.91976	4.5137
2	36.664	BB	1.2396	6828.97754	73.65752	95.4863

Supplementary Figure 28. HPLC Chromatograph of chiral **3an**

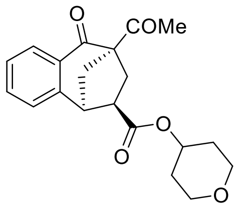

tetrahydro-2H-pyran-4-yl (5S,6R,8R)-8-acetyl-9-oxo-6,7,8,9-tetrahydro-5H-5,8-methanobenzo[7]annulene-6-carboxylate (3ao)

17.7 mg, 52% yield, >20:1 dr, 91% ee; R_f = 0.3 (PE/EA = 4/1); White solid, m.p. 38 – 40 ºC; $[\alpha]_D^{20}$ = +69 (*c* = 0.1, EA).

^{1}H NMR (400 MHz, $CDCl_3$) δ 8.05 (dd, *J* = 7.9, 1.2 Hz, 1H), 7.49 (td, *J* = 7.4, 1.5 Hz, 1H), 7.39 (td, *J* = 7.5, 1.3 Hz, 1H), 7.22 (d, *J* = 7.2 Hz, 1H), 4.68 (tt, *J* = 8.8, 4.2 Hz, 1H), 3.84 (ddd, *J* = 18.0, 9.4, 4.6 Hz, 2H), 3.69 (dt, *J* = 11.8, 4.6 Hz, 1H), 3.51 – 3.43 (m, 2H), 3.42 – 3.33 (m, 1H), 2.76 (dd, *J* = 14.8, 10.6 Hz, 1H), 2.49 (d, *J* = 12.1 Hz, 1H), 2.35 (s, 3H), 2.30 – 2.20 (m, 2H), 1.76 – 1.71 (m, 1H), 1.61 – 1.52 (m, 2H), 1.37 – 1.28 (m, 1H).

^{13}C NMR (101 MHz, $CDCl_3$) δ 205.9, 197.3, 170.8, 144.6, 133.7, 130.4, 128.3, 128.2, 127.9, 69.8, 68.8, 65.3, 65.3, 48.7, 45.8, 42.5, 31.8, 31.6, 28.6, 28.6.

HRMS (ESI) calcd. for $[C_{20}H_{22}O_5+H]^+$ 343.1540, found 343.1540.

HPLC: Daicel Chiralcel AD-H, *n*-hexane/isopropanol 98/2, flow rate = 1.0 mL/min, uv-vis λ = 254 nm, t_{R1} = 57.3 min (major), t_{R2} = 62.3 min (minor).

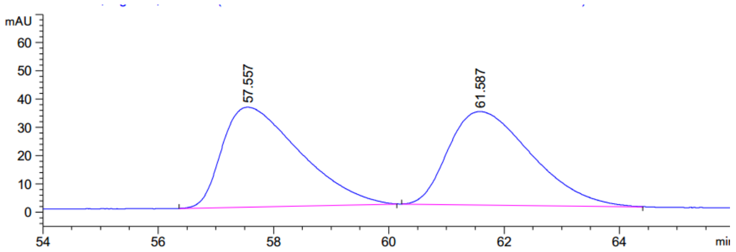

Peak #	RetTime [min]	Type	Width [min]	Area [mAU*s]	Height [mAU]	Area %
1	57.557	BB	1.2970	3306.64673	35.35984	49.9662
2	61.587	BB	1.3523	3311.12085	33.06433	50.0338

Supplementary Figure 29. HPLC Chromatograph of racemic **3ao**

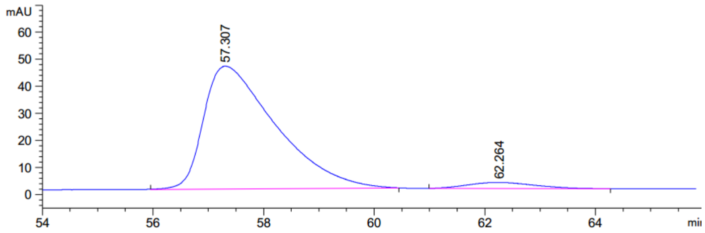

Peak #	RetTime [min]	Type	Width [min]	Area [mAU*s]	Height [mAU]	Area %
1	57.307	BB	1.2526	4121.55957	45.42485	95.7055
2	62.264	BB	0.9738	184.94498	2.24329	4.2945

Supplementary Figure 30. HPLC Chromatograph of chiral **3ao**

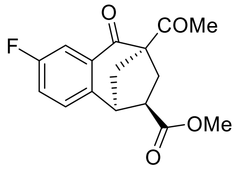

methyl (5S,6R,8R)-8-acetyl-2-fluoro-9-oxo-6,7,8,9-tetrahydro-5H-5,8-methanobenzo[7]annulene-6-carboxylate (3ba)

21.1 mg, 73% yield, >20:1 dr, 95% ee; R_f = 0.4 (PE/EA = 4/1); Yellow solid, m.p. 86 – 88 ºC; $[\alpha]_D^{20}$ = +62 (c = 0.1, EA).

^{1}H NMR (300 MHz, $CDCl_3$) δ 7.69 (d, J = 8.6 Hz, 1H), 7.22 (d, J = 1.6 Hz, 1H), 7.19 (d, J = 1.9 Hz, 1H), 3.80 (t, J = 5.0 Hz, 1H), 3.52 – 3.44 (m, 4H), 2.73 (dd, J = 14.9, 10.7 Hz, 1H), 2.45 (d, J = 12.0 Hz, 1H), 2.35 (d, J = 0.5 Hz, 3H), 2.29 – 2.19 (m, 2H).

^{13}C NMR (75 MHz, $CDCl_3$) δ 205.6, 196.2, 171.8, 162.3 (d, J = 248.7 Hz), 140.5 (d, J = 3.1 Hz), 132.3 (d, J = 6.6 Hz), 129.8 (d, J = 7.3 Hz), 121.0 (d, J = 22.2 Hz), 114.0 (d, J = 22.4 Hz), 68.3, 51.8, 48.3, 45.0, 42.4, 28.6, 28.5.

^{19}F NMR (282 MHz, $CDCl_3$) δ -112.46.

HRMS (ESI) calcd. for $[C_{16}H_{15}FO_4+Na]^+$ 313.0847, found 313.0852.

HPLC: Daicel Chiralcel AD-H, *n*-hexane/isopropanol 95/5, flow rate = 1.0 mL/min, uv-vis λ = 254 nm, t_{R1} = 16.9 min (minor), t_{R2} = 25.1 min (major).

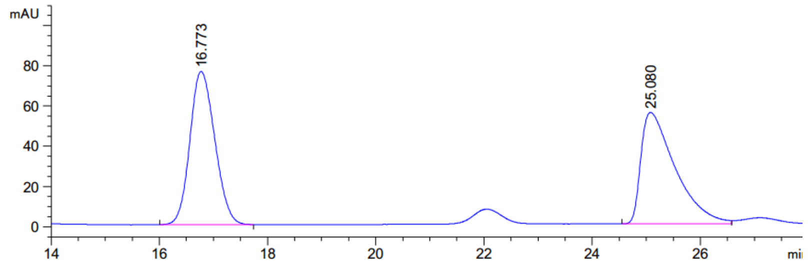

Peak #	RetTime [min]	Type	Width [min]	Area [mAU*s]	Height [mAU]	Area %
1	16.773	BB	0.4995	2438.02563	76.16235	49.9558
2	25.080	BB	0.6406	2442.34351	55.37399	50.0442

Supplementary Figure 31. HPLC Chromatograph of racemic **3ba**

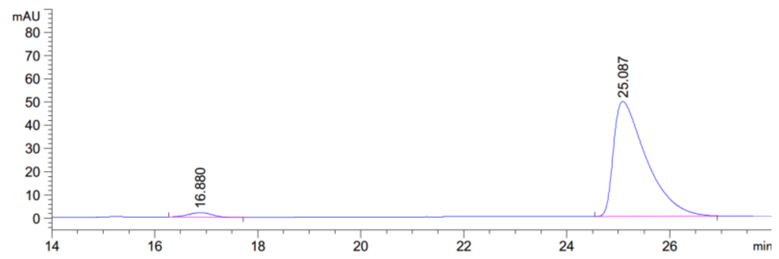

Peak #	RetTime [min]	Type	Width [min]	Area [mAU*s]	Height [mAU]	Area %
1	16.880	BB	0.3739	60.88161	1.92039	2.7056
2	25.087	BB	0.6462	2189.28711	49.48370	97.2944

Supplementary Figure 32. HPLC Chromatograph of chiral **3ba**

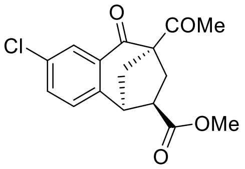

methyl (5S,6R,8R)-8-acetyl-2-chloro-9-oxo-6,7,8,9-tetrahydro-5H-5,8-methanobenzo[7]annulene-6-carboxylate (3ca)

20.5 mg, 67% yield, >20:1 dr, 92% ee; R_f = 0.4 (PE/EA = 4/1); Light yellow solid, m.p. 110 – 112 °C; $[\alpha]_D^{20}$ = +96 (c = 0.1, EA).

^{1}H NMR (300 MHz, $CDCl_3$) δ 7.99 (d, J = 2.4 Hz, 1H), 7.46 (dd, J = 8.0, 2.4 Hz, 1H), 7.17 (d, J = 8.2 Hz, 1H), 3.78 (t, J = 5.0 Hz, 1H), 3.59 – 3.45 (m, 4H), 2.74 (dd, J = 14.8, 10.7 Hz, 1H), 2.44 (d, J = 12.0 Hz, 1H), 2.34 (s, 3H), 2.29 – 2.19 (m, 2H).

^{13}C NMR (75 MHz, $CDCl_3$) δ 205.5, 196.1, 171.7, 143.0, 134.4, 133.7, 131.7, 129.5, 127.7, 68.4, 51.9, 48.3, 45.1, 42.1, 28.6, 28.5.

HRMS (ESI) calcd. for $[C_{16}H_{15}ClO_4+Na]^+$ 329.0551, found 329.0557.

HPLC: Daicel Chiralcel AS-H, *n*-hexane/isopropanol 95/5, flow rate = 1.0 mL/min, uv-vis λ = 254 nm, t_{R1} = 27.9 min (major), t_{R2} = 31.2 min (minor).

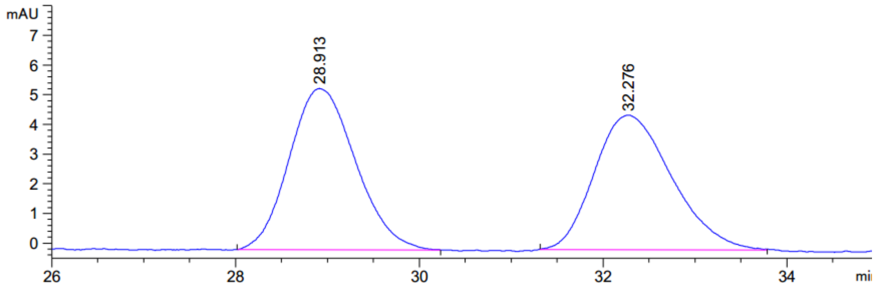

Peak #	RetTime [min]	Type	Width [min]	Area [mAU*s]	Height [mAU]	Area %
1	28.913	BB	0.6369	273.50287	5.43715	50.8230
2	32.276	BB	0.7014	264.64539	4.53118	49.1770

Supplementary Figure 33. HPLC Chromatograph of racemic **3ca**

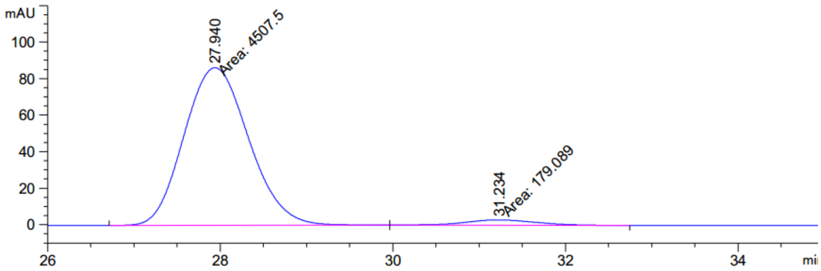

Peak #	RetTime [min]	Type	Width [min]	Area [mAU*s]	Height [mAU]	Area %
1	27.940	MM	0.8696	4507.50439	86.38972	96.1787
2	31.234	MM	0.9960	179.08876	2.99695	3.8213

Supplementary Figure 34. HPLC Chromatograph of chiral **3ca**

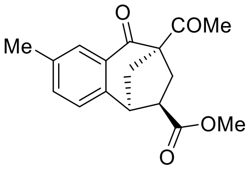

methyl (5S,6R,8R)-8-acetyl-2-methyl-9-oxo-6,7,8,9-tetrahydro-5H-5,8-methanobenzo[7]annulene-6-carboxylate (3da)

23.5 mg, 82% yield, >20:1 dr, 88% ee; R_f = 0.4 (PE/EA = 4/1); White solid, m.p. 38 – 40 ºC; $[\alpha]_D^{20}$ = +95 (c = 0.1, EA).

^{1}H NMR (300 MHz, $CDCl_3$) δ 7.84 (s, 1H), 7.31 (dd, J = 7.7, 1.3 Hz, 1H), 7.10 (d, J = 7.7 Hz, 1H), 3.77 (t, J = 4.9 Hz, 1H), 3.50 (s, 4H), 2.73 (dd, J = 14.8, 10.6 Hz, 1H), 2.46 (d, J = 11.8 Hz, 1H), 2.38 (s, 3H), 2.34 (s, 3H), 2.30 – 2.17 (m, 2H).

^{13}C NMR (75 MHz, $CDCl_3$) δ 206.0, 197.5, 172.0, 141.8, 137.9, 134.7, 130.1, 128.0, 127.8, 68.7, 64.4, 51.6, 48.4, 45.4, 42.5, 28.5, 21.0.

HRMS (ESI) calcd. for $[C_{17}H_{18}O_4+H]^+$ 287.1278, found 287.1277.

HPLC: Daicel Chiralcel AD-H, *n*-hexane/isopropanol 95/5, flow rate = 1.0 mL/min, uv-vis λ = 254 nm, t_{R1} = 18.3 min (minor), t_{R2} = 22.3 min (major).

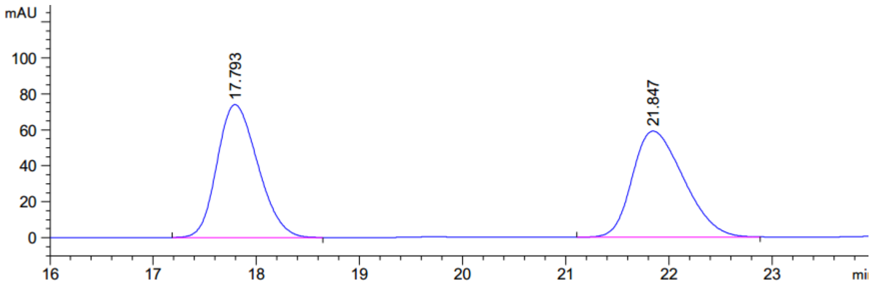

Peak #	RetTime [min]	Type	Width [min]	Area [mAU*s]	Height [mAU]	Area %
1	17.793	BB	0.4305	2030.88916	73.92899	49.8999
2	21.847	BB	0.5375	2039.03430	58.96180	50.1001

Supplementary Figure 35. HPLC Chromatograph of racemic **3da**

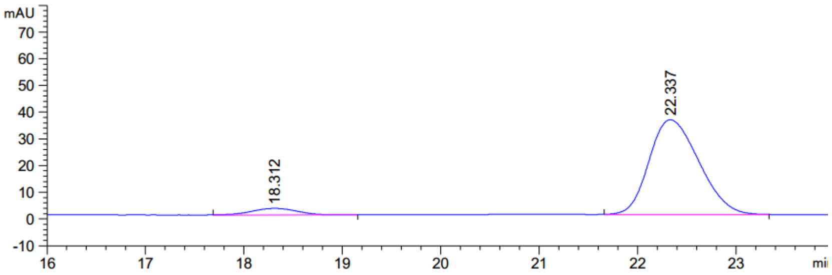

Peak #	RetTime [min]	Type	Width [min]	Area [mAU*s]	Height [mAU]	Area %
1	18.312	BB	0.4022	78.30002	2.44123	5.9024
2	22.337	BB	0.5562	1248.27734	35.50083	94.0976

Supplementary Figure 36. HPLC Chromatograph of chiral **3da**

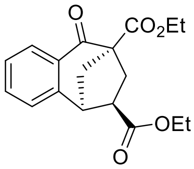

diethyl (5S,6R,8S)-9-oxo-5,6,7,9-tetrahydro-8H-5,8-methanobenzo[7]annulene-6,8-dicarboxylate (3eb)

21.5 mg, 68% yield, 12:1 dr, 95% ee; R_f = 0.4 (PE/EA = 4/1); Colorless oil; $[\alpha]_D^{20}$ = +105 (c = 0.1, EA).

^{1}H NMR (300 MHz, $CDCl_3$) δ 8.04 (d, J = 7.7 Hz, 1H), 7.47 (t, J = 7.2 Hz, 1H), 7.36 (t, J = 7.6 Hz, 1H), 7.20 (d, J = 7.4 Hz, 1H), 4.29 (q, J = 7.1 Hz, 2H), 3.92 – 3.85 (m, 2H), 3.76 (s, 1H), 3.49 (dd, J = 6.5, 3.8 Hz, 1H), 2.75 (s, 1H), 2.56 (d, J = 12.1 Hz, 1H), 2.41 – 2.26 (m, 2H), 1.32 (t, J = 7.2 Hz, 3H), 1.08 (t, J = 7.2 Hz, 3H).

^{13}C NMR (75 MHz, $CDCl_3$) δ 195.6, 171.4, 171.3, 144.5, 133.6, 130.0, 128.1, 128.0, 63.6, 61.4, 60.7, 48.6, 45.6, 42.8, 29.4, 14.2, 14.1.

HRMS (ESI) calcd. for $[C_{18}H_{20}O_5+H]^+$ 317.1384, found 317.1385.

HPLC: Daicel Chiralcel OD-H, n-hexane/isopropanol 80/20, flow rate = 0.5 mL/min, uv-vis λ = 254 nm, t_{R1} = 14.0 min (minor), t_{R2} = 19.4 min (major).

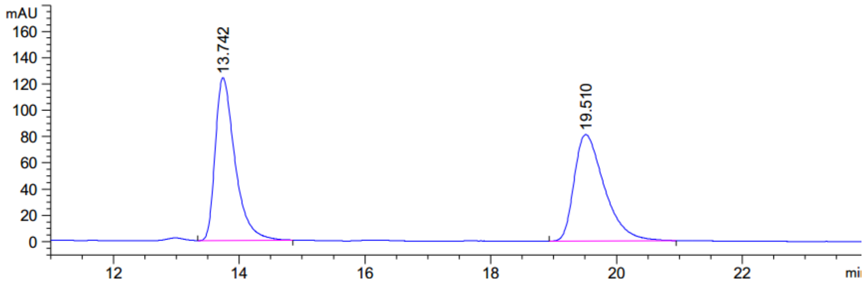

Peak #	RetTime [min]	Type	Width [min]	Area [mAU*s]	Height [mAU]	Area %
1	13.742	VB	0.3302	2700.92847	123.95464	49.9975
2	19.510	BB	0.5088	2701.19800	81.04819	50.0025

Supplementary Figure 37. HPLC Chromatograph of racemic **3eb**

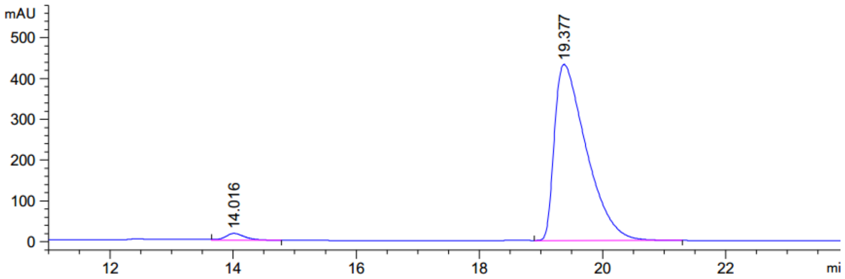

Peak #	RetTime [min]	Type	Width [min]	Area [mAU*s]	Height [mAU]	Area %
1	14.016	VB	0.3390	392.83438	17.16616	2.4674
2	19.377	VB	0.5413	1.55280e4	432.05859	97.5326

Supplementary Figure 38. HPLC Chromatograph of chiral **3eb**

General Procedure for the Palladium-Catalyzed Asymmetric Tandem Heck/Carbonylation Desymmetrization of Cyclopentenes with Phenols Exemplified by the Synthesis of 5aa

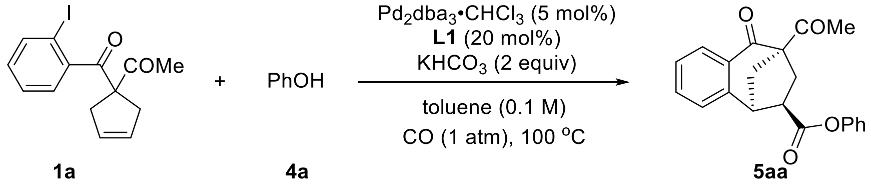

A sealed tube was charged with the substrate **1a** (34 mg, 0.1 mmol, 1 equiv), **4a** (23.5 mg, 0.25 mmol, 2.5 equiv), Pd_2dba_3•$CHCl_3$ (5.2 mg, 5 mol%), **L1** (13.4 mg, 20 mol%), and $KHCO_3$ (20 mg, 0.2 mmol, 2 equiv). The vial is thoroughly flushed with CO, and toluene (1 mL) was added under CO atmosphere. Then the reaction mixture was stirred at 100 °C for 36h. After the reaction vessel was cooled to room temperature, the solution was concentrated in *vacuo* and purified by careful chromatography on silica gel (200–300 mesh) (PE/EA = 4/1) to afford the desired product **5aa**.

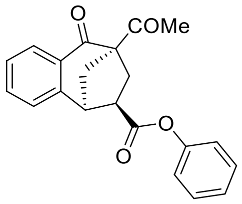

phenyl (5S,6R,8R)-8-acetyl-9-oxo-6,7,8,9-tetrahydro-5H-5,8-methanobenzo[7]annulene-6-carboxylate (5aa)

23.8 mg, 71% yield, >20:1 dr, 94% ee; R_f = 0.3 (PE/EA = 4/1); Colorless oil; $[\alpha]_D^{20}$ = +92 (c = 0.1, EA).

^{1}H NMR (400 MHz, $CDCl_3$) δ 8.09 (d, J = 7.8 Hz, 1H), 7.56 (t, J = 7.5 Hz, 1H), 7.45 (t, J = 7.6 Hz, 1H), 7.38 (d, J = 7.5 Hz, 1H), 7.29 (t, J = 7.9 Hz, 2H), 7.17 (td, J = 7.3, 1.3 Hz, 1H), 6.74 (dd, J = 7.2, 1.5 Hz, 2H), 3.97 (t, J = 4.8 Hz, 1H), 3.76 – 3.70 (m, 1H), 2.85 (dd, J = 14.9, 10.8 Hz, 1H), 2.56 (d, J = 11.6 Hz, 1H), 2.42 – 2.37 (m, 4H), 2.29 (dd, J = 12.0, 4.1 Hz, 1H).

^{13}C NMR (101 MHz, $CDCl_3$) δ 205.9, 197.2, 170.1, 150.3, 144.4, 134.0, 130.5, 129.4, 128.5, 128.5, 128.1, 125.9, 121.3, 68.7, 48.5, 45.8, 42.6, 28.7, 28.6.

HRMS (ESI) calcd. for $[C_{21}H_{18}O_4+H]^+$ 335.1278, found 335.1279.

HPLC: Daicel Chiralcel OD-H, n-hexane/isopropanol 80/20, flow rate = 1.0 mL/min, uv-vis λ = 254 nm, t_{R1} = 12.1 min (minor), t_{R2} = 15.1 min (major).

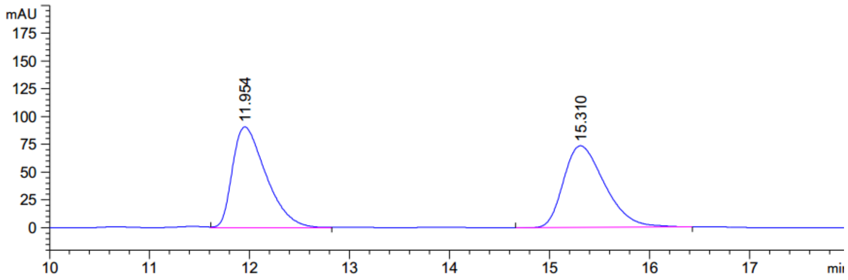

Peak #	RetTime [min]	Type	Width [min]	Area [mAU*s]	Height [mAU]	Area %
1	11.954	VB	0.3617	2130.77563	90.80982	50.1848
2	15.310	VB	0.4452	2115.08594	73.62263	49.8152

Supplementary Figure 39. HPLC Chromatograph of racemic **5aa**

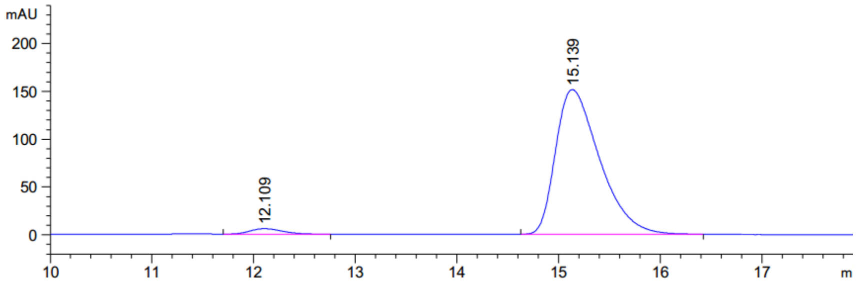

Peak #	RetTime [min]	Type	Width [min]	Area [mAU*s]	Height [mAU]	Area %
1	12.109	VB	0.3410	133.69592	5.84175	2.8501
2	15.139	BB	0.4594	4557.15918	151.28465	97.1499

Supplementary Figure 40. HPLC Chromatograph of chiral **5aa**

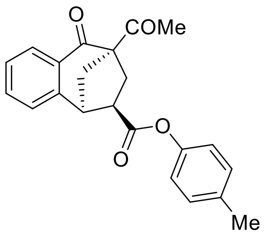

***p*-tolyl (5S,6R,8R)-8-acetyl-9-oxo-6,7,8,9-tetrahydro-5H-5,8-methanobenzo[7]annulene-6-carboxylate (5ab)**

26.8 mg, 77% yield, >20:1 dr, 95% ee; R_f = 0.4 (PE/EA = 4/1); Yellow solid, m.p. 86 – 88 ºC; $[\alpha]^{20}_{D}$ = +53 (*c* = 0.1, EA).

^{1}H NMR (400 MHz, $CDCl_3$) δ 8.08 (dd, *J* = 7.8, 1.6 Hz, 1H), 7.55 (td, *J* = 7.5, 1.5 Hz, 1H), 7.44 (td, *J* = 7.6, 1.4 Hz, 1H), 7.37 (d, *J* = 7.6 Hz, 1H), 7.07 (d, *J* = 8.8 Hz, 2H), 6.62 (d, *J* = 8.6 Hz, 2H), 3.96 (t, *J* = 4.9 Hz, 1H), 3.74 – 3.68 (m, 1H), 2.83 (dd, *J* = 14.7, 10.9 Hz, 1H), 2.55 (d, *J* = 11.8 Hz, 1H), 2.41 – 2.37 (m, 4H), 2.29 – 2.27 (m, 4H).

^{13}C NMR (101 MHz, $CDCl_3$) δ 206.0, 197.3, 170.3, 148.1, 144.4, 135.6, 134.0, 130.4, 129.9, 128.5, 128.4, 128.1, 120.9, 68.7, 48.5, 45.8, 42.6, 28.7, 20.9.

HRMS (ESI) calcd. for $[C_{22}H_{20}O_4+H]^+$ 349.1434, found 349.1432.

HPLC: Daicel Chiralcel OD-H, *n*-hexane/isopropanol 80/20, flow rate = 1.0 mL/min, uv-vis λ = 254 nm, t_{R1} = 14.0 min (minor), t_{R2} = 17.9 min (major).

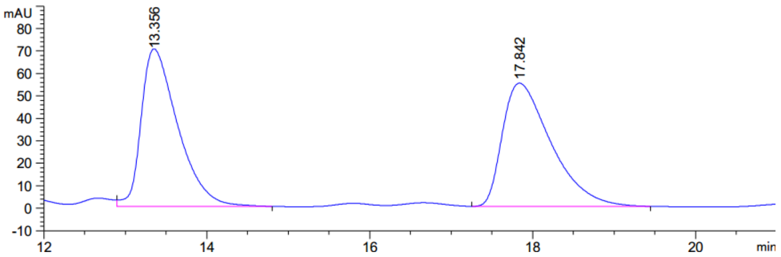

Peak #	RetTime [min]	Type	Width [min]	Area [mAU*s]	Height [mAU]	Area %
1	13.356	VB	0.4717	2198.79834	70.15205	48.9744
2	17.842	VB	0.6200	2290.88916	54.99202	51.0256

Supplementary Figure 41. HPLC Chromatograph of racemic **5ab**

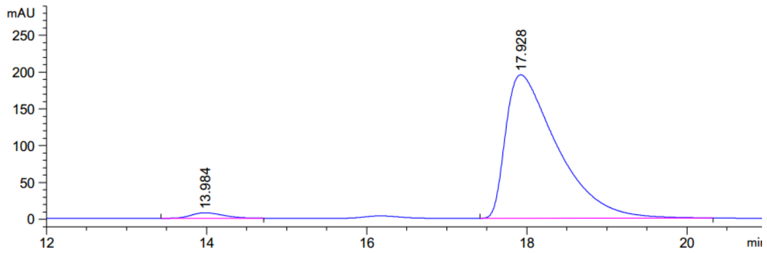

Peak #	RetTime [min]	Type	Width [min]	Area [mAU*s]	Height [mAU]	Area %
1	13.984	BB	0.4201	207.97981	7.48727	2.3225
2	17.928	BB	0.6635	8746.97754	194.93213	97.6775

Supplementary Figure 42. HPLC Chromatograph of chiral **5ab**

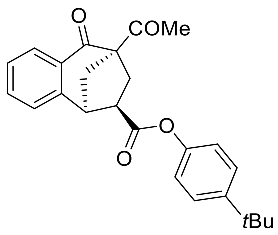

4-(tert-butyl)phenyl (5S,6R,8R)-8-acetyl-9-oxo-6,7,8,9-tetrahydro-5H-5,8-methanobenzo[7]annulene-6-carboxylate ((5S,6R,8R)-5ac)

29.5 mg, 76% yield, >20:1 dr, 92% ee; R_f = 0.5 (PE/EA = 4/1); White solid, m.p. 114 – 116 ºC; $[\alpha]_D^{20}$ = +173 (*c* = 0.1, EA).

^{1}H NMR (300 MHz, $CDCl_3$) δ 8.08 (dd, *J* = 7.7, 1.4 Hz, 1H), 7.57 (td, *J* = 7.4, 1.5 Hz, 1H), 7.44 (td, *J* = 7.6, 1.3 Hz, 1H), 7.38 (dd, *J* = 7.5, 1.2 Hz, 1H), 7.29 (dt, *J* = 9.7, 2.9, 2.1 Hz, 2H), 6.66 (dt, *J* = 9.7, 2.9, 2.1 Hz, 2H), 3.97 (t, *J* = 5.1, 4.6 Hz, 1H), 3.72 (ddd, *J* = 10.7, 6.7, 5.8 Hz, 1H), 2.84 (dd, *J* = 14.9, 10.7 Hz, 1H), 2.55 (d, *J* = 11.9 Hz, 1H), 2.43 – 2.35 (m, 4H), 2.29 (dd, *J* = 11.9, 4.2 Hz, 1H), 1.27 (s, 9H).

^{13}C NMR (75 MHz, $CDCl_3$) δ 205.9, 197.3, 170.2, 148.8, 148.0, 144.4, 134.0, 130.5, 128.5, 128.4, 128.1, 126.3, 120.6, 68.7, 48.5, 45.8, 42.6, 34.5, 31.4, 28.7, 28.6.

HRMS (ESI) calcd. for $[C_{25}H_{26}O_4+H]^+$ 391.1904, found 391.1902.

HPLC: Daicel Chiralcel AD-H, *n*-hexane/isopropanol 90/10, flow rate = 1.0 mL/min, uv-vis λ = 254 nm, t_{R1} = 10.5 min (minor), t_{R2} = 12.4 min (major).

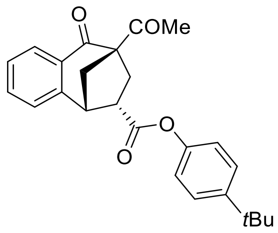

4-(tert-butyl)phenyl (5R,6S,8S)-8-acetyl-9-oxo-6,7,8,9-tetrahydro-5H-5,8-methanobenzo[7]annulene-6-carboxylate (5R,6S,8S-5ac')

27.5 mg, 70% yield, >20:1 dr, 97% ee; R_f = 0.5 (PE/EA = 4/1); White solid, m.p. 102 – 104 ºC; $[\alpha]_D^{20}$ = -181 (*c* = 0.1, EA).

HPLC: Daicel Chiralcel AD-H, *n*-hexane/isopropanol 90/10, flow rate = 1.0 mL/min, uv-vis λ = 254 nm, t_{R1} = 10.2 min (major), t_{R2} = 12.3 min (minor).

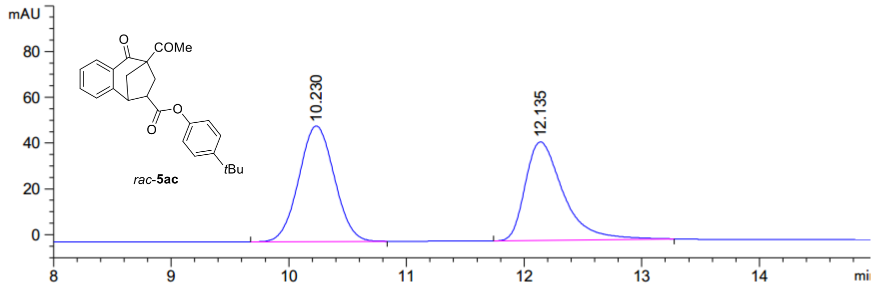

Peak #	RetTime [min]	Type	Width [min]	Area [mAU*s]	Height [mAU]	Area %
1	10.230	BB	0.3285	1058.77039	50.51239	52.1609
2	12.135	BB	0.3433	971.04437	43.03283	47.8391

Supplementary Figure 43. HPLC Chromatograph of racemic **5ac**

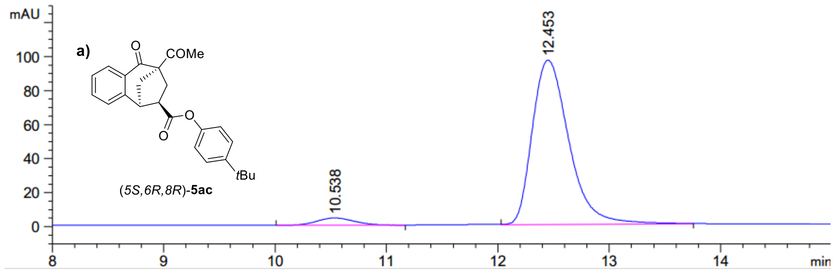

Peak #	RetTime [min]	Type	Width [min]	Area [mAU*s]	Height [mAU]	Area %
1	10.538	BB	0.3463	97.96191	4.22864	4.2461
2	12.453	VB	0.3588	2209.12646	96.59844	95.7539

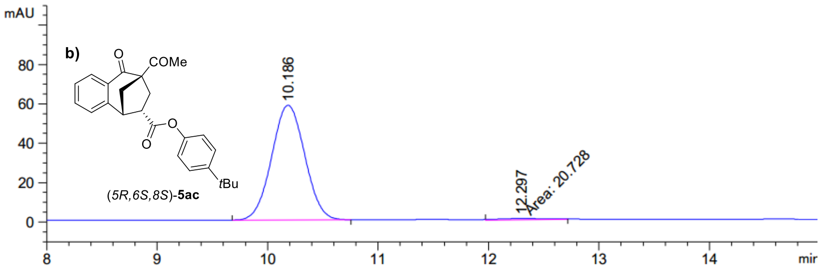

Peak #	RetTime [min]	Type	Width [min]	Area [mAU*s]	Height [mAU]	Area %
1	10.186	BB	0.3274	1216.68494	58.31234	98.3249
2	12.297	MM	0.5317	20.72796	6.49758e-1	1.6751

Supplementary Figure 44. HPLC Chromatograph of chiral **5ac. a (5*S*,6*R*,8*R*)-5ac**

b (5*R*,6*S*,8*S*)-5ac

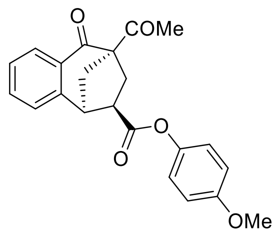

4-methoxyphenyl (5S,6R,8R)-8-acetyl-9-oxo-6,7,8,9-tetrahydro-5H-5,8-methanobenzo[7]annulene-6-carboxylate(5ad)

34.6 mg, 95% yield, >20:1 dr, 93% ee; R_f = 0.1 (PE/EA = 4/1); White solid, m.p. 158 – 160 °C; $[\alpha]^{20}_{D}$ = +208 (*c* = 0.1, EA).

^{1}H NMR (400 MHz, $CDCl_3$) δ 8.08 (dd, *J* = 7.8, 1.4 Hz, 1H), 7.56 (td, *J* = 7.5, 1.5 Hz, 1H), 7.44 (td, *J* = 7.6, 1.3 Hz, 1H), 7.37 (d, *J* = 7.5 Hz, 1H), 6.81 – 6.77 (m, 2H), 6.67 – 6.63 (m, 2H), 3.96 (t, *J* = 5.3, 4.6 Hz, 1H), 3.75 (s, 3H), 3.71 (ddd, *J* = 10.7, 6.6, 5.8 Hz, 1H), 2.83 (dd, *J* = 14.9, 10.7 Hz, 1H), 2.55 (d, *J* = 11.8 Hz, 1H), 2.41 – 2.36 (m, 4H), 2.29 (dd, *J* = 11.9, 4.2 Hz, 1H).

^{13}C NMR (101 MHz, $CDCl_3$) δ 205.9, 197.2, 170.4, 157.3, 144.5, 143.8, 133.9, 130.5, 128.5, 128.4, 128.1, 122.0, 114.4, 68.7, 55.6, 48.5, 45.8, 42.6, 28.7, 28.6.

HRMS (ESI) calcd. for $[C_{22}H_{20}O_5+H]^+$ 365.1384, found 365.1382.

HPLC: Daicel Chiralcel OD-H, *n*-hexane/isopropanol 80/20, flow rate = 1.0 mL/min, uv-vis λ = 254 nm, t_{R1} = 16.1 min (minor), t_{R2} = 21.2 min (major).

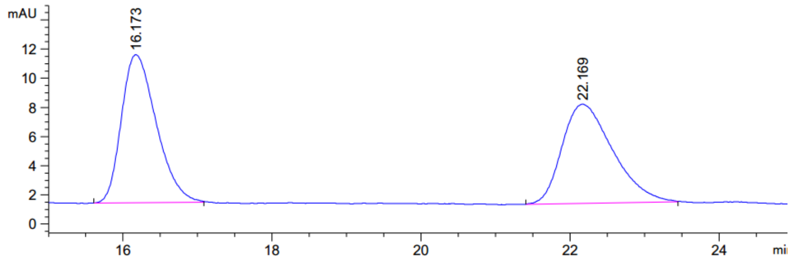

Peak #	RetTime [min]	Type	Width [min]	Area [mAU*s]	Height [mAU]	Area %
1	16.173	BB	0.4866	331.56555	10.16415	50.7758
2	22.169	BB	0.6350	321.43399	6.80576	49.2242

Supplementary Figure 45. HPLC Chromatograph of racemic **5ad**

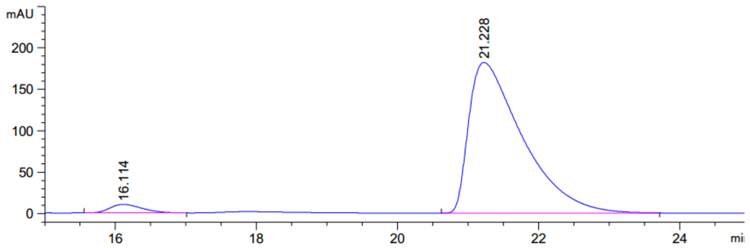

Peak #	RetTime [min]	Type	Width [min]	Area [mAU*s]	Height [mAU]	Area %
1	16.114	BB	0.4681	329.91016	10.28870	3.3162
2	21.228	BB	0.7717	9618.51172	181.67981	96.6838

Supplementary Figure 46. HPLC Chromatograph of chiral **5ad**

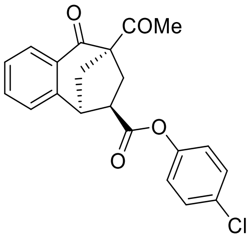

4-chlorophenyl (5S,6R,8R)-8-acetyl-9-oxo-6,7,8,9-tetrahydro-5H-5,8-methanobenzo[7]annulene-6-carboxylate (5ae)

26.2 mg, 71% yield, >20:1 dr, 91% ee; R_f = 0.3 (PE/EA = 4/1); Brown solid, m.p. 146 – 148 ºC; $[\alpha]_D^{20}$ = +92 (c = 0.1, EA).

^{1}H NMR (400 MHz, $CDCl_3$) δ 8.09 (dd, J = 7.7, 1.5 Hz, 1H), 7.55 (td, J = 7.5, 1.5 Hz, 1H), 7.45 (td, J = 7.6, 1.3 Hz, 1H), 7.34 (dd, J = 7.5, 1.2 Hz, 1H), 7.25 (dt, J = 9.0, 2.2 Hz, 2H), 6.70 – 6.65 (m, 2H), 3.96 (t, J = 5.4, 4.6 Hz, 1H), 3.72 (ddd, J = 10.7, 6.6, 5.8 Hz, 1H), 2.85 (dd, J = 14.9, 10.7 Hz, 1H), 2.56 (d, J = 11.9 Hz, 1H), 2.41 – 2.33 (m, 4H), 2.29 (dd, J = 11.9, 4.2 Hz, 1H).

^{13}C NMR (101 MHz, $CDCl_3$) δ 205.7, 197.1, 169.9, 148.8, 144.3, 134.0, 131.3, 130.5, 129.4, 128.5, 128.3, 128.2, 122.6, 68.7, 48.5, 45.8, 42.6, 28.6, 28.6.

HRMS (ESI) calcd. for $[C_{21}H_{17}ClO_4+H]^+$ 369.0888, found 369.0888.

HPLC: Daicel Chiralcel OD-H, n-hexane/isopropanol 90/10, flow rate = 1.0 mL/min, uv-vis λ = 254 nm, t_{R1} = 21.1 min (major), t_{R2} = 23.7 min (minor).

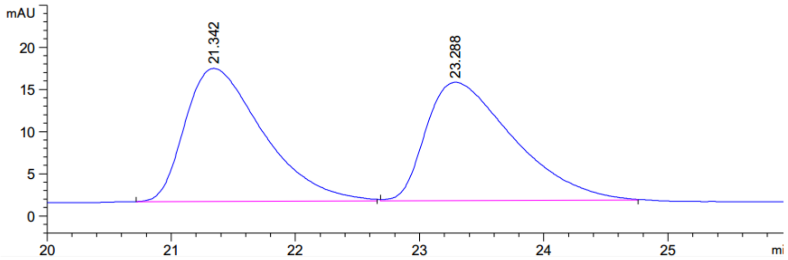

Peak #	RetTime [min]	Type	Width [min]	Area [mAU*s]	Height [mAU]	Area %
1	21.342	BB	0.6377	688.89484	15.76836	50.0688
2	23.288	BB	0.6992	687.00128	14.03169	49.9312

Supplementary Figure 47. HPLC Chromatograph of racemic **5ae**

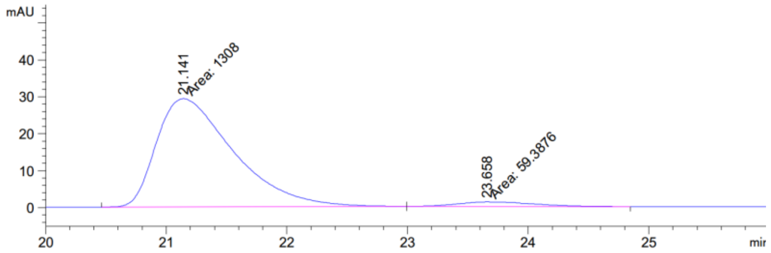

Peak #	RetTime [min]	Type	Width [min]	Area [mAU*s]	Height [mAU]	Area %
1	21.141	MM	0.7432	1308.00085	29.33399	95.6569
2	23.658	MM	0.7699	59.38757	1.28562	4.3431

Supplementary Figure 48. HPLC Chromatograph of chiral **5ae**

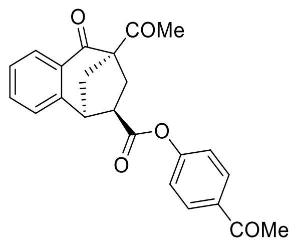

4-acetylphenyl (5S,6R,8R)-8-acetyl-9-oxo-6,7,8,9-tetrahydro-5H-5,8-methanobenzo[7]annulene-6-carboxylate (5af)

18.9 mg, 50% yield, >20:1 dr, 93% ee; R_f = 0.2 (PE/EA = 4/1); White solid, m.p. 160 – 162 ºC; $[\alpha]_D^{20}$ = +137 (c = 0.1, EA).

^{1}H NMR (400 MHz, $CDCl_3$) δ 8.10 (dd, J = 7.7, 1.5 Hz, 1H), 7.96 – 7.87 (m, 2H), 7.56 (td, J = 7.5, 1.5 Hz, 1H), 7.46 (td, J = 7.6, 1.3 Hz, 1H), 7.35 (d, J = 7.5 Hz, 1H), 6.84 (dt, J = 9.4, 2.5, 2.1 Hz, 2H), 3.99 (t, J = 5.1 Hz, 1H), 3.76 (ddd, J = 10.7, 6.5, 5.8 Hz, 1H), 2.87 (dd, J = 15.0, 10.7 Hz, 1H), 2.59 – 2.53 (m, 4H), 2.41 – 2.35 (m, 4H), 2.31 (dd, J = 11.9, 4.2 Hz, 1H).

^{13}C NMR (101 MHz, $CDCl_3$) δ 205.7, 197.0, 196.8, 169.6, 153.9, 144.2, 134.8, 134.0, 130.5, 129.9, 128.6, 128.3, 128.2, 121.4, 68.8, 48.5, 45.9, 42.6, 28.6, 28.6, 26.6.

HRMS (ESI) calcd. for $[C_{23}H_{20}O_5+H]^+$ 377.1384 found 377.1383.

HPLC: Daicel Chiralcel AD-H, n-hexane/isopropanol 80/20, flow rate = 1.0 mL/min, uv-vis λ = 254 nm, t_{R1} = 19.3 min (minor), t_{R2} = 25.2 min (major).

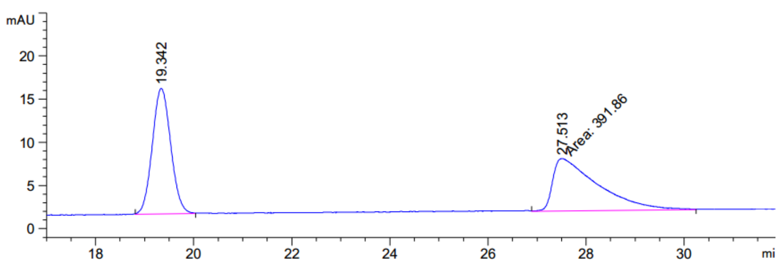

Peak #	RetTime [min]	Type	Width [min]	Area [mAU*s]	Height [mAU]	Area %
1	19.342	BB	0.4041	379.15057	14.54664	49.1758
2	27.513	MM	1.0749	391.85962	6.07599	50.8242

Supplementary Figure 49. HPLC Chromatograph of racemic **5af**

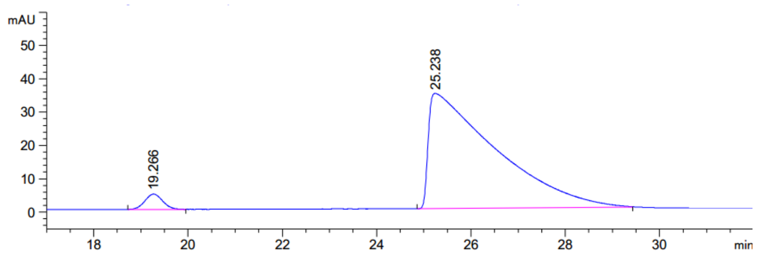

Peak #	RetTime [min]	Type	Width [min]	Area [mAU*s]	Height [mAU]	Area %
1	19.266	BB	0.4048	120.67721	4.61854	3.4027
2	25.238	BB	1.2410	3425.81055	34.54548	96.5973

Supplementary Figure 50. HPLC Chromatograph of chiral **5af**

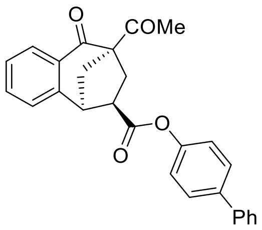

[1,1'-biphenyl]-4-yl (5S,6R,8R)-8-acetyl-9-oxo-6,7,8,9-tetrahydro-5H-5,8-methanobenzo[7]annulene-6-carboxylate (5ag)

28.2 mg, 69% yield, >20:1 dr, 94% ee; R_f = 0.5 (PE/EA = 4/1); Light yellow solid, m.p. 182 – 184 °C; $[\alpha]_D^{20}$ = +55 (c = 0.1, EA).

^{1}H NMR (300 MHz, $CDCl_3$) δ 8.10 (d, J = 7.5 Hz, 1H), 7.57 (td, J = 7.4, 1.5 Hz, 1H), 7.52 – 7.47 (m, 4H), 7.45 – 7.38 (m, 4H), 7.33 (t, J = 7.2 Hz, 1H), 6.81 (d, J = 8.6 Hz, 2H), 3.98 (t, J = 4.9 Hz, 1H), 3.75 (dt, J = 10.6, 6.2 Hz, 1H), 2.86 (dd, J = 14.9, 10.7 Hz, 1H), 2.56 (d, J = 11.9 Hz, 1H), 2.44 – 2.37 (m, 4H), 2.30 (dd, J = 11.9, 4.2 Hz, 1H).

^{13}C NMR (75 MHz, $CDCl_3$) δ 205.7, 197.1, 170.0, 149.7, 144.4, 140.2, 139.0, 133.9, 130.5, 128.7, 128.4, 128.1, 128.0, 127.4, 127.0, 121.4, 68.7, 48.5, 45.8, 42.6, 28.7, 28.5.

HRMS (ESI) calcd. for $[C_{27}H_{22}O_4+H]^+$ 411.1591, found 411.1587.

HPLC: Daicel Chiralcel OD-H, n-hexane/isopropanol 80/20, flow rate = 1.0 mL/min, uv-vis λ = 254 nm, t_{R1} = 19.3 min (minor), t_{R2} = 25.9 min (major).

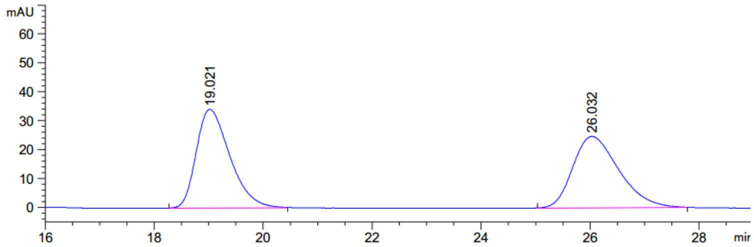

Peak #	RetTime [min]	Type	Width [min]	Area [mAU*s]	Height [mAU]	Area %
1	19.021	BB	0.6540	1463.64880	34.15186	50.1847
2	26.032	BB	0.8473	1452.87305	24.73450	49.8153

Supplementary Figure 51. HPLC Chromatograph of racemic **5ag**

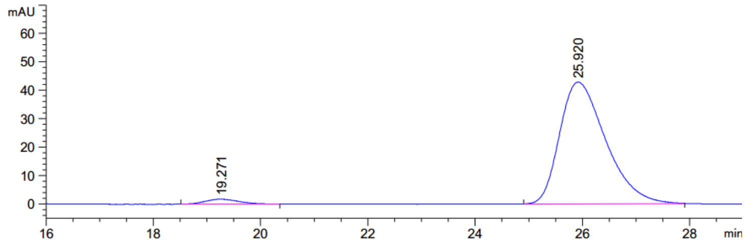

Peak #	RetTime [min]	Type	Width [min]	Area [mAU*s]	Height [mAU]	Area %
1	19.271	BB	0.5160	74.63992	1.71415	2.8362
2	25.920	BB	0.8959	2557.05713	42.86594	97.1638

Supplementary Figure 52. HPLC Chromatograph of chiral **5ag**

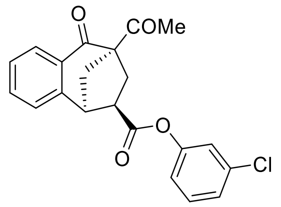

3-chlorophenyl (5S,6R,8R)-8-acetyl-9-oxo-6,7,8,9-tetrahydro-5H-5,8-methanobenzo[7]annulene-6-carboxylate (5ah)

20.0 mg, 54% yield, >20:1 dr, 95% ee; R_f = 0.2 (PE/EA = 4/1); Light yellow oil; $[\alpha]_D^{20}$ = +21 (*c* = 0.1, EA).

^{1}H NMR (300 MHz, $CDCl_3$) δ 8.10 (dd, *J* = 7.7, 1.5 Hz, 1H), 7.57 (td, *J* = 7.4, 1.5 Hz, 1H), 7.47 (td, *J* = 7.5, 1.3 Hz, 1H), 7.35 (dd, *J* = 7.5, 1.3 Hz, 1H), 7.22 – 7.19 (m, 1H), 7.18 – 7.17 (m, 1H), 6.78 – 6.70 (m, 1H), 6.65 (ddd, *J* = 7.8, 2.2, 1.4 Hz, 1H), 3.96 (t, *J* = 5.3, 4.7 Hz, 1H), 3.73 (ddd, *J* = 10.7, 6.6, 5.8 Hz, 1H), 2.86 (dd, *J* = 14.9, 10.7 Hz, 1H), 2.57 (d, *J* = 12.1 Hz, 1H), 2.41 – 2.36 (m, 4H), 2.29 (dd, *J* = 11.9, 4.2 Hz, 1H).

^{13}C NMR (75 MHz, $CDCl_3$) δ 205.7, 197.1, 169.7, 150.7, 144.2, 134.6, 134.0, 130.5, 130.1, 128.6, 128.3, 128.2, 126.2, 122.0, 119.6, 68.8, 48.5, 45.9, 42.6, 28.7, 28.6.

HRMS (ESI) calcd. for $[C_{21}H_{17}ClO_4+H]^+$ 369.0888, found 369.0887.

HPLC: Daicel Chiralcel OD-H, *n*-hexane/isopropanol 90/10, flow rate = 1.0 mL/min, uv-vis λ = 254 nm, t_{R1} = 21.1 min (major), t_{R2} = 23.7 min (minor).

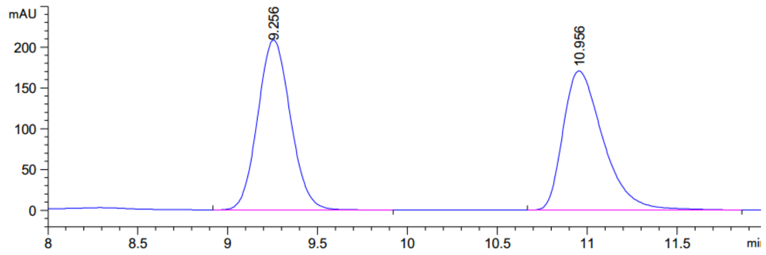

Peak #	RetTime [min]	Type	Width [min]	Area [mAU*s]	Height [mAU]	Area %
1	9.256	VB	0.1984	2650.91162	208.41206	50.1024
2	10.956	BB	0.2367	2640.07837	170.49718	49.8976

Supplementary Figure 53. HPLC Chromatograph of racemic **5ah**

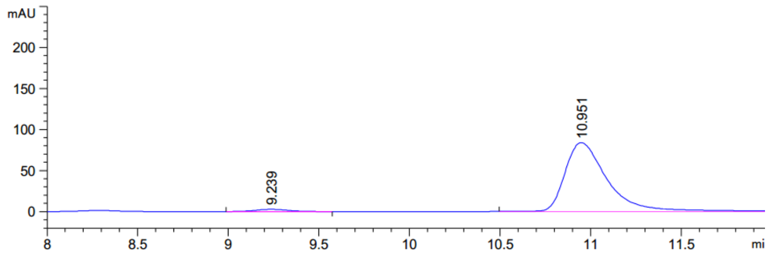

Peak #	RetTime [min]	Type	Width [min]	Area [mAU*s]	Height [mAU]	Area %
1	9.239	BB	0.1938	33.89311	2.75002	2.4389
2	10.951	BB	0.2448	1355.78137	83.80091	97.5611

Supplementary Figure 54. HPLC Chromatograph of chiral **5ah**

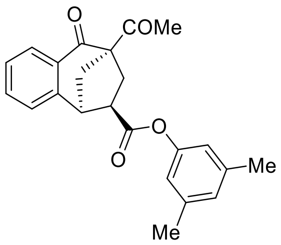

3,5-dimethylphenyl (5S,6R,8R)-8-acetyl-9-oxo-6,7,8,9-tetrahydro-5H-5,8-methanobenzo[7]annulene-6-carboxylate (5ai)

25.3 mg, 70% yield, >20:1 dr, 92% ee; R_f = 0.4 (PE/EA = 4/1); White solid, m.p. 154 – 156 ºC; $[\alpha]^{20}_{D}$ = +154 (*c* = 0.1, EA).

^{1}H NMR (300 MHz, $CDCl_3$) δ 8.08 (dd, *J* = 7.8, 1.5 Hz, 1H), 7.54 (td, *J* = 7.4, 1.5 Hz, 1H), 7.44 (td, *J* = 7.5, 1.4 Hz, 1H), 7.37 (d, *J* = 7.5 Hz, 1H), 6.79 (s, 1H), 6.33 (s, 2H), 3.94 (t, *J* = 4.9 Hz, 1H), 3.69 (dt, *J* = 10.6, 6.6 Hz, 1H), 2.83 (dd, *J* = 14.9, 10.7 Hz, 1H), 2.54 (d, *J* = 11.9 Hz, 1H), 2.42 – 2.35 (m, 4H), 2.31 – 2.23 (m, 7H).

^{13}C NMR (101 MHz, $CDCl_3$) δ 205.9, 197.2, 170.2, 150.2, 144.5, 139.2, 133.9, 130.5, 128.7, 128.4, 128.1, 127.6, 118.8, 68.7, 48.5, 45.8, 42.6, 28.7, 28.6, 21.2.

HRMS (ESI) calcd. for $[C_{23}H_{22}O_4+H]^+$ 363.1591, found 363.1590.

HPLC: Daicel Chiralcel AD-H, *n*-hexane/isopropanol 80/20, flow rate = 1.0 mL/min, uv-vis λ = 254 nm, t_{R1} = 7.1 min (minor), t_{R2} = 13.3 min (major).

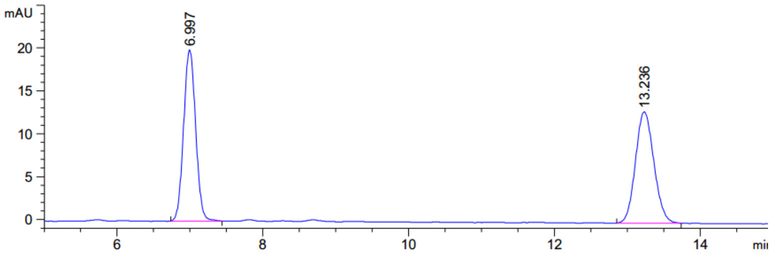

Peak #	RetTime [min]	Type	Width [min]	Area [mAU*s]	Height [mAU]	Area %
1	6.997	BB	0.1802	229.75894	19.96772	50.1646
2	13.236	BB	0.2750	228.25093	12.99232	49.8354

Supplementary Figure 55. HPLC Chromatograph of racemic **5ai**

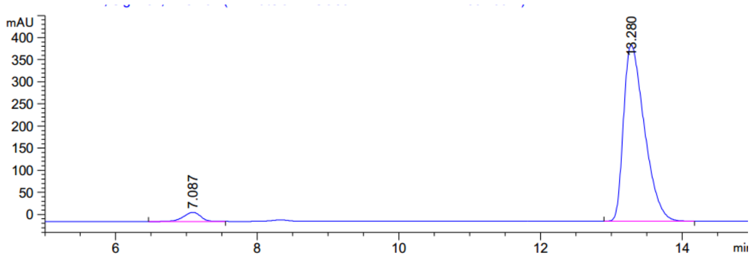

Peak #	RetTime [min]	Type	Width [min]	Area [mAU*s]	Height [mAU]	Area %
1	7.087	BB	0.2574	346.06717	20.65800	4.0719
2	13.280	BB	0.3147	8152.76758	398.53070	95.9281

Supplementary Figure 56. HPLC Chromatograph of chiral **5ai**

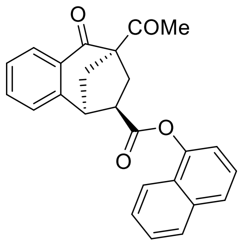

naphthalen-1-yl (5S,6R,8R)-8-acetyl-9-oxo-6,7,8,9-tetrahydro-5H-5,8-methanobenzo[7]annulene-6-carboxylate (5aj)

24.2 mg, 63% yield, >20:1 dr, 95% ee; R_f = 0.3 (PE/EA = 4/1); Brown solid, m.p. 98 – 100 °C; $[\alpha]^{20}_{D}$ = -51 (*c* = 0.1, EA).

^{1}H NMR (300 MHz, $CDCl_3$) δ 8.13 (dd, *J* = 7.6, 1.6 Hz, 1H), 7.82 (d, *J* = 8.1 Hz, 1H), 7.67 (d, *J* = 8.3 Hz, 1H), 7.58 – 7.39 (m, 6H), 7.34 (t, *J* = 8.2, 7.7 Hz, 1H), 6.80 (dd, *J* = 7.5, 1.1 Hz, 1H), 4.06 (t, *J* = 5.0 Hz, 1H), 3.91 (ddd, *J* = 10.7, 6.8, 5.7 Hz, 1H), 2.98 (dd, *J* = 15.0, 10.7 Hz, 1H), 2.59 (d, *J* = 11.9 Hz, 1H), 2.49 (ddd, *J* = 14.9, 6.9, 1.4 Hz, 1H), 2.39 (s, 3H), 2.34 (dd, *J* = 11.9, 4.2 Hz, 1H).

^{13}C NMR (75 MHz, $CDCl_3$) δ 205.8, 197.3, 170.1, 146.2, 144.5, 134.6, 134.2, 130.4, 128.9, 128.5, 128.1, 128.0, 126.5, 126.5, 126.4, 126.1, 125.2, 120.9, 117.7, 68.7, 48.7, 45.9, 42.7, 28.8, 28.6.

HRMS (ESI) calcd. for $[C_{25}H_{20}O_4+H]^+$ 385.1434, found 385.1435.

HPLC: Daicel Chiralcel AD-H, *n*-hexane/isopropanol 80/20, flow rate = 1.0 mL/min, uv-vis λ = 254 nm, t_{R1} = 11.7 min (minor), t_{R2} = 19.6 min (major).

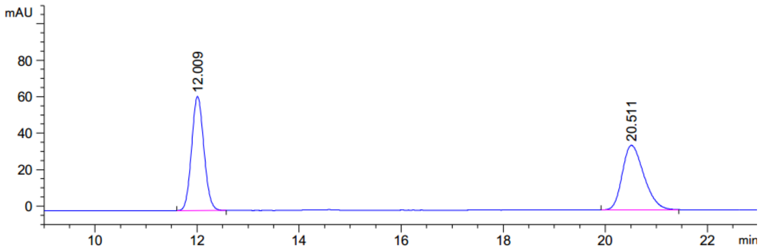

Peak #	RetTime [min]	Type	Width [min]	Area [mAU*s]	Height [mAU]	Area %
1	12.009	BB	0.2594	1047.63318	62.56199	50.1077
2	20.511	BB	0.4495	1043.13098	35.43254	49.8923

Supplementary Figure 57. HPLC Chromatograph of racemic **5aj**

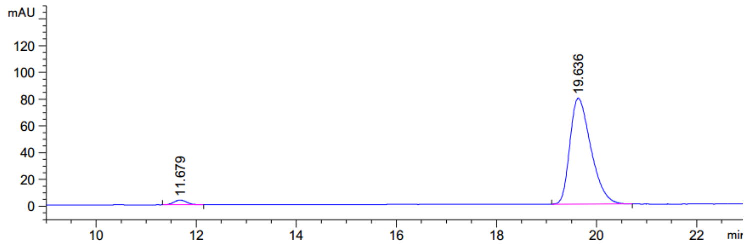

Peak #	RetTime [min]	Type	Width [min]	Area [mAU*s]	Height [mAU]	Area %
1	11.679	BB	0.2689	62.89478	3.54596	2.6469
2	19.636	BB	0.4468	2313.31201	79.18945	97.3531

Supplementary Figure 58. HPLC Chromatograph of chiral **5aj**

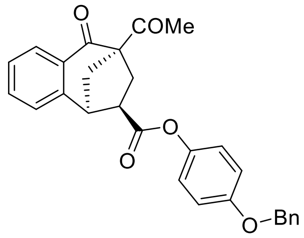

4-(benzyloxy)phenyl (5S,6R,8R)-8-acetyl-9-oxo-6,7,8,9-tetrahydro-5H-5,8-methanobenzo[7]annulene-6-carboxylate (5ak)

25.2 mg, 57% yield, >20:1 dr, 91% ee; R_f = 0.4 (PE/EA = 4/1); White solid, m.p. 150 – 152 ºC; $[\alpha]_D^{20}$ = +73 (*c* = 0.1, EA).

^{1}H NMR (300 MHz, $CDCl_3$) δ 8.08 (d, *J* = 7.0 Hz, 1H), 7.55 (td, *J* = 7.4, 1.5 Hz, 1H), 7.46 – 7.30 (m, 7H), 6.86 (d, *J* = 9.1 Hz, 2H), 6.65 (d, *J* = 9.0 Hz, 2H), 5.00 (s, 2H), 3.95 (t, *J* = 5.1, 4.7 Hz, 1H), 3.70 (dt, *J* = 10.7, 6.3 Hz, 1H), 2.83 (dd, *J* = 14.9, 10.7 Hz, 1H), 2.54 (d, *J* = 11.9 Hz, 1H), 2.42 – 2.35 (m, 4H), 2.28 (dd, *J* = 11.9, 4.2 Hz, 1H).

^{13}C NMR (75 MHz, $CDCl_3$) δ 205.7, 197.2, 170.3, 156.5, 144.4, 144.0, 136.8, 133.9, 130.5, 128.6, 128.5, 128.4, 128.1, 128.0, 127.4, 122.0, 115.4, 70.4, 68.7, 48.5, 45.8, 42.6, 28.7, 28.6.

HRMS (ESI) calcd. for $[C_{28}H_{24}O_5+H]^+$ 441.1697, found 441.1693.

HPLC: Daicel Chiralcel OD-H, *n*-hexane/isopropanol 80/20, flow rate = 1.0 mL/min, uv-vis λ = 254 nm, t_{R1} = 30.6 min (minor), t_{R2} = 42.6 min (major).

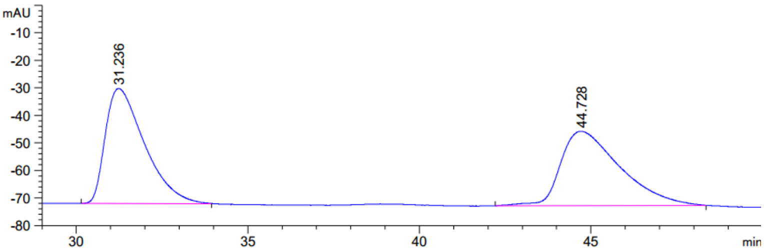

Peak #	RetTime [min]	Type	Width [min]	Area [mAU*s]	Height [mAU]	Area %
1	31.236	BB	1.1157	3321.55005	41.77168	50.4302
2	44.728	BB	1.4272	3264.88672	27.02976	49.5698

Supplementary Figure 59. HPLC Chromatograph of racemic **5ak**

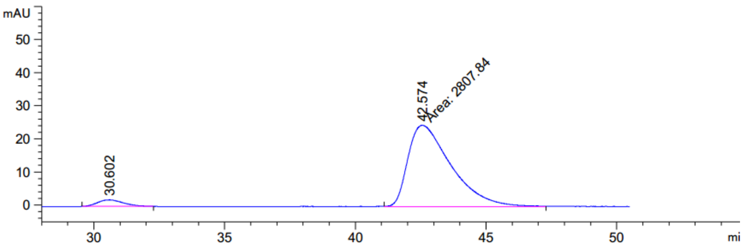

Peak #	RetTime [min]	Type	Width [min]	Area [mAU*s]	Height [mAU]	Area %
1	30.602	BB	0.8176	136.94196	1.98314	4.6503
2	42.574	MM	1.9099	2807.83984	24.50189	95.3497

Supplementary Figure 60. HPLC Chromatograph of chiral **5ak**

General Procedure for the Palladium-Catalyzed Asymmetric Tandem Heck/Carbonylation Desymmetrization of Cyclopentenes with Amines Exemplified by the Synthesis of 7aa

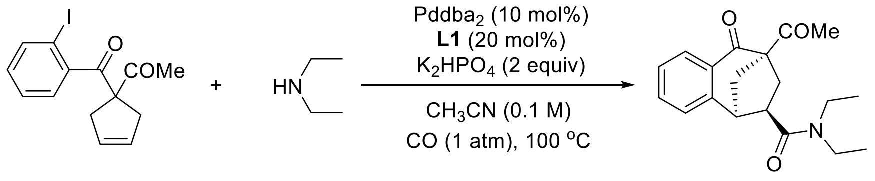

1a **6a** **7aa**

A sealed tube was charged with the substrate **1a** (34 mg, 0.1 mmol, 1 equiv), $Pddba_2$ (5.8 mg, 10 mol%), **L1** (13.4 mg, 20 mol%), and K_2HPO_4 (34.8 mg, 0.2 mmol, 2 equiv). The vial is thoroughly flushed with CO, and **6a** (14.6 mg, 0.2 mmol, 2 equiv), as well as CH_3CN (1 mL) were added under CO atmosphere. Then the reaction mixture was stirred at 100 °C for 36h. After the reaction vessel was cooled to room temperature, the solution was concentrated in *vacuo* and purified by careful chromatography on silica gel (200–300 mesh) (PE/EA = 2/1) to afford the desired product **7aa**.

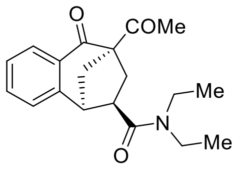

(5S,6R,8R)-8-acetyl-N,N-diethyl-9-oxo-6,7,8,9-tetrahydro-5H-5,8-methanobenzo[7]annulene-6-carboxamide (7aa)

22.1 mg, 71 % yield, >20:1 dr, 93% ee; R_f = 0.1 (PE/EA = 4/1); White solid, m.p. 100 – 102 ºC; $[\alpha]^{20}_{D}$ = +252 (c = 0.1, EA).

^{1}H NMR (400 MHz, $CDCl_3$) δ 8.04 (dd, J = 7.7, 1.4 Hz, 1H), 7.44 (td, J = 7.4, 1.5 Hz, 1H), 7.36 (td, J = 7.6, 1.3 Hz, 1H), 7.02 (dd, J = 7.5, 1.3 Hz, 1H), 3.78 – 3.69 (m, 1H), 3.62 (t, J = 4.8 Hz, 1H), 3.58 – 3.52 (m, 2H), 3.35 (dq, J = 14.3, 7.1 Hz, 1H), 2.87 (dq, J = 14.0, 7.1 Hz, 1H), 2.58 (d, J = 8.5 Hz, 2H), 2.46 (d, J = 11.7 Hz, 1H), 2.37 (s, 3H), 2.26 (dd, J = 11.8, 4.1 Hz, 1H), 1.27 (t, J = 7.1 Hz, 3H), 0.94 (t, J = 7.1 Hz, 3H).

^{13}C NMR (101 MHz, $CDCl_3$) δ 206.8, 197.5, 169.2, 144.5, 133.6, 131.0, 128.0, 127.7, 127.4, 68.5, 46.0, 45.5, 42.8, 41.5, 40.2, 30.3, 28.8, 14.8, 13.0.

HRMS (ESI) calcd. for $[C_{19}H_{23}NO_3+H]^+$ 314.1751, found 314.1749.

HPLC: Daicel Chiralcel AD-H, n-hexane/isopropanol 80/20, flow rate = 1.0 mL/min, uv-vis λ = 254 nm, t_{R1} = 8.9 min (minor), t_{R2} = 11.3 min (major).

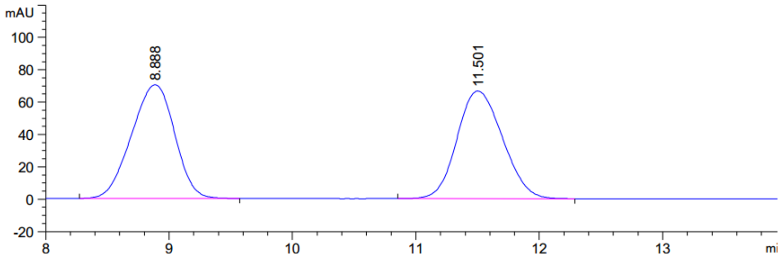

Peak #	RetTime [min]	Type	Width [min]	Area [mAU*s]	Height [mAU]	Area %
1	8.888	BB	0.3836	1708.99207	70.30553	49.9877
2	11.501	BB	0.4056	1709.82971	66.57976	50.0123

Supplementary Figure 61. HPLC Chromatograph of racemic **7aa**

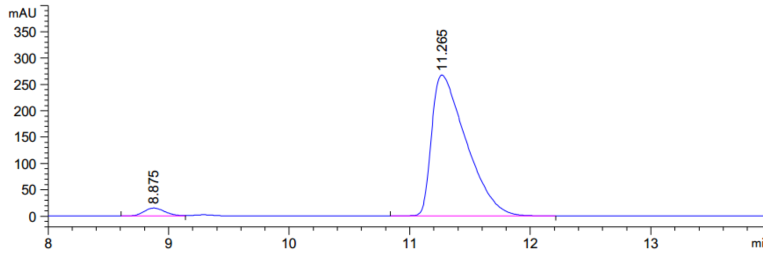

Peak #	RetTime [min]	Type	Width [min]	Area [mAU*s]	Height [mAU]	Area %
1	8.875	BV	0.1906	185.35800	15.16562	3.3630
2	11.265	BB	0.3002	5326.38184	267.67392	96.6370

Supplementary Figure 62. HPLC Chromatograph of chiral **7aa**

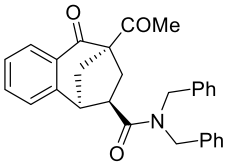

(5S,6R,8R)-8-acetyl-N,N-dibenzyl-9-oxo-6,7,8,9-tetrahydro-5H-5,8-methanobenzo[7]annulene-6-carboxamide (7ab)

42.7 mg, 98% yield, >20:1 dr, 94% ee; R_f = 0.2 (PE/EA = 4/1); Brown solid, m.p. 174 – 175 ºC; $[\alpha]^{20}_{D}$ = +37 (*c* = 0.1, EA).

^{1}H NMR (400 MHz, $CDCl_3$) δ 8.08 (dd, *J* = 5.7, 3.4 Hz, 1H), 7.43 – 7.40 (m, 4H), 7.37 – 7.29 (m, 4H), 7.19 (d, *J* = 7.1 Hz, 2H), 7.07 (dd, *J* = 6.4, 3.0 Hz, 2H), 6.87 (dd, *J* = 5.5, 3.2 Hz, 1H), 5.13 (d, *J* = 14.3 Hz, 1H), 4.95 (d, *J* = 17.1 Hz, 1H), 4.40 (d, *J* = 17.1 Hz, 1H), 3.68 (d, *J* = 14.3 Hz, 1H), 3.59 (ddd, *J* = 8.7, 7.6, 5.4 Hz, 1H), 3.52 (t, *J* = 4.7 Hz, 1H), 2.64 (d, *J* = 9.0 Hz, 2H), 2.41 (d, *J* = 11.9 Hz, 1H), 2.34 (s, 3H), 2.15 (dd, *J* = 11.8, 4.1 Hz, 1H).

^{13}C NMR (101 MHz, $CDCl_3$) δ 206.5, 197.4, 170.9, 144.3, 137.3, 136.2, 133.8, 130.9, 129.4, 129.2, 128.5, 128.1, 128.0, 127.9, 127.8, 127.7, 126.3, 68.5, 49.5, 48.6, 46.2, 45.8, 43.1, 30.7, 28.8.

HRMS (ESI) calcd. for $[C_{29}H_{27}NO_3+Na]^+$ 460.1883, found 460.1881.

HPLC: Daicel Chiralcel OD-H, *n*-hexane/isopropanol 80/20, flow rate = 1.0 mL/min, uv-vis λ = 254 nm, t_{R1} = 19.3 min (minor), t_{R2} = 23.9 min (major).

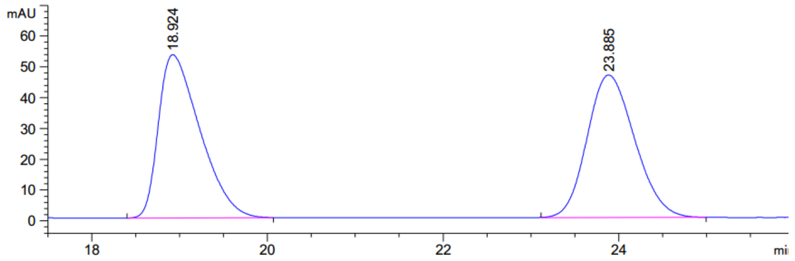

Peak #	RetTime [min]	Type	Width [min]	Area [mAU*s]	Height [mAU]	Area %
1	18.924	BB	0.4957	1744.75867	53.05250	49.9969
2	23.885	BB	0.5839	1744.97583	46.27977	50.0031

Supplementary Figure 63. HPLC Chromatograph of racemic **7ab**

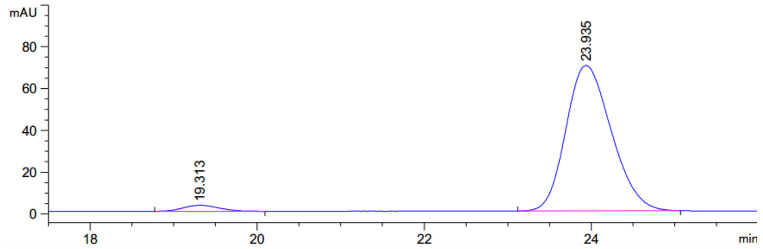

Peak #	RetTime [min]	Type	Width [min]	Area [mAU*s]	Height [mAU]	Area %
1	19.313	BB	0.4014	87.30909	2.91531	3.2087
2	23.935	BB	0.5837	2633.72974	69.56274	96.7913

Supplementary Figure 64. HPLC Chromatograph of chiral **7ab**

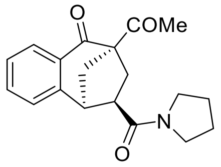

(5S,6R,8R)-8-acetyl-6-(pyrrolidine-1-carbonyl)-5,6,7,8-tetrahydro-9H-5,8-methanobenzo[7]annulen-9-one(7ac)

22.8 mg, 73% yield, >20:1 dr, 92% ee; R_f = 0.1 (PE/EA = 4/1); Brown solid, m.p. 154 – 156 ºC; $[\alpha]^{20}_{D}$ = +153 (*c* = 0.1, EA).

^{1}H NMR (400 MHz, $CDCl_3$) δ 8.03 (d, *J* = 7.7 Hz, 1H), 7.46 (t, *J* = 7.4 Hz, 1H), 7.36 (t, *J* = 7.6 Hz, 1H), 7.00 (d, *J* = 7.6 Hz, 1H), 3.75 – 3.69 (m, 2H), 3.61 (q, *J* = 9.4, 8.2 Hz, 1H), 3.51 (q, *J* = 7.5 Hz, 1H), 3.37 (dt, *J* = 13.4, 7.1 Hz, 1H), 3.14 (dt, *J* = 12.7, 6.9 Hz, 1H), 2.59 – 2.56 (m, 2H), 2.47 (d, *J* = 11.8 Hz, 1H), 2.37 (s, 3H), 2.24 (dd, *J* = 11.9, 4.2 Hz, 1H), 2.03 (q, *J* = 6.4 Hz, 2H), 1.86 (t, *J* = 7.1, 6.3 Hz, 2H).

^{13}C NMR (101 MHz, $CDCl_3$) δ 206.8, 197.5, 168.9, 144.8, 133.8, 130.9, 128.0, 127.7, 127.0, 68.5, 47.9, 46.4, 46.1, 44.6, 42.7, 29.5, 28.8, 26.4, 24.2.

HRMS (ESI) calcd. for $[C_{19}H_{21}NO_3+H]^+$ 312.1594, found 312.1593.

HPLC: Daicel Chiralcel AD-H, *n*-hexane/isopropanol 80/20, flow rate = 1.0 mL/min, uv-vis λ = 254 nm, t_{R1} = 11.7 min (minor), t_{R2} = 17.3 min (major).

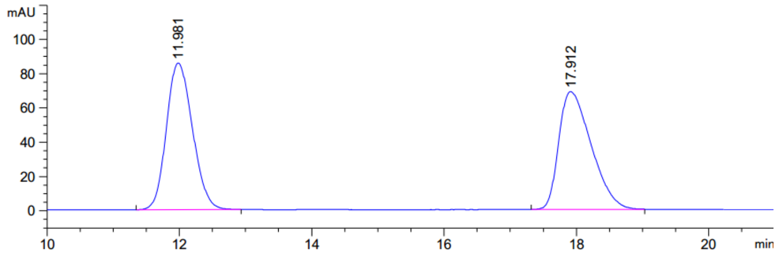

Peak #	RetTime [min]	Type	Width [min]	Area [mAU*s]	Height [mAU]	Area %
1	11.981	BB	0.4322	2349.89307	85.61943	50.1036
2	17.912	BB	0.5269	2340.17285	68.78484	49.8964

Supplementary Figure 65. HPLC Chromatograph of racemic **7ac**

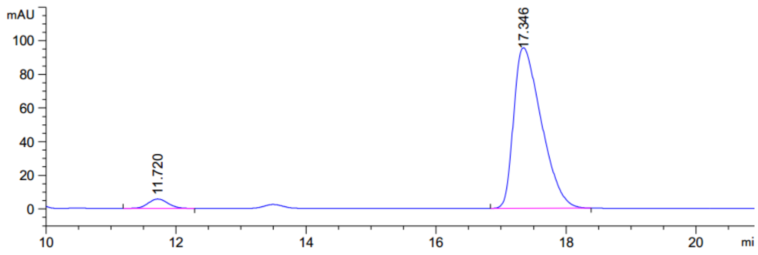

Peak #	RetTime [min]	Type	Width [min]	Area [mAU*s]	Height [mAU]	Area %
1	11.720	BB	0.3302	120.13693	5.60295	4.0863
2	17.346	BB	0.4566	2819.83716	95.45700	95.9137

Supplementary Figure 66. HPLC Chromatograph of chiral **7ac**

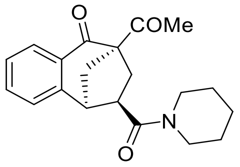

(5S,6R,8R)-8-acetyl-6-(piperidine-1-carbonyl)-5,6,7,8-tetrahydro-9H-5,8-methanobenzo[7]annulen-9-one (7ad)

25.5 mg, 78% yield, >20:1 dr, 94% ee; R_f = 0.3 (PE/EA = 1/1); Light yellow solid, m.p. 170 – 172 °C; $[\alpha]_D^{20}$ = +299 (c = 0.1, EA).

^{1}H NMR (400 MHz, $CDCl_3$) δ 8.03 (d, J = 7.7 Hz, 1H), 7.49 – 7.45 (m, 1H), 7.39 – 7.34 (m, 1H), 7.07 (d, J = 7.5 Hz, 1H), 3.67 – 3.60 (m, 4H), 3.40 – 3.31 (m, 2H), 2.65 – 2.57 (m, 2H), 2.45 (d, J = 11.9 Hz, 1H), 2.37 (d, J = 1.8 Hz, 3H), 2.25 (ddd, J = 11.8, 4.0, 1.8 Hz, 1H), 1.71 – 1.70 (m, 4H), 1.50 – 1.49 (m, 2H).

^{13}C NMR (101 MHz, $CDCl_3$) δ 206.8, 197.5, 168.4, 144.5, 133.6, 130.9, 128.0, 127.7, 127.4, 68.3, 46.5, 45.7, 45.6, 43.0, 42.7, 29.8, 28.8, 26.9, 25.6, 24.6.

HRMS (ESI) calcd. for $[C_{20}H_{23}NO_3+H]^+$ 326.1751, found 326.1750.

HPLC: Daicel Chiralcel AD-H, n-hexane/isopropanol 80/20, flow rate = 1.0 mL/min, uv-vis λ = 254 nm, t_{R1} = 10.6 min (minor), t_{R2} = 24.3 min (major).

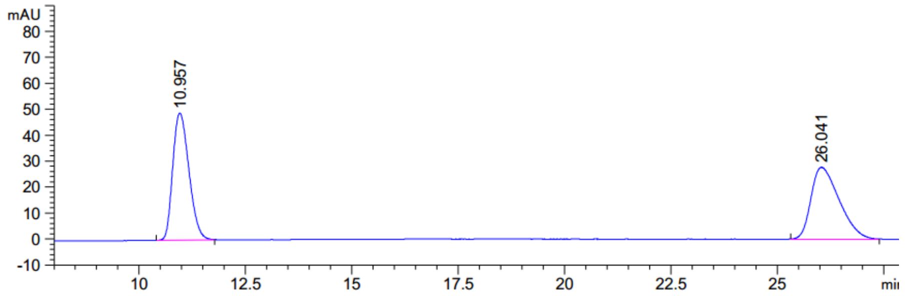

Peak #	RetTime [min]	Type	Width [min]	Area [mAU*s]	Height [mAU]	Area %
1	10.957	BB	0.4161	1293.09253	48.95950	50.3121
2	26.041	BB	0.6978	1277.04993	27.70530	49.6879

Supplementary Figure 67. HPLC Chromatograph of racemic **7ad**

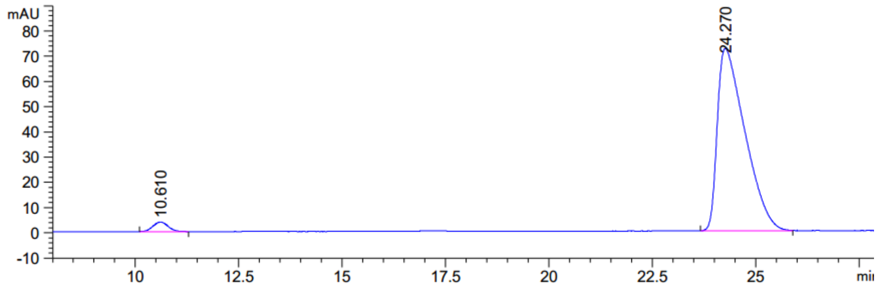

Peak #	RetTime [min]	Type	Width [min]	Area [mAU*s]	Height [mAU]	Area %
1	10.610	BB	0.3856	97.16087	3.78378	2.8123
2	24.270	BB	0.6877	3357.64355	72.31985	97.1877

Supplementary Figure 68. HPLC Chromatograph of chiral **7ad**

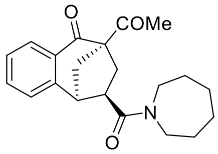

(5S,6R,8R)-8-acetyl-6-(azepane-1-carbonyl)-5,6,7,8-tetrahydro-9H-5,8-methanobenzo[7]annulen-9-one (7ae)

19.4 mg, 57% yield, >20:1 dr, 93% ee; R_f = 0.3 (PE/EA = 2/1); Light yellow solid, m.p. 123 – 124 °C; $[\alpha]_D^{20}$ = +188 (*c* = 0.1, EA).

^{1}H NMR (300 MHz, $CDCl_3$) δ 8.03 (dd, *J* = 7.7, 1.5 Hz, 1H), 7.46 (td, *J* = 7.4, 1.5 Hz, 1H), 7.36 (td, *J* = 7.6, 1.3 Hz, 1H), 7.04 (dd, *J* = 7.7, 1.1 Hz, 1H), 3.85 (dt, *J* = 14.1, 5.4 Hz, 1H), 3.67 – 3.58 (m, 2H), 3.54 – 3.45 (m, 2H), 3.08 (ddd, *J* = 13.9, 7.8, 3.2 Hz, 1H), 2.60 – 2.56 (m, 2H), 2.46 (d, *J* = 11.9 Hz, 1H), 2.36 (s, 3H), 2.25 (dd, *J* = 11.8, 3.9 Hz, 1H), 1.91 – 1.73 (m, 2H), 1.72 – 1.56 (m, 4H), 1.55 – 1.45 (m, 2H).

^{13}C NMR (75 MHz, $CDCl_3$) δ 206.8, 197.6, 169.9, 144.8, 133.6, 130.9, 127.9, 127.6, 127.3, 68.5, 47.7, 46.1, 45.9, 45.6, 42.8, 30.2, 29.8, 28.8, 27.5, 27.4, 26.6.

HRMS (ESI) calcd. for $[C_{21}H_{25}NO_3+H]^+$ 340.1907, found 340.1905.

HPLC: Daicel Chiralcel IA-3, *n*-hexane/isopropanol 70/30, flow rate = 0.5 mL/min, uv-vis λ = 254 nm, t_{R1} = 15.8 min (minor), t_{R2} = 27.0 min (major).

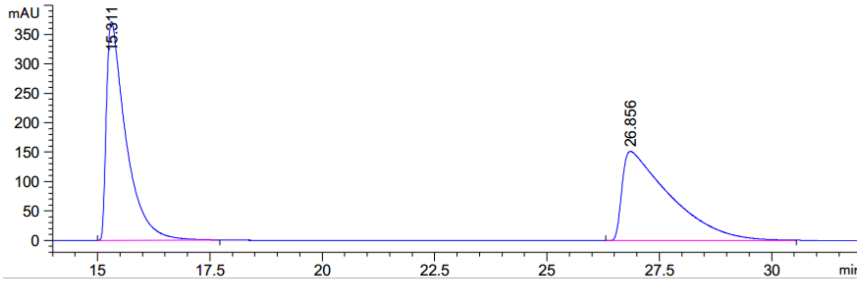

Peak #	RetTime [min]	Type	Width [min]	Area [mAU*s]	Height [mAU]	Area %
1	15.311	BB	0.4333	1.12577e4	371.70016	49.9891
2	26.856	BB	1.0003	1.12626e4	151.68198	50.0109

Supplementary Figure 69. HPLC Chromatograph of racemic **7ae**

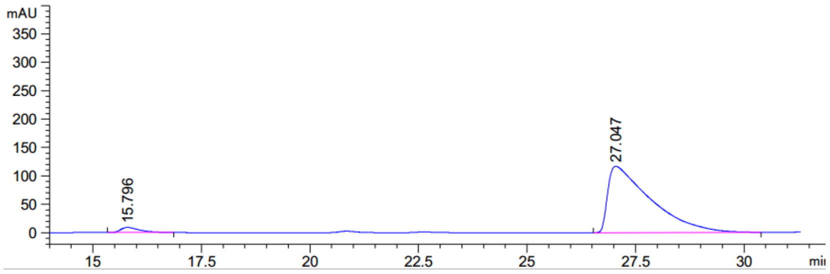

Peak #	RetTime [min]	Type	Width [min]	Area [mAU*s]	Height [mAU]	Area %
1	15.796	BB	0.4495	276.14847	8.96148	3.3254
2	27.047	BB	0.9452	8027.99561	116.90233	96.6746

Supplementary Figure 70. HPLC Chromatograph of chiral **7ae**

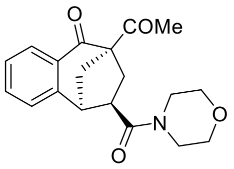

(5S,6R,8R)-8-acetyl-6-(morpholine-4-carbonyl)-5,6,7,8-tetrahydro-9H-5,8-methanobenzo[7]annulen-9-one (7af)

20.5 mg, 63% yield, >20:1 dr, 93% ee; R_f = 0.2 (PE/EA = 1/2); Light yellow solid, m.p. 178 – 180 °C; $[\alpha]_D^{20}$ = +151 (*c* = 0.1, EA).

^{1}H NMR (400 MHz, $CDCl_3$) δ 8.05 (dd, *J* = 7.7, 1.4 Hz, 1H), 7.49 (td, *J* = 7.5, 1.5 Hz, 1H), 7.39 (td, *J* = 7.5, 1.3 Hz, 1H), 7.08 (d, *J* = 7.5 Hz, 1H), 3.84 (dt, *J* = 11.8, 4.3 Hz, 1H), 3.77 (t, *J* = 4.9 Hz, 1H), 3.71 (q, *J* = 5.3, 4.7 Hz, 2H), 3.62 (q, *J* = 4.2 Hz, 4H), 3.50 (dt, *J* = 13.6, 4.6 Hz, 1H), 3.33 (dt, *J* = 13.6, 5.2 Hz, 1H), 2.60 (d, *J* = 8.4 Hz, 2H), 2.48 (d, *J* = 11.6 Hz, 1H), 2.37 (s, 3H), 2.25 (dd, *J* = 11.8, 3.7 Hz, 1H).

^{13}C NMR (101 MHz, $CDCl_3$) δ 206.5, 197.2, 169.0, 144.2, 133.8, 130.9, 128.2, 127.9, 127.1, 68.4, 67.0, 66.8, 46.1, 45.6, 45.5, 42.7, 42.3, 29.7, 28.8.

HRMS (ESI) calcd. for $[C_{19}H_{21}NO_4+H]^+$ 328.1543, found 328.1543.

HPLC: Daicel Chiralcel AD-H, *n*-hexane/isopropanol 70/30, flow rate = 1.0 mL/min, uv-vis λ = 254 nm, t_{R1} = 9.6 min (minor), t_{R2} = 12.0 min (major).

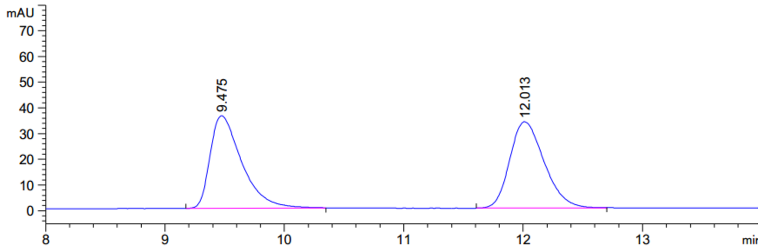

Peak #	RetTime [min]	Type	Width [min]	Area [mAU*s]	Height [mAU]	Area %
1	9.475	BB	0.2789	663.38141	36.02207	49.6916
2	12.013	BB	0.3114	671.61530	33.57582	50.3084

Supplementary Figure 71. HPLC Chromatograph of racemic **7af**

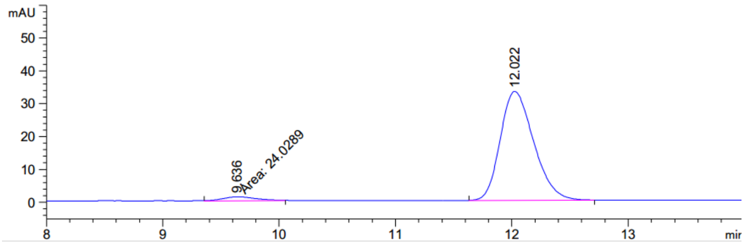

Peak #	RetTime [min]	Type	Width [min]	Area [mAU*s]	Height [mAU]	Area %
1	9.636	MM	0.3364	24.02895	1.19044	3.4968
2	12.022	BB	0.3114	663.13910	33.15278	96.5032

Supplementary Figure 72. HPLC Chromatograph of chiral **7af**

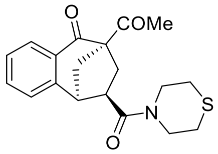

(5S,6R,8R)-8-acetyl-6-(thiomorpholine-4-carbonyl)-5,6,7,8-tetrahydro-9H-5,8-methanobenzo[7]annulen-9-one (7ag)

19.1 mg, 56% yield, >20:1 dr, 92% ee; R_f = 0.3 (PE/EA = 1/1); Brown solid, m.p. 168 – 170 ºC; $[\alpha]^{20}_{D}$ = +236 (*c* = 0.1, EA).

^{1}H NMR (400 MHz, $CDCl_3$) δ 8.04 (d, *J* = 7.7 Hz, 1H), 7.48 (t, *J* = 7.4 Hz, 1H), 7.38 (t, *J* = 7.5 Hz, 1H), 7.07 (d, *J* = 7.1 Hz, 1H), 4.04 – 3.90 (m, 2H), 3.80 – 3.75 (m, 1H), 3.63 – 3.59 (m, 3H), 2.76 (s, 2H), 2.61 – 2.59 (m, 3H), 2.47 (d, *J* = 11.3 Hz, 2H), 2.37 – 2.36 (m, 3H), 2.26 – 2.23 (m, 1H).

^{13}C NMR (101 MHz, $CDCl_3$) δ 206.5, 197.3, 168.9, 144.2, 133.7, 130.9, 128.2, 127.9, 127.3, 68.3, 48.1, 45.7, 45.6, 44.6, 42.7, 29.9, 28.8, 28.3, 27.4.

HRMS (ESI) calcd. for $[C_{19}H_{21}NO_3S+H]^+$ 344.1315, found 344.1314.

HPLC: Daicel Chiralcel IA-3, *n*-hexane/isopropanol 70/30, flow rate = 0.5 mL/min, uv-vis λ = 254 nm, t_{R1} = 21.6 min (minor), t_{R2} = 46.1 min (major).

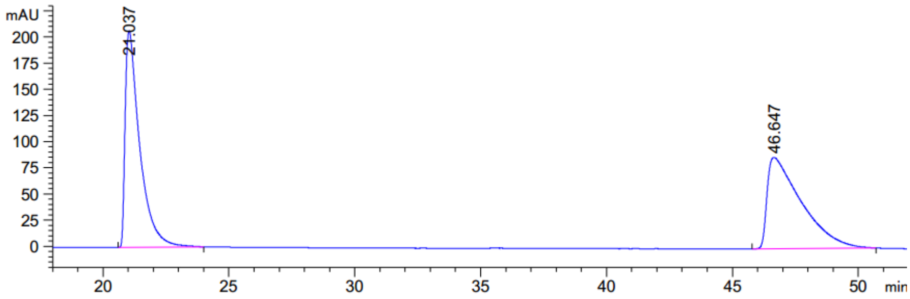

Peak #	RetTime [min]	Type	Width [min]	Area [mAU*s]	Height [mAU]	Area %
1	21.037	BB	0.5827	8328.01758	207.30510	50.2673
2	46.647	BB	1.2775	8239.45996	87.27084	49.7327

Supplementary Figure 73. HPLC Chromatograph of racemic **7ag**

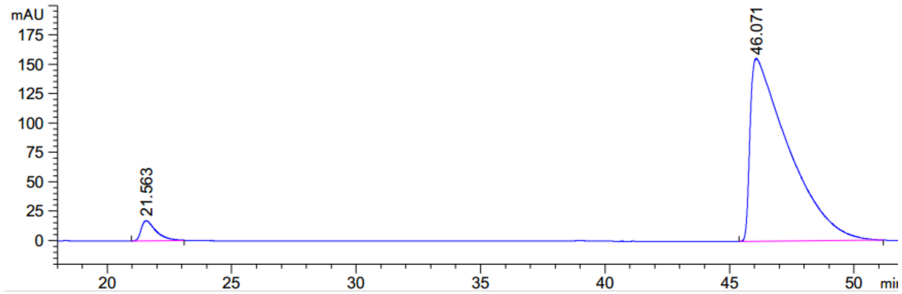

Peak #	RetTime [min]	Type	Width [min]	Area [mAU*s]	Height [mAU]	Area %
1	21.563	BB	0.5900	698.74689	16.98399	3.9999
2	46.071	BB	1.4116	1.67704e4	155.32928	96.0001

Supplementary Figure 74. HPLC Chromatograph of chiral **7ag**

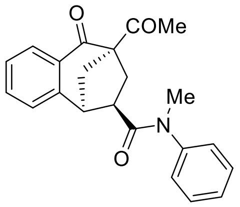

(5S,6R,8R)-8-acetyl-N-methyl-9-oxo-N-phenyl-6,7,8,9-tetrahydro-5H-5,8-methanobenzo[7]annulene-6-carboxamide (7ah)

29.7 mg, 86% yield, >20:1 dr, 92% ee; R_f = 0.4 (PE/EA = 2/1); White soild, m.p. 174 – 175 ºC; $[\alpha]_D^{20}$ = +194 (c = 0.1, EA).

^{1}H NMR (300 MHz, $CDCl_3$) δ 8.09 (dd, J = 7.8, 1.4 Hz, 1H), 7.60 – 7.52 (m, 3H), 7.47 – 7.40 (m, 2H), 7.35 (d, J = 7.1 Hz, 2H), 7.23 (d, J = 7.4 Hz, 1H), 3.46 (ddd, J = 10.0, 7.1, 5.4 Hz, 1H), 3.24 (t, J = 4.7 Hz, 1H), 3.13 (s, 3H), 2.53 (dd, J = 14.4, 10.0 Hz, 1H), 2.41 (dd, J = 14.4, 7.1 Hz, 1H), 2.32 – 2.27 (m, 4H), 1.92 (dd, J = 11.8, 4.1 Hz, 1H).

^{13}C NMR (75 MHz, $CDCl_3$) δ 206.5, 197.5, 170.6, 145.0, 143.7, 133.8, 130.9, 130.1, 128.3, 128.0, 127.8, 127.6, 127.2, 68.5, 46.4, 45.7, 42.8, 38.0, 29.9, 28.7.

HRMS (ESI) calcd. for $[C_{22}H_{21}NO_3+H]^+$ 348.1594, found 348.1591.

HPLC: Daicel Chiralcel AD-H, n-hexane/isopropanol 60/40, flow rate = 0.5 mL/min, uv-vis λ = 250 nm, t_{R1} = 23.5 min (minor), t_{R2} = 44.7 min (major).

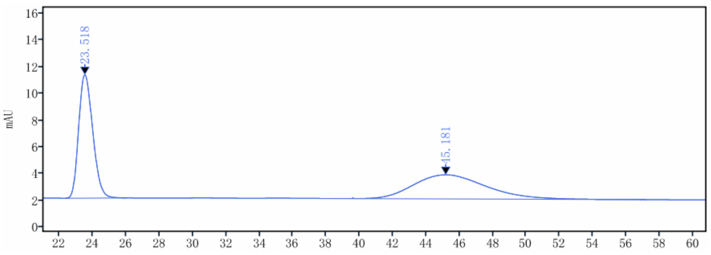

RetTime [min]	Type	Width [min]	Area [mAU*s]	Height [mAU]	Area %
23.518	BB	3.6883	542.08937	9.23019	49.9753
45.181	MM m	3.5225	542.62558	1.80029	50.0247

Supplementary Figure 75. HPLC Chromatograph of racemic **7ah**

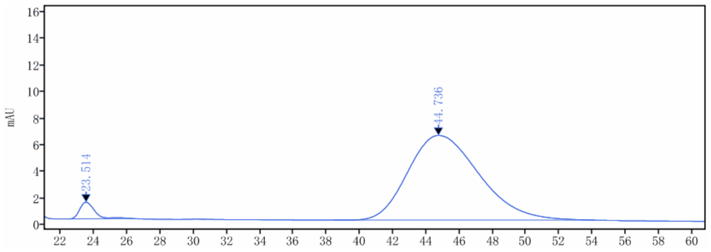

RetTime [min]	Type	Width [min]	Area [mAU*s]	Height [mAU]	Area %
23.514	MM m	0.7413	77.75476	1.24896	3.9383
44.736	MM m	3.4816	1896.57108	6.36076	96.0617

Supplementary Figure 76. HPLC Chromatograph of chiral 7**ah**

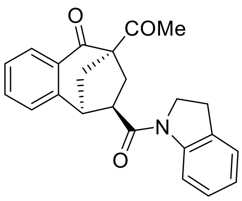

(5S,6R,8R)-8-acetyl-6-(indoline-1-carbonyl)-5,6,7,8-tetrahydro-9H-5,8-methanobenzo[7]annulen-9-one (7ai)

17.9 mg, 50% yield, >20:1 dr, 91% ee; R_f = 0.2 (PE/EA = 2/1); White solid, m.p. 205 – 207 ºC; $[\alpha]^{20}_{D}$ = +203 (*c* = 0.1, EA).

^{1}H NMR (300 MHz, $CDCl_3$) δ 8.13 – 8.10 (m, 1H), 7.82 (d, *J* = 8.0 Hz, 1H), 7.40 (dd, *J* = 5.8, 3.2 Hz, 2H), 7.24 (d, *J* = 7.3 Hz, 1H), 7.12 (t, *J* = 7.6 Hz, 1H), 7.02 (d, *J* = 6.1 Hz, 2H), 4.35 (dq, *J* = 17.8, 9.1 Hz, 2H), 3.83 (t, *J* = 4.9 Hz, 1H), 3.72 (q, *J* = 8.1 Hz, 1H), 3.32 (t, *J* = 8.4 Hz, 2H), 2.69 (d, *J* = 9.2 Hz, 2H), 2.57 (d, *J* = 11.9 Hz, 1H), 2.43 (s, 3H), 2.34 (dd, *J* = 11.8, 4.1 Hz, 1H).

^{13}C NMR (75 MHz, $CDCl_3$) δ 205.9, 196.7, 168.1, 143.7, 142.4, 133.4, 130.4, 130.3, 127.6, 127.3, 127.0, 126.7, 123.9, 123.3, 116.8, 67.9, 48.3, 47.6, 44.6, 42.3, 29.0, 28.2, 27.9.

HRMS (ESI) calcd. for $[C_{23}H_{21}NO_3+H]^+$ 360.1594, found 360.1590.

HPLC: Daicel Chiralcel AD-H, *n*-hexane/isopropanol 70/30, flow rate = 1.0 mL/min, uv-vis λ = 254 nm, t_{R1} = 11.8 min (minor), t_{R2} = 19.6 min (major).

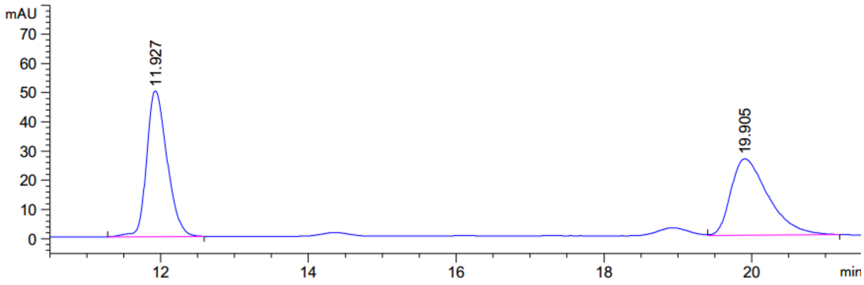

Peak #	RetTime [min]	Type	Width [min]	Area [mAU*s]	Height [mAU]	Area %
1	11.927	BB	0.2963	958.91803	49.87128	51.0131
2	19.905	VB	0.5353	920.82916	26.12227	48.9869

Supplementary Figure 77. HPLC Chromatograph of racemic **7ai**

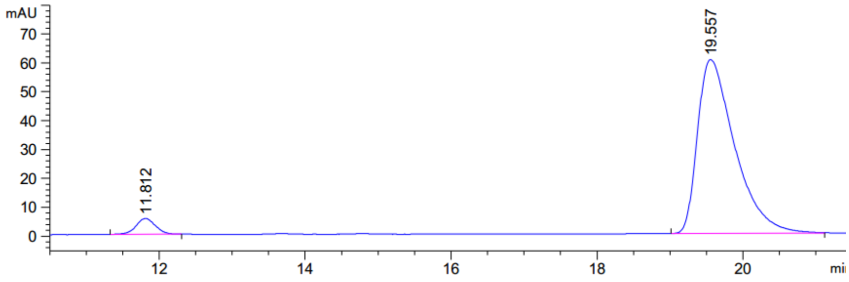

Peak #	RetTime [min]	Type	Width [min]	Area [mAU*s]	Height [mAU]	Area %
1	11.812	BB	0.2876	103.60993	5.50552	4.6434
2	19.557	BB	0.5327	2127.72314	60.14845	95.3566

Supplementary Figure 78. HPLC Chromatograph of chiral **7ai**

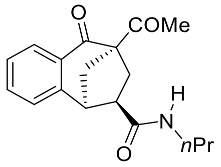

(5S,6R,8R)-8-acetyl-9-oxo-N-propyl-6,7,8,9-tetrahydro-5H-5,8-methanobenzo[7]annulene-6-carboxamide (7aj)

15.4 mg, 51% yield, >20:1 dr, 93% ee; R_f = 0.3 (PE/EA = 1/1); Light yellow solid, m.p. 122 – 124 °C; $[\alpha]_D^{20}$ = +250 (*c* = 0.1, EA).

^{1}H NMR (400 MHz, $CDCl_3$) δ 8.02 (d, *J* = 7.6 Hz, 1H), 7.49 (td, *J* = 7.4, 1.5 Hz, 1H), 7.38 (td, *J* = 7.7, 1.3 Hz, 1H), 7.21 (d, *J* = 7.5 Hz, 1H), 5.70 (s, 1H), 3.70 (t, *J* = 4.9 Hz, 1H), 3.31 (ddd, *J* = 10.5, 7.2, 5.5 Hz, 1H), 3.03 – 2.97 (m, 2H), 2.72 (dd, *J* = 14.6, 10.4 Hz, 1H), 2.48 (d, *J* = 12.0 Hz, 1H), 2.34 (s, 3H), 2.22 – 2.12 (m, 2H), 1.33 (q, *J* = 7.1 Hz, 2H), 0.79 (t, *J* = 7.4 Hz, 3H).

^{13}C NMR (101 MHz, $CDCl_3$) δ 206.3, 197.6, 170.2, 144.8, 133.9, 130.2, 128.4, 128.0, 127.6, 68.8, 49.6, 46.5, 42.6, 41.3, 28.8, 28.6, 22.8, 11.3.

HRMS (ESI) calcd. for $[C_{18}H_{21}NO_3+H]^+$ 300.1594, found 300.1594.

HPLC: Daicel Chiralcel OD-H, *n*-hexane/isopropanol 80/20, flow rate = 1.0 mL/min, uv-vis λ = 254 nm, t_{R1} = 8.7 min (major), t_{R2} = 11.9 min (minor).

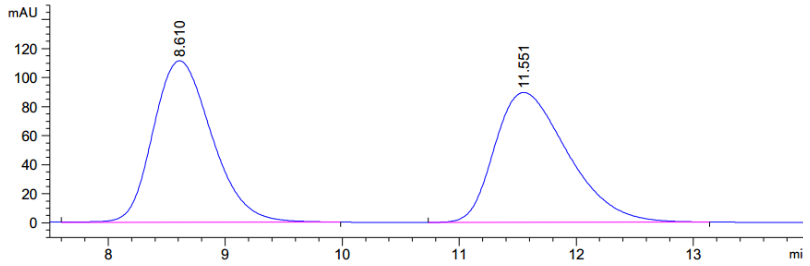

Peak #	RetTime [min]	Type	Width [min]	Area [mAU*s]	Height [mAU]	Area %
1	8.610	VB	0.5473	3882.80078	111.20850	49.9779
2	11.551	BB	0.6671	3886.23486	89.41330	50.0221

Supplementary Figure 79. HPLC Chromatograph of racemic **7aj**

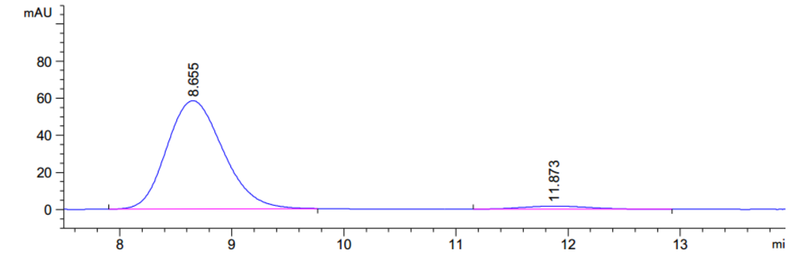

Peak #	RetTime [min]	Type	Width [min]	Area [mAU*s]	Height [mAU]	Area %
1	8.655	BB	0.5477	2040.44739	58.39203	96.6196
2	11.873	BB	0.4939	71.38796	1.70708	3.3804

Supplementary Figure 80. HPLC Chromatograph of chiral **7aj**

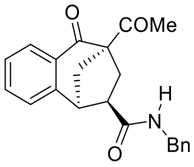

(5S,6R,8R)-8-acetyl-N-benzyl-9-oxo-6,7,8,9-tetrahydro-5H-5,8-methanobenzo[7]annulene-6-carboxamide (7ak)

24.0 mg, 69% yield, >20:1 dr, 94% ee; R_f = 0.3 (PE/EA = 1/1); Light yellow oil; $[\alpha]_D^{20}$ = +214 (*c* = 0.1, EA).

^{1}H NMR (300 MHz, $CDCl_3$) δ 8.00 (dd, *J* = 7.4, 1.8 Hz, 1H), 7.44 – 7.34 (m, 2H), 7.27 – 7.25 (m, 3H), 7.09 (dd, *J* = 7.4, 1.5 Hz, 2H), 7.05 – 7.02 (m, 1H), 6.00 (t, *J* = 4.9 Hz, 1H), 4.20 (dd, *J* = 5.7, 2.1 Hz, 2H), 3.68 (t, *J* = 4.8 Hz, 1H), 3.32 (ddd, *J* = 10.4, 7.2, 5.5 Hz, 1H), 2.71 (dd, *J* = 14.6, 10.4 Hz, 1H), 2.45 (d, *J* = 11.8 Hz, 1H), 2.32 (s, 3H), 2.19 – 2.11 (m, 2H).

^{13}C NMR (75 MHz, $CDCl_3$) δ 206.3, 197.5, 170.2, 144.6, 138.0, 134.0, 130.2, 128.6, 128.5, 128.0, 128.0, 127.7, 127.5, 68.8, 49.6, 46.4, 43.6, 42.6, 28.7, 28.6.

HRMS (ESI) calcd. for $[C_{22}H_{21}NO_3+H]^+$ 348.1594, found 348.1594.

HPLC: Daicel Chiralcel OD-H, *n*-hexane/isopropanol 70/30, flow rate = 1.0 mL/min, uv-vis λ = 254 nm, t_{R1} = 10.2 min (major), t_{R2} = 14.6 min (minor).

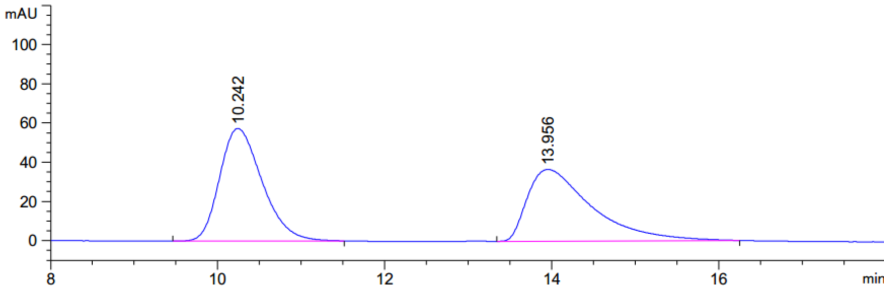

Peak #	RetTime [min]	Type	Width [min]	Area [mAU*s]	Height [mAU]	Area %
1	10.242	BB	0.5489	2032.56348	57.43769	50.8466
2	13.956	BB	0.7904	1964.87732	36.59425	49.1534

Supplementary Figure 81. HPLC Chromatograph of racemic **7ak**

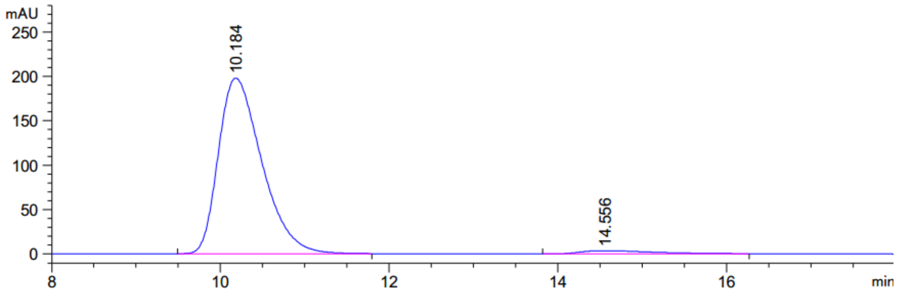

Peak #	RetTime [min]	Type	Width [min]	Area [mAU*s]	Height [mAU]	Area %
1	10.184	VB	0.5514	7077.95996	197.81886	96.9955
2	14.556	BB	0.7167	219.24426	3.63990	3.0045

Supplementary Figure 82. HPLC Chromatograph of chiral **7ak**

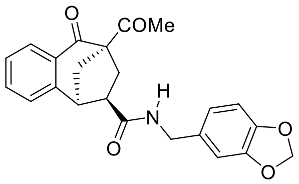

(5S,6R,8R)-8-acetyl-N-(benzo[d][1,3]dioxol-5-ylmethyl)-9-oxo-6,7,8,9-tetrahydro-5H-5,8-methanobenzo[7]annulene-6-carboxamide (7al)

22.8 mg, 58% yield, >20:1 dr, 92% ee; R_f = 0.2 (PE/EA = 1/1); White solid, m.p. 173 – 175 °C; $[\alpha]^{20}_{D}$ = +264 (*c* = 0.1, EA).

^{1}H NMR (300 MHz, $CDCl_3$) δ 7.96 (dd, *J* = 7.6, 1.5 Hz, 1H), 7.43 (td, *J* = 7.4, 1.6 Hz, 1H), 7.34 (td, *J* = 7.5, 1.3 Hz, 1H), 7.05 (dd, *J* = 7.5, 1.3 Hz, 1H), 6.67 (d, *J* = 8.3 Hz, 1H), 6.51 – 6.49 (m, 3H), 5.92 (dd, *J* = 4.1, 1.3 Hz, 2H), 4.10 – 3.97 (m, 2H), 3.61 (t, *J* = 4.8 Hz, 1H), 3.27 (ddd, *J* = 10.4, 7.0, 5.6 Hz, 1H), 2.66 (dd, *J* = 14.6, 10.4 Hz, 1H), 2.42 (d, *J* = 11.7 Hz, 1H), 2.30 (s, 3H), 2.21 – 2.10 (m, 2H).

^{13}C NMR (75 MHz, $CDCl_3$) δ 206.5, 197.7, 170.4, 147.7, 146.9, 144.7, 134.0, 132.1, 130.1, 128.4, 128.0, 127.6, 121.1, 108.6, 108.1, 101.1, 68.8, 49.2, 46.4, 43.3, 42.7, 28.6.

HRMS (ESI) calcd. for $[C_{23}H_{21}NO_5+H]^+$ 392.1492, found 392.1488.

HPLC: Daicel Chiralcel OJ-H, *n*-hexane/isopropanol 70/30, flow rate = 0.5 mL/min, uv-vis λ = 254 nm, t_{R1} = 40.7 min (minor), t_{R2} = 48.3 min (major).

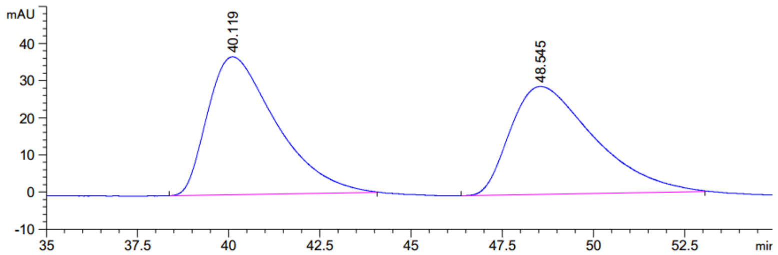

Peak #	RetTime [min]	Type	Width [min]	Area [mAU*s]	Height [mAU]	Area %
1	40.119	BB	1.5580	4854.54834	37.11366	51.1989
2	48.545	BB	1.8674	4627.19775	29.03274	48.8011

Supplementary Figure 83. HPLC Chromatograph of racemic **7al**

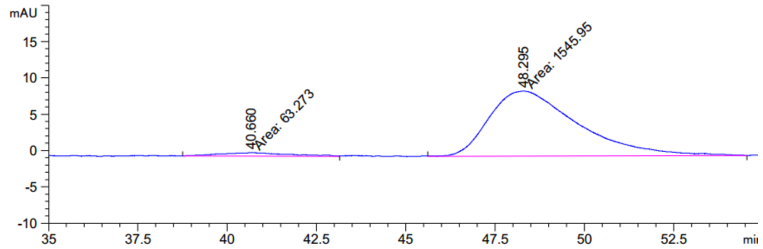

Peak #	RetTime [min]	Type	Width [min]	Area [mAU*s]	Height [mAU]	Area %
1	40.660	MM	2.2000	63.27300	4.79338e-1	3.9319
2	48.295	MM	2.8824	1545.94934	8.93908	96.0681

Supplementary Figure 84. HPLC Chromatograph of chiral **7al**

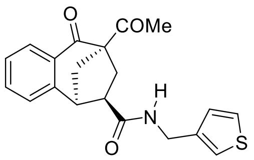

(5S,6R,8R)-8-acetyl-9-oxo-N-(thiophen-3-ylmethyl)-6,7,8,9-tetrahydro-5H-5,8-methanobenzo[7]annulene-6-carboxamide (7am)

21.7 mg, 61% yield, >20:1 dr, 93% ee; R_f = 0.2 (PE/EA = 1/1); Light yellow solid, m.p. 142 – 144 °C; $[\alpha]_D^{20}$ = +274 (*c* = 0.1, EA).

^{1}H NMR (400 MHz, $CDCl_3$) δ 8.00 (d, *J* = 7.7 Hz, 1H), 7.42 (td, *J* = 7.4, 1.6 Hz, 1H), 7.36 (t, *J* = 7.5 Hz, 1H), 7.23 (dd, *J* = 4.9, 3.0 Hz, 1H), 7.08 (d, *J* = 7.3 Hz, 1H), 6.91 – 6.90 (m, 1H), 6.80 (d, *J* = 4.9 Hz, 1H), 6.13 (t, *J* = 5.7 Hz, 1H), 4.20 (d, *J* = 5.0 Hz, 2H), 3.66 (t, *J* = 4.8 Hz, 1H), 3.31 (dt, *J* = 10.2, 6.9 Hz, 1H), 2.70 (dd, *J* = 14.6, 10.4 Hz, 1H), 2.45 (d, *J* = 11.8 Hz, 1H), 2.31 (s, 3H), 2.18 – 2.13 (m, 2H).

^{13}C NMR (101 MHz, $CDCl_3$) δ 206.3, 197.6, 170.2, 144.6, 138.8, 134.0, 130.2, 128.4, 128.0, 127.7, 127.5, 126.3, 122.6, 68.8, 49.4, 46.4, 42.7, 38.6, 28.6, 28.6.

HRMS (ESI) calcd. for $[C_{20}H_{19}NO_3S+H]^+$ 354.1158, found 354.1160.

HPLC: Daicel Chiralcel OD-H, *n*-hexane/isopropanol 70/30, flow rate = 0.5 mL/min, uv-vis λ = 254 nm, t_{R1} = 21.2 min (major), t_{R2} = 27.7 min (minor).

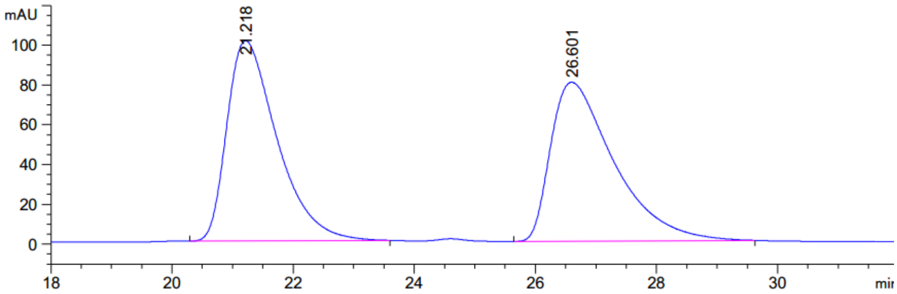

Peak #	RetTime [min]	Type	Width [min]	Area [mAU*s]	Height [mAU]	Area %
1	21.218	BB	0.8783	5804.55469	100.73049	49.9204
2	26.601	BB	1.0557	5823.06689	79.95158	50.0796

Supplementary Figure 85. HPLC Chromatograph of racemic **7am**

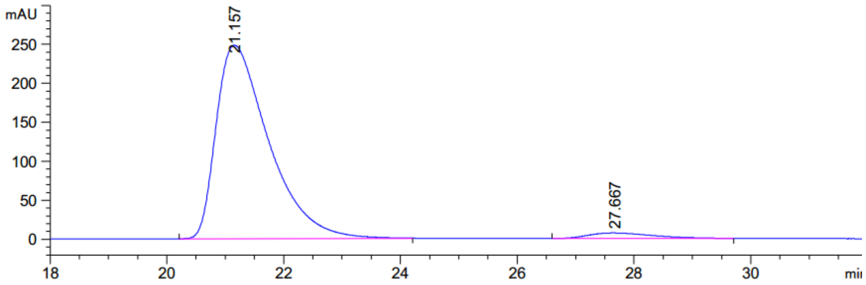

Peak #	RetTime [min]	Type	Width [min]	Area [mAU*s]	Height [mAU]	Area %
1	21.157	BB	0.9486	1.54986e4	248.17043	96.5339
2	27.667	BB	0.9171	556.48810	7.18901	3.4661

Supplementary Figure 86. HPLC Chromatograph of chiral **7am**

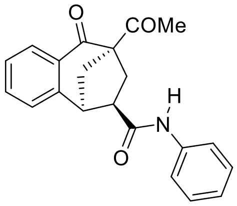

(5S,6R,8R)-8-acetyl-9-oxo-N-phenyl-6,7,8,9-tetrahydro-5H-5,8-methanobenzo[7]annulene-6-carboxamide (7an)

27.8 mg, 83% yield, >20:1 dr, 93% ee; R_f = 0.2 (PE/EA = 2/1); White soild, m.p. 171 – 172 ºC; $[\alpha]^{20}_{D}$ = +91 (c = 0.1, EA).

^{1}H NMR (300 MHz, $CDCl_3$) δ 8.07 (dd, J = 7.4, 1.7 Hz, 1H), 7.48 – 7.36 (m, 2H), 7.25 – 7.17 (m, 5H), 7.10 – 7.05 (m, 1H), 3.77 (t, J = 4.8 Hz, 1H), 3.44 (dt, J = 10.4, 6.4 Hz, 1H), 2.76 (dd, J = 14.7, 10.3 Hz, 1H), 2.52 (d, J = 11.8 Hz, 1H), 2.37 – 2.30 (m, 4H), 2.24 (dd, J = 11.8, 4.1 Hz, 1H).

^{13}C NMR (75 MHz, $CDCl_3$) δ 206.3, 197.4, 168.7, 144.2, 137.3, 134.1, 130.3, 129.0, 128.3, 128.2, 127.9, 124.6, 120.2, 68.7, 50.4, 46.7, 42.7, 28.7, 28.7.

HRMS (ESI) calcd. for $[C_{21}H_{19}NO_3+H]^+$ 334.1438, found 334.1433.

HPLC: Daicel Chiralcel AD-H, n-hexane/isopropanol 70/30, flow rate = 0.5 mL/min, uv-vis λ = 250 nm, t_{R1} = 11.3 min (minor), t_{R2} = 16.4 min (major).

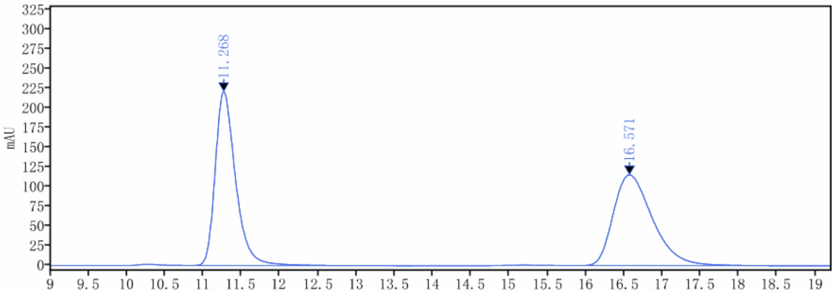

RetTime [min]	Type	Width [min]	Area [mAU*s]	Height [mAU]	Area %
11.268	BB	2.8900	4097.99365	221.06171	49.9059
16.571	BB	3.8333	4113.44835	115.46905	50.0941

Supplementary Figure 87. HPLC Chromatograph of racemic **7an**

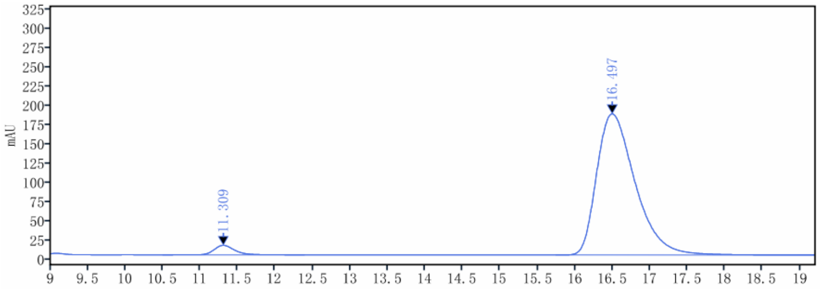

RetTime [min]	Type	Width [min]	Area [mAU*s]	Height [mAU]	Area %
11.309	BM m	0.2911	236.69159	12.32044	3.4901
16.497	BB	4.2600	6545.11502	183.40610	96.5099

Supplementary Figure 88. HPLC Chromatograph of chiral **7an**

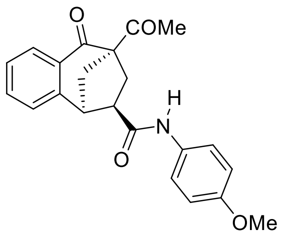

(5S,6R,8R)-8-acetyl-N-(4-methoxyphenyl)-9-oxo-6,7,8,9-tetrahydro-5H-5,8-methanobenzo[7]annulene-6-carboxamide (7ao)

31.3 mg, 86% yield, >20:1 dr, 93% ee; R_f = 0.1 (PE/EA = 2/1); White soild, m.p. 172 – 173 ºC; $[\alpha]^{20}_{D}$ = +226 (c = 0.1, EA).

^{1}H NMR (300 MHz, Acetone-d_6) δ 9.12 (s, 1H), 7.91 (dd, J = 7.6, 1.6 Hz, 1H), 7.42 (td, J = 7.4, 1.6 Hz, 1H), 7.37 – 7.28 (m, 3H), 7.24 (d, J = 7.5 Hz, 1H), 6.76 (d, J = 9.0 Hz, 2H), 3.91 (t, J = 4.9 Hz, 1H), 3.70 (s, 3H), 3.62 (dt, J = 10.4, 6.3 Hz, 1H), 2.65 (dd, J = 14.4, 10.4 Hz, 1H), 2.49 (d, J = 11.7 Hz, 1H), 2.32 – 2.26 (m, 2H), 2.24 (s, 3H).

^{13}C NMR (75 MHz, Acetone-d_6) δ 205.0, 197.7, 168.4, 155.8, 145.5, 133.6, 132.3, 130.7, 128.2, 127.6, 126.9, 121.1, 113.6, 68.9, 54.7, 49.5, 46.3, 42.5, 27.8, 27.7.

HRMS (ESI) calcd. for $[C_{22}H_{21}NO_4+Na]^+$ 386.1363, found 386.1359.

HPLC: Daicel Chiralcel AD-H, n-hexane/isopropanol 85/15, flow rate = 0.5 mL/min, uv-vis λ = 250 nm, t_{R1} = 45.2 min (minor), t_{R2} = 51.3 min (major).

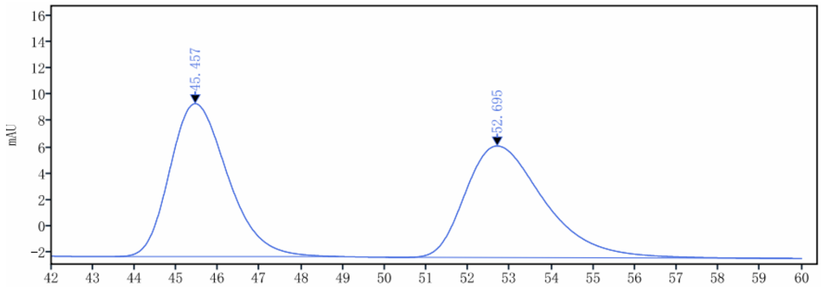

RetTime [min]	Type	Width [min]	Area [mAU*s]	Height [mAU]	Area %
45.457	BM m	1.4163	1117.51820	11.59610	50.0017
52.695	MM m	1.5662	1117.44415	8.46457	49.9983

Supplementary Figure 89. HPLC Chromatograph of racemic **7ao**

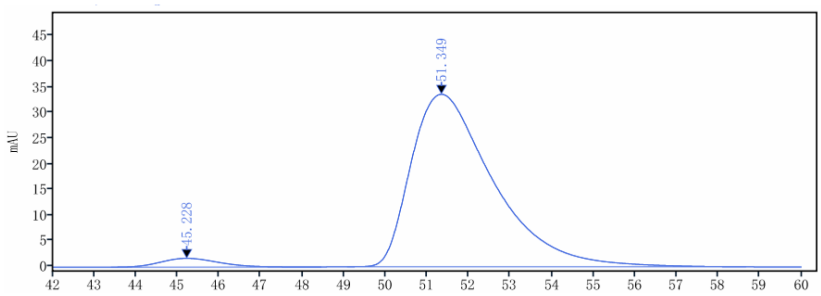

RetTime [min]	Type	Width [min]	Area [mAU*s]	Height [mAU]	Area %
45.228	MM m	1.1248	164.08869	1.70807	3.4269
51.349	MM m	2.0271	4624.13731	33.61009	96.5731

Supplementary Figure 90. HPLC Chromatograph of chiral **7ao**

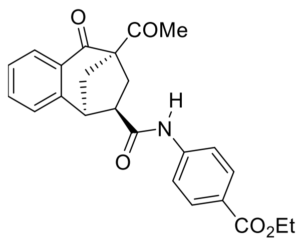

ethyl 4-((5S,6R,8R)-8-acetyl-9-oxo-6,7,8,9-tetrahydro-5H-5,8-methanobenzo[7]annulene-6-carboxamido)benzoate (7ap)

22.0 mg, 54% yield, >20:1 dr, 89% ee; R_f = 0.2 (PE/EA = 1/1); White soild, m.p. 206 – 207 ºC; $[\alpha]_D^{20}$ = +234 (*c* = 0.1, EA).

^{1}H NMR (300 MHz, $CDCl_3$) δ 8.08 (dd, *J* = 7.2, 2.0 Hz, 1H), 7.94 (d, *J* = 8.7 Hz, 2H), 7.83 (s, 1H), 7.47 – 7.38 (m, 2H), 7.35 (d, *J* = 8.7 Hz, 2H), 7.16 (dd, *J* = 7.1, 1.7 Hz, 1H), 4.36 (q, *J* = 7.1 Hz, 2H), 3.81 (t, *J* = 4.8 Hz, 1H), 3.51 (dt, *J* = 10.3, 6.3 Hz, 1H), 2.78 (dd, *J* = 14.7, 10.3 Hz, 1H), 2.56 (d, *J* = 11.8 Hz, 1H), 2.47 – 2.33 (m, 4H), 2.27 (dd, *J* = 11.8, 4.1 Hz, 1H), 1.39 (t, *J* = 7.1 Hz, 3H).

^{13}C NMR (75 MHz, $CDCl_3$) δ 206.5, 197.4, 169.1, 166.2, 144.1, 141.6, 134.2, 130.7, 130.3, 128.4, 128.0, 127.9, 126.1, 119.1, 68.8, 61.0, 50.5, 46.7, 42.7, 28.7, 28.7, 14.4.

HRMS (ESI) calcd. for $[C_{24}H_{23}NO_5 +H]^+$ 406.1649, found 406.1650.

HPLC: Daicel Chiralcel AD-H, *n*-hexane/isopropanol 90/10, flow rate = 1.0 mL/min, uv-vis λ = 250 nm, t_{R1} = 34.7 min (minor), t_{R2} = 41.0 min (major).

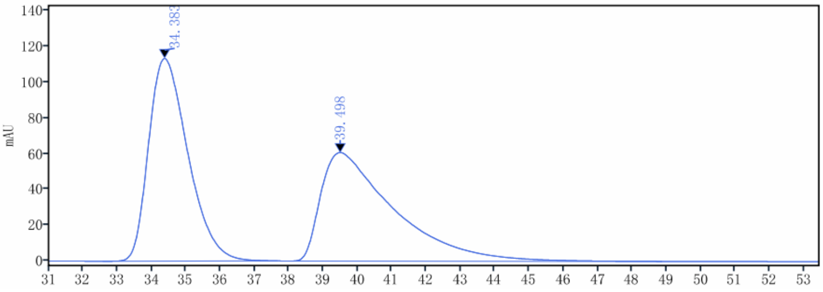

RetTime [min]	Type	Width [min]	Area [mAU*s]	Height [mAU]	Area %
34.383	MM m	1.2390	9022.52687	113.42632	50.1675
39.498	BB	10.8533	8962.28514	60.77771	49.8325

Supplementary Figure 91. HPLC Chromatograph of racemic **7ap**

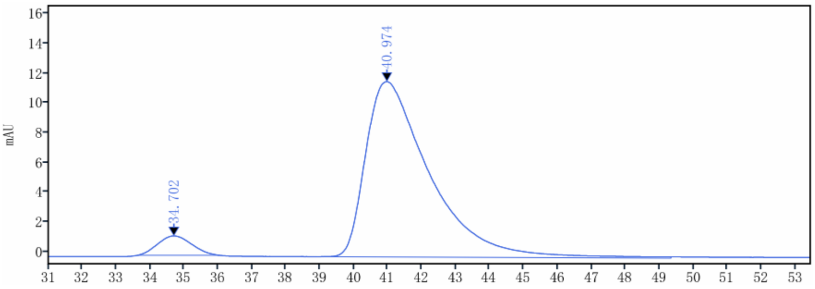

RetTime [min]	Type	Width [min]	Area [mAU*s]	Height [mAU]	Area %
34.702	MM m	0.8138	90.14072	1.30031	5.4993
40.974	MM m	1.7876	1548.98044	11.80711	94.5007

Supplementary Figure 92. HPLC Chromatograph of chiral **7ap**

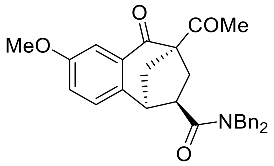

(5S,6R,8R)-8-acetyl-N,N-dibenzyl-2-methoxy-9-oxo-6,7,8,9-tetrahydro-5H-5,8-methanobenzo[7]annulene-6-carboxamide (7fb)

32.3 mg, 69% yield, >20:1 dr, 97% ee; R_f = 0.3 (PE/EA = 1/1); Brown solid, m.p. 124 – 126 ºC; $[\alpha]^{20}_{D}$ = +285 (*c* = 0.1, EA).

^{1}H NMR (300 MHz, $CDCl_3$) δ 7.56 (d, *J* = 2.8 Hz, 1H), 7.44 – 7.29 (m, 6H), 7.19 (d, *J* = 7.1 Hz, 2H), 7.08 (d, *J* = 3.6 Hz, 2H), 6.96 (dd, *J* = 8.3, 2.8 Hz, 1H), 6.77 (d, *J* = 8.3 Hz, 1H), 5.14 (d, *J* = 14.3 Hz, 1H), 4.95 (d, *J* = 17.1 Hz, 1H), 4.40 (d, *J* = 17.1 Hz, 1H), 3.86 (s, 3H), 3.69 (d, *J* = 14.3 Hz, 1H), 3.60 – 3.52 (m, 1H), 3.48 (t, *J* = 4.6 Hz, 1H), 2.62 (dd, *J* = 8.2, 3.9 Hz, 2H), 2.39 – 2.33 (m, 4H), 2.12 (dd, *J* = 11.8, 4.1 Hz, 1H).

^{13}C NMR (75 MHz, $CDCl_3$) δ 206.4, 197.3, 171.0, 159.2, 137.2, 136.8, 136.2, 131.8, 129.3, 129.2, 128.9, 128.4, 127.9, 127.6, 126.3, 121.7, 110.0, 68.3, 55.4, 49.5, 48.6, 45.7, 45.4, 43.5, 30.6, 28.6..

HRMS (ESI) calcd. for $[C_{30}H_{29}NO_4+H]^+$ 468.2169, found 468.2166.

HPLC: Daicel Chiralcel AD-H, *n*-hexane/isopropanol 80/20, flow rate = 1.0 mL/min, uv-vis λ = 254 nm, t_{R1} = 18.4 min (minor), t_{R2} = 27.2 min (major).

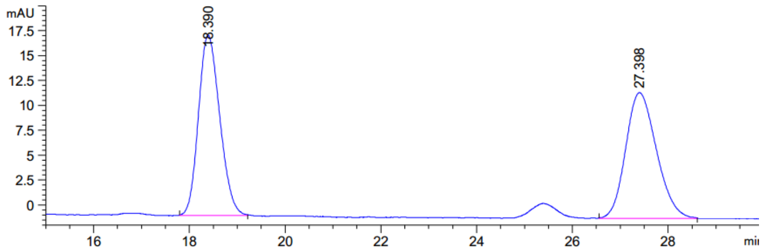

Peak #	RetTime [min]	Type	Width [min]	Area [mAU*s]	Height [mAU]	Area %
1	18.390	BB	0.4827	559.75580	18.10404	49.8980
2	27.398	BB	0.6342	562.04401	12.60582	50.1020

Supplementary Figure 93. HPLC Chromatograph of racemic **7fb**

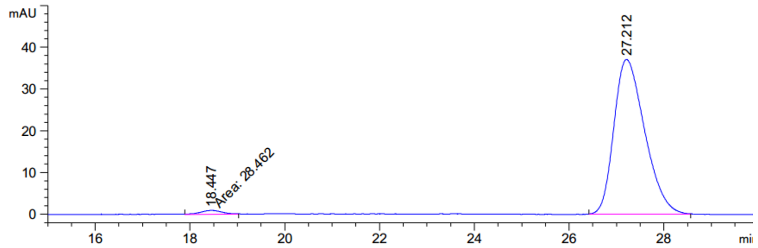

Peak #	RetTime [min]	Type	Width [min]	Area [mAU*s]	Height [mAU]	Area %
1	18.447	MM	0.5214	28.46198	9.09706e-1	1.6727
2	27.212	BB	0.6688	1673.12122	37.05694	98.3273

Supplementary Figure 94. HPLC Chromatograph of chiral **7fb**

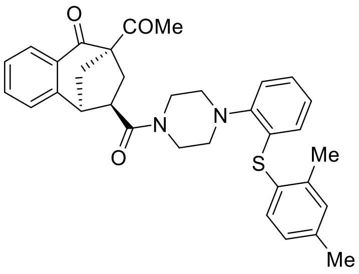

(5S,6R,8R)-8-acetyl-6-(4-(2-((2,4-dimethylphenyl)thio)phenyl)piperazine-1-carbonyl)-5,6,7,8-tetrahydro-9H-5,8-methanobenzo[7]annulen-9-one (7aq)

26.9 mg, 50% yield, >20:1 dr, 94% ee; R_f = 0.4 (PE/EA = 2/1); Light yellow solid, m.p. 68 – 69 °C; $[\alpha]_D^{20}$ = +141 (*c* = 0.1, EA).

^{1}H NMR (300 MHz, $CDCl_3$) δ 8.09 (dd, *J* = 7.7, 1.4 Hz, 1H), 7.53 (td, *J* = 7.4, 1.3 Hz, 1H), 7.42 (t, *J* = 7.4 Hz, 2H), 7.20 (s, 1H), 7.16 – 7.05 (m, 2H), 7.12 – 7.02 (m, 1H), 6.94 (td, *J* = 7.5, 1.5 Hz, 1H), 6.58 (dd, *J* = 7.9, 1.4 Hz, 1H), 4.00 – 3.81 (m, 3H), 3.72 (d, *J* = 14.9 Hz, 1H), 3.44 (t, *J* = 9.0 Hz, 1H), 3.32 (t, *J* = 5.8 Hz, 1H), 3.15 – 2.94 (m, 3H), 2.71 – 2.59 (m, 2H), 2.51 (d, *J* = 11.8 Hz, 1H), 2.41 (s, 3H), 2.40 (s, 3H), 2.36 (s, 3H), 2.29 (dd, *J* = 11.9, 3.8 Hz, 1H).

^{13}C NMR (75 MHz, $CDCl_3$) δ 206.7, 197.4, 168.9, 148.3, 144.3, 142.3, 139.5, 136.1, 134.5, 133.8, 131.8, 130.9, 128.2, 127.9, 127.9, 127.5, 127.3, 126.4, 125.7, 125.0, 119.8, 68.4, 52.2, 51.4, 46.2, 45.9, 45.5, 42.7, 42.5, 29.7, 28.9, 21.3, 20.6.

HRMS (ESI) calcd. for $[C_{33}H_{34}N_2O_3S + H]^+$ 539.2363, found 539.2363.

HPLC: Daicel Chiralcel AD-H, *n*-hexane/isopropanol 80/20, flow rate = 0.5 mL/min, uv-vis λ = 250 nm, t_{R1} = 48.7 min (major), t_{R2} = 58.1 min (minor).

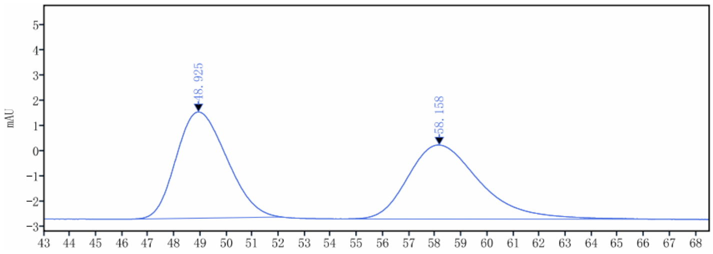

RetTime [min]	Type	Width [min]	Area [mAU*s]	Height [mAU]	Area %
48.925	MM m	1.5729	566.54839	4.21565	50.1296
58.158	MM m	2.2475	563.62011	2.93753	49.8704

Supplementary Figure 95. HPLC Chromatograph of racemic **7aq**

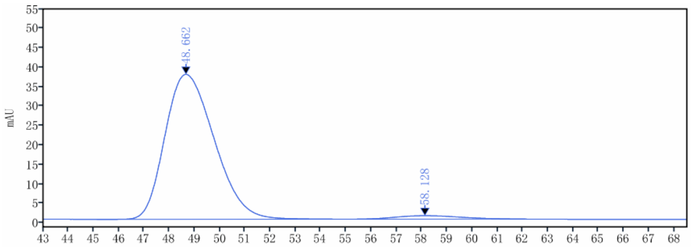

RetTime [min]	Type	Width [min]	Area [mAU*s]	Height [mAU]	Area %
48.662	MM m	2.0634	5042.65956	37.20260	96.9571
58.128	MM m	2.0896	158.25639	0.88526	3.0429

Supplementary Figure 96. HPLC Chromatograph of chiral **7aq**

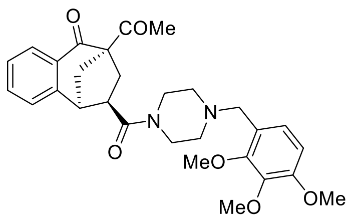

(5S,6R,8R)-8-acetyl-6-(4-(2,3,4-trimethoxybenzyl)piperazine-1-carbonyl)-5,6,7,8-tetrahydro-9H-5,8-methanobenzo[7]annulen-9-one (7ar)

27.9 mg, 55% yield, >20:1 dr, 88% ee; R_f = 0.2 (DCM/EtOH = 40/1); White solid, m.p. 164 – 165 °C; $[\alpha]_D^{20}$ = +135 (*c* = 0.1, EA).

^{1}H NMR (400 MHz, $CDCl_3$) δ 8.03 (d, *J* = 7.4 Hz, 1H), 7.40 (dt, *J* = 32.9, 6.9 Hz, 2H), 7.02 (dd, *J* = 21.6, 7.9 Hz, 2H), 6.67 (d, *J* = 8.6 Hz, 1H), 3.92 – 3.87 (m, 9H), 3.70 (d, *J* = 4.9 Hz, 2H), 3.64 – 3.49 (m, 5H), 3.36 – 3.34 (m, 1H), 2.67 – 2.52 (m, 4H), 2.46 – 2.36 (m, 6H), 2.23 (dd, *J* = 11.9, 3.9 Hz, 1H).

^{13}C NMR (75 MHz, $CDCl_3$) δ 206.6, 197.4, 168.6, 153.2, 152.6, 144.3, 142.3, 133.7, 130.9, 128.1, 127.8, 127.3, 125.2, 123.2, 107.0, 68.3, 61.3, 60.8, 56.4, 56.0, 53.2, 52.5, 45.7, 45.5, 42.7, 42.0, 29.7, 28.8.

HRMS (ESI) calcd. for $[C_{29}H_{34}N_2O_6+H]^+$ 507.2490, found 507.2484 .

HPLC: Daicel Chiralcel IA-3, *n*-hexane/isopropanol 70/30, flow rate = 0.5 mL/min, uv-vis λ = 254 nm, t_{R1} = 23.0 min (minor), t_{R2} = 27.2 min (major).

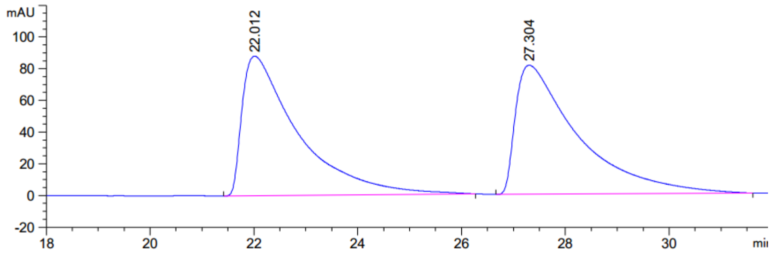

Peak #	RetTime [min]	Type	Width [min]	Area [mAU*s]	Height [mAU]	Area %
1	22.012	BB	1.0486	6627.34180	88.19863	49.2820
2	27.304	BB	1.1606	6820.44824	81.48243	50.7180

Supplementary Figure 97. HPLC Chromatograph of racemic **7ar**

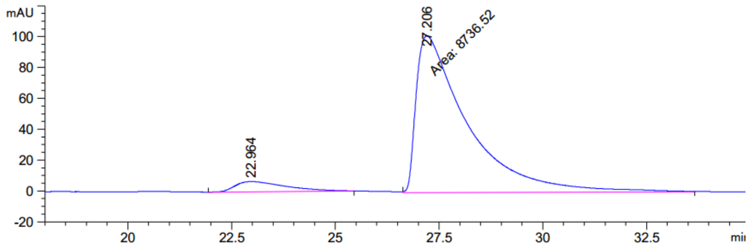

Peak #	RetTime [min]	Type	Width [min]	Area [mAU*s]	Height [mAU]	Area %
1	22.964	BB	1.0011	561.47656	6.60946	6.0387
2	27.206	MM	1.4356	8736.52441	101.42917	93.9613

Supplementary Figure 98. HPLC Chromatograph of chiral **7ar**

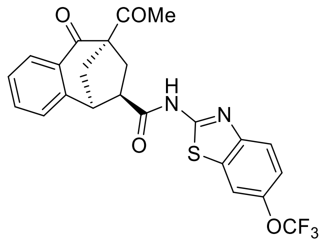

(5S,6R,8R)-8-acetyl-9-oxo-N-(6-(trifluoromethoxy)benzo[d]thiazol-2-yl)-6,7,8,9-tetrahydro-5H-5,8-methanobenzo[7]annulene-6-carboxamide (7as)

33.6 mg, 71% yield, >20:1 dr, 95% ee; R_f = 0.3 (PE/EA = 1/2); White solid, m.p. 178 – 180 ºC; $[\alpha]^{20}_{D}$ = +281 (*c* = 0.1, EA).

^{1}H NMR (300 MHz, $CDCl_3$) δ 10.42 (brs, 1H), 8.11 – 8.08 (m, 1H), 7.73 (d, *J* = 8.8 Hz, 1H), 7.62 (d, *J* = 1.3 Hz, 1H), 7.40 – 7.37 (m, 2H), 7.30 (dd, *J* = 8.8, 1.5 Hz, 1H), 7.13 – 7.10 (m, 1H), 3.81 (t, *J* = 4.6 Hz, 1H), 3.65 (dt, *J* = 11.5, 6.2 Hz, 1H), 2.83 (dd, *J* = 14.8, 10.2 Hz, 1H), 2.57 (d, *J* = 11.7 Hz, 1H), 2.46 (dd, *J* = 14.8, 6.4 Hz, 1H), 2.38 (s, 3H), 2.24 (dd, *J* = 11.8, 4.1 Hz, 1H).

^{13}C NMR (75 MHz, $CDCl_3$) δ 206.3, 197.1, 169.5, 159.0, 146.8, 145.5, 143.6, 134.4, 133.0, 130.3, 128.6, 128.2, 127.5, 121.4, 120.3, 114.4, 68.7, 49.7, 46.6, 42.7, 28.7, 28.3.

^{19}F NMR (282 MHz, $CDCl_3$) δ -73.4.

HRMS (ESI) calcd. for $[C_{23}H_{17}F_3N_2O_4S+H]^+$ 475.0934, found 475.0928.

HPLC: Daicel Chiralcel IA-3, *n*-hexane/isopropanol 70/30, flow rate = 0.5 mL/min, uv-vis λ = 254 nm, t_{R1} = 15.6 min (minor), t_{R2} = 18.4 min (major).

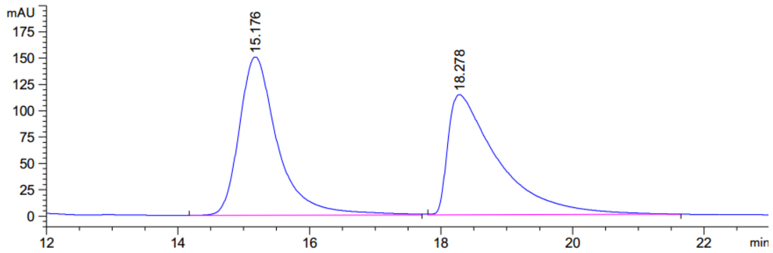

Peak #	RetTime [min]	Type	Width [min]	Area [mAU*s]	Height [mAU]	Area %
1	15.176	VB	0.6087	6120.15332	150.40971	50.2328
2	18.278	BB	0.7351	6063.41748	114.25931	49.7672

Supplementary Figure 99. HPLC Chromatograph of racemic **7as**

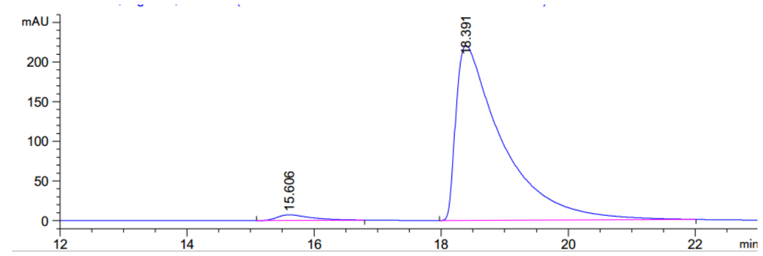

Peak #	RetTime [min]	Type	Width [min]	Area [mAU*s]	Height [mAU]	Area %
1	15.606	BB	0.5370	270.09885	7.17827	2.3484
2	18.391	BB	0.7107	1.12315e4	220.32130	97.6516

Supplementary Figure 100. HPLC Chromatograph of chiral **7as**

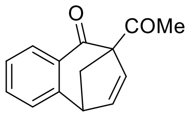

8-acetyl-5,8-dihydro-9H-5,8-methanobenzo[7]annulen-9-one (3a')

1.7 mg, 8% yield, 61% ee; R_f = 0.3 (PE/EA = 20/1); White soild, m.p. 91 – 92 ºC; $[\alpha]_D^{20}$ = -58 (c = 0.1, EA).

^{1}H NMR (300 MHz, $CDCl_3$) δ 7.94 (dd, J = 7.5, 1.6 Hz, 1H), 7.41 (td, J = 7.4, 1.6 Hz, 1H), 7.33 (td, J = 7.5, 1.4 Hz, 1H), 7.17 (dd, J = 7.3, 1.4 Hz, 1H), 6.78 (dd, J = 5.4, 3.1 Hz, 1H), 6.32 (d, J = 5.4 Hz, 1H), 3.81 (dd, J = 4.9, 3.1 Hz, 1H), 2.94 (dd, J = 10.5, 0.9 Hz, 1H), 2.77 (dd, J = 10.6, 4.9 Hz, 1H), 2.35 (s, 3H).

^{13}C NMR (75 MHz, $CDCl_3$) δ 205.4, 193.0, 147.5, 145.4, 133.2, 130.7, 128.9, 128.3, 127.7, 125.0, 74.9, 50.6, 47.0, 28.5.

HRMS (ESI) calcd. for $[C_{14}H_{12}O_2+Na]^+$ 235.0730, found 235.0730.

HPLC: Daicel Chiralcel OD-H, n-hexane/isopropanol 95/5, flow rate = 0.5 mL/min, uv-vis λ = 250 nm, t_{R1} = 19.6 min (minor), t_{R2} = 21.1 min (major).

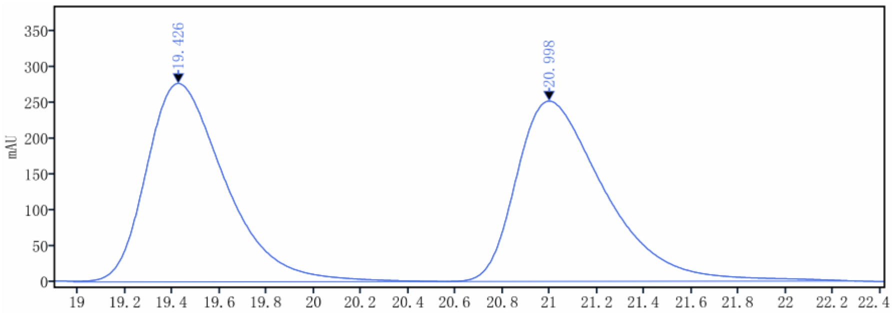

RetTime [min]	Type	Width [min]	Area [mAU*s]	Height [mAU]	Area %
19.426	MM m	0.3552	6441.00460	277.08550	49.5421
20.998	MM m	0.3960	6560.07440	251.79093	50.4579

Supplementary Figure 101. HPLC Chromatograph of racemic **3a'**

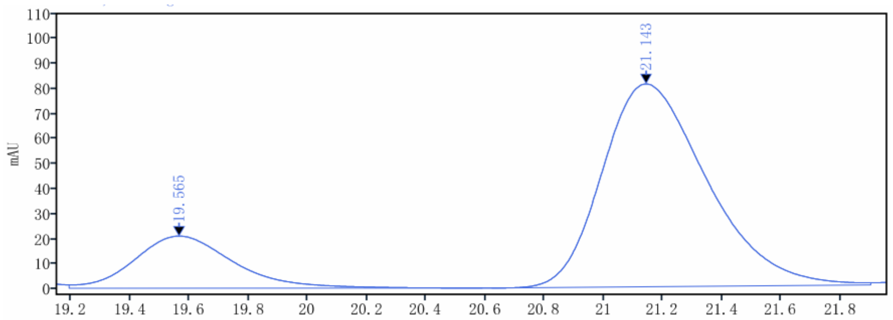

RetTime [min]	Type	Width [min]	Area [mAU*s]	Height [mAU]	Area %
19.565	MB m	0.3536	481.66930	20.76811	19.5004
21.143	BM m	0.3787	1988.38251	80.91776	80.4996

Supplementary Figure 102. HPLC Chromatograph of chiral **3a'**

Enantiodivergent synthesis of (5*S*, 6*R*, 8*R*)-5ac and (5*R*, 6*S*, 8*S*)-5ac’

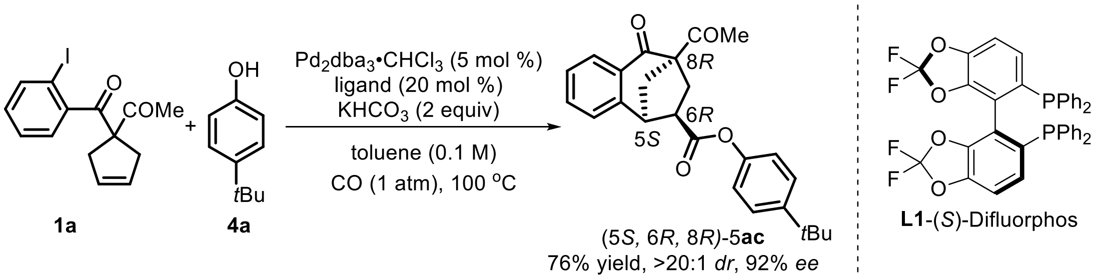

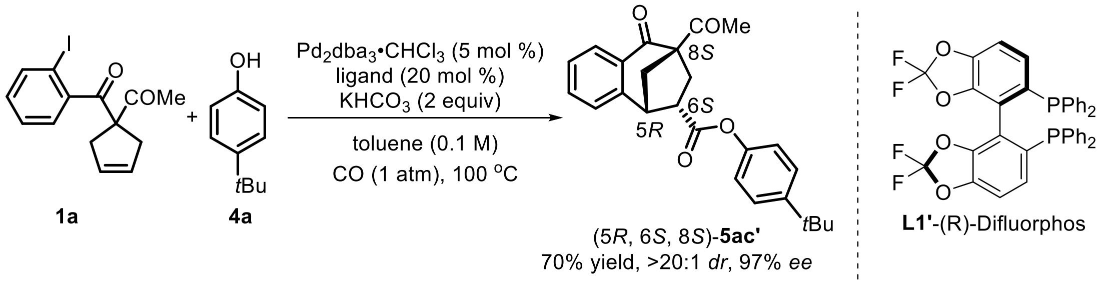

Enantiodivergent synthesis of (5*S*, 6*R*, 8*R*)-**5ac** and (5*R*, 6*S*, 8*S*)-**5ac’**could be realized through the employment of ligand with different configurations from the same substrates. When (*S*)-Difluorphos (**L1**) was employed in the reaction, (5*S*, 6*R*, 8*R*)-**5ac** could be achieved in 76% yield with 92% *ee*; when (*R*)-Difluorphos (**L1’**) was employed, (5*R*, 6*S*, 8*S*)-**5ac'** could be obtained in 70% yield and 97% *ee*.

The HPLC results of **5ac** were depicted in **Supplementary Figure 43** and **Supplementary Figure 44**.

Supplementary Notes

Supplementary Note 1: Details of optimizing the reaction conditions

Supplementary Table 1. Optimization of reaction conditions

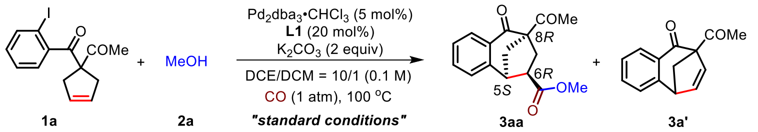

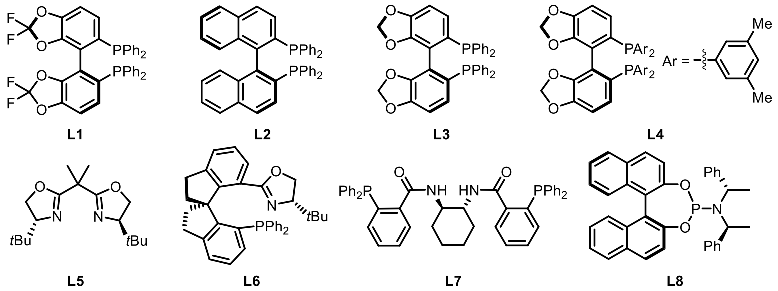

Entry	Deviation of standard conditions	Yield of 3aa (%)[b]	*dr* of 3aa[c]	*ee* of 3aa (%)[d]	Yield of 3a' (%)[b]	*ee* of 3a' (%)[d]
1	none	81	>20:1	96	<2	-
2	$Pd(OAc)_2$ instead of $Pd_2dba_3{\cdot}CHCl_3$	71	>20:1	66	6	21
3	Pd_2dba_3 instead of $Pd_2dba_3{\cdot}CHCl_3$	31	>20:1	85	7	14
4	**L2** instead of **L1**	82	>20:1	52	<2	-
5	**L3** instead of **L1**	75	15:1	79	9	8
6	**L4** instead of **L1**	60	>20:1	80	<2	-
7	**L5** instead of **L1**	59	>20:1	-5	<2	-
8	**L6** instead of **L1**	21	>20:1	5	<2	-
9	**L7** instead of **L1**	<2	-	-	13	13
10	**L8** instead of **L1**	<2	-	-	21	31
11	10 mol% of **L1** instead of 20 mol% of **L1**	63	11:1	36	24	0
12	2.5 mol% $Pd_2dba_3{\cdot}CHCl_3$, 10 mol% of **L1** instead of 5 mol% $Pd_2dba_3{\cdot}CHCl_3$, 20 mol% of **L1**	55	10:1	35	16	0
13	$KHCO_3$ instead of K_2CO_3	57	4:1	80	<2	-
14	Na_2CO_3 instead of K_2CO_3	32	7:1	53	<2	-
15	AgOAc instead of K_2CO_3	<2	-	-	83	0
16	DCE instead of DCE/DCM	78	4:1	80	<2	-
17	DCM instead of DCE/DCM	75	>20:1	88	<2	-
18	toluene instead of DCE/DCM	69	5:1	53	<2	-
19	CH_3CN instead of DCE/DCM	90	8:1	77	<2	-
20	DCE/DCM = 5/1 instead of 10/1	65	>20:1	92	<2	-
21	DCE/DCM = 1/1 instead of 10/1	71	10:1	96	8	61
22	DCE/DCM = 1/10 instead of 10/1	38	12:1	93	38	42
23	80 °C instead of 100 °C	71	>20:1	77	<2	-
24	120 °C instead of 100 °C	38	13:1	94	<2	-

[a] Reaction conditions: **1a** (0.1 mmol), **2a** (1 mmol), [Pd] (10 mol%), ligand (20 mol%), base (0.2 mmol) in 1 mL solvent, 100 °C, 36 h, under 1 atm CO [b] Isolated yield [c] Determined by ^{1}H NMR analysis [d] Determined by HPLC analysis on a chiral stationary phase

Supplementary Note 2: Details of Nonlinear Effect (NLE) Experiments

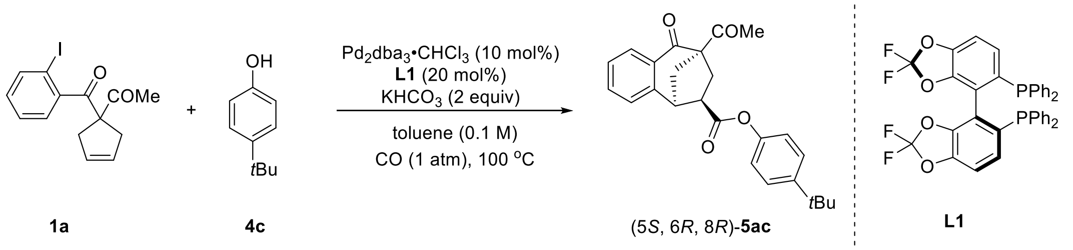

A sealed tube was charged with the substrate **1a** (0.1 mmol, 1 equiv), **4c** (2.5 equiv), Pd_2dba_3•$CHCl_3$ (5 mol%), ligand **L1** (20 mol%, mixtures of two enantiomers determined by weight), and $KHCO_3$ (2 equiv). The vial is thoroughly flushed with CO and toluene (0.1 M) was added under CO atmosphere. Then the reaction mixture was stirred at 100 ºC for 36 h. After the reaction vessel was cooled to room temperature, the solution was concentrated in *vacuo* and purified by chromatography on silica gel (200–300 mesh) (PE/EA = 4/1) to afford the desired product. Analysis of the pure samples by chiral-HPLC provided the reported enantiomeric excesses.

The results were listed as follows in **Supplementary Table 2** and **Supplementary Figure 103**.

Supplementary Table 2. Nonlinear Effect Results

Entry	Ligand *ee* (%)	Product *ee* (%)
1	0.0	0.0
2	17.6	12.9
3	41.0	33.2
4	60.6	48.7
5	79.7	75.1
6	>99.5	91.5

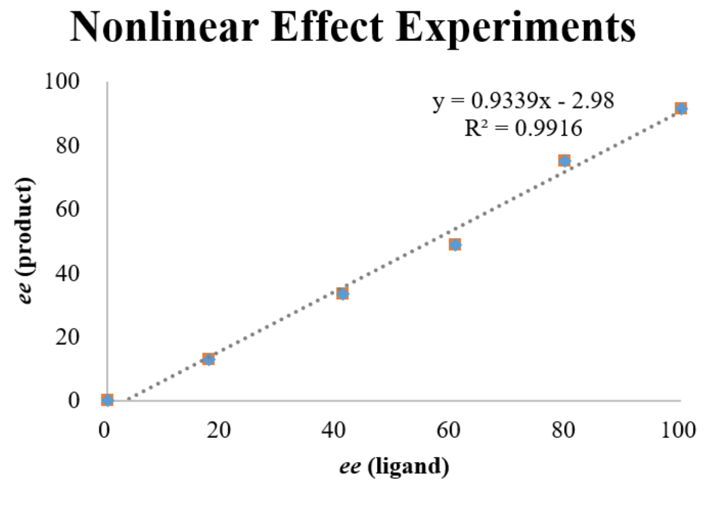

Supplementary Figure 103. Nonlinear Effect Results

Supplementary Note 3: Crystal Structure of 3aa

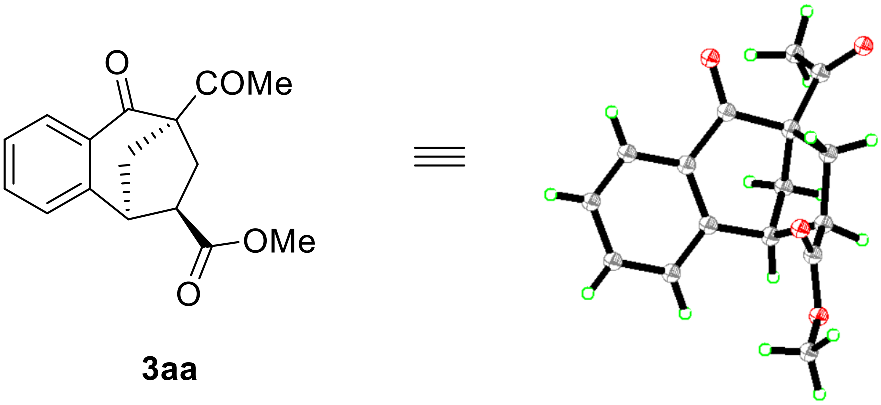

Supplementary Figure 104. ORTEP plot of the crystal structure of **3aa**

Supplementary Table 3. X-ray crystallographic data of **3aa**

CCDC number	1936197
Empirical formula	$C_{16}H_{16}O_4$
Formula weight	272.29
Temperature	297 K
Wavelength	1.54178 Å
Space group	$P2_12_12_1$
Unit cell dimensions	a=7.1298(1) Å =90º
	b=13.3353(3) Å =90º
	c=14.7054(3) Å =90º
Volume	1398.16(5) $Å^3$
Z	4
F(000)	576.0
Completeness to theta = 74.600°	1.72/1.00
Max. and min. transmission	0.754 and 0.471
R indices (all data)	R= 0.0344(2639)<br>wR2(reflections)= 0.0896(2868)
S	1.060

Supplementary Note 4: Crystal Structure of 7aa

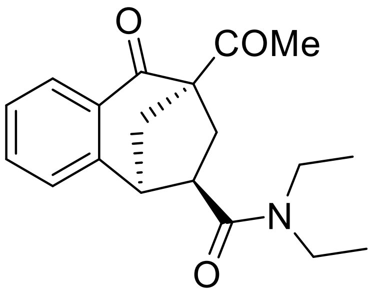

7aa

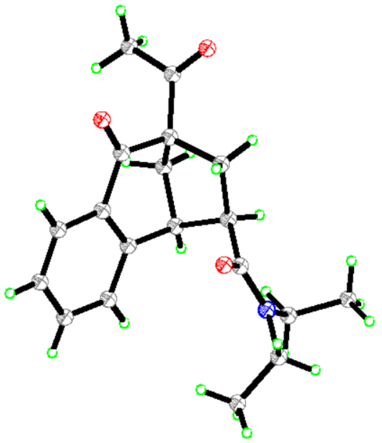

Supplementary Figure 105. ORTEP plot of the crystal structure of **7aa**

Supplementary Table 4. X-ray crystallographic data of **7aa**

CCDC number	1936198
Empirical formula	$C_{19}H_{23}NO_3$
Formula weight	313.38
Temperature	297 K
Wavelength	1.54178 Å
Space group	$P2_12_12_1$
Unit cell dimensions	a=5.7743(3) Å =90º
	b=15.3088(7) Å =90º
	c=19.9259(10) Å =90º
Volume	1761.40(15) Å^3
Z	4
F(000)	672.0
Completeness to theta = 74.630°	1.71/1.00
Max. and min. transmission	0.960 and 0.800
R indices (all data)	R= 0.0501(2798)<br>wR2(reflections)= 0.1479(3584)
S	1.037

Supplementary Note 5: NMR Spectra of all characterized compounds

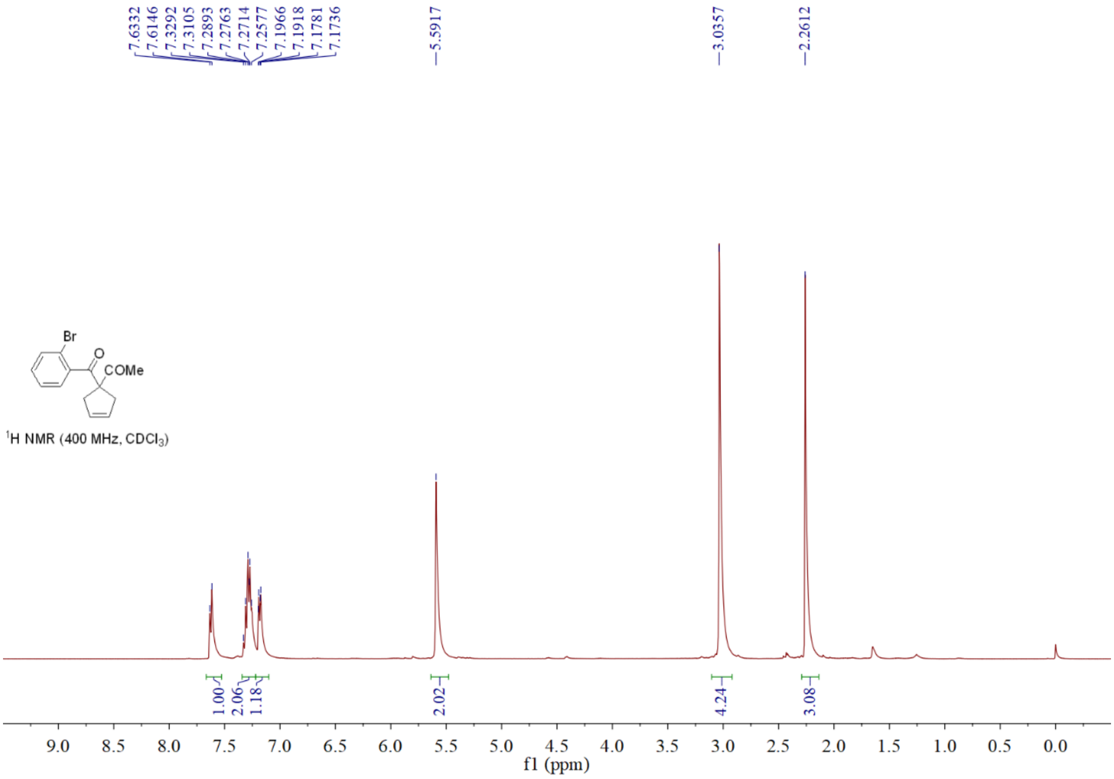

Supplementary Figure 106. ^{1}H NMR of **1a'**

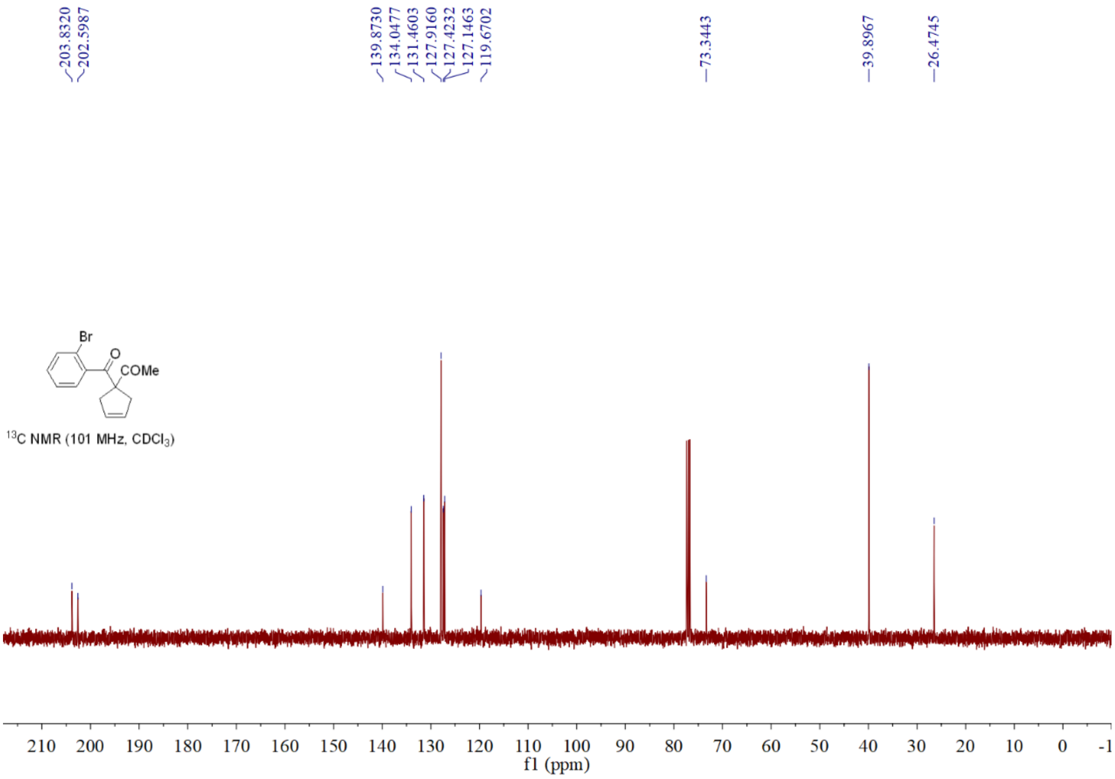

Supplementary Figure 107. ^{13}C NMR of **1a'**

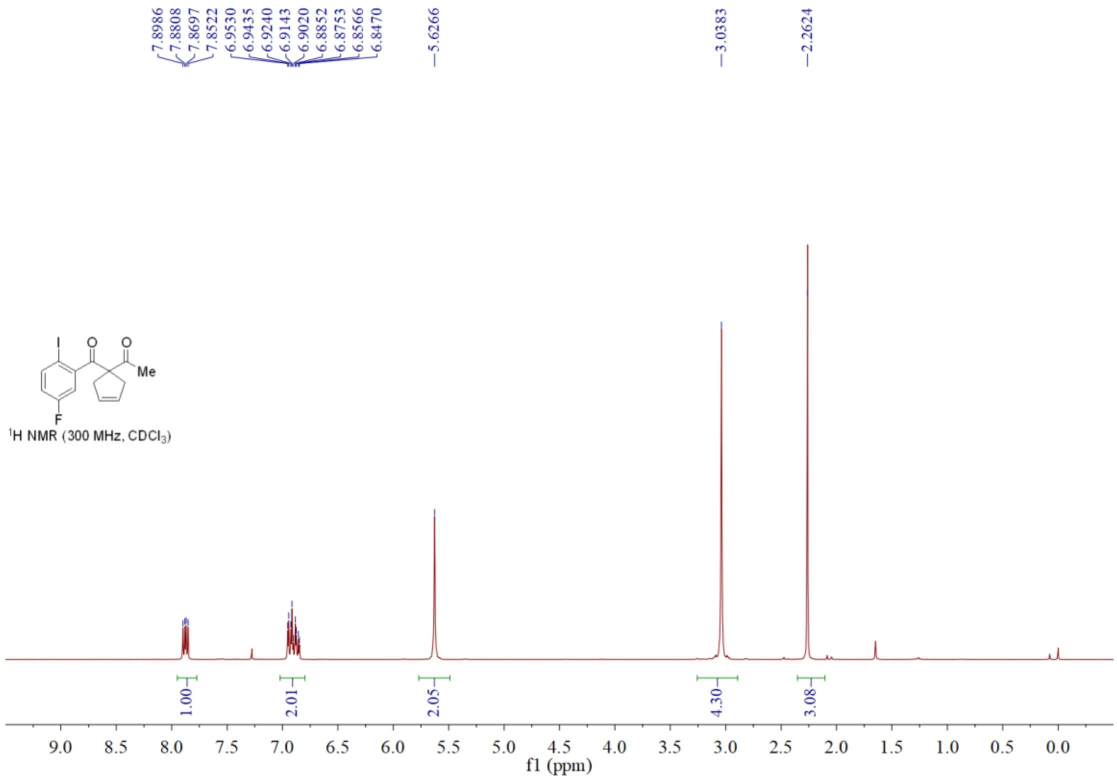

Supplementary Figure 108. ^{1}H NMR of **1b**

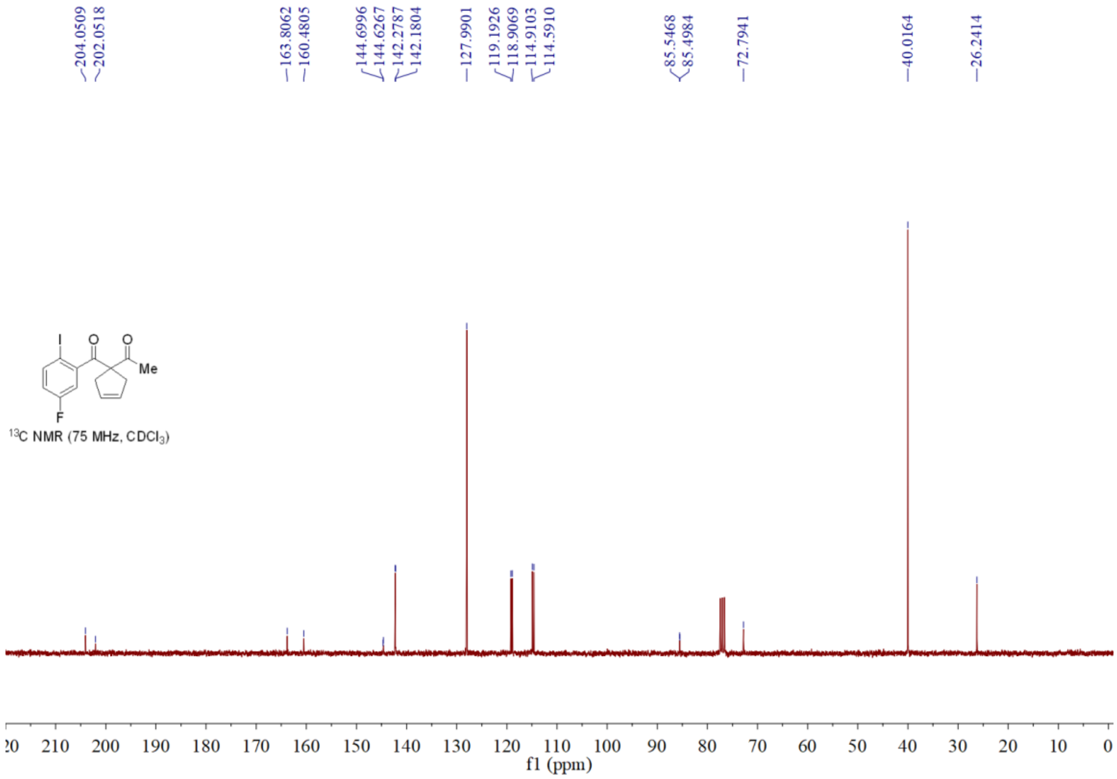

Supplementary Figure 109. ^{13}C NMR of **1b**

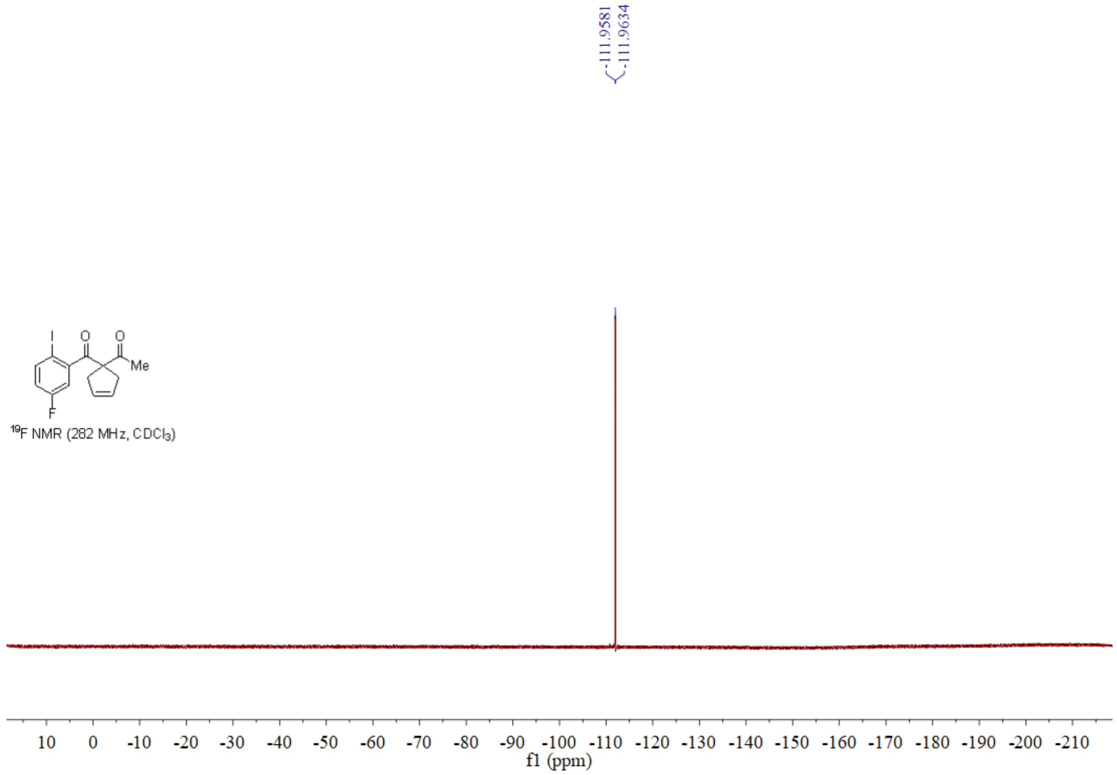

Supplementary Figure 110. ^{19}F NMR of **1b**

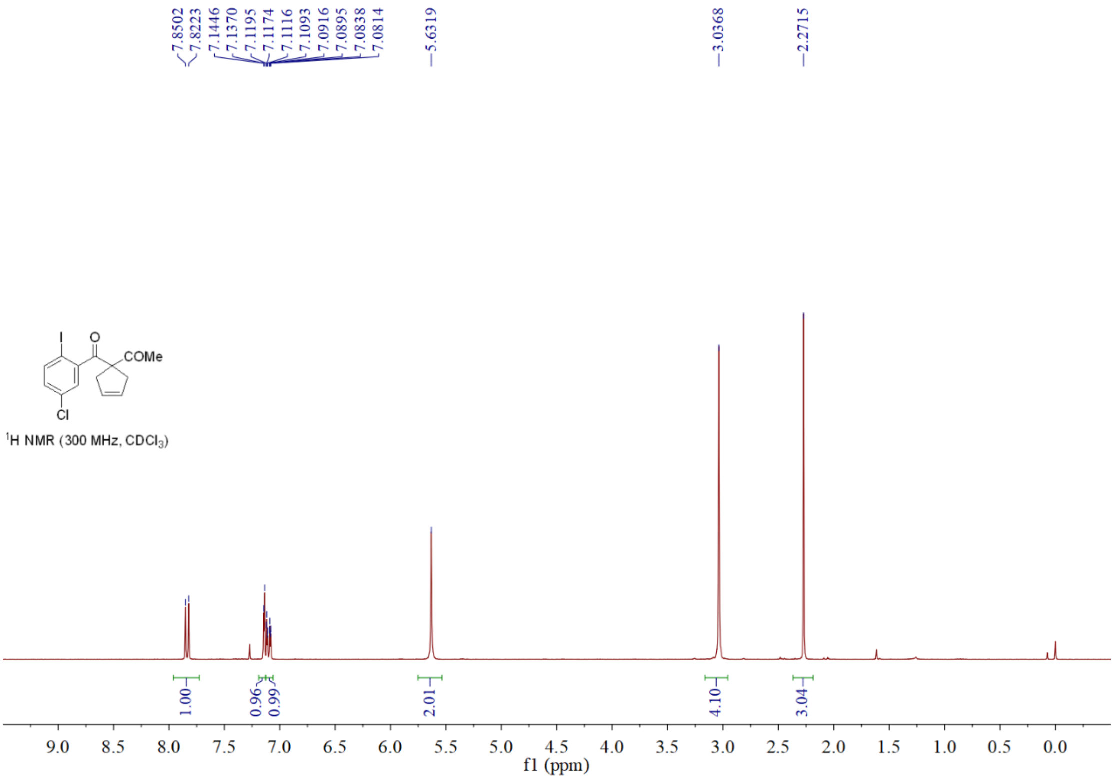

Supplementary Figure 111. ^{1}H NMR of **1c**

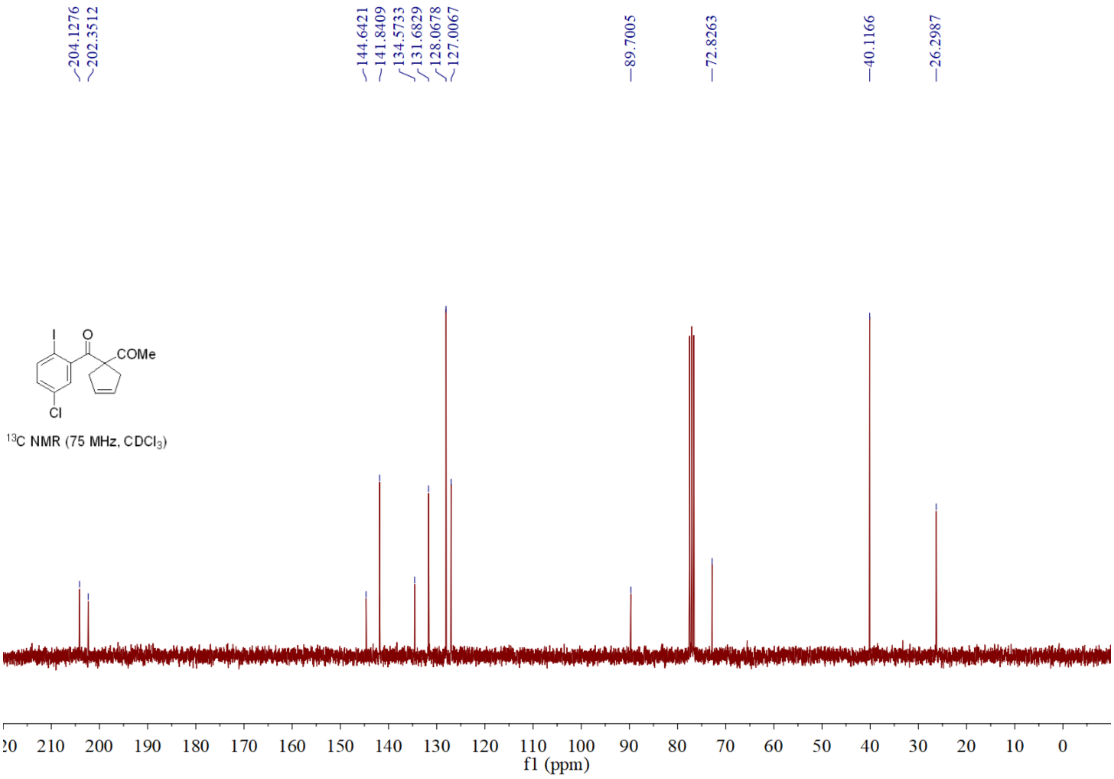

Supplementary Figure 112. ^{13}C NMR of **1c**

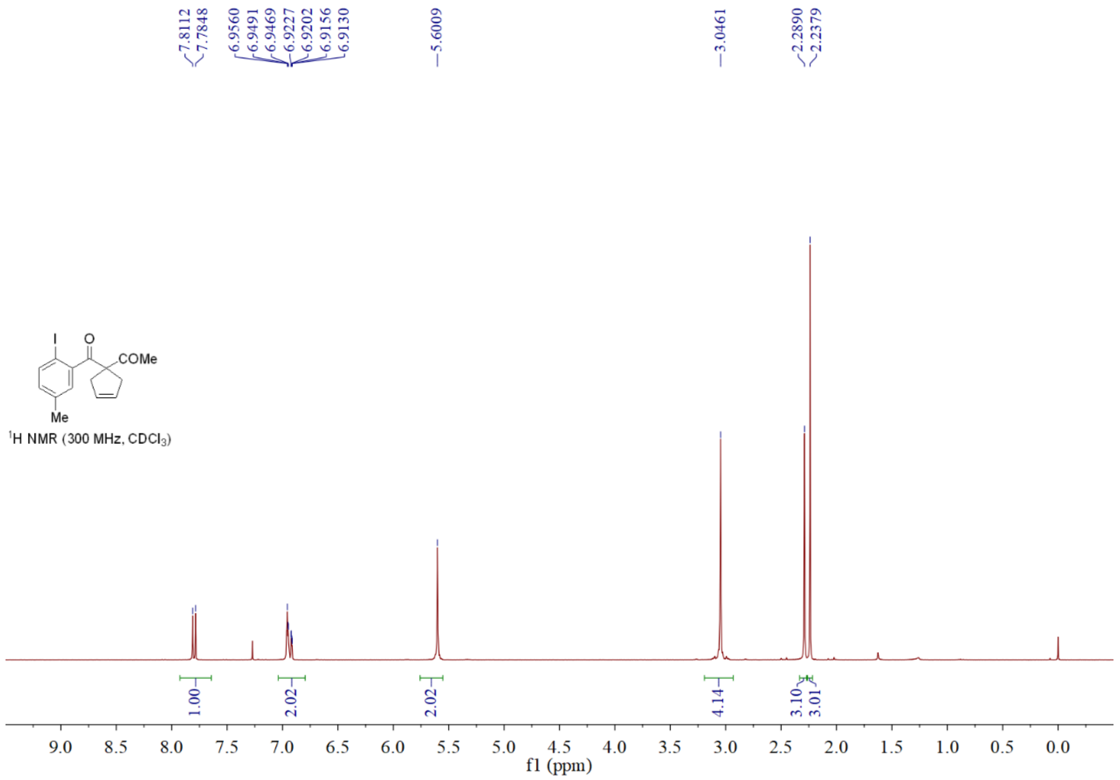

Supplementary Figure 113. ^{1}H NMR of **1d**

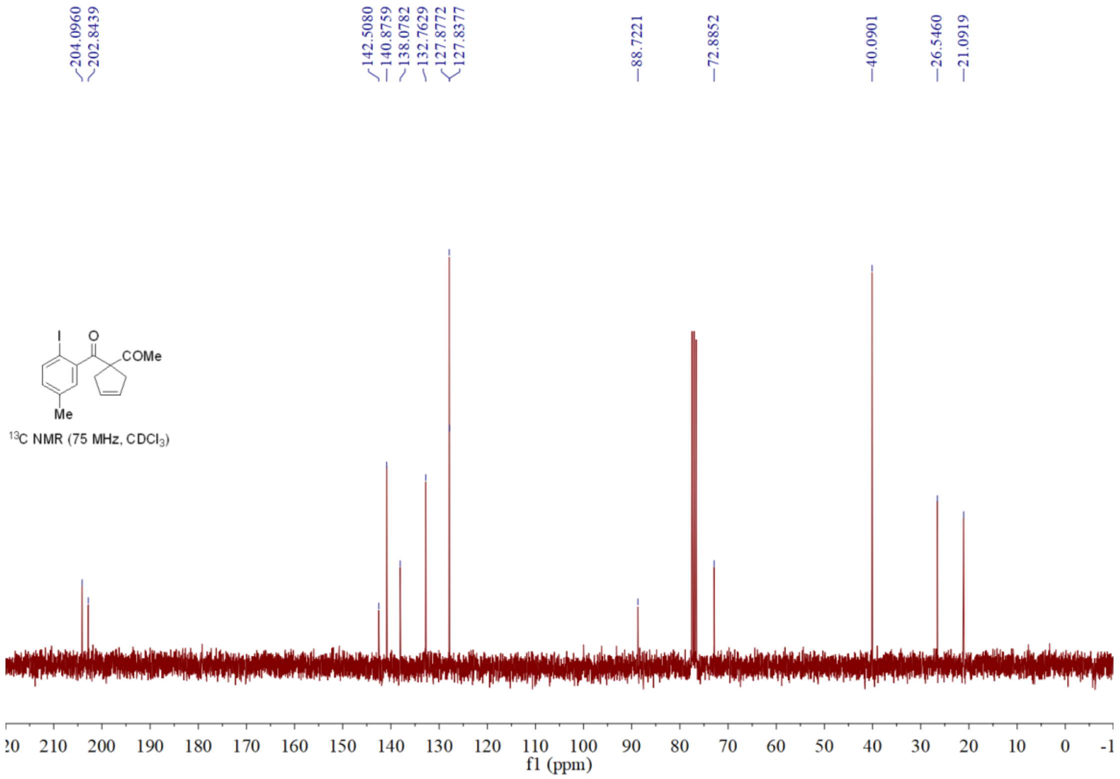

Supplementary Figure 114. ^{13}C NMR of **1d**

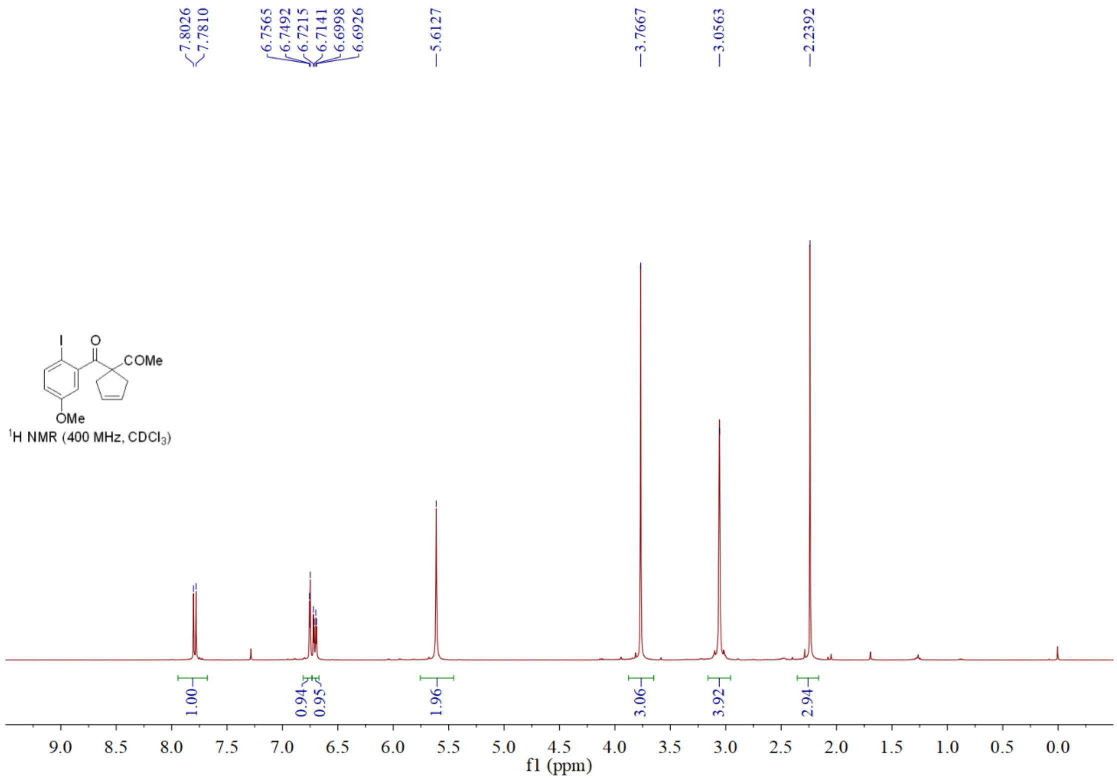

Supplementary Figure 115. ^{1}H NMR of **1e**

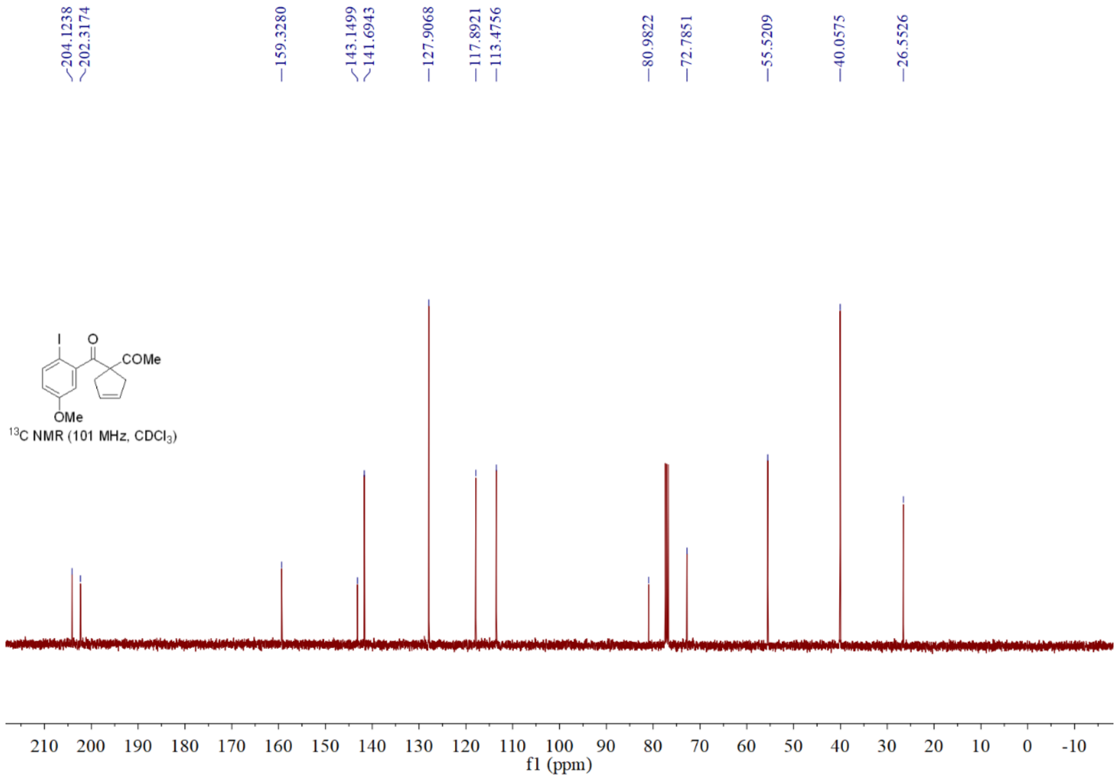

Supplementary Figure 116. ^{13}C NMR of **1e**

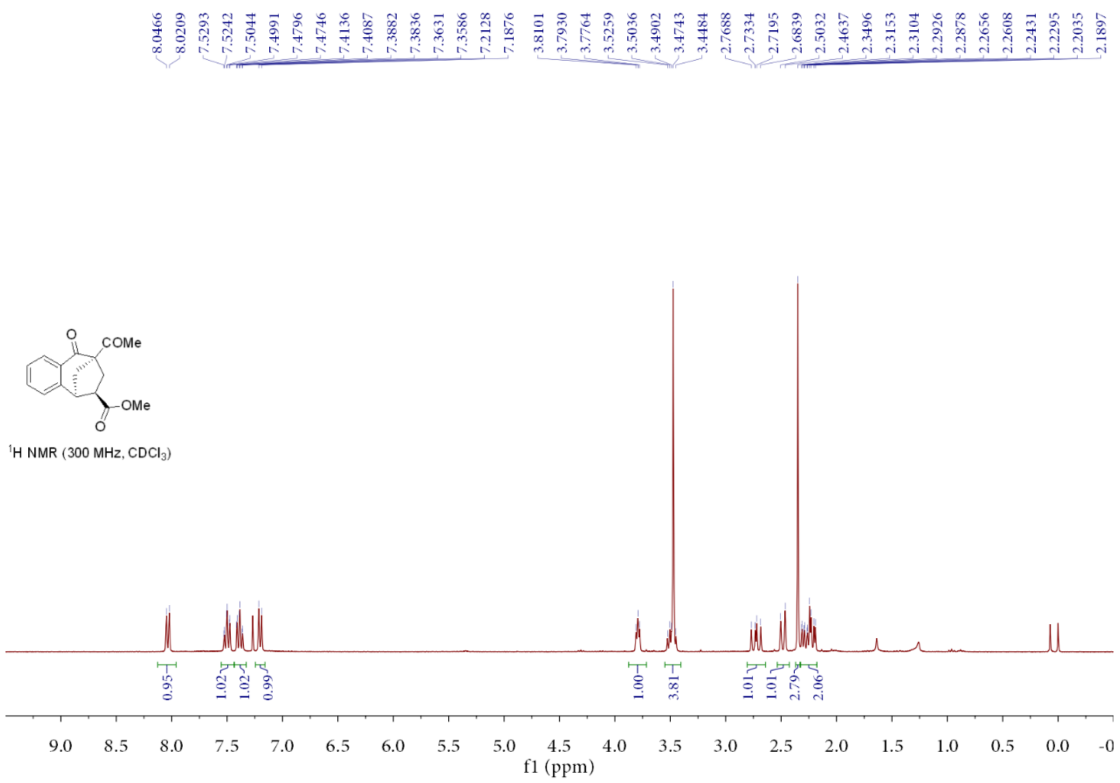

Supplementary Figure 117. ^{1}H NMR of **3aa**

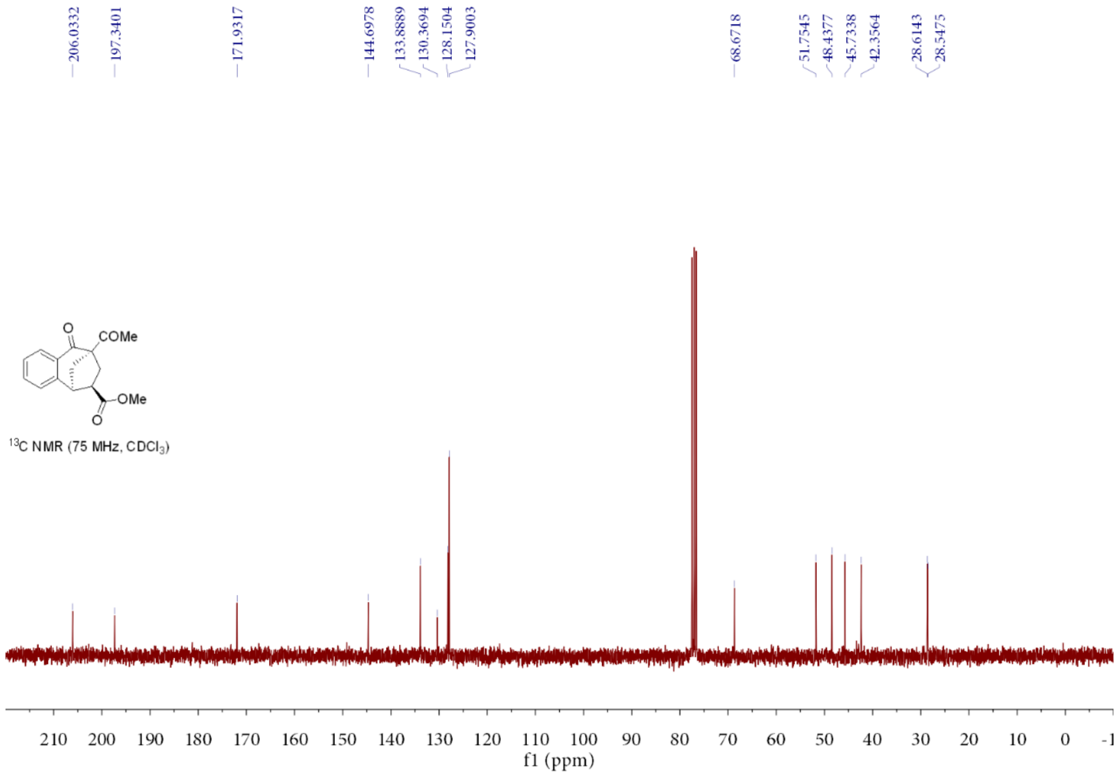

Supplementary Figure 118. ^{13}C NMR of **3aa**

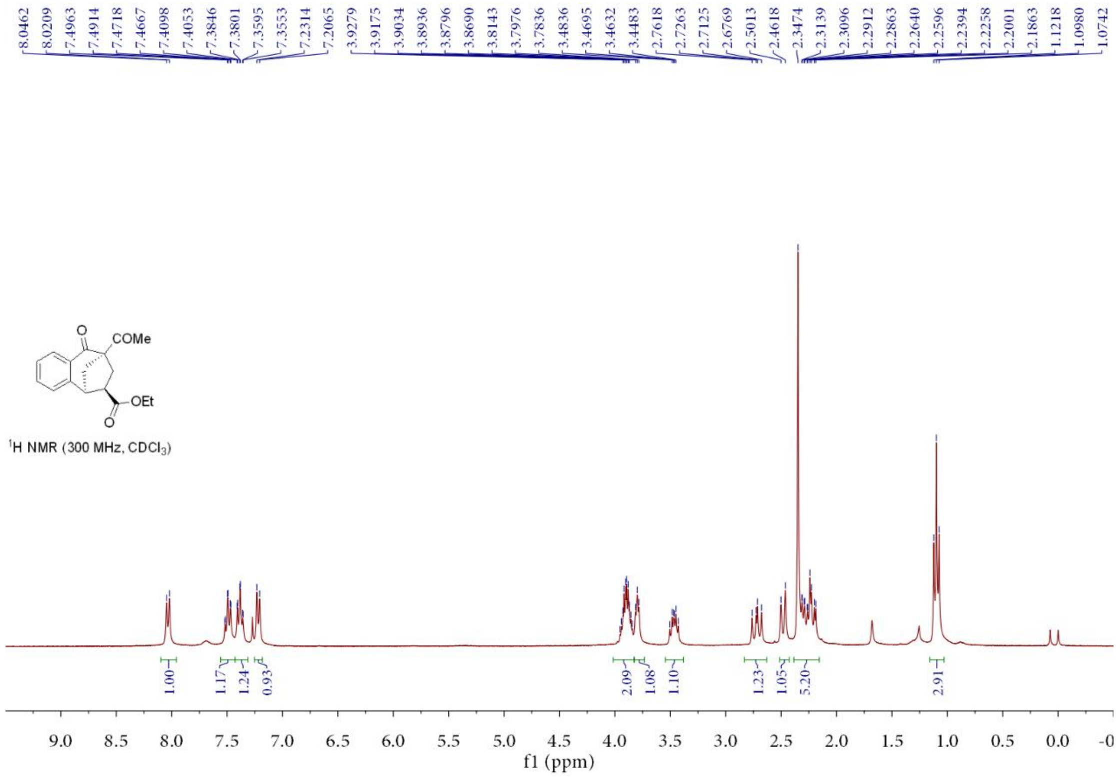

Supplementary Figure 119. ^{1}H NMR of **3ab**

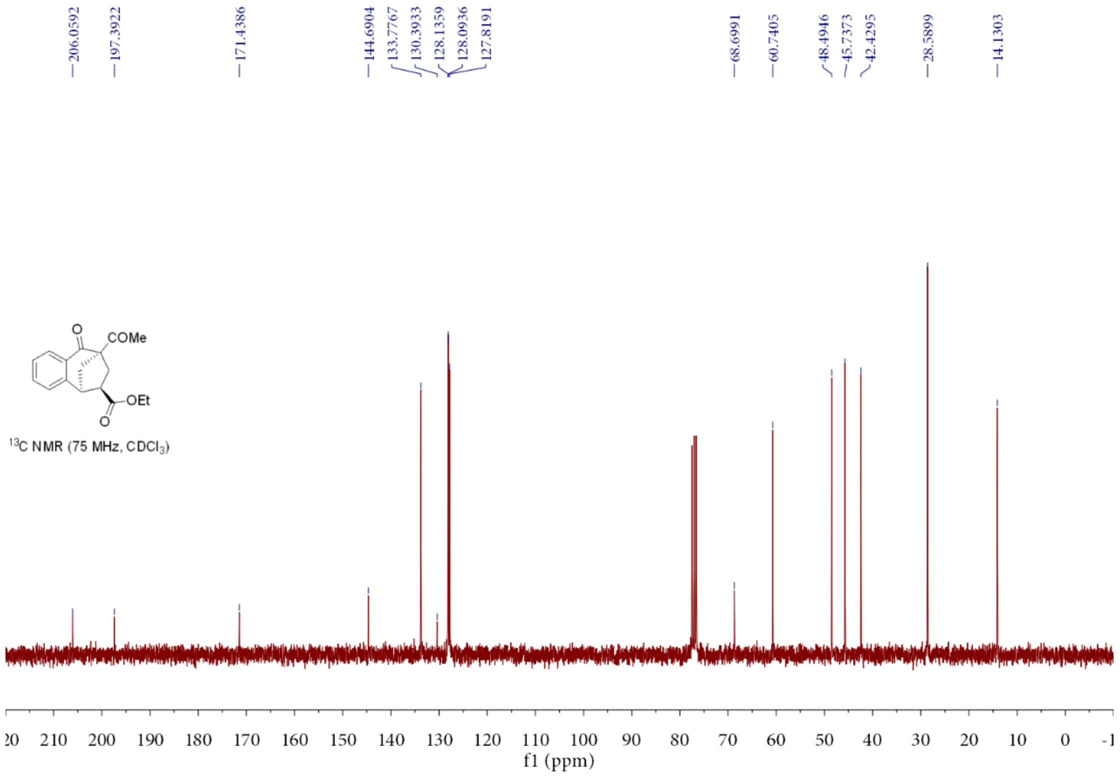

Supplementary Figure 120. ^{13}C NMR of **3ab**

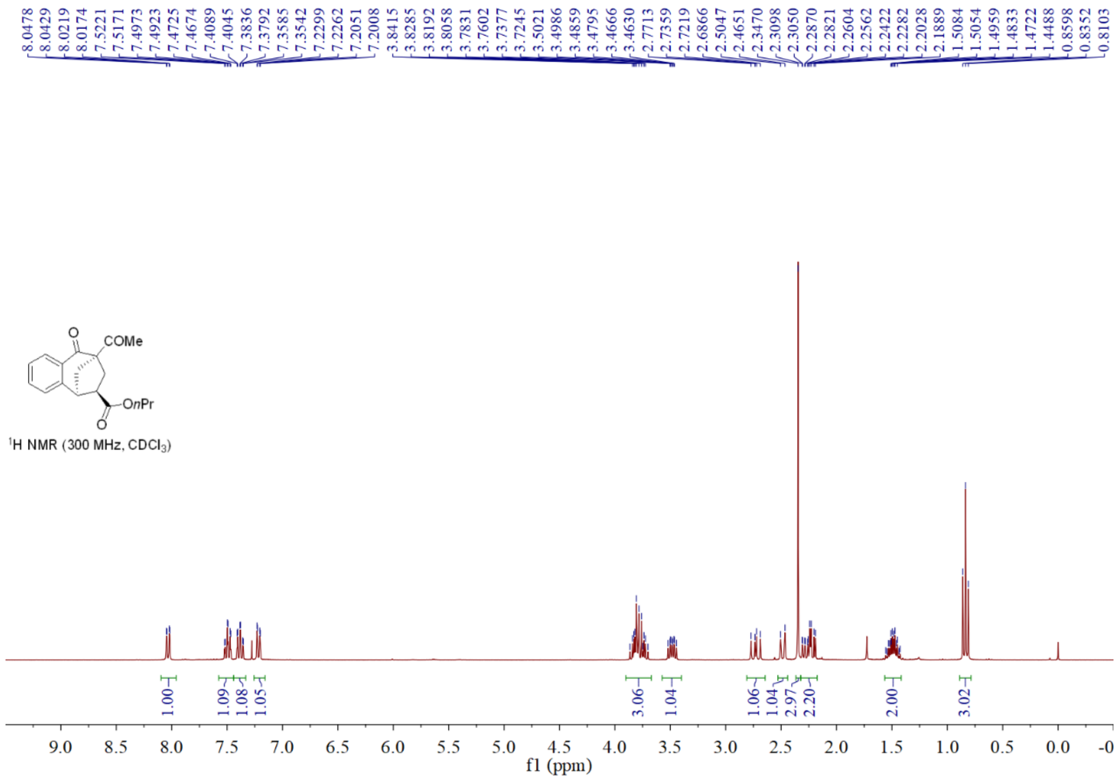

Supplementary Figure 121. ^{1}H NMR of **3ac**

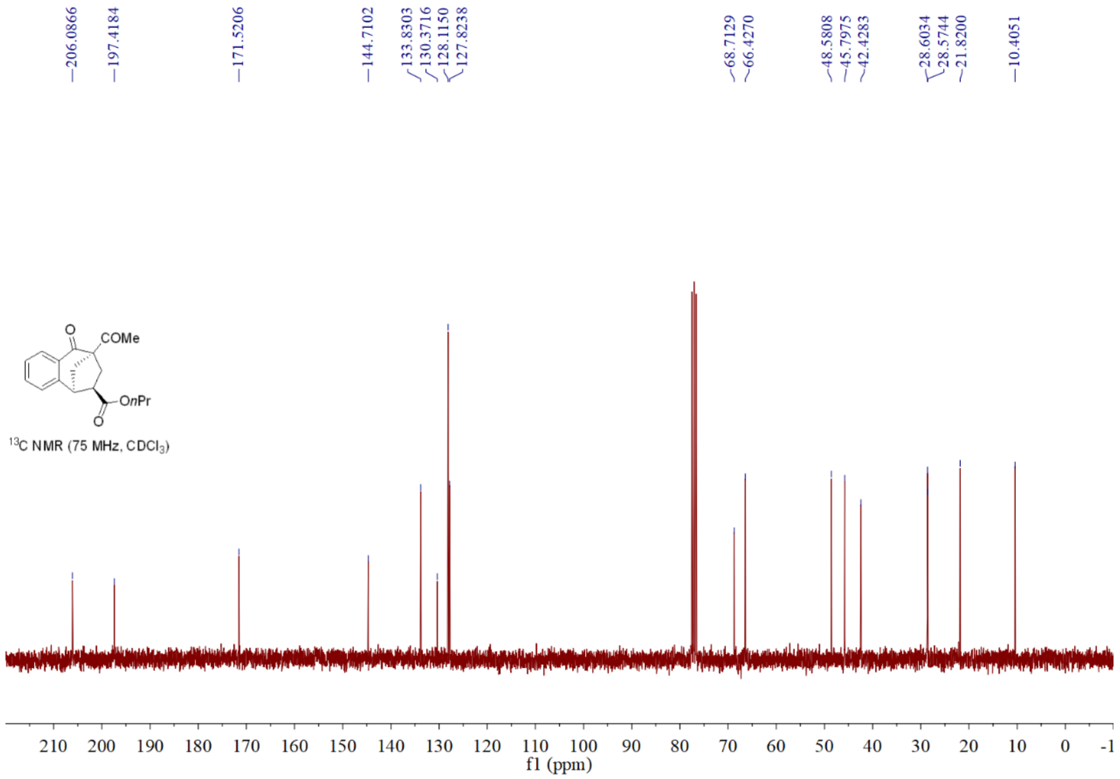

Supplementary Figure 122. ^{13}C NMR of **3ac**

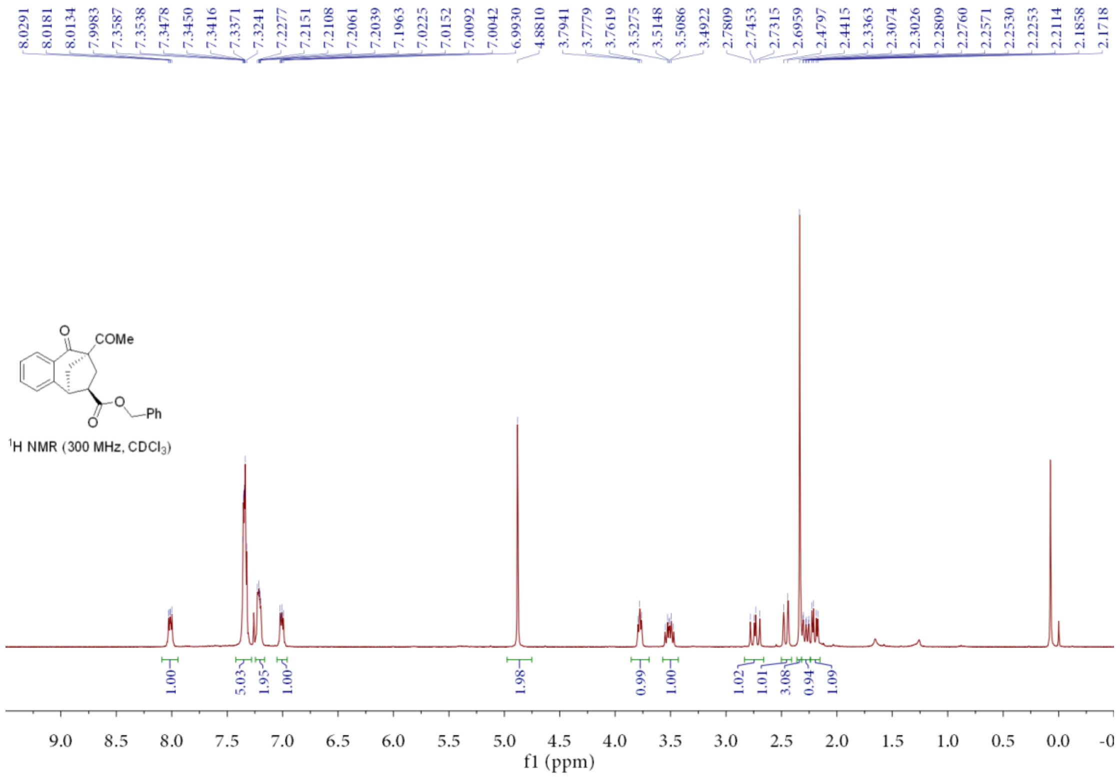

Supplementary Figure 123. ^{1}H NMR of **3ad**

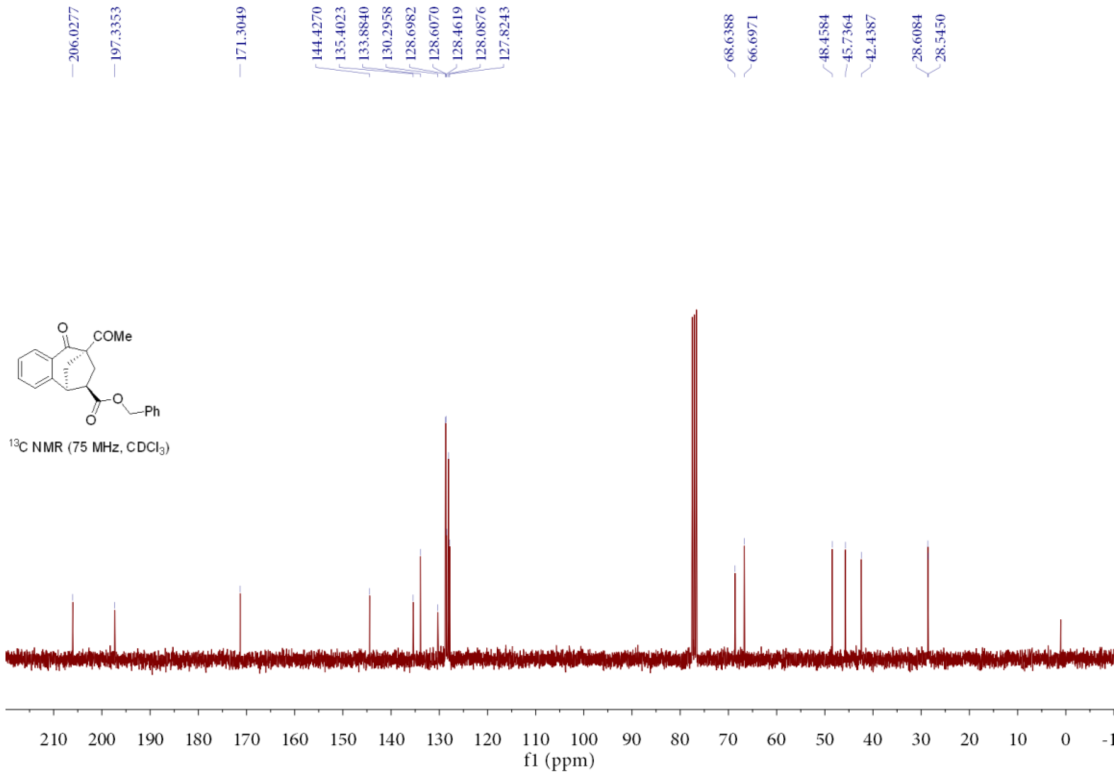

Supplementary Figure 124. ^{13}C NMR of **3ad**

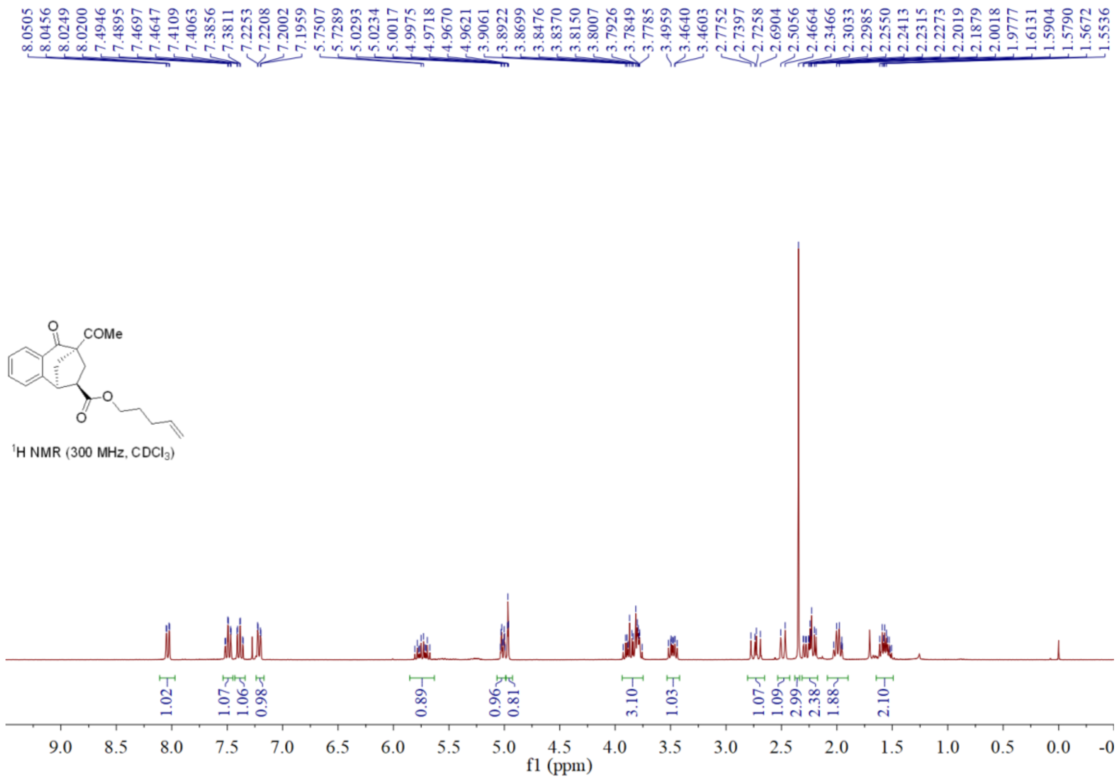

Supplementary Figure 125. ^{1}H NMR of **3ae**

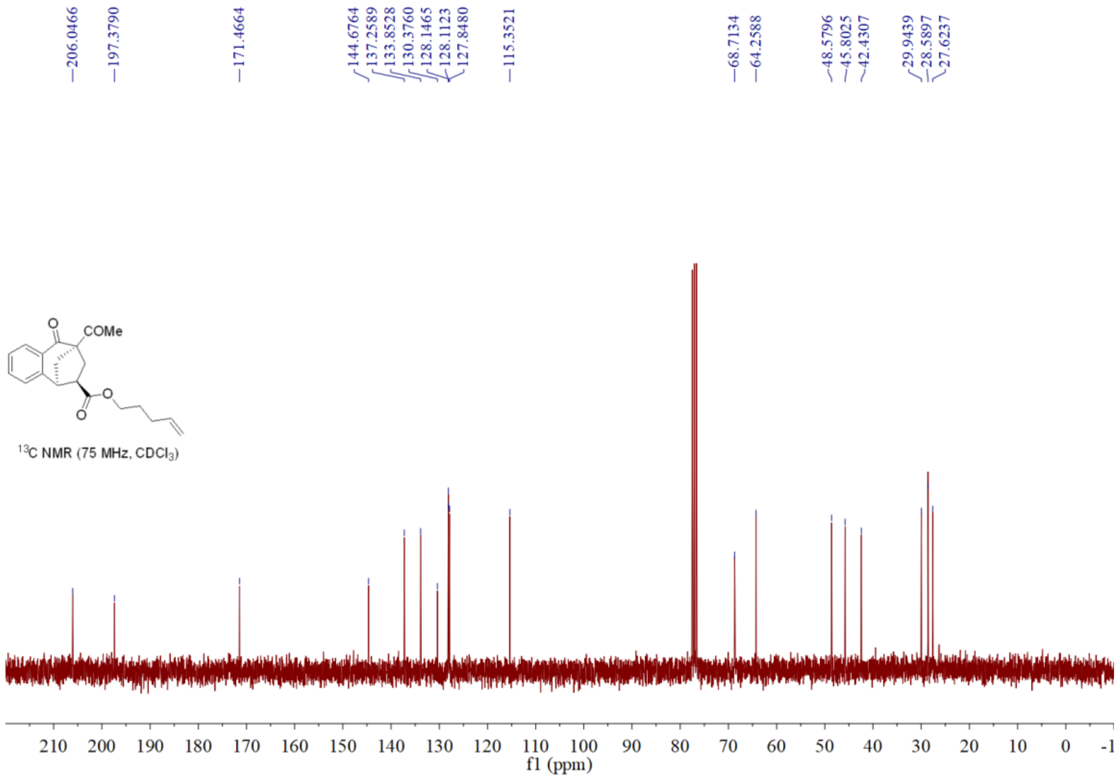

Supplementary Figure 126. ^{13}C NMR of **3ae**

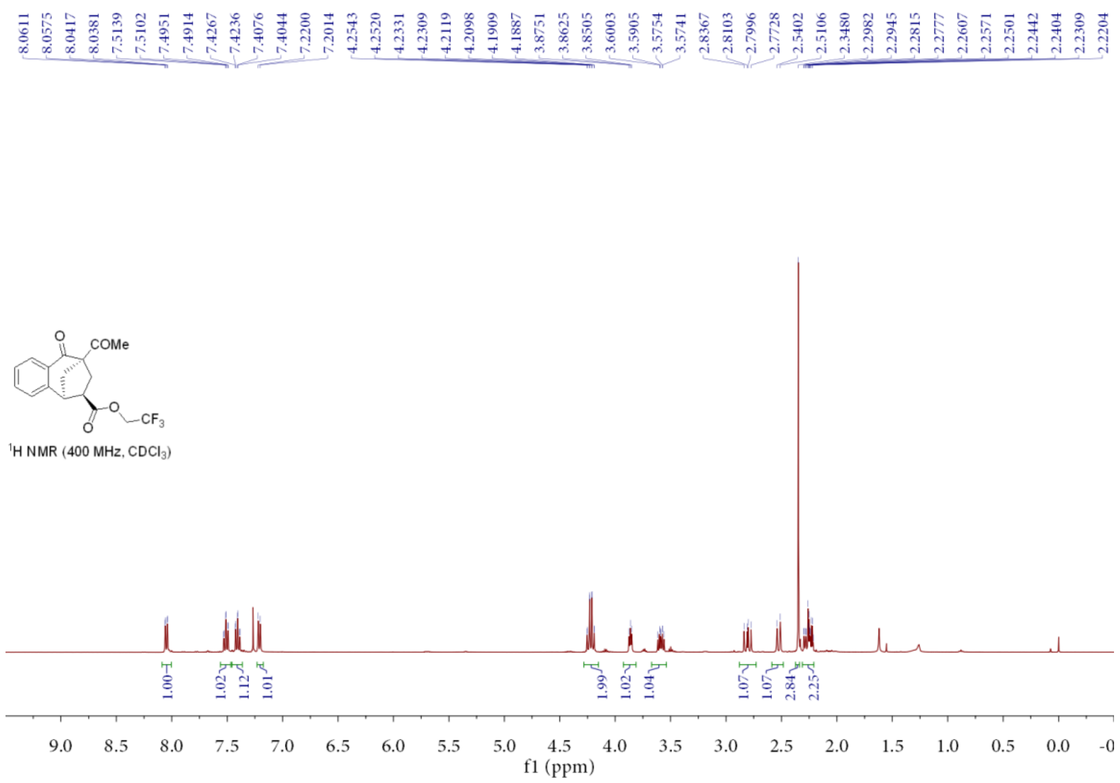

Supplementary Figure 127. ^{1}H NMR of **3af**

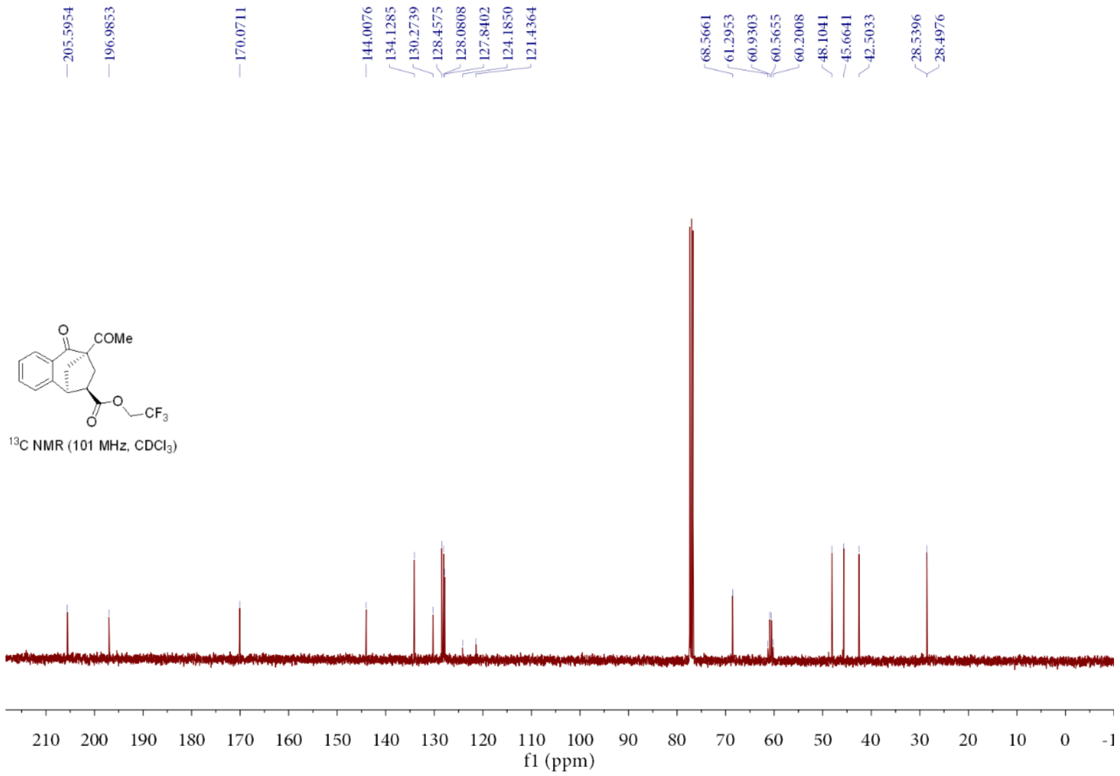

Supplementary Figure 128. ^{13}C NMR of **3af**

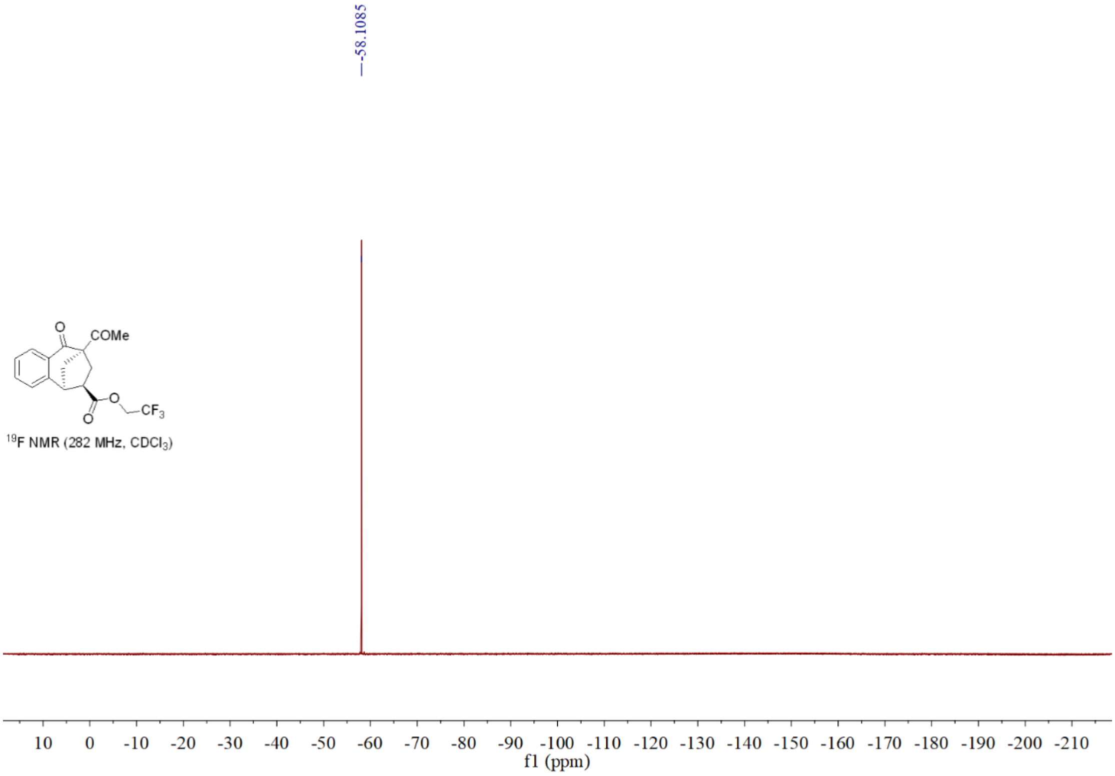

Supplementary Figure 129. ^{19}F NMR of **3af**

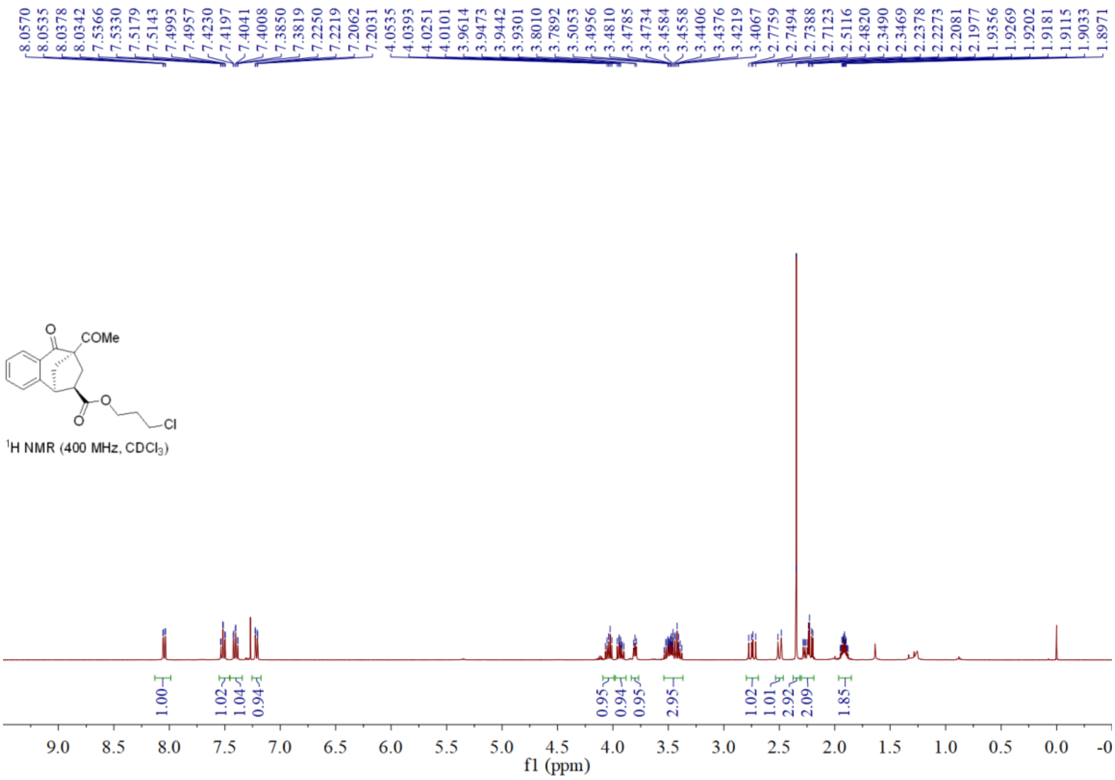

Supplementary Figure 130. ^{1}H NMR of **3ag**

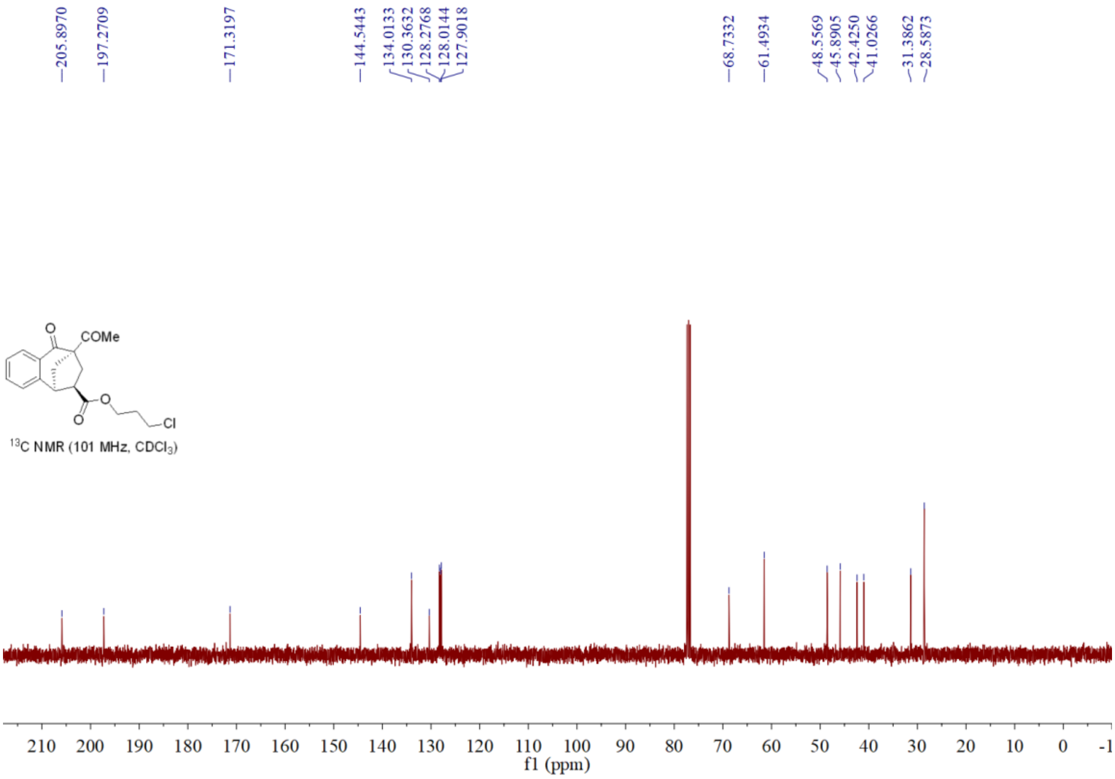

Supplementary Figure 131. ^{13}C NMR of **3ag**

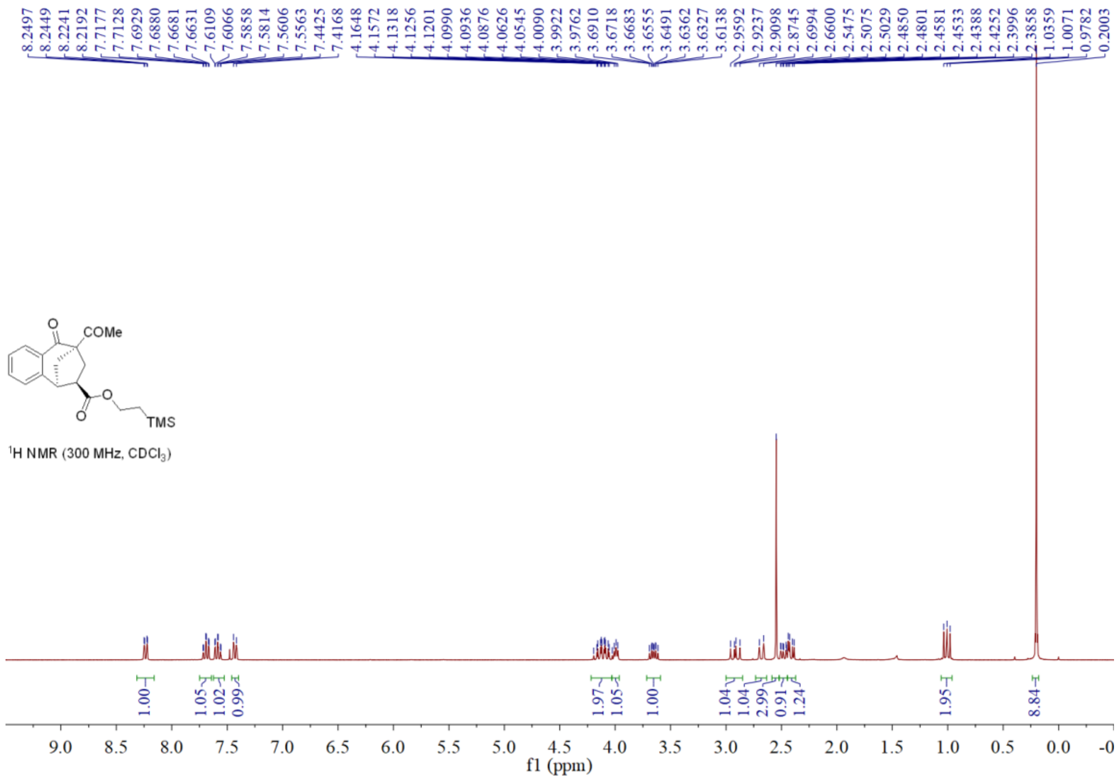

Supplementary Figure 132. ^{1}H NMR of **3ah**

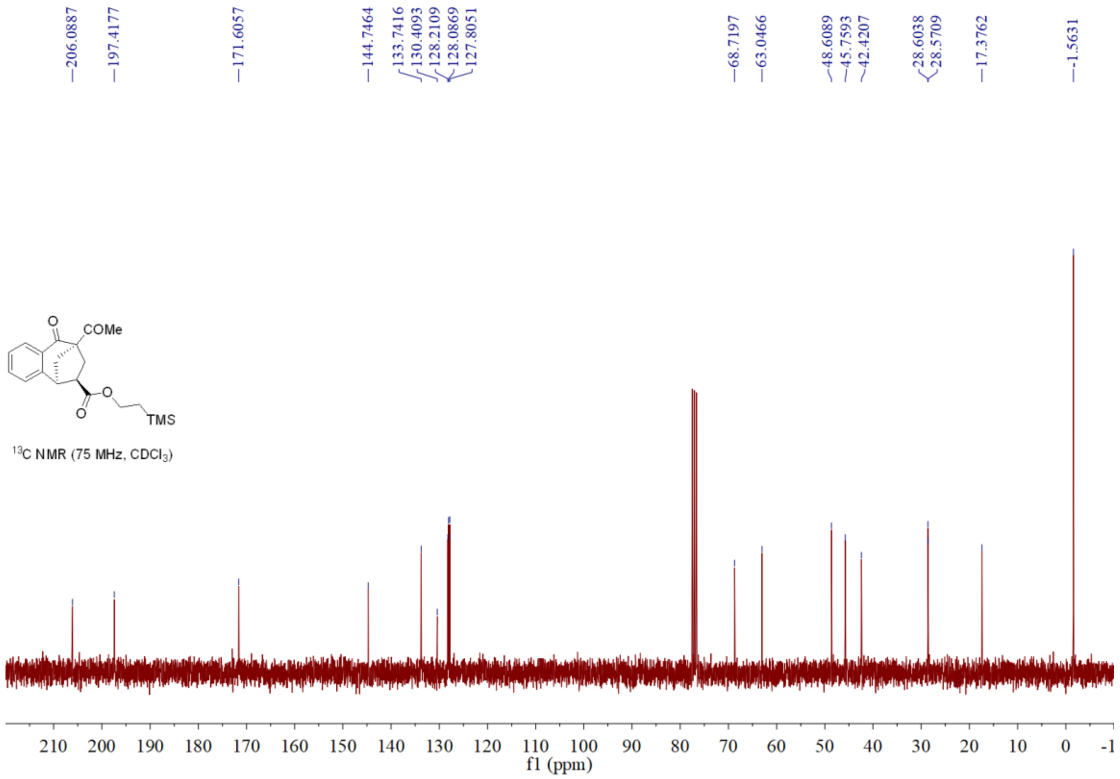

Supplementary Figure 133. ^{13}C NMR of **3ah**

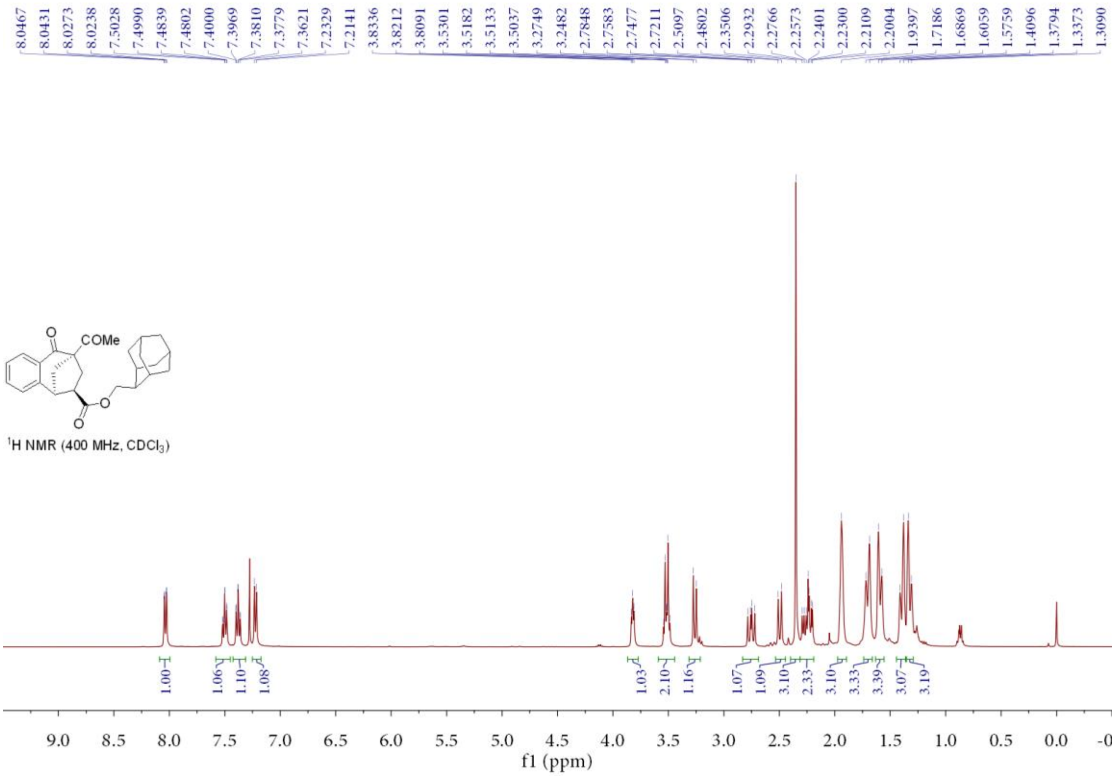

Supplementary Figure 134. ^{1}H NMR of **3ai**

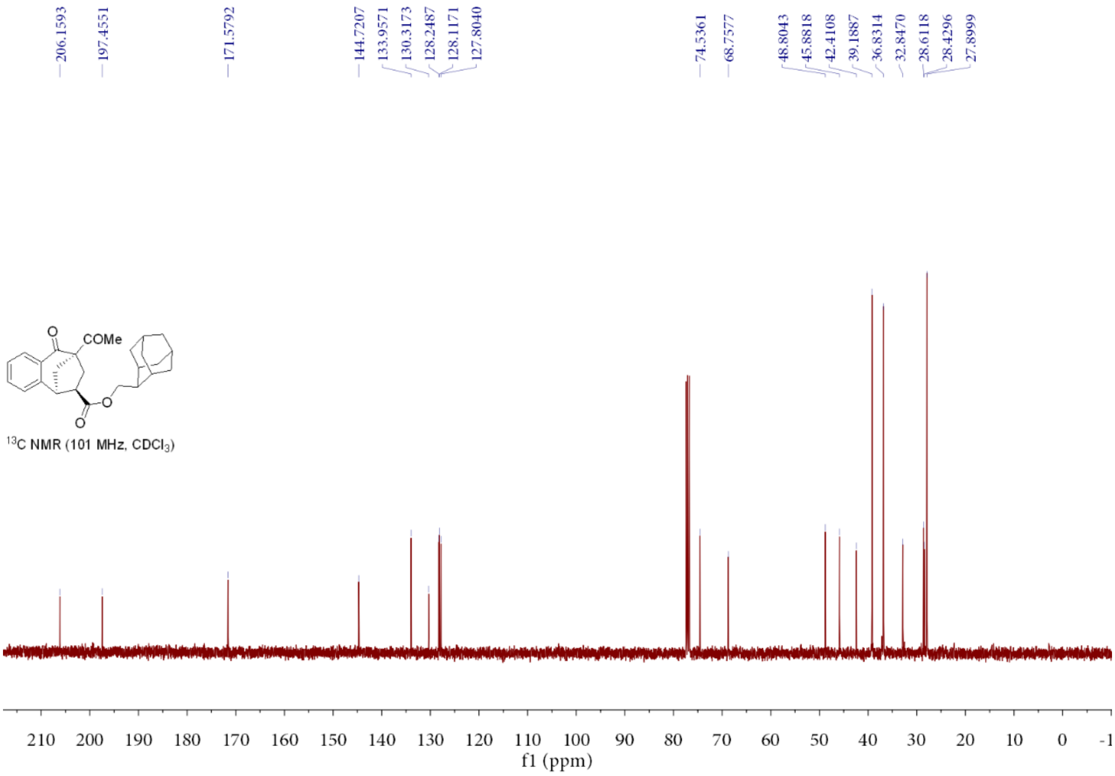

Supplementary Figure 135. ^{13}C NMR of **3ai**

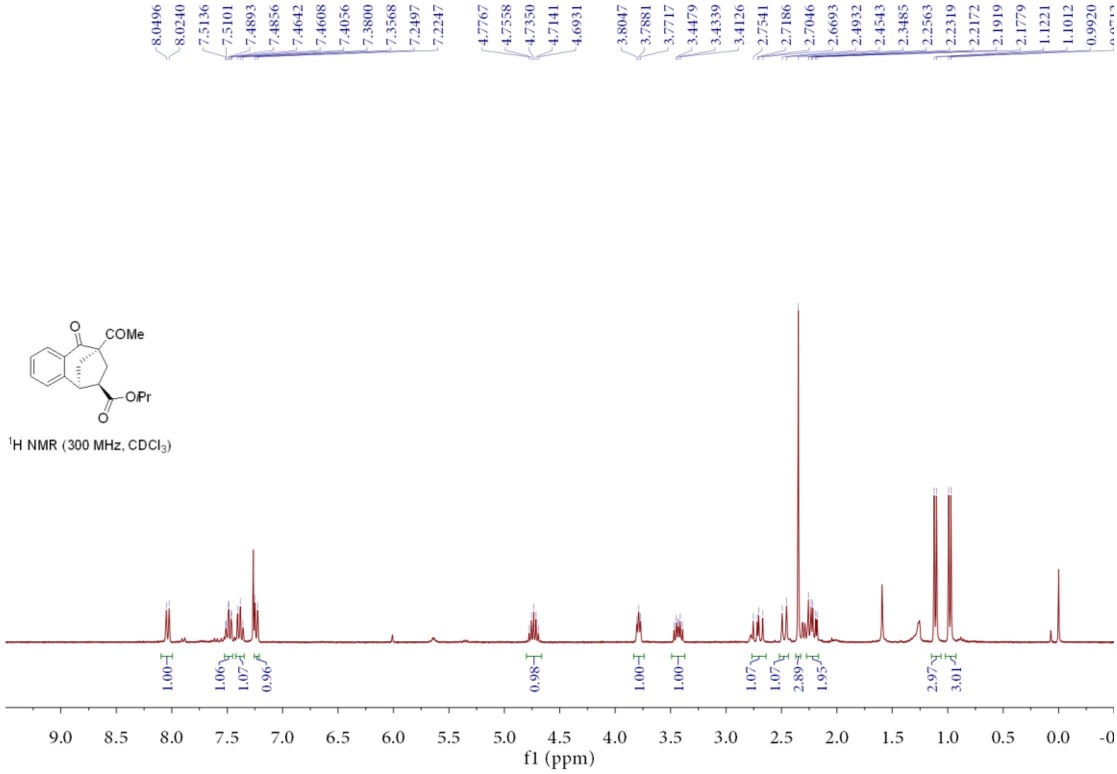

Supplementary Figure 136. ^{1}H NMR of **3aj**

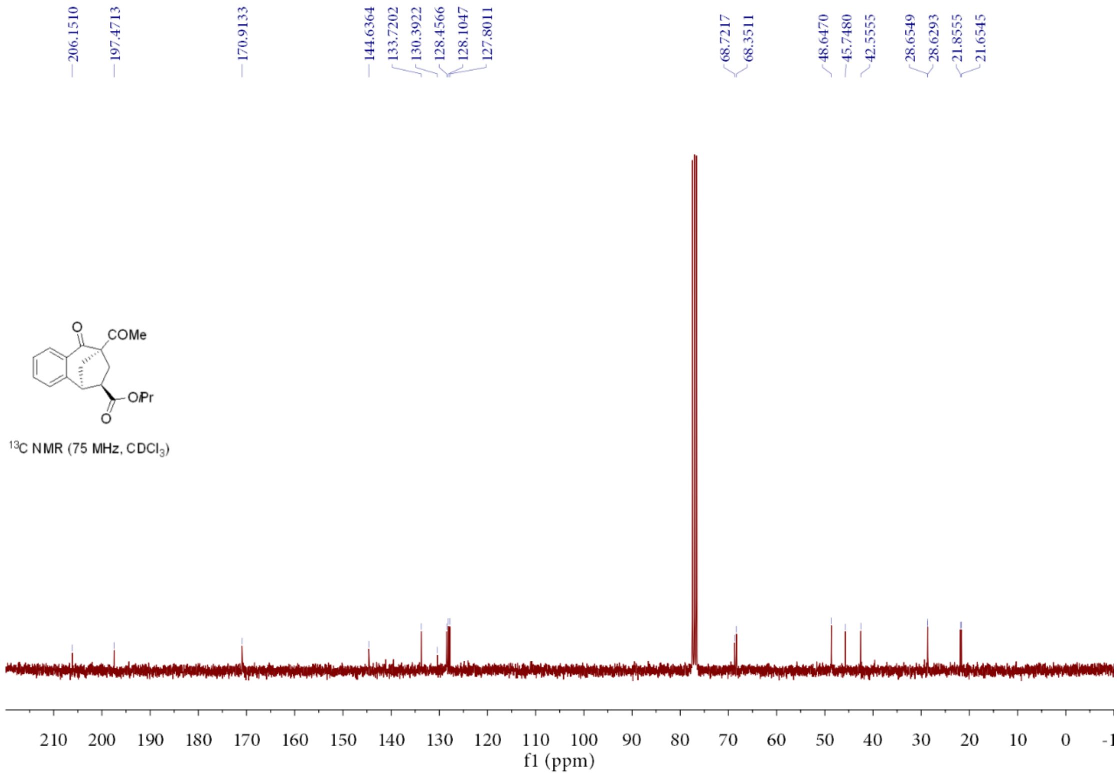

Supplementary Figure 137. ^{13}C NMR of **3aj**

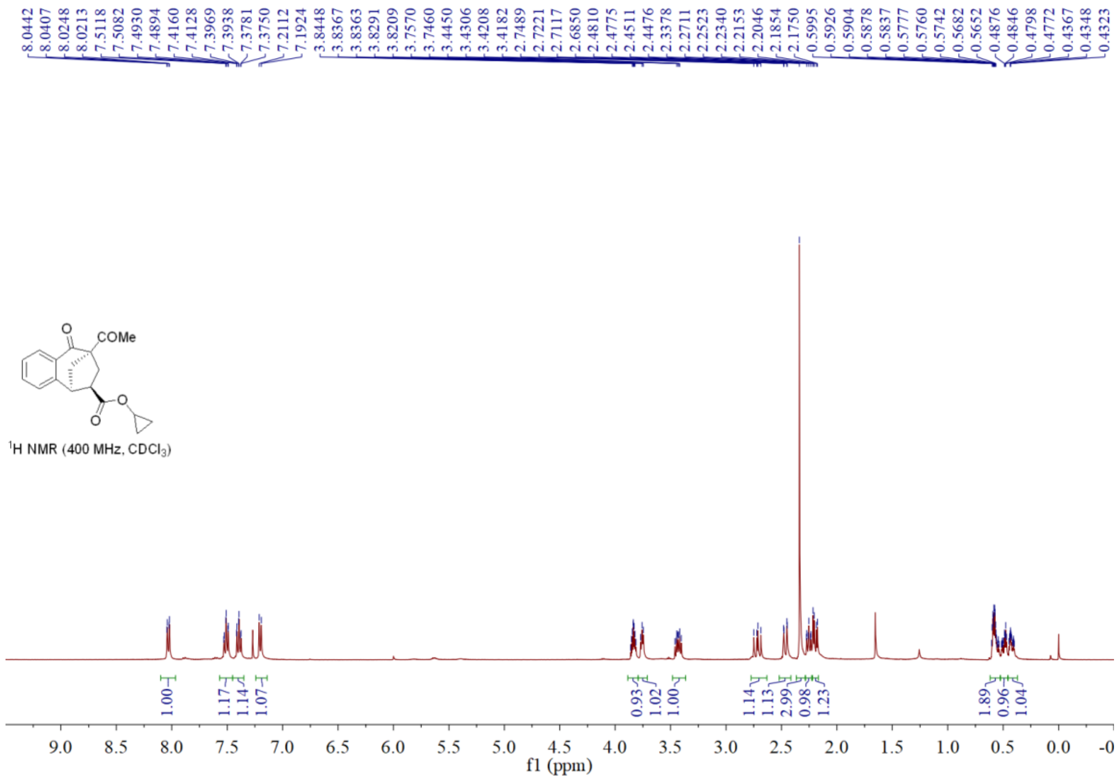

Supplementary Figure 138. ^{1}H NMR of **3ak**

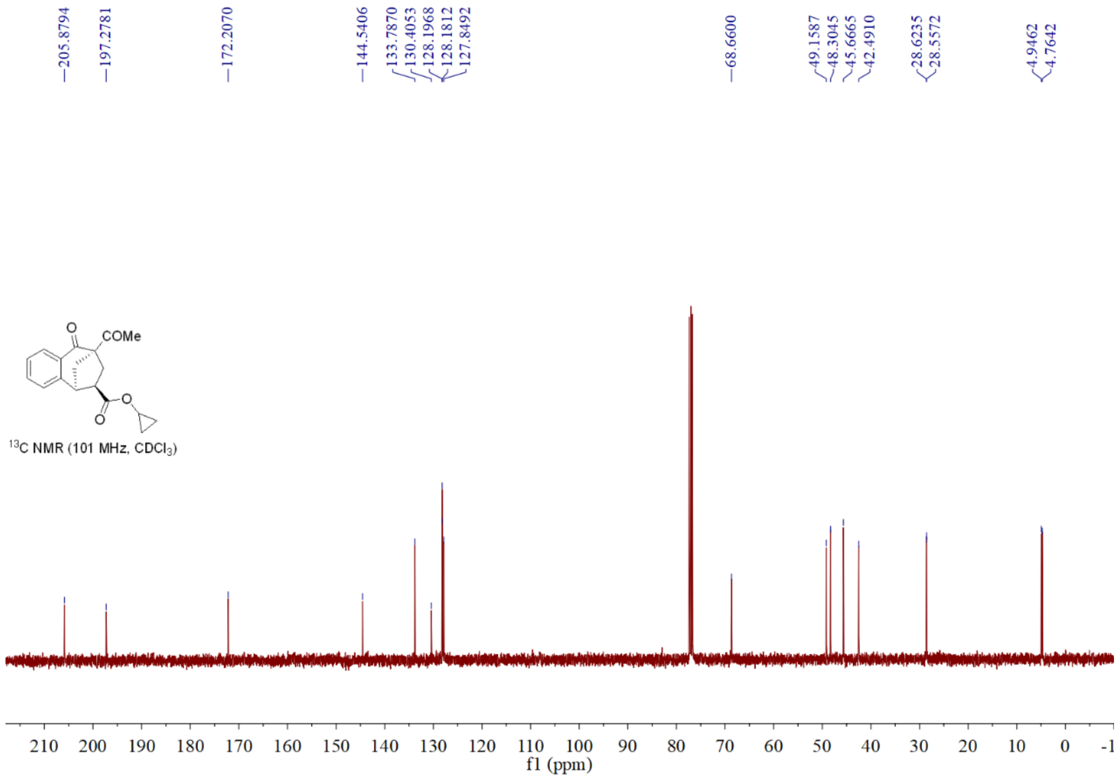

Supplementary Figure 139. ^{13}C NMR of **3ak**

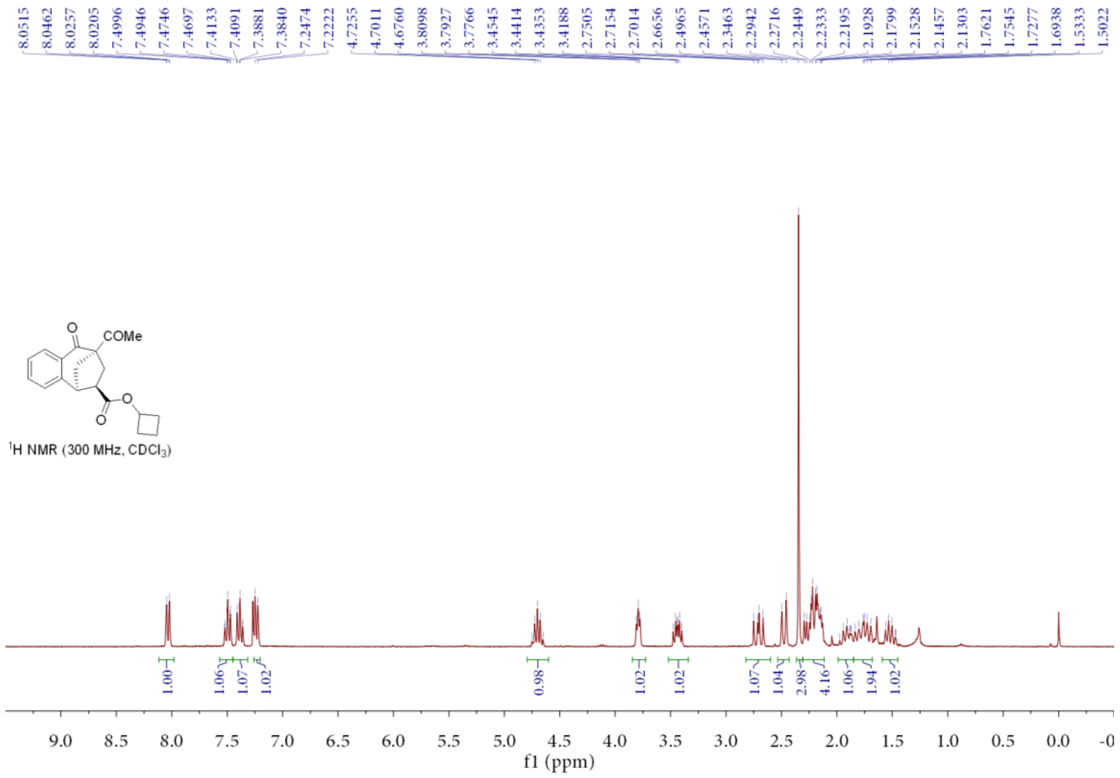

Supplementary Figure 140. ^{1}H NMR of **3al**

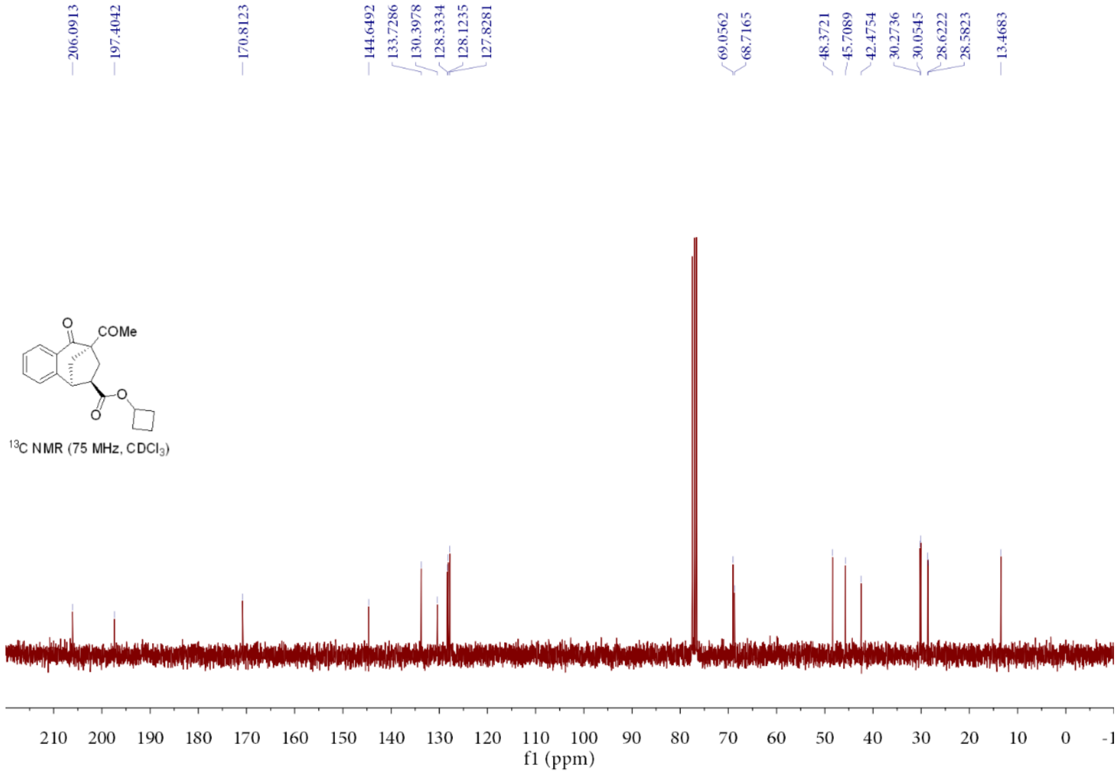

Supplementary Figure 141. ^{13}C NMR of **3al**

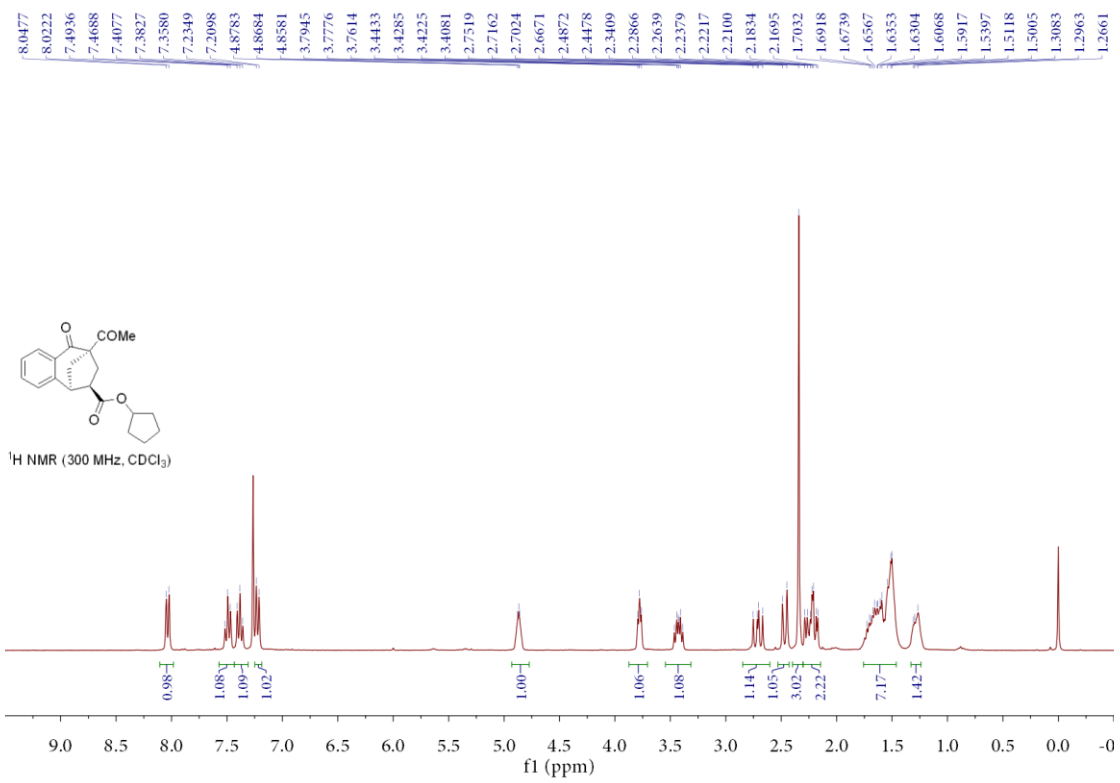

Supplementary Figure 142. ^{1}H NMR of **3am**

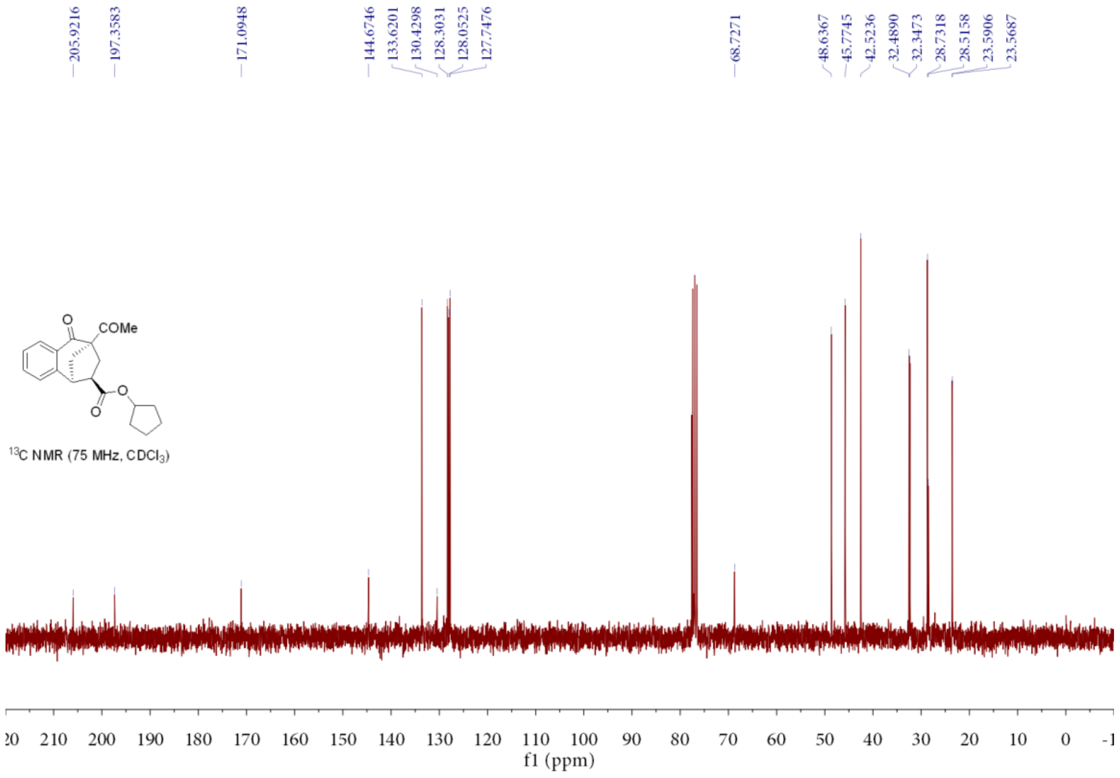

Supplementary Figure 143. ^{13}C NMR of **3am**

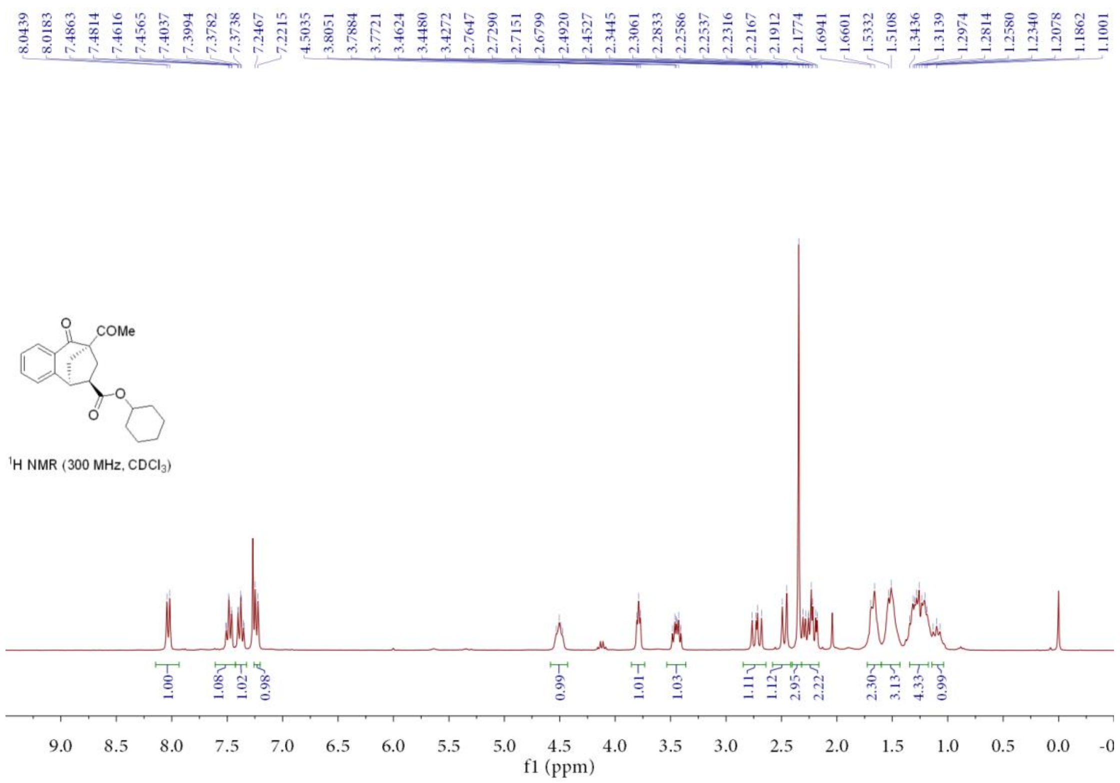

Supplementary Figure 144. ^{1}H NMR of **3an**

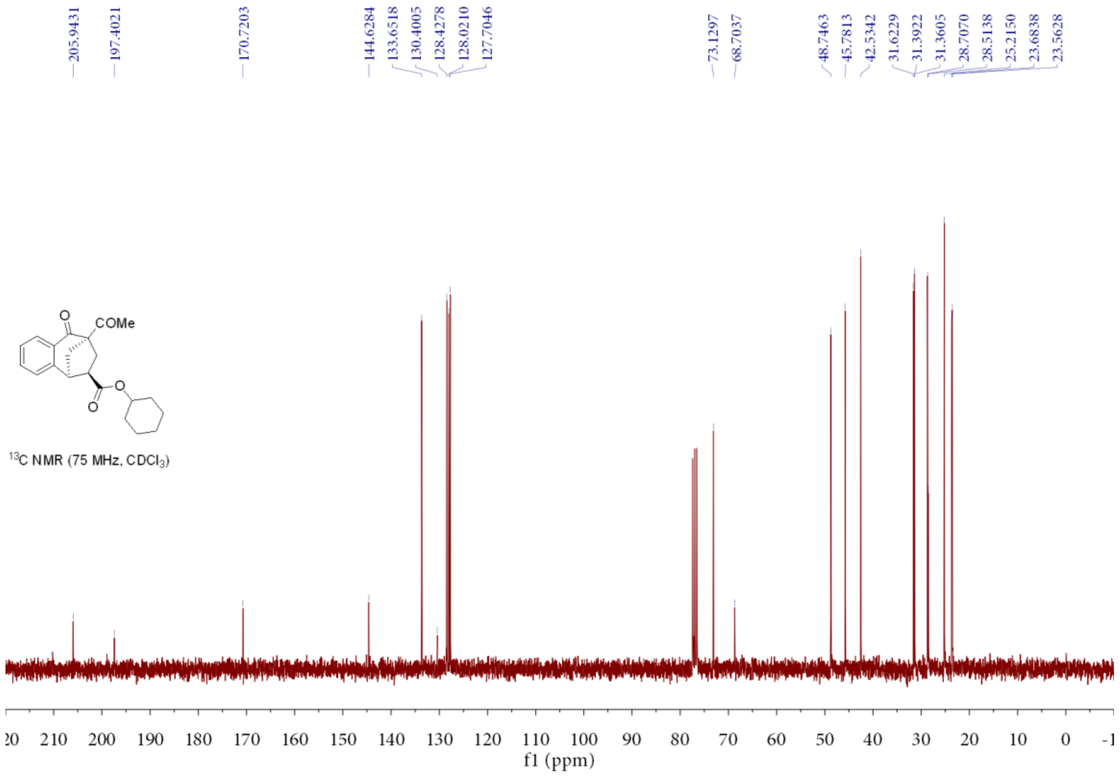

Supplementary Figure 145. ^{13}C NMR of **3an**

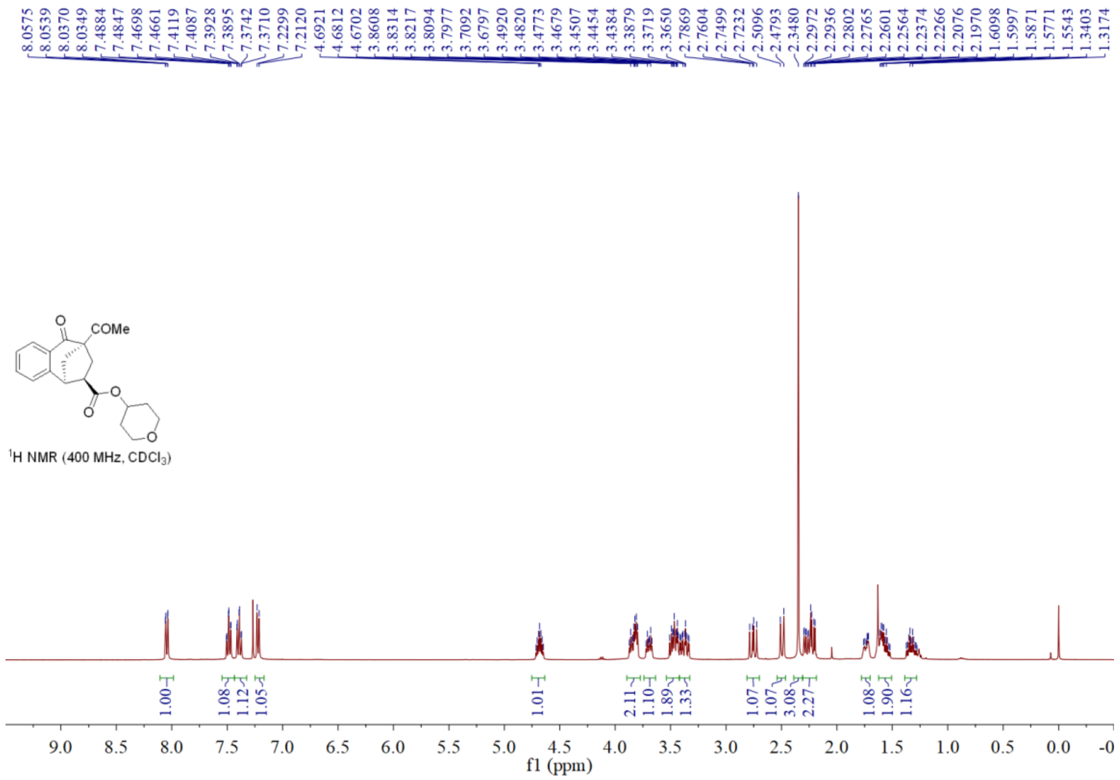

Supplementary Figure 146. ^{1}H NMR of **3ao**

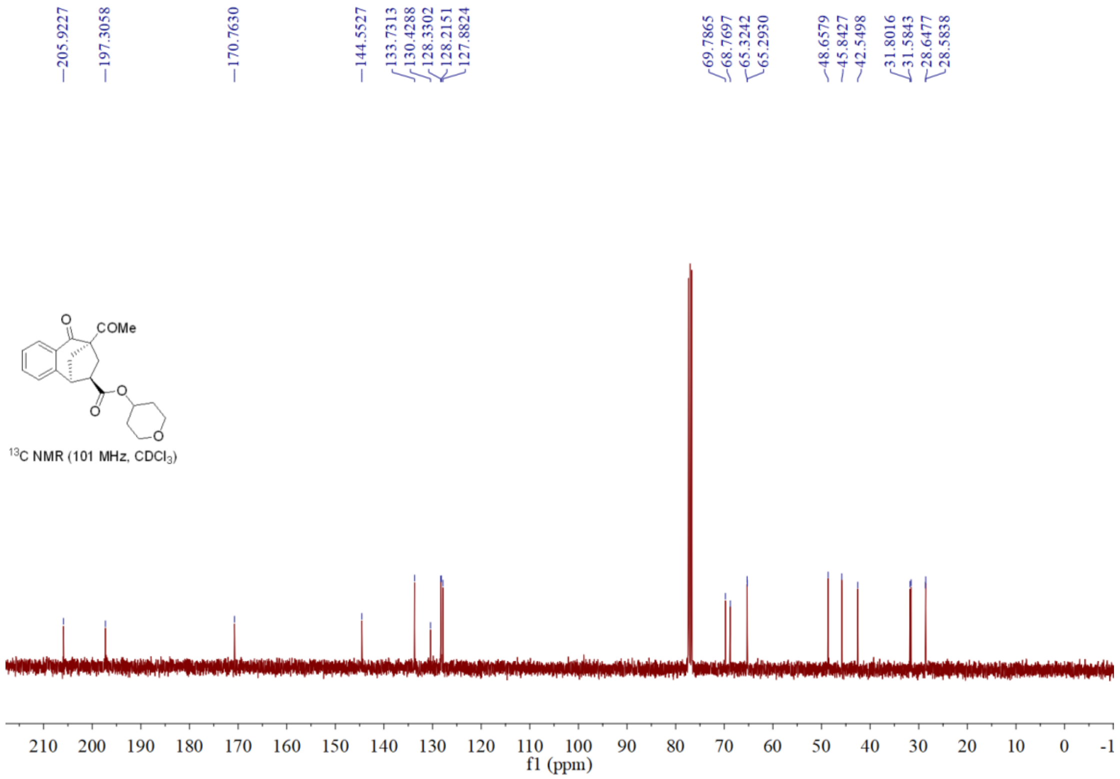

Supplementary Figure 147. ^{13}C NMR of **3ao**

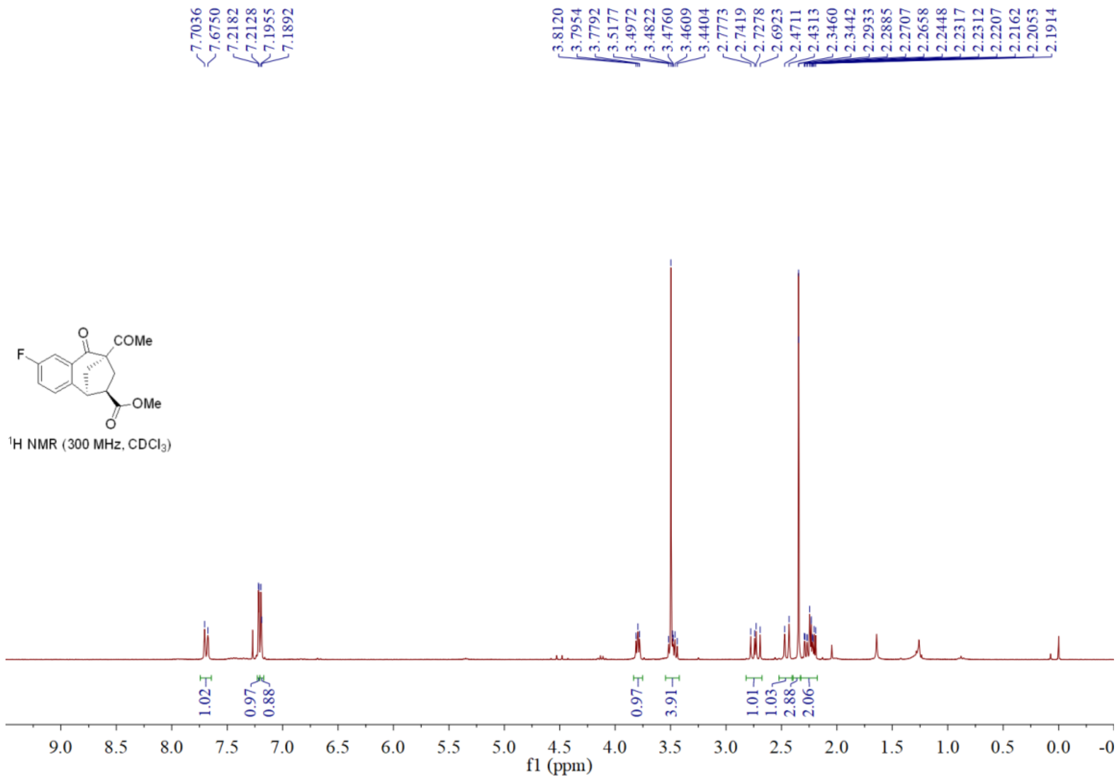

Supplementary Figure 148. ^{1}H NMR of **3ba**

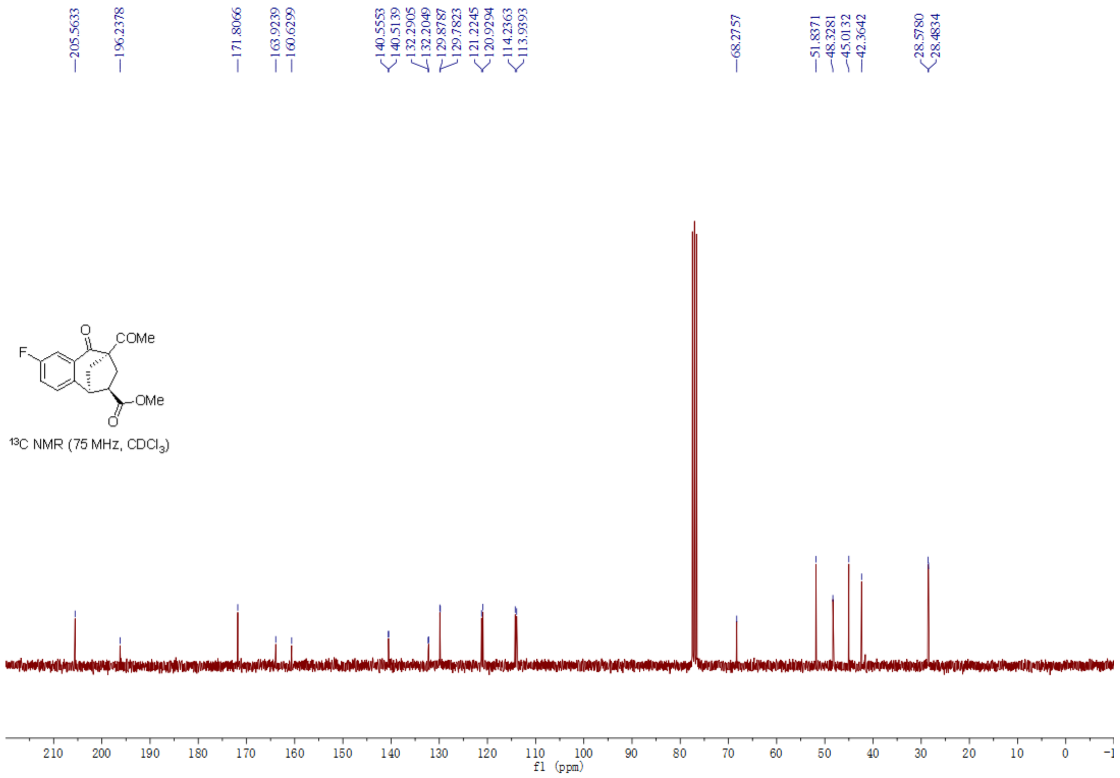

Supplementary Figure 149. ^{13}C NMR of **3ba**

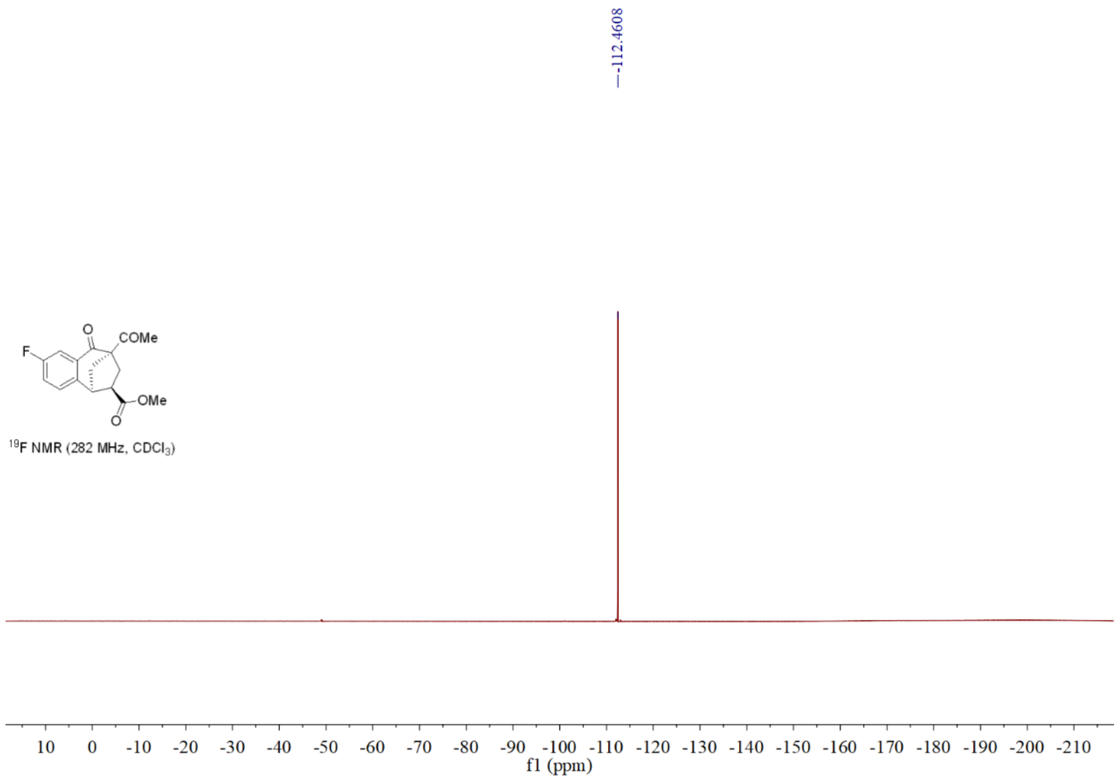

Supplementary Figure 150. ^{19}F NMR of **3ba**

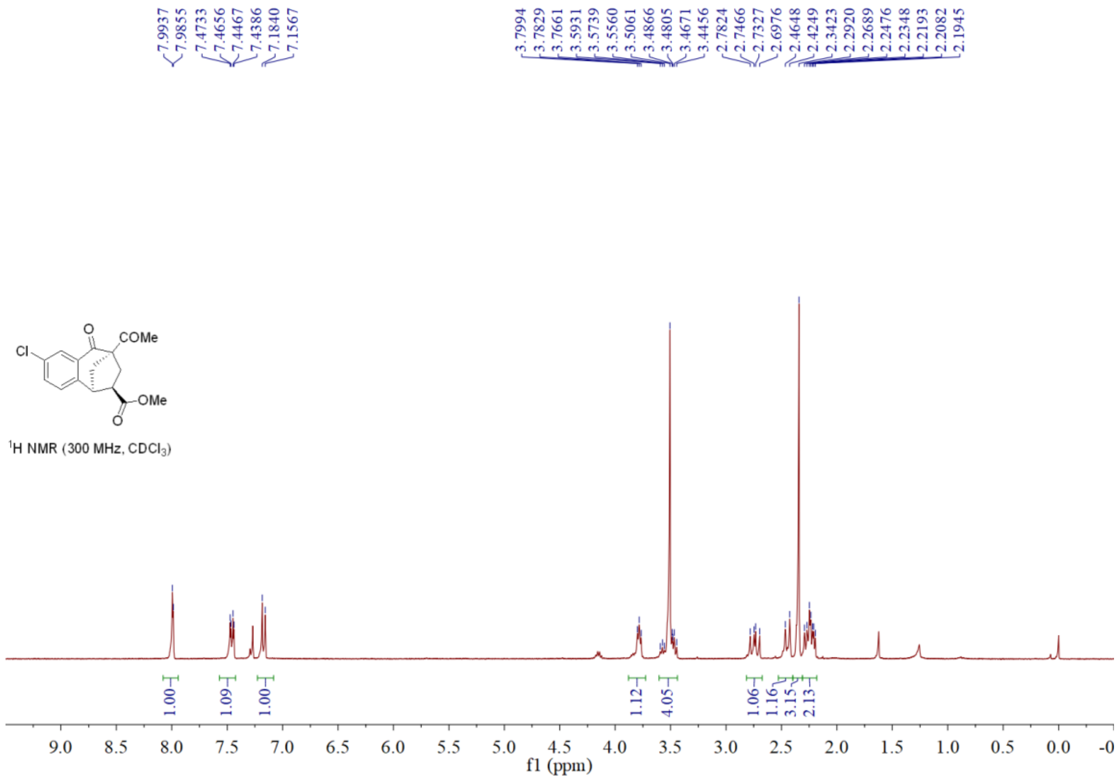

Supplementary Figure 151. ^{1}H NMR of **3ca**

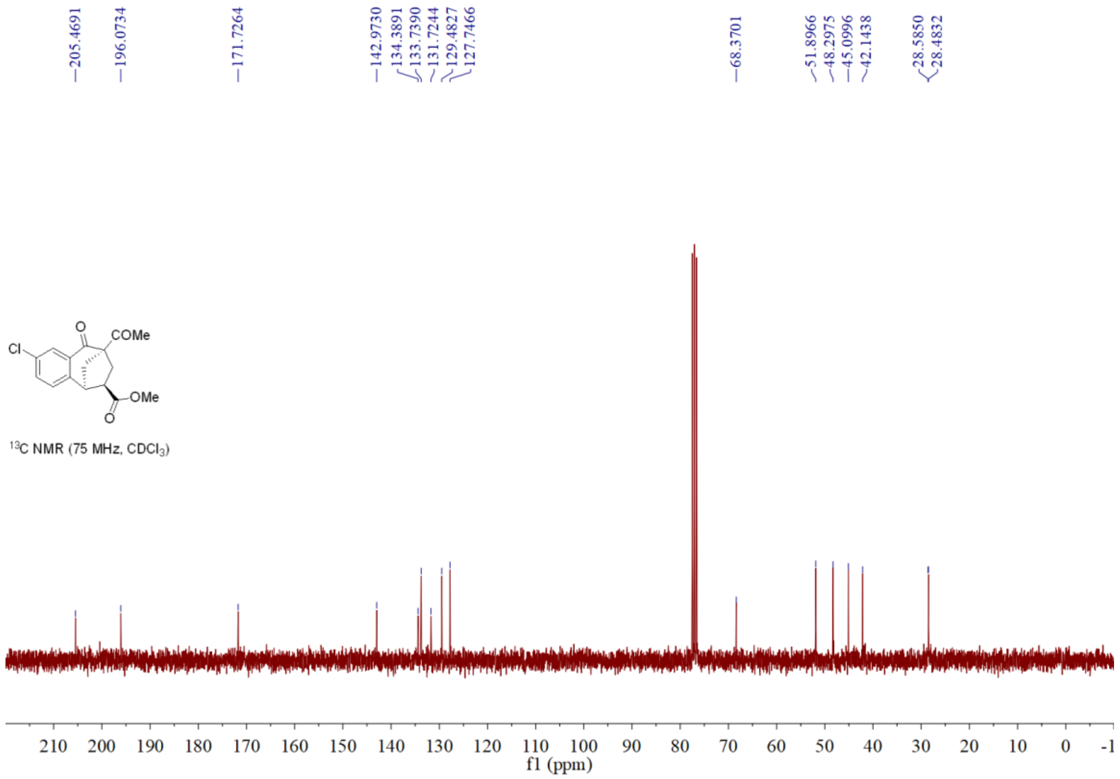

Supplementary Figure 152. ^{13}C NMR of **3ca**

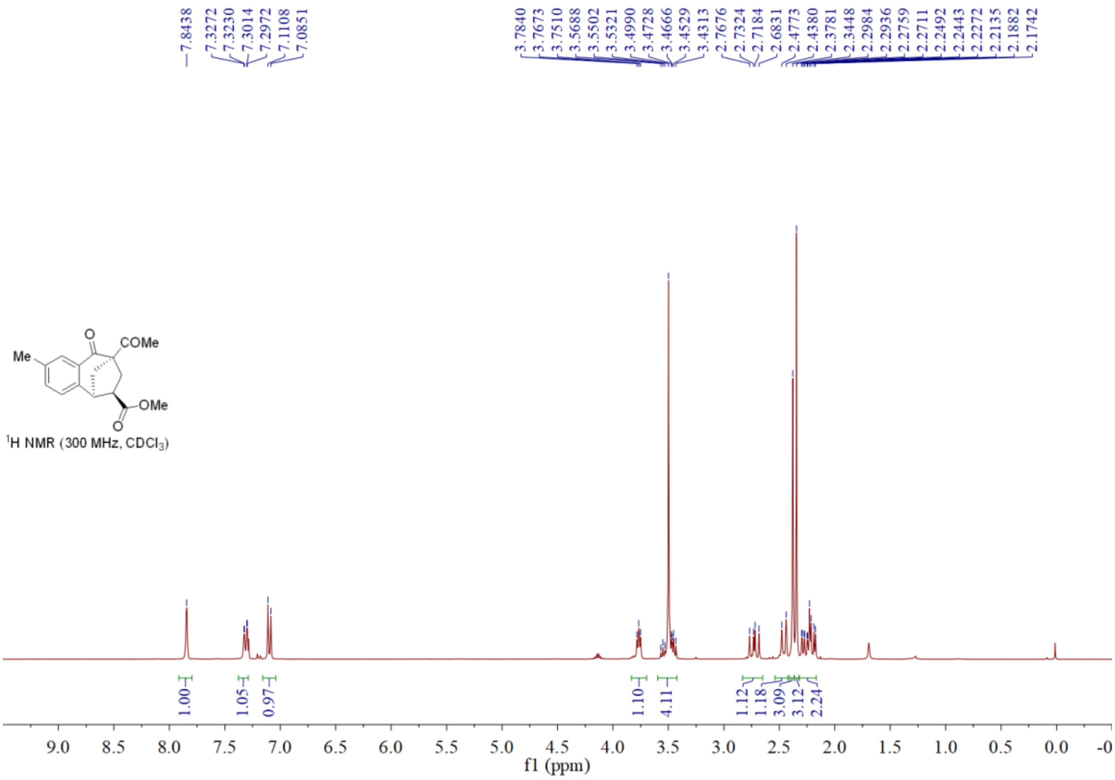

Supplementary Figure 153. ^{1}H NMR of **3da**

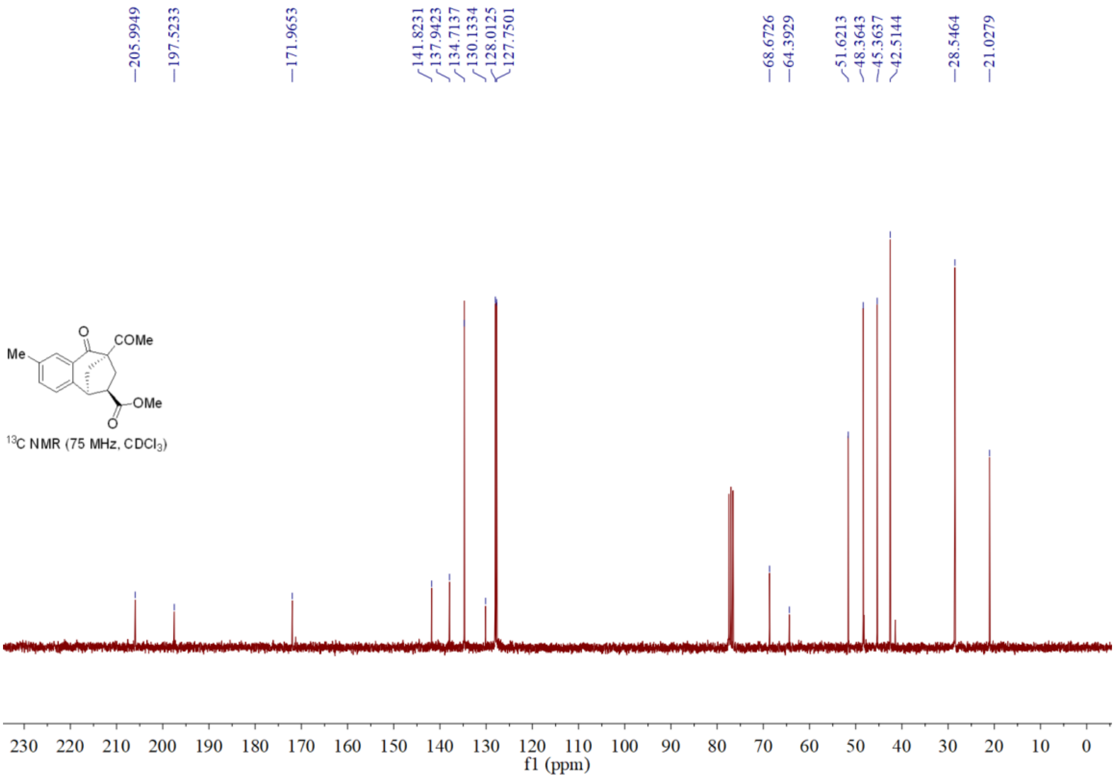

Supplementary Figure 154. ^{13}C NMR of **3da**

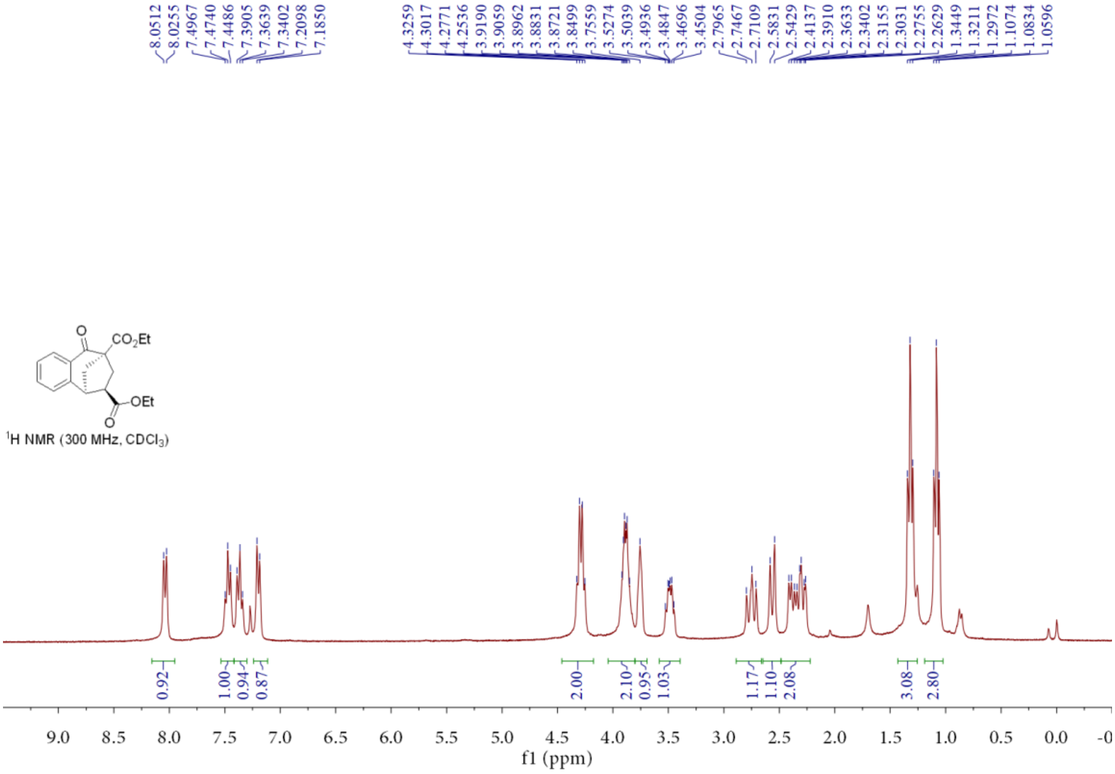

Supplementary Figure 155. ^{1}H NMR of **3eb**

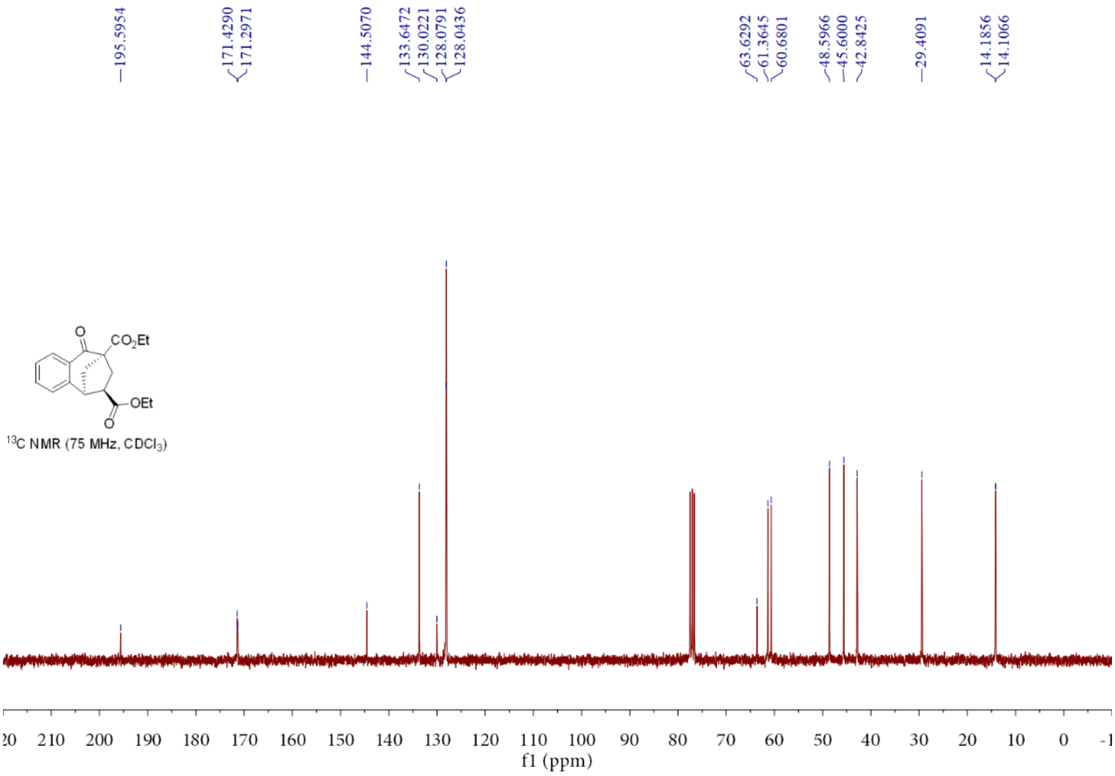

Supplementary Figure 156. ^{13}C NMR of **3eb**

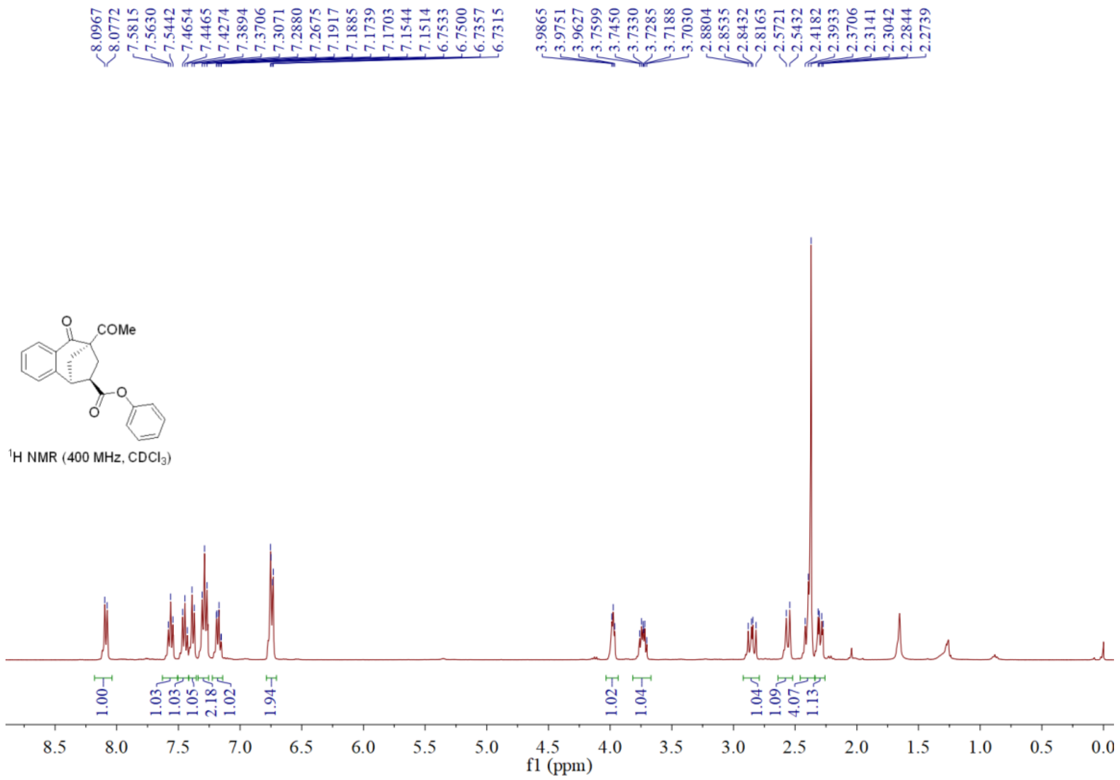

Supplementary Figure 157. ^{1}H NMR of **5aa**

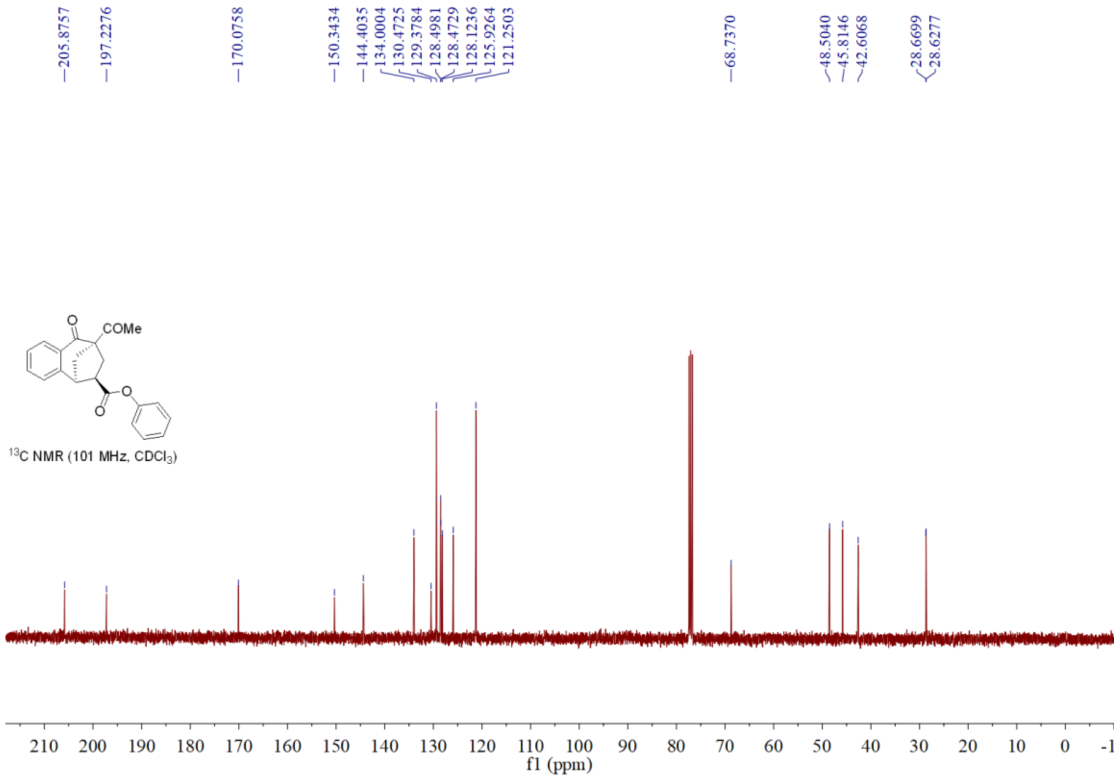

Supplementary Figure 158. ^{13}C NMR of **5aa**

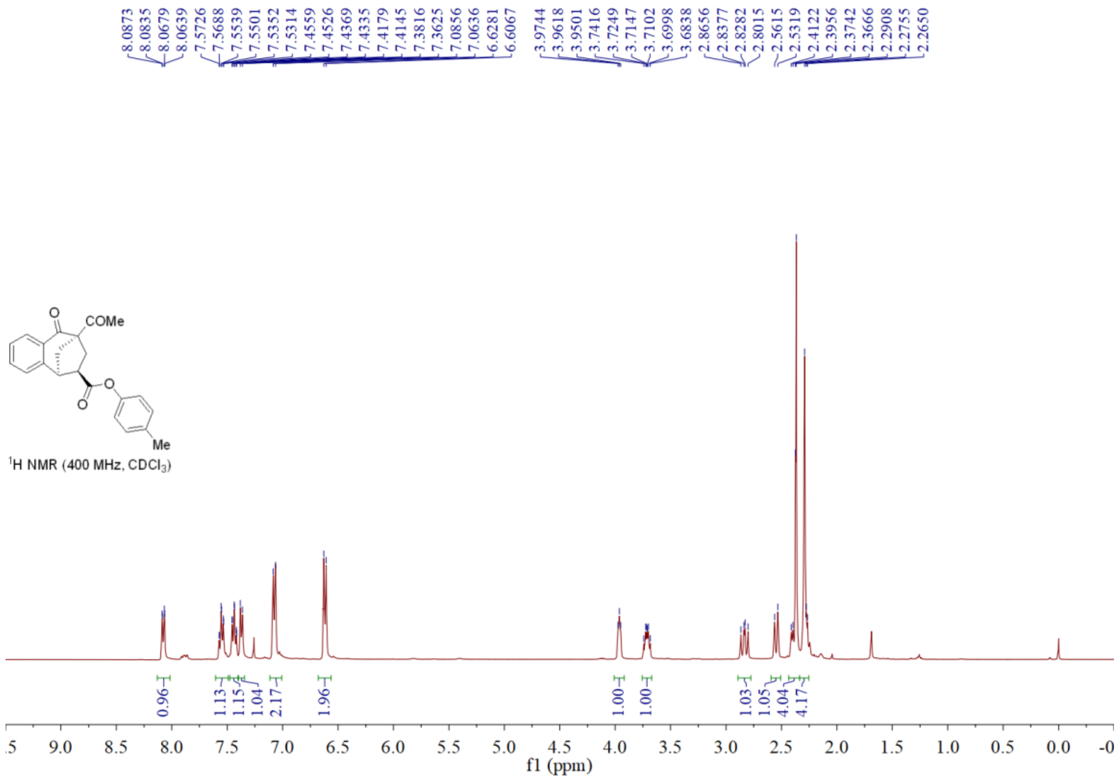

Supplementary Figure 159. ^{1}H NMR of **5ab**

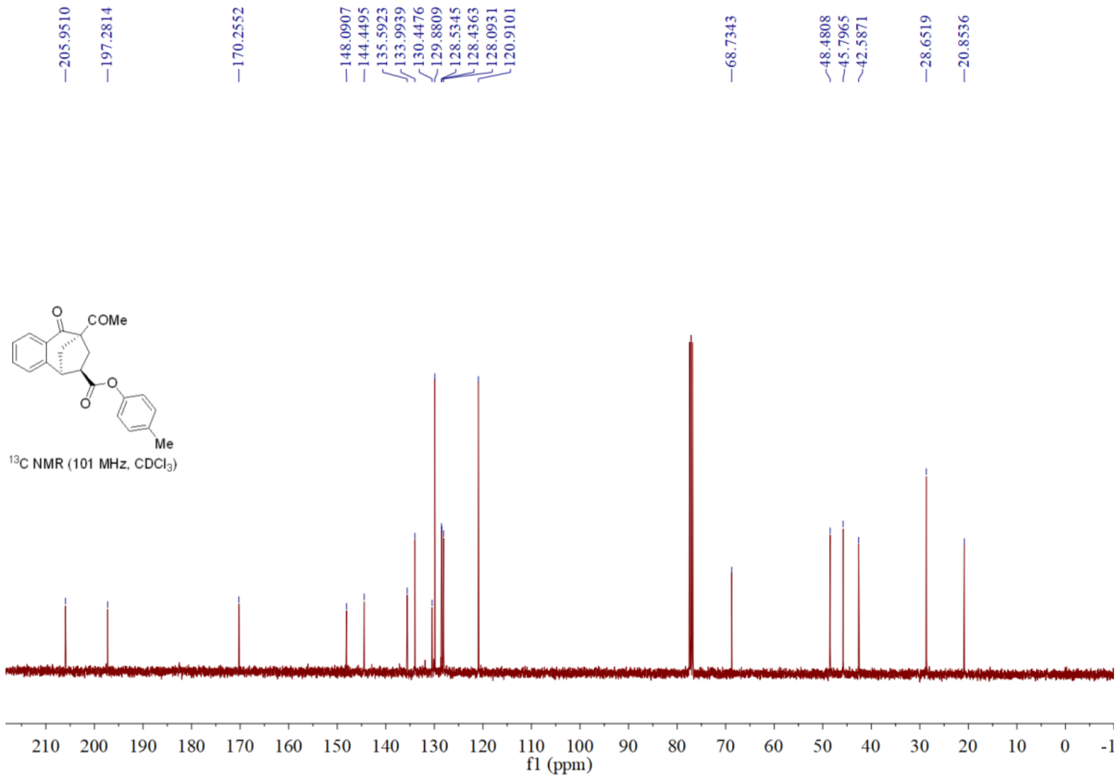

Supplementary Figure 160. ^{13}C NMR of **5ab**

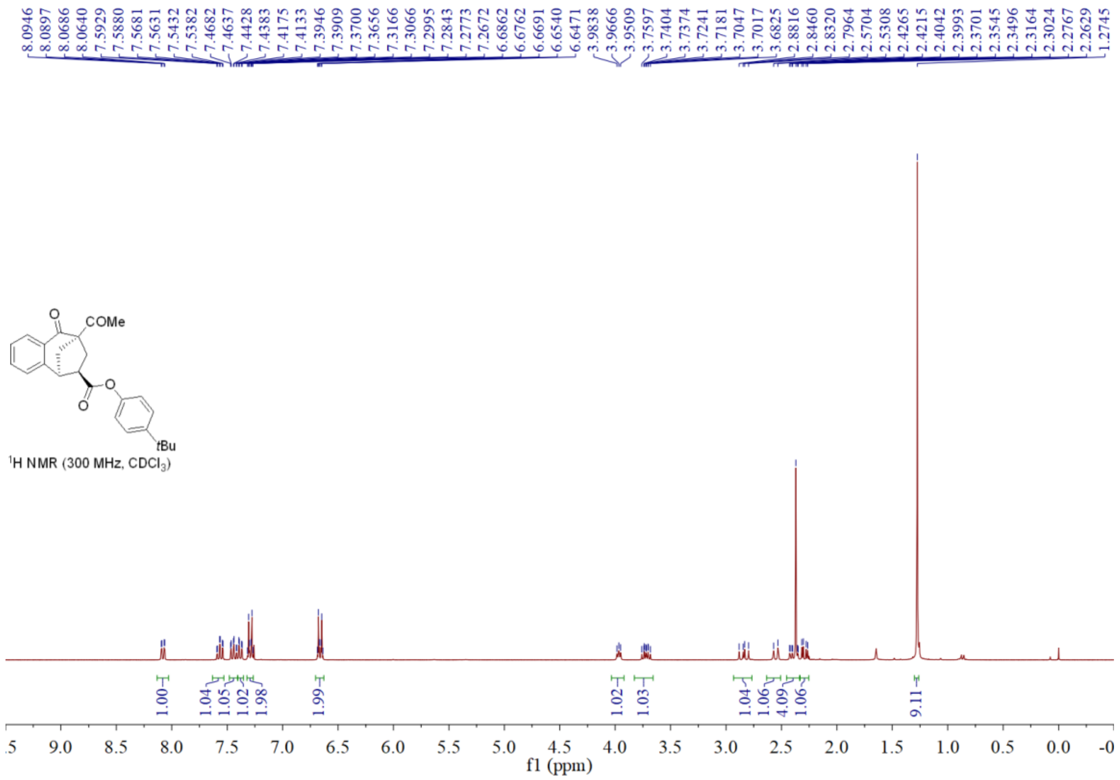

Supplementary Figure 161. ^{1}H NMR of **5ac**

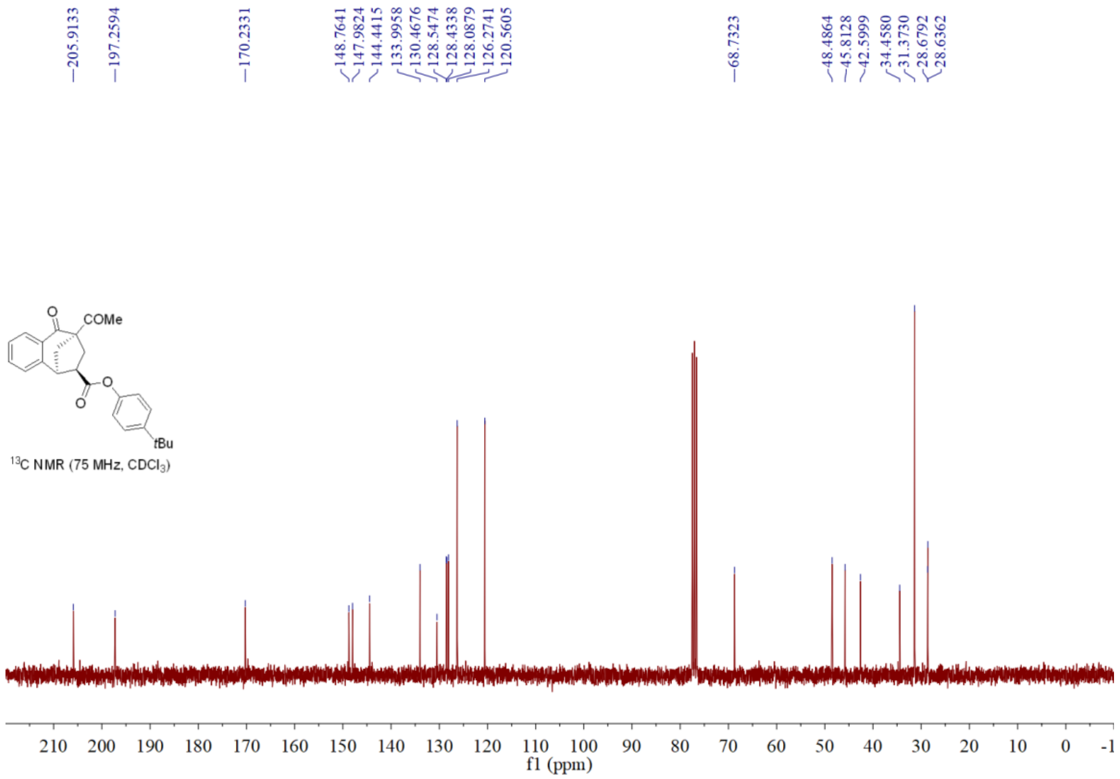

Supplementary Figure 162. ^{13}C NMR of **5ac**

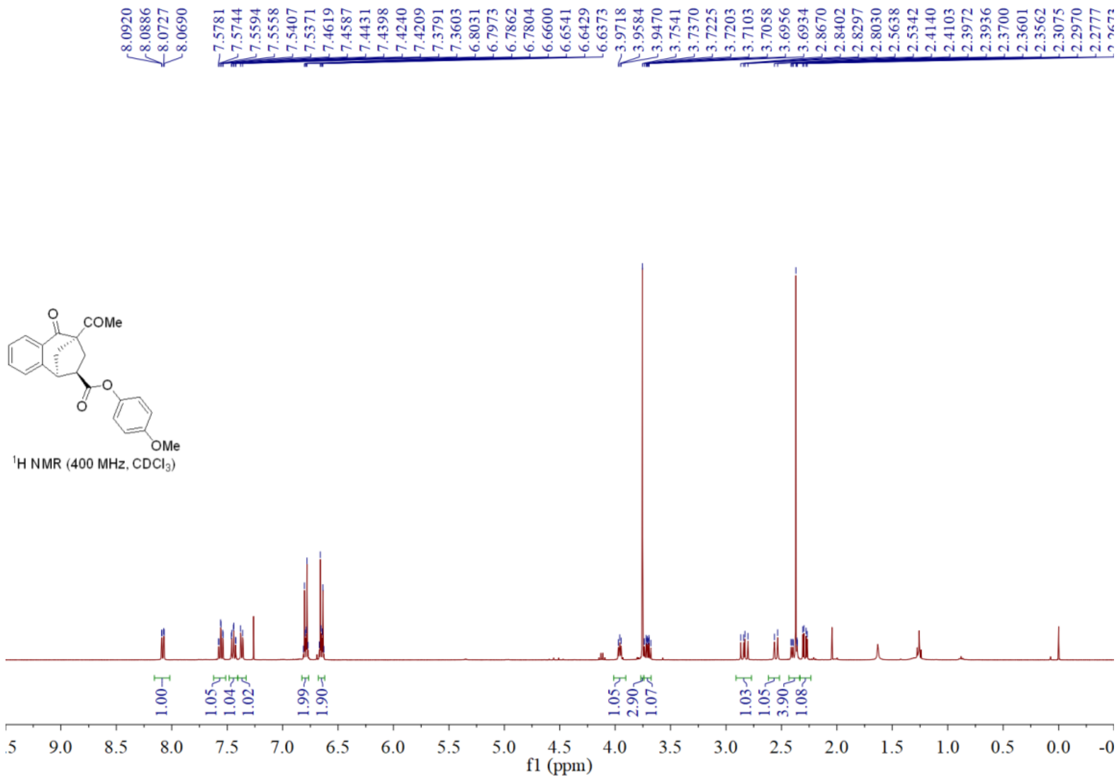

Supplementary Figure 163. ^{1}H NMR of **5ad**

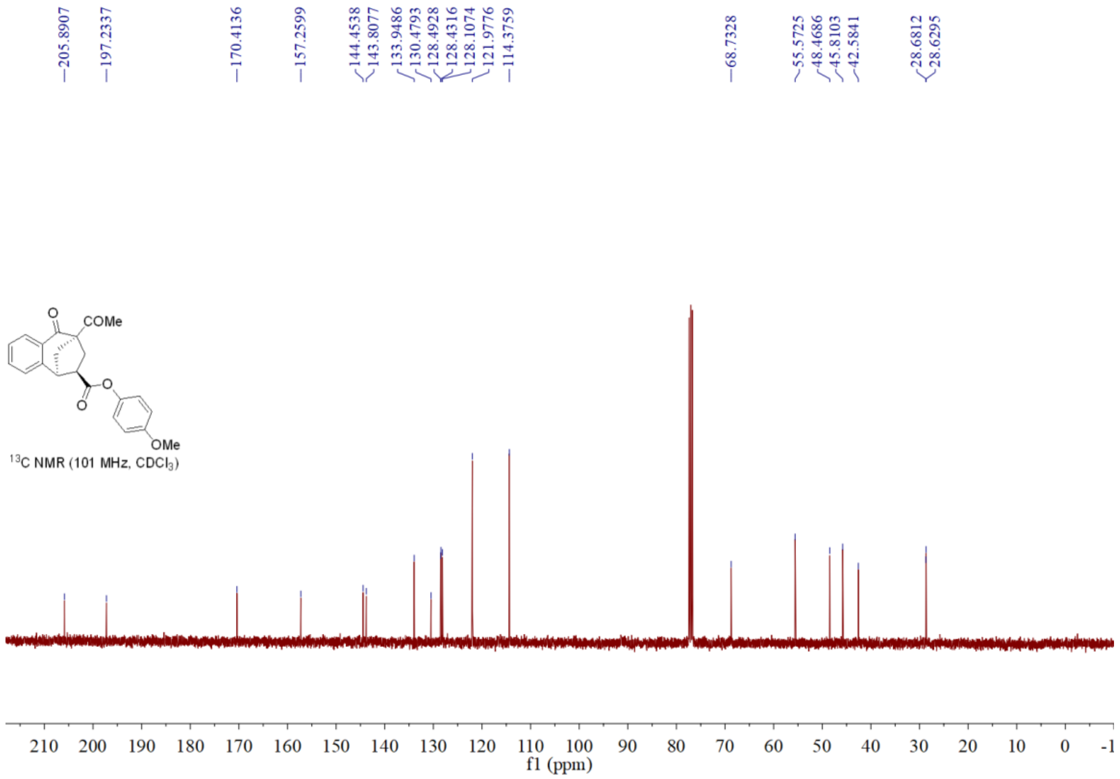

Supplementary Figure 164. ^{13}C NMR of **5ad**

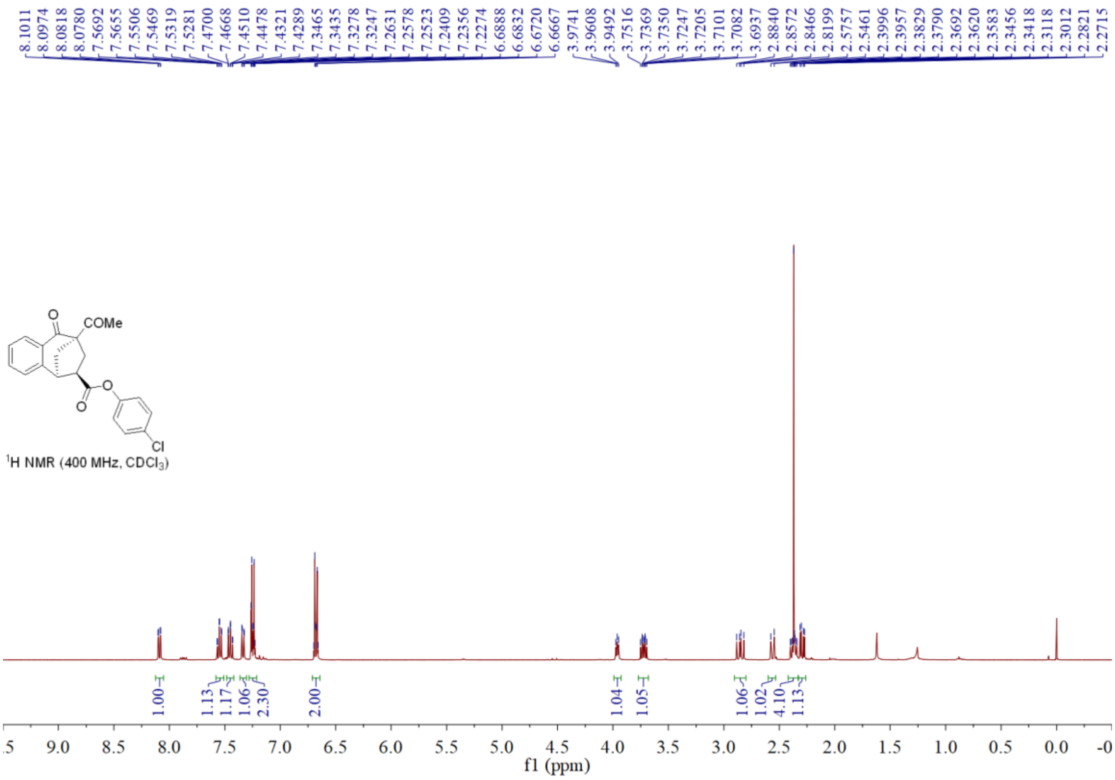

Supplementary Figure 165. ^{1}H NMR of **5ae**

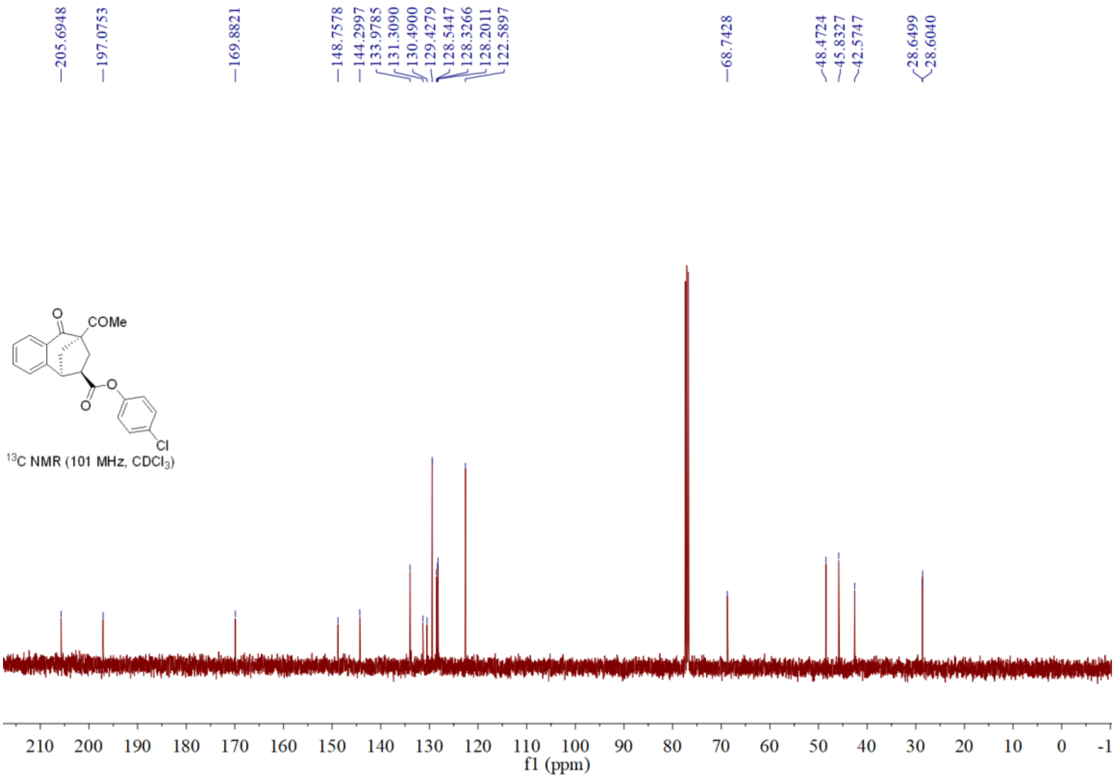

Supplementary Figure 166. ^{13}C NMR of **5ae**

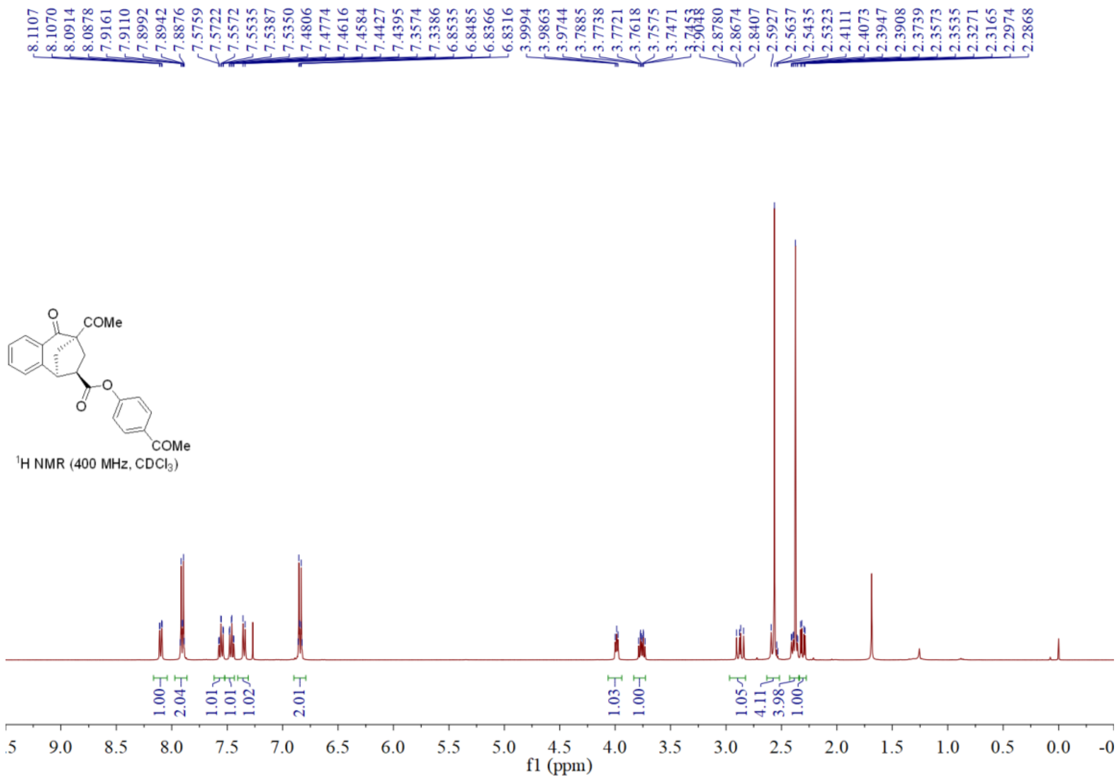

Supplementary Figure 167. ^{1}H NMR of **5af**

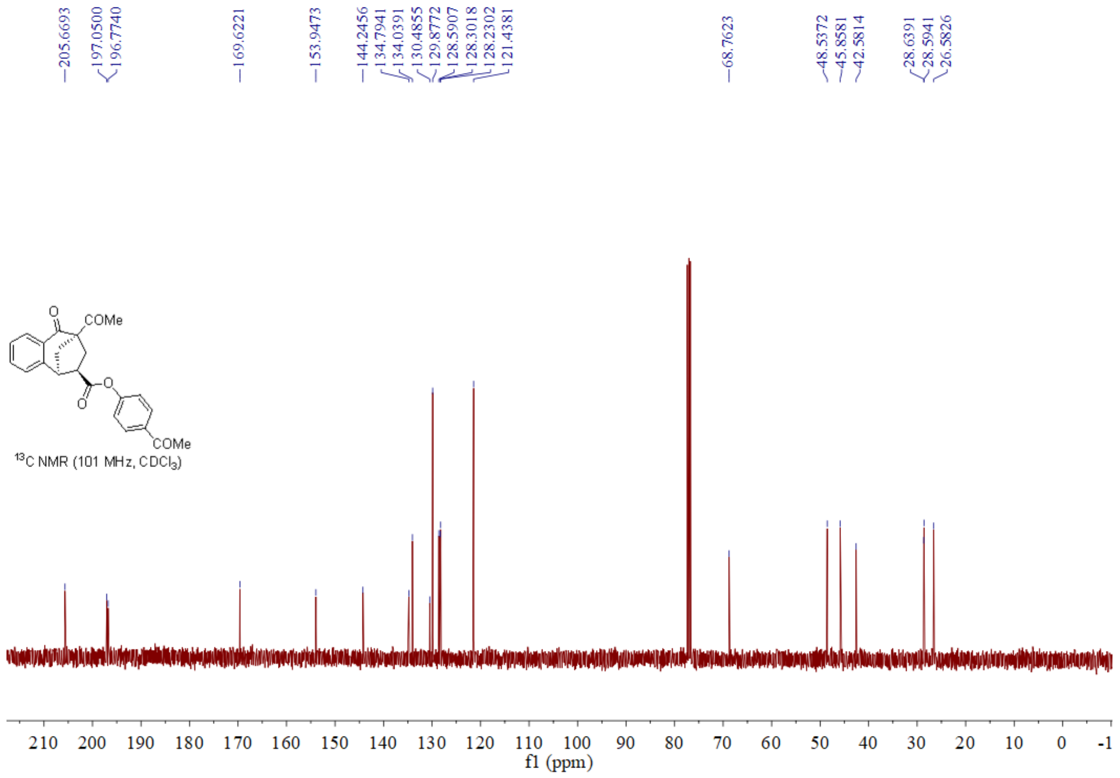

Supplementary Figure 168. ^{13}C NMR of **5af**

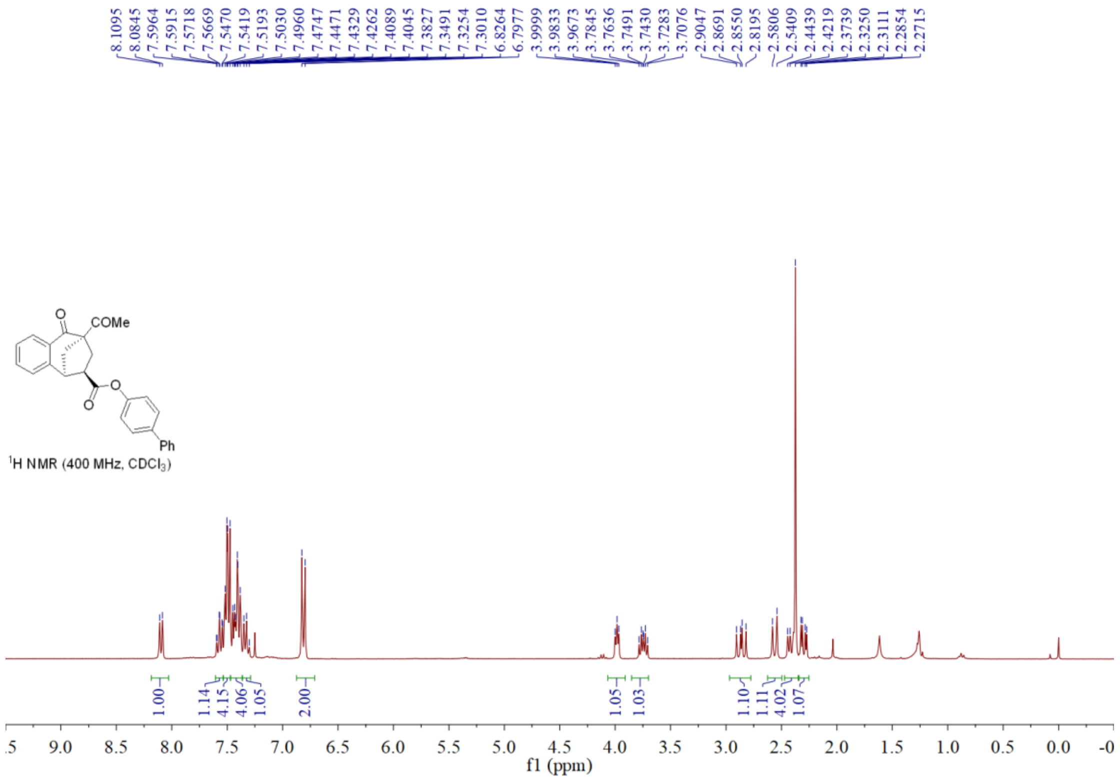

Supplementary Figure 169. ^{1}H NMR of **5ag**

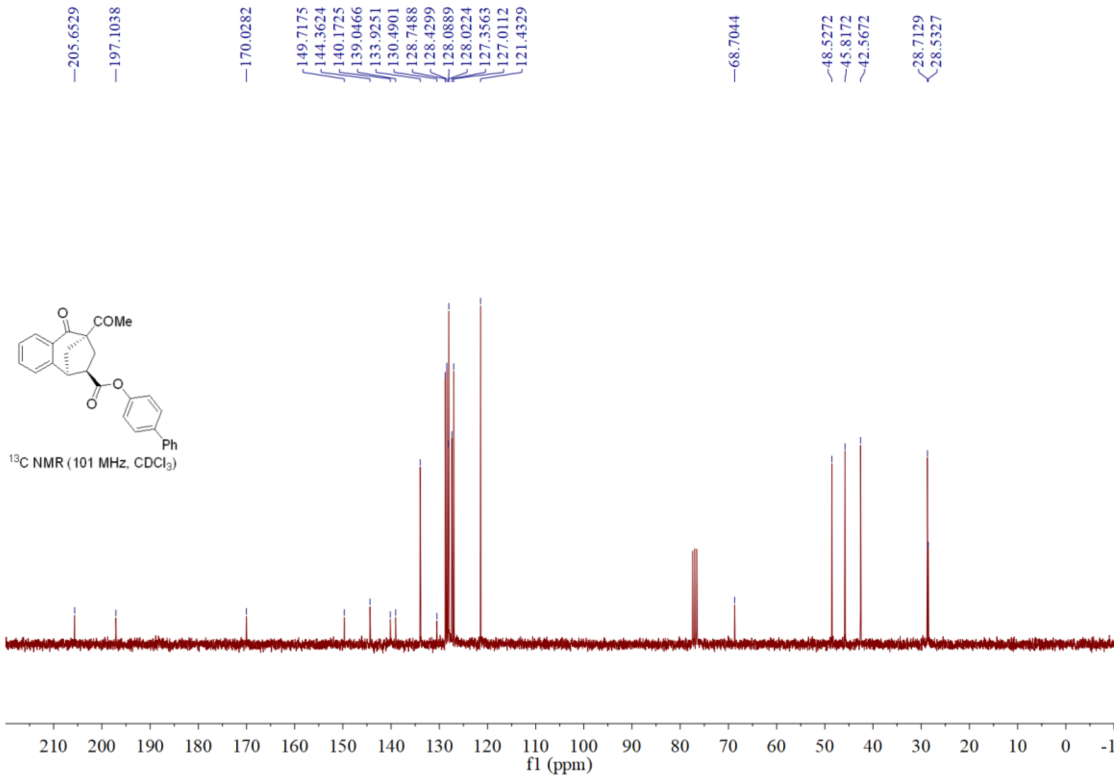

Supplementary Figure 170. ^{13}C NMR of **5ag**

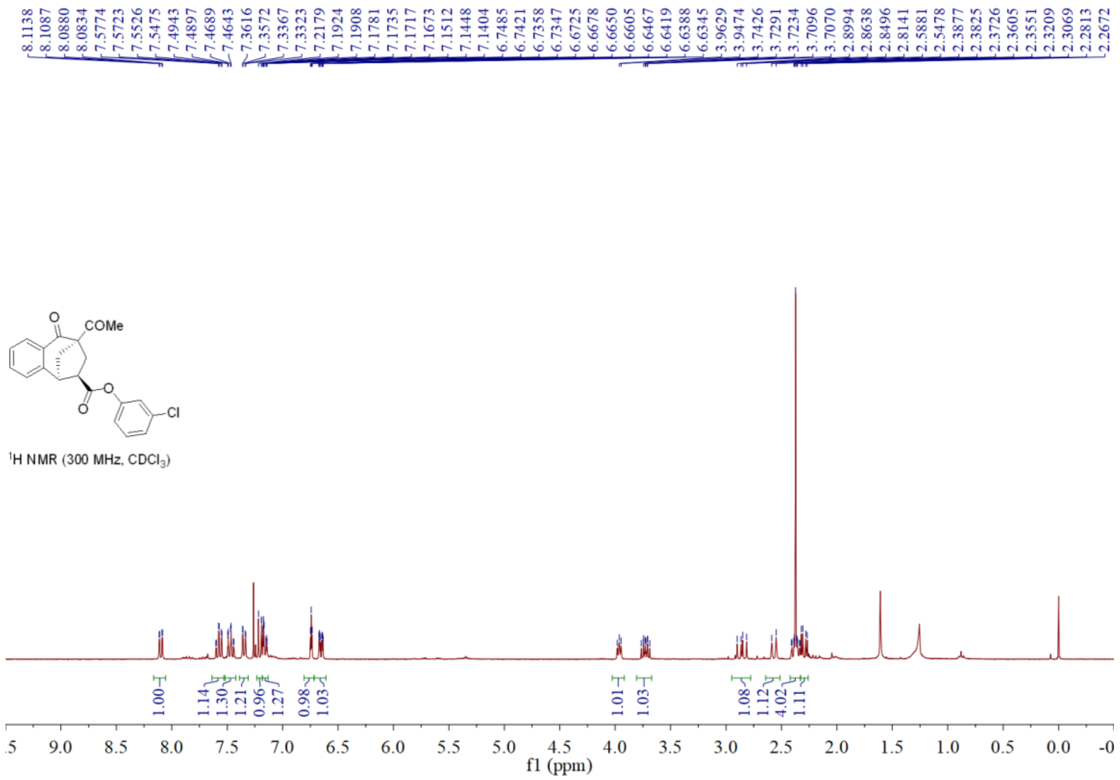

Supplementary Figure 171. ^{1}H NMR of **5ah**

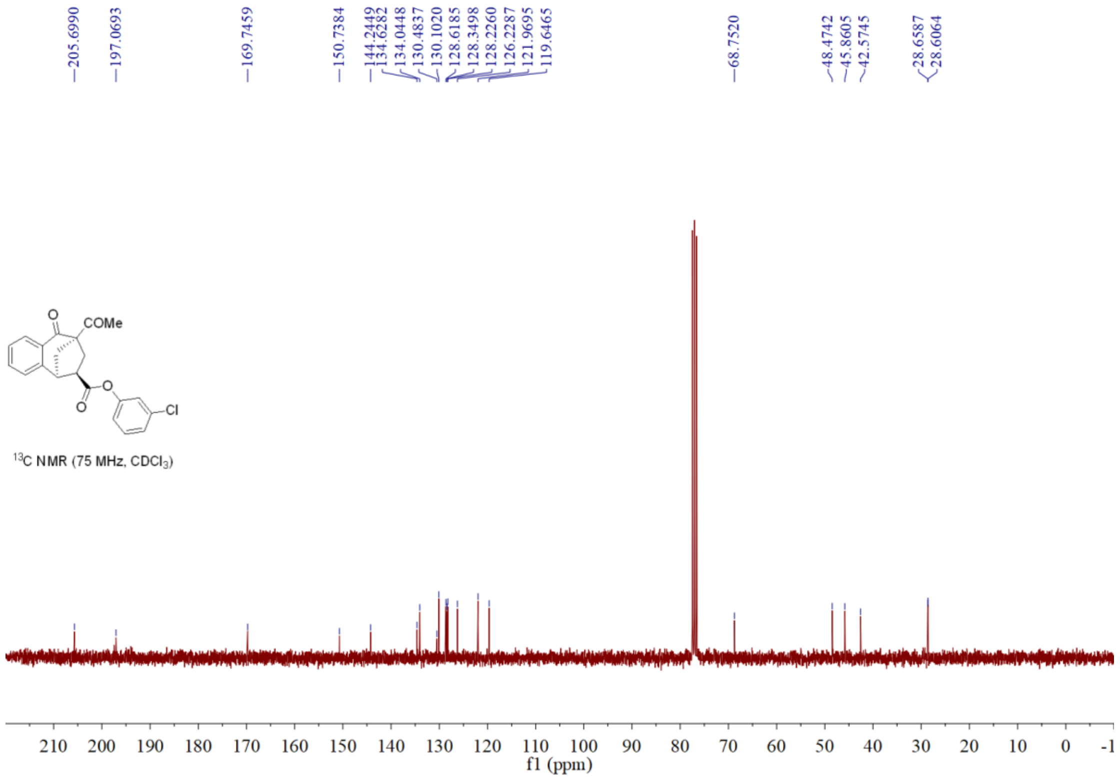

Supplementary Figure 172. ^{13}C NMR of **5ah**

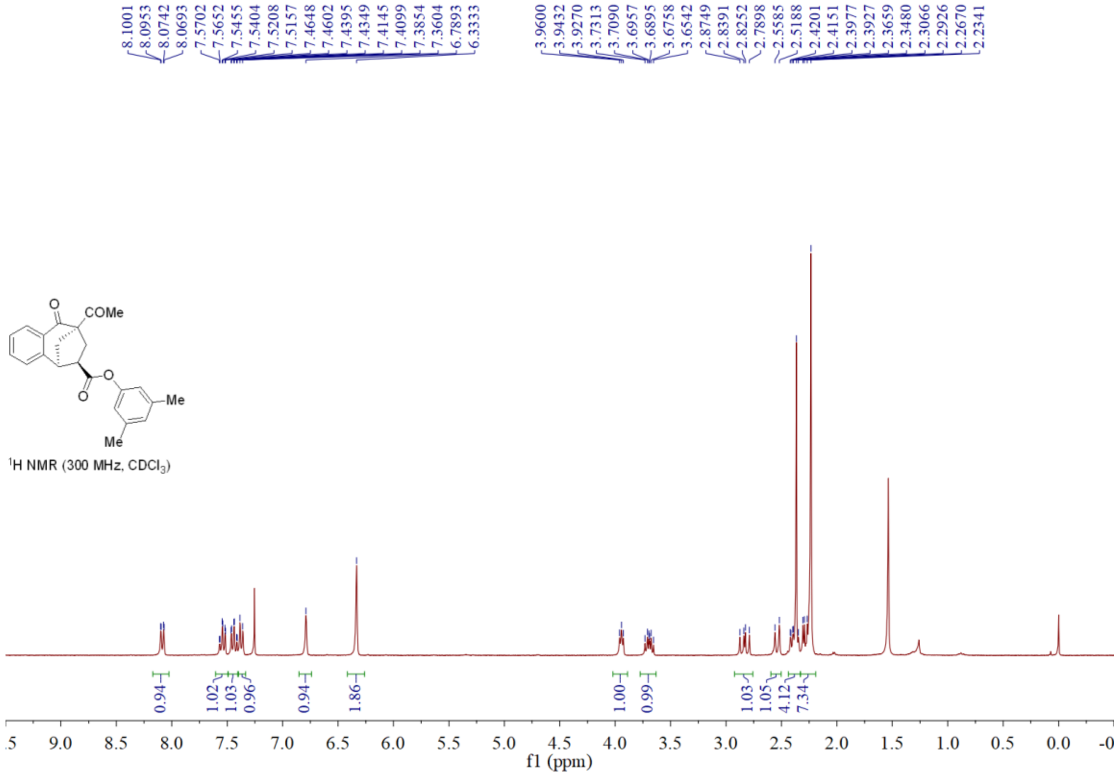

Supplementary Figure 173. ^{1}H NMR of **5ai**

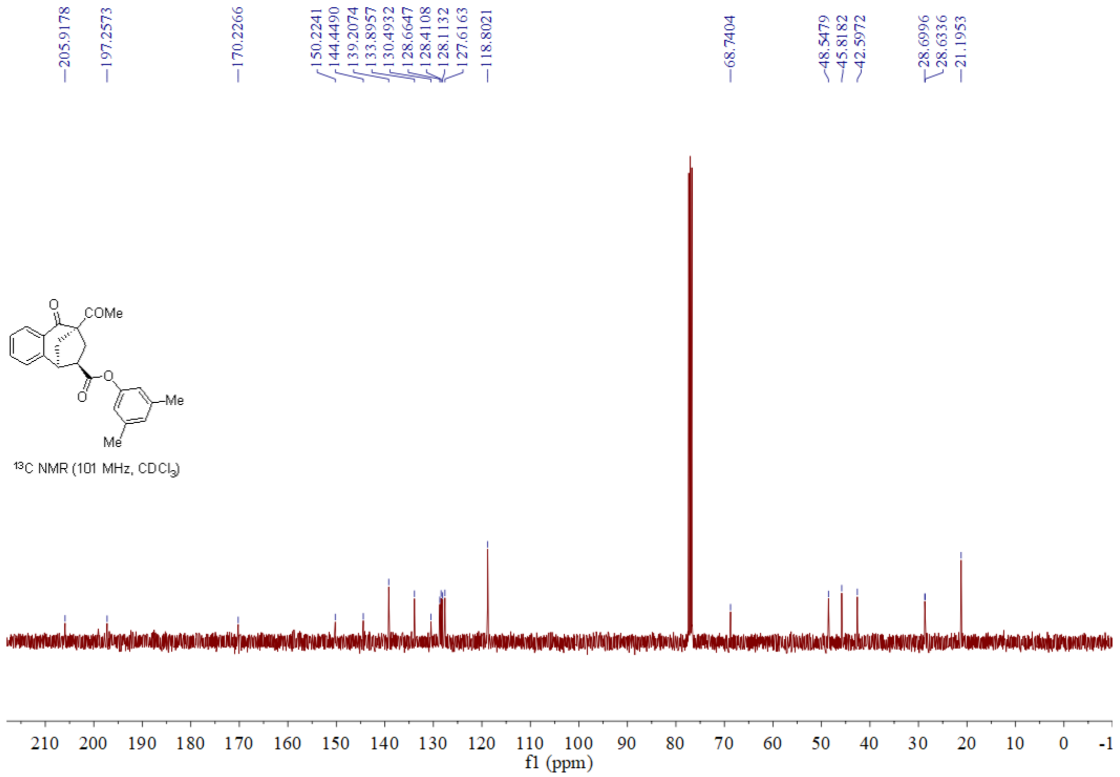

Supplementary Figure 174. ^{13}C NMR of **5ai**

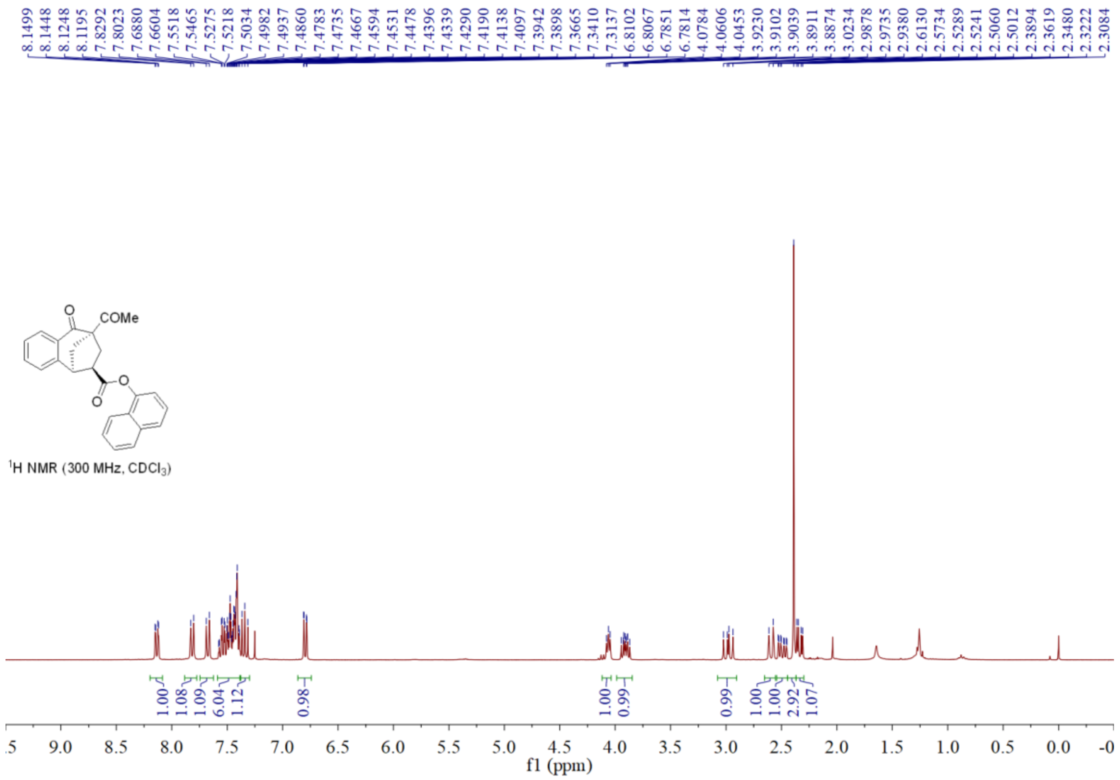

Supplementary Figure 175. ^{1}H NMR of **5aj**

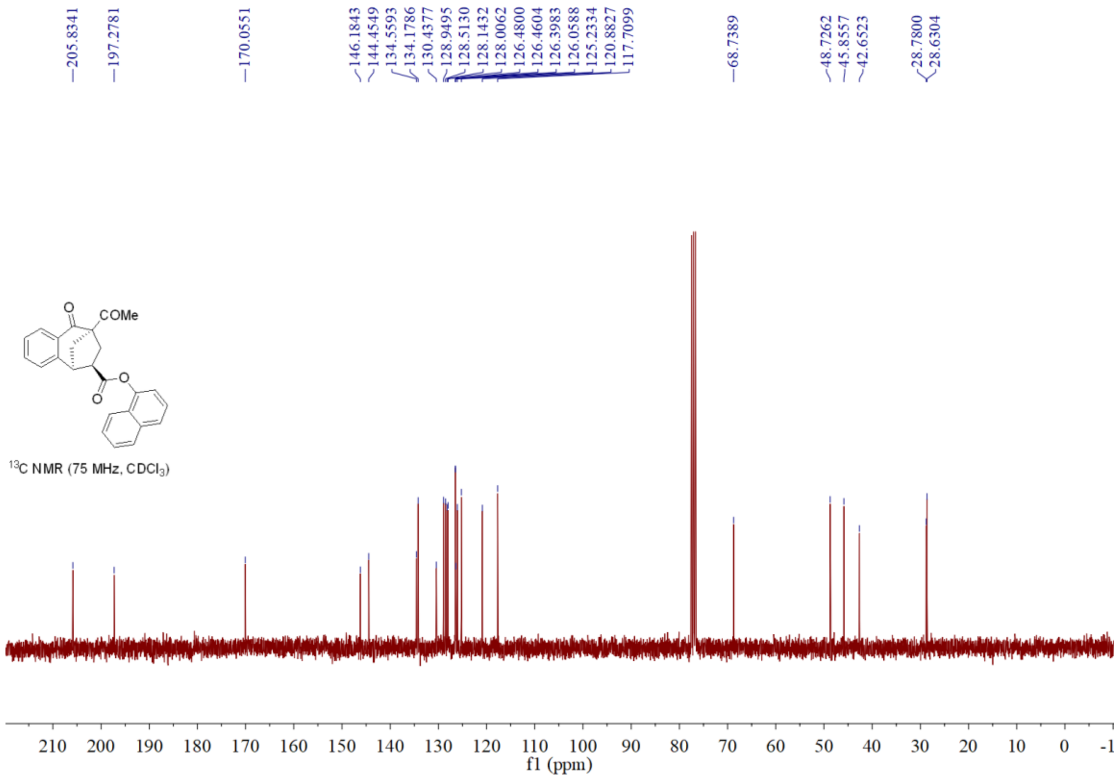

Supplementary Figure 176. ^{13}C NMR of **5aj**

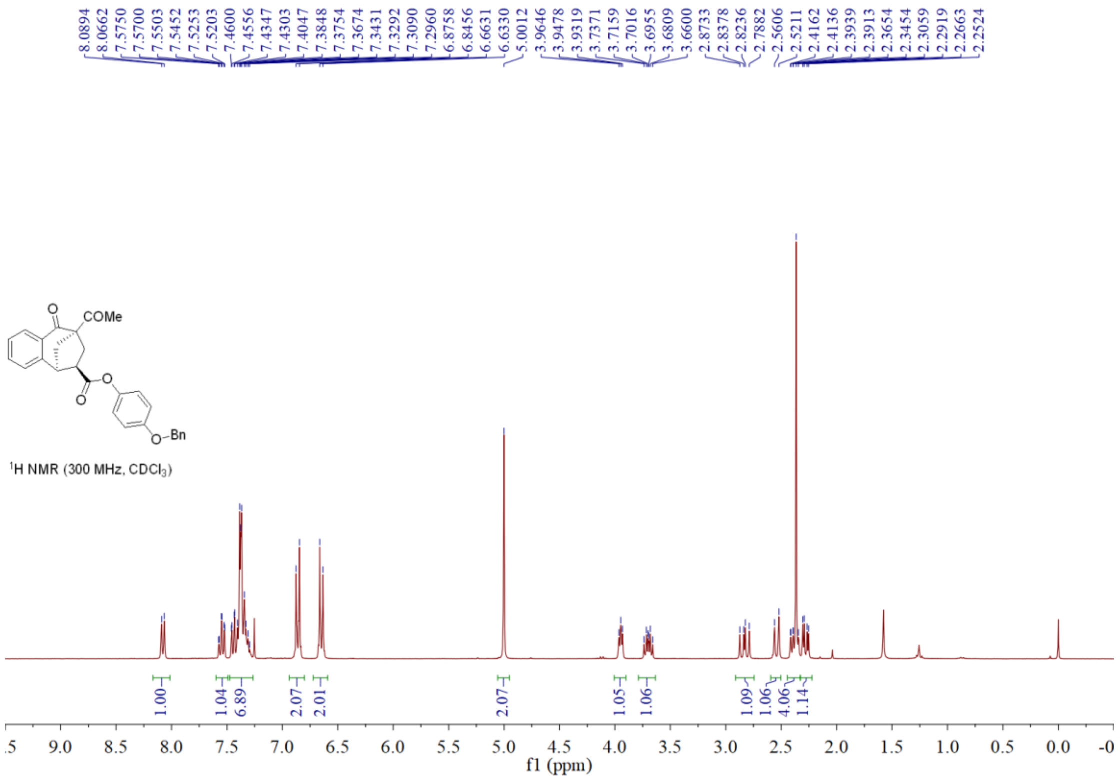

Supplementary Figure 177. ^{1}H NMR of **5ak**

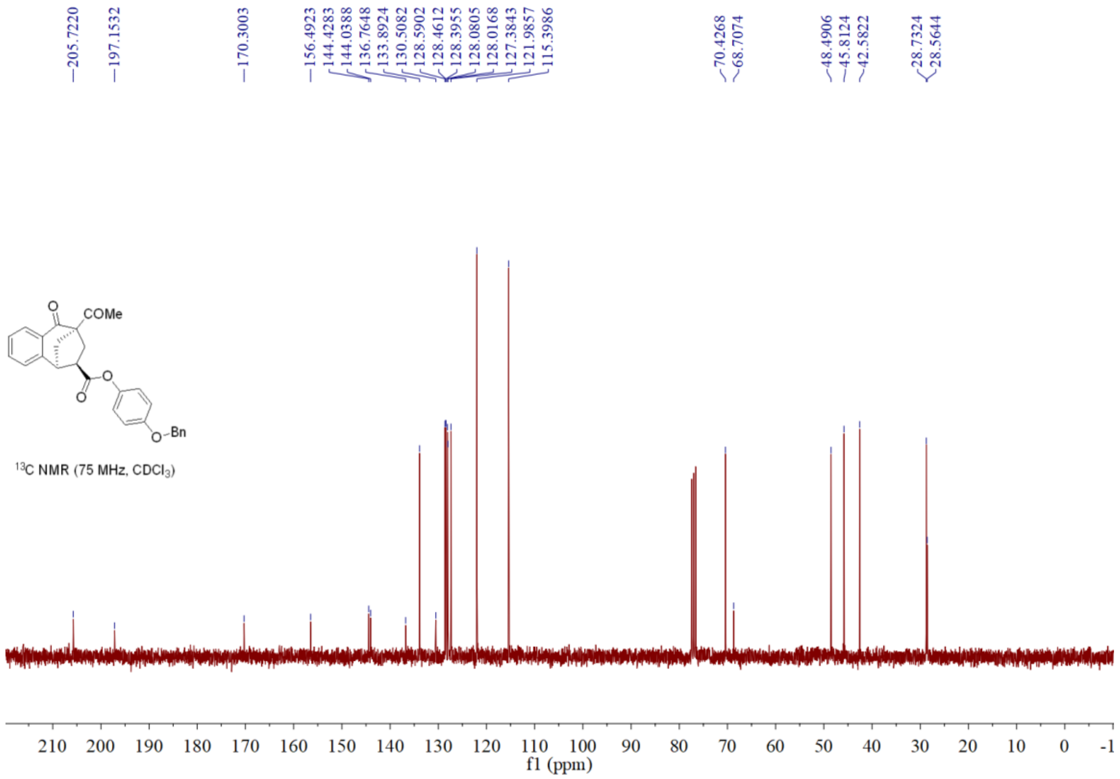

Supplementary Figure 178. ^{13}C NMR of **5ak**

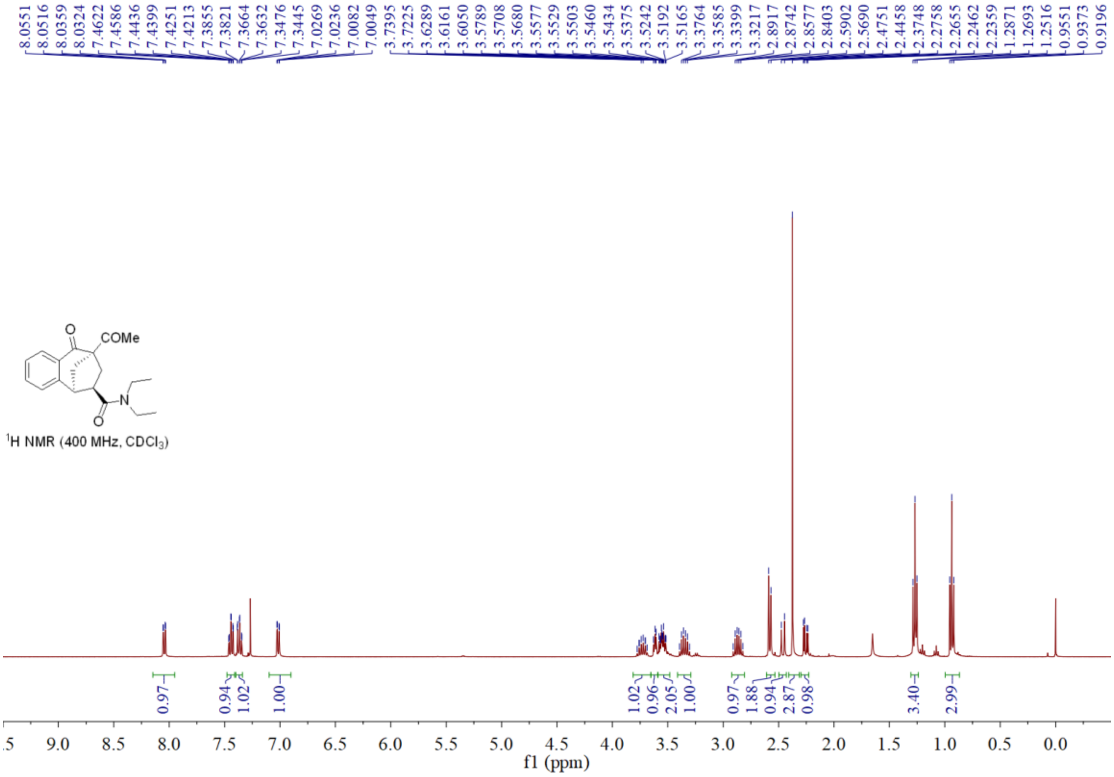

Supplementary Figure 179. ^{1}H NMR of **7aa**

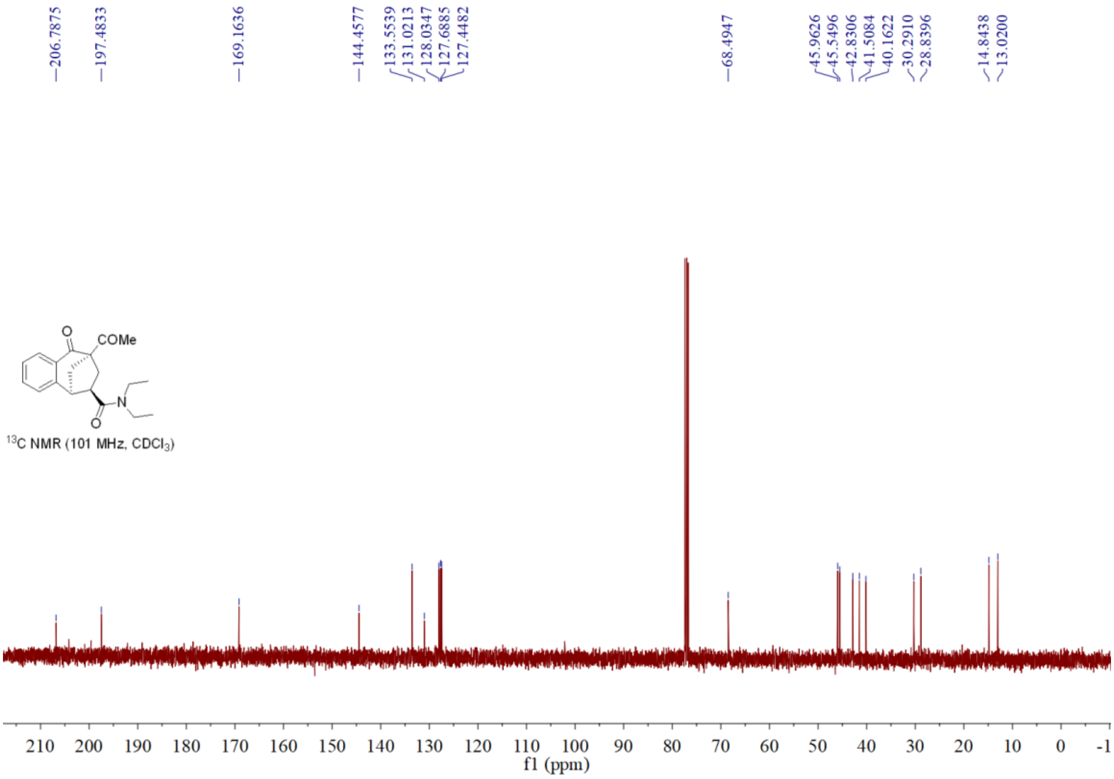

Supplementary Figure 180. ^{13}C NMR of **7aa**

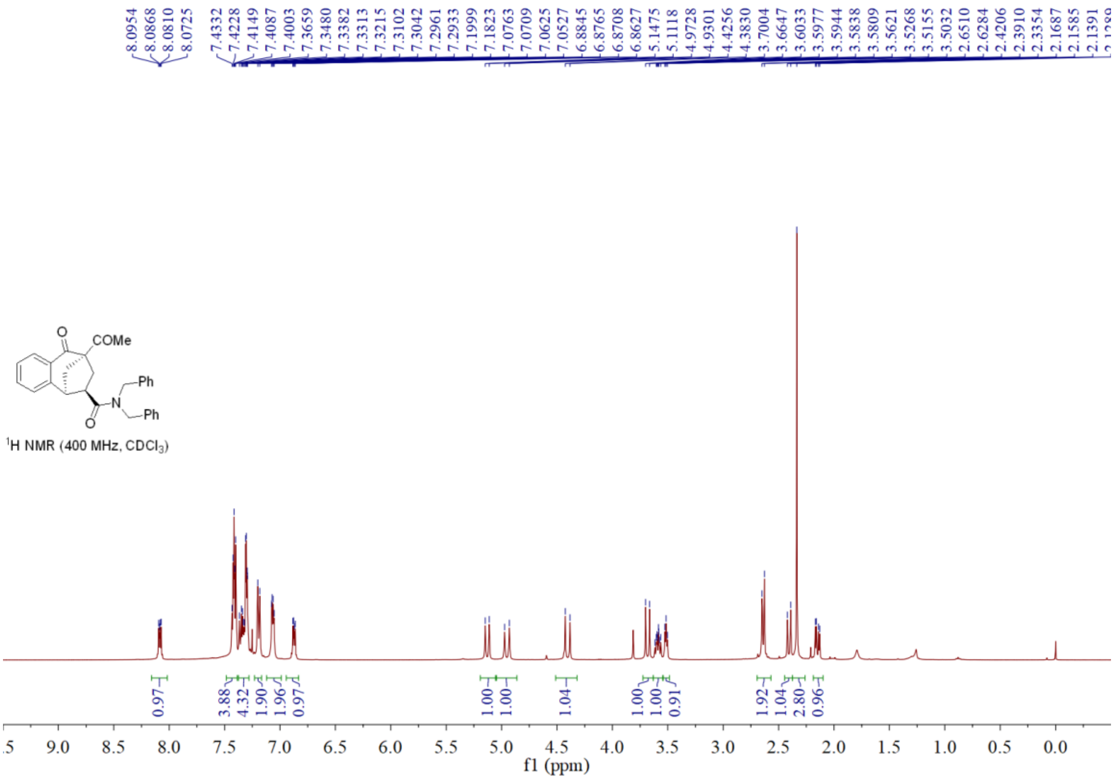

Supplementary Figure 181. ^{1}H NMR of **7ab**

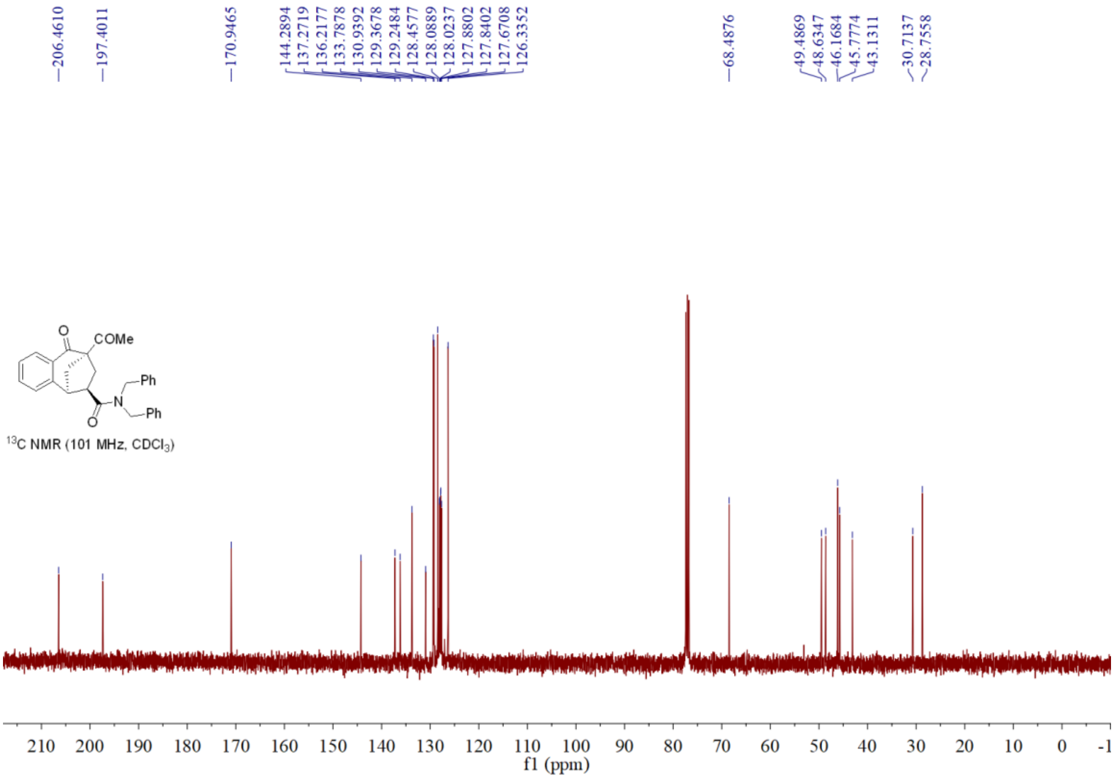

Supplementary Figure 182. ^{13}C NMR of **7ab**

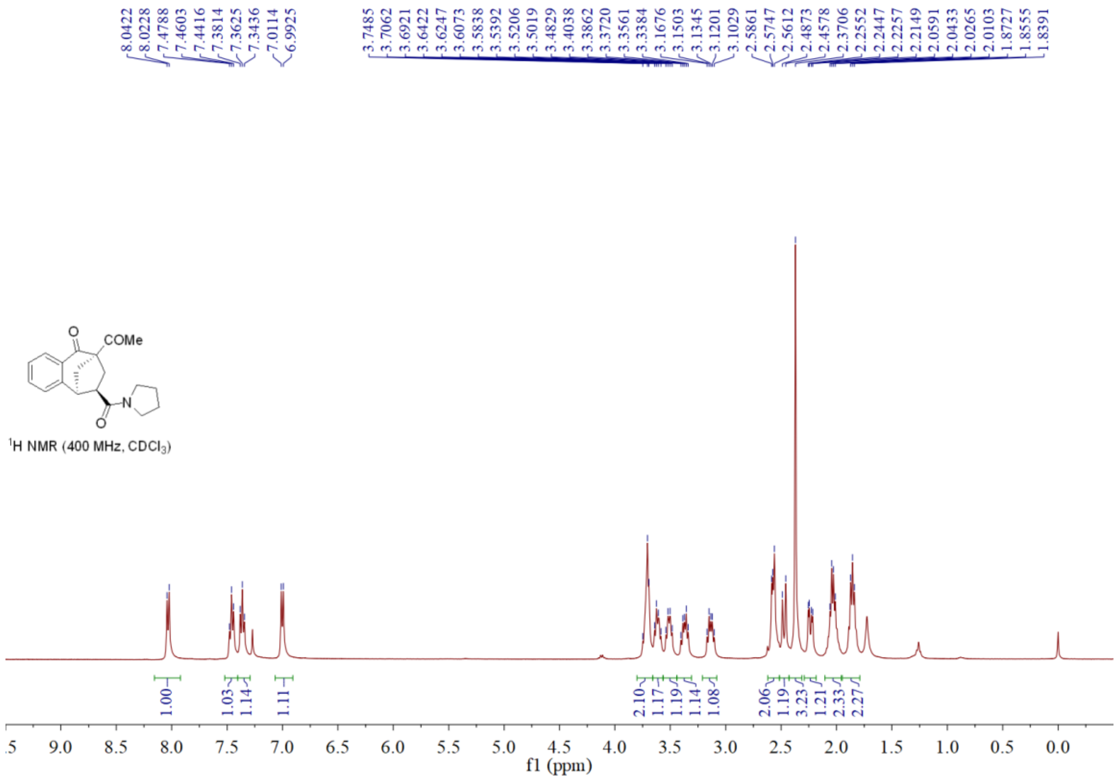

Supplementary Figure 183. ^{1}H NMR of **7ac**

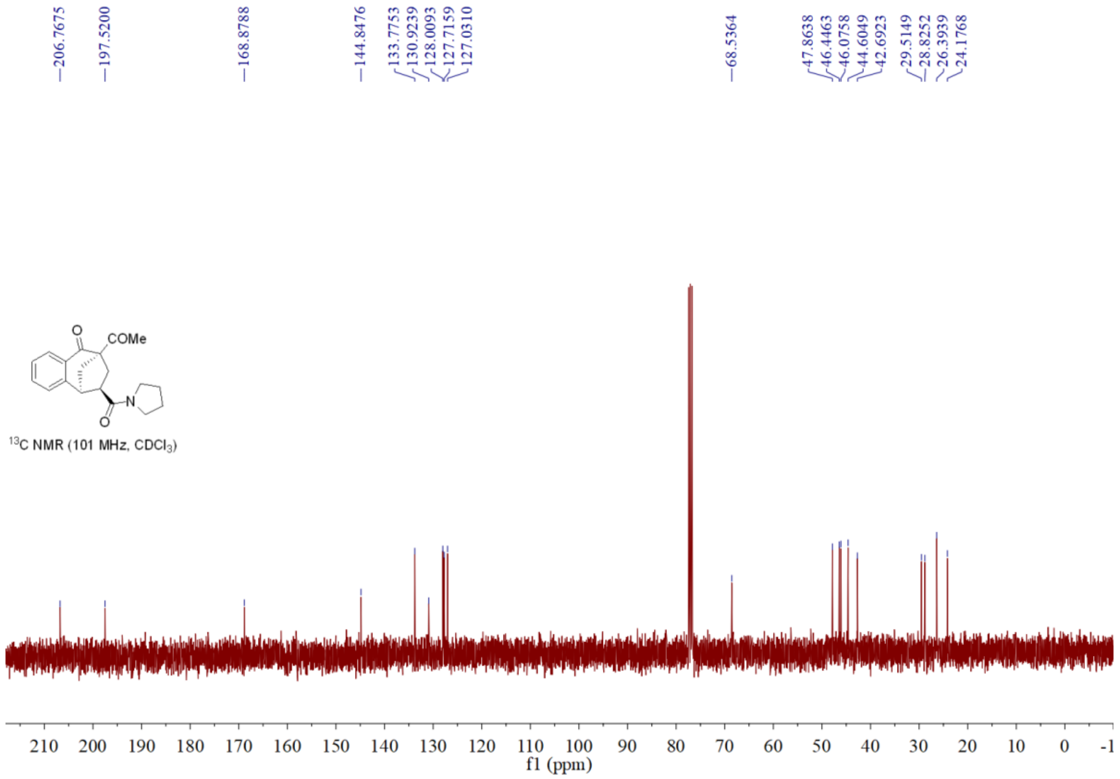

Supplementary Figure 184. ^{13}C NMR of **7ac**

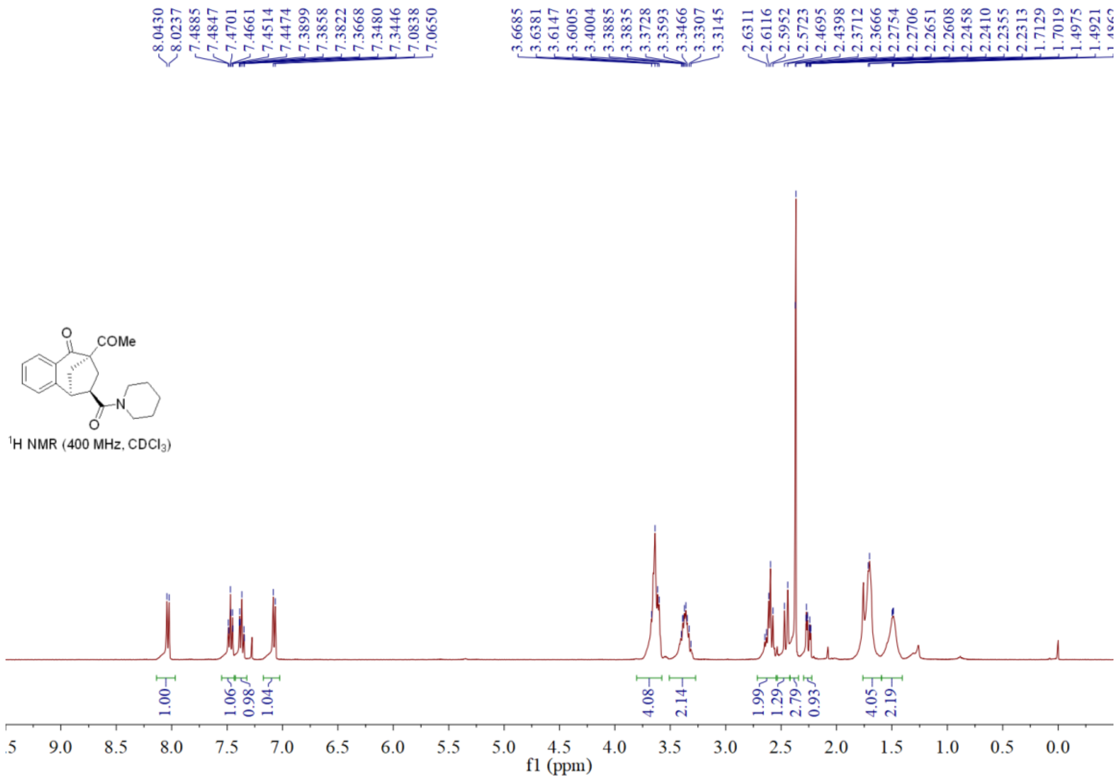

Supplementary Figure 185. ^{1}H NMR of **7ad**

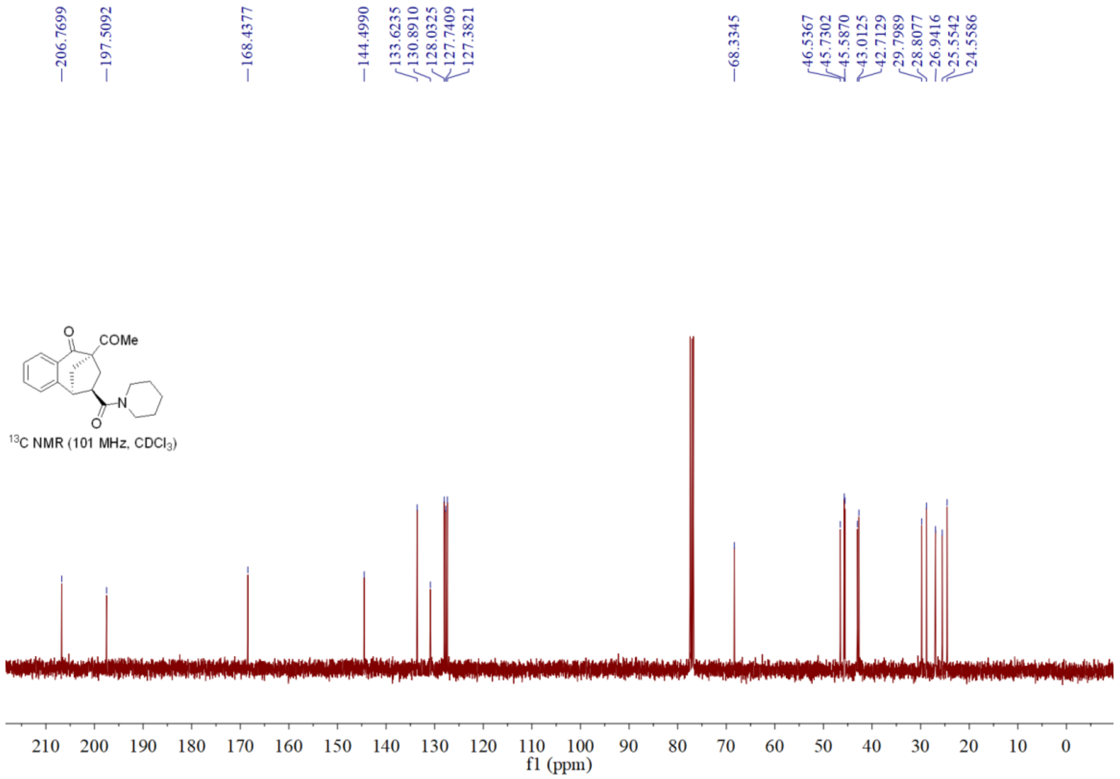

Supplementary Figure 186. ^{13}C NMR of **7ad**

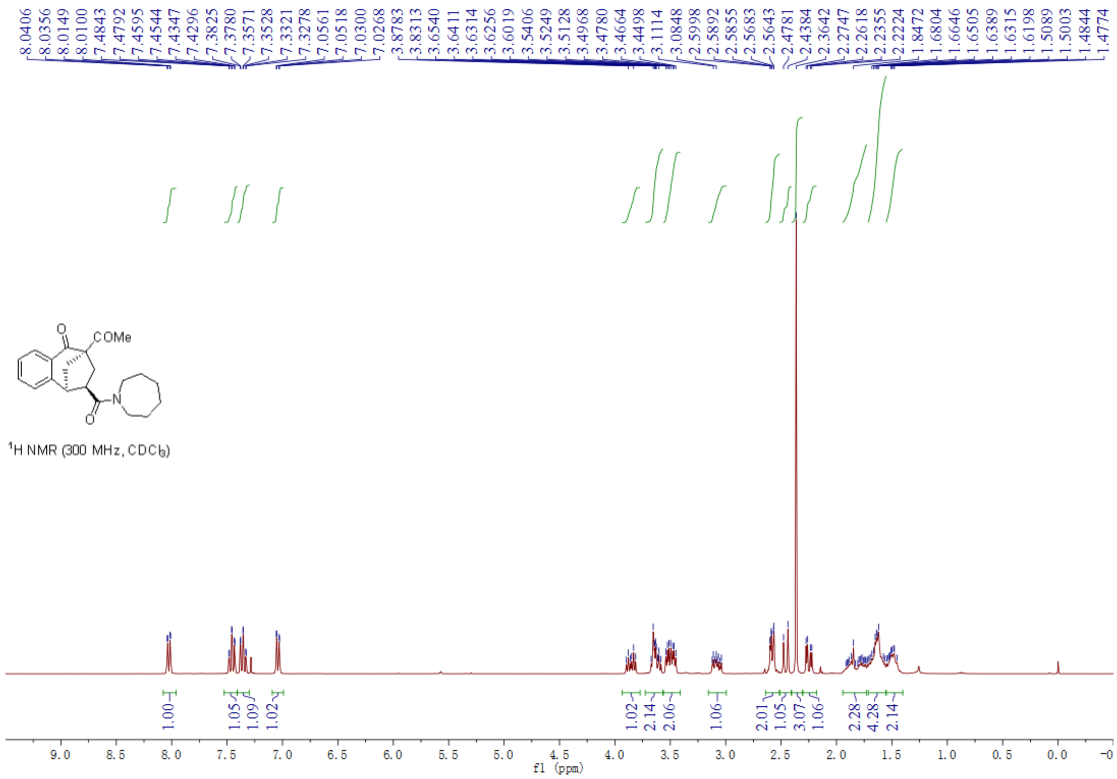

Supplementary Figure 187. ^{1}H NMR of **7ae**

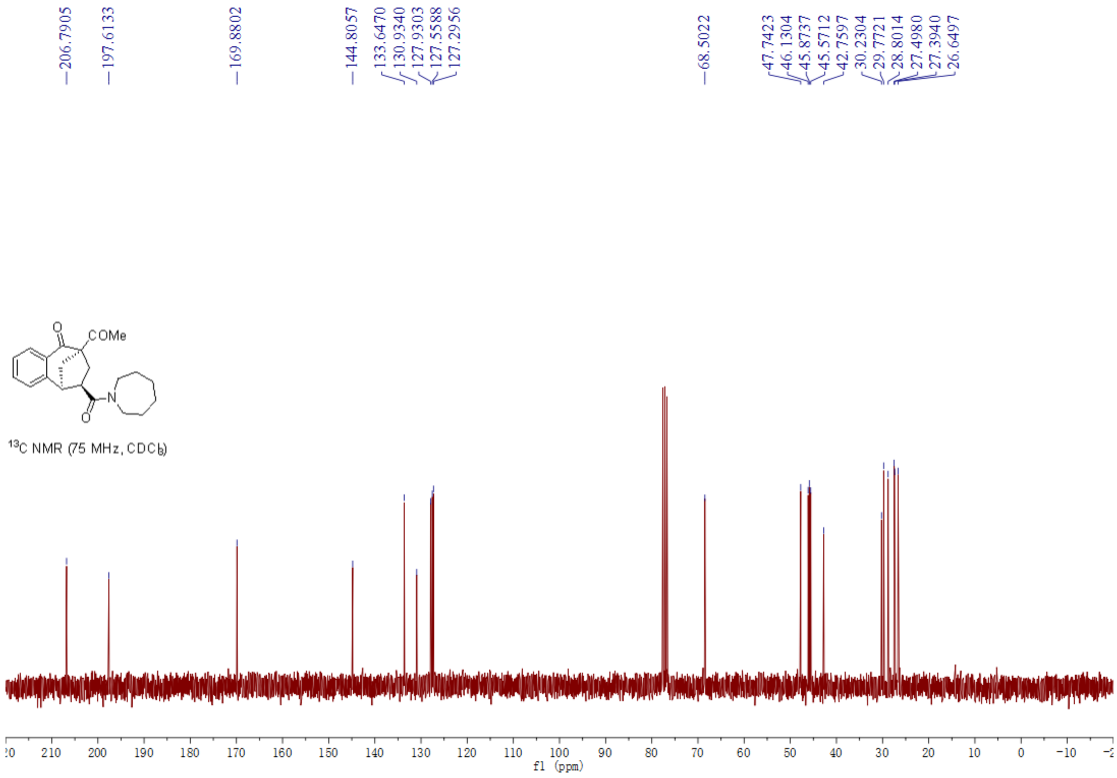

Supplementary Figure 188. ^{13}C NMR of **7ae**

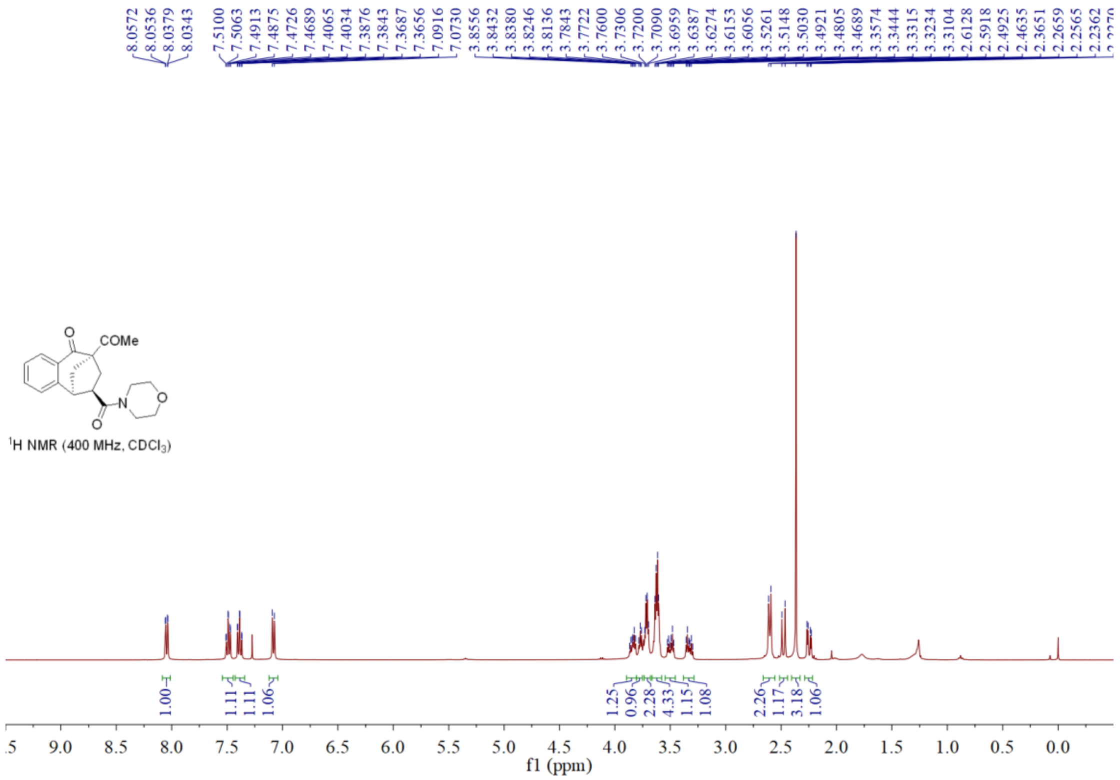

Supplementary Figure 189. ^{1}H NMR of **7af**

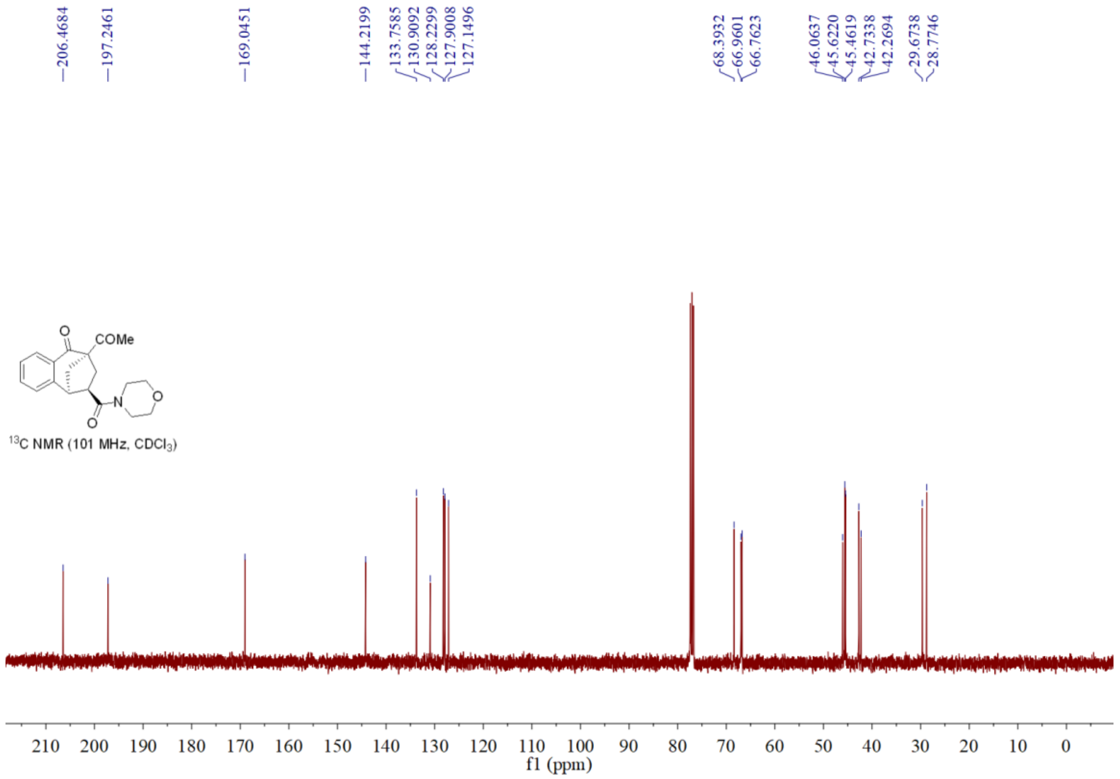

Supplementary Figure 190. ^{13}C NMR of **7af**

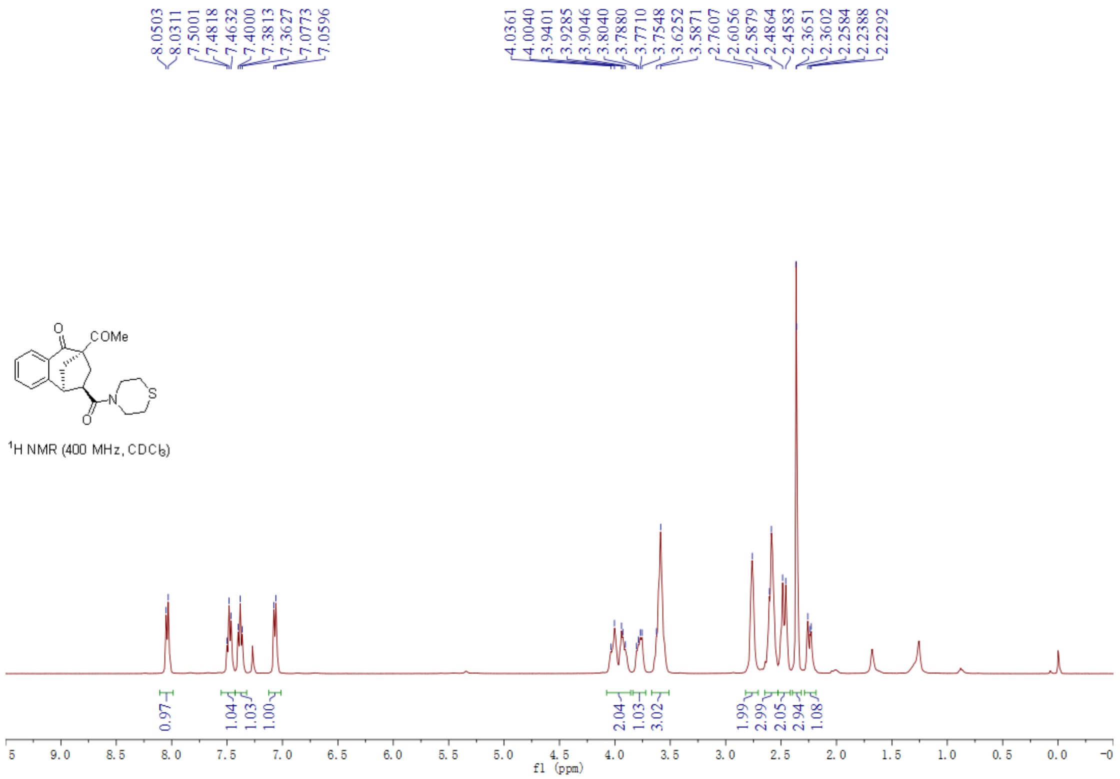

Supplementary Figure 191. ^{1}H NMR of **7ag**

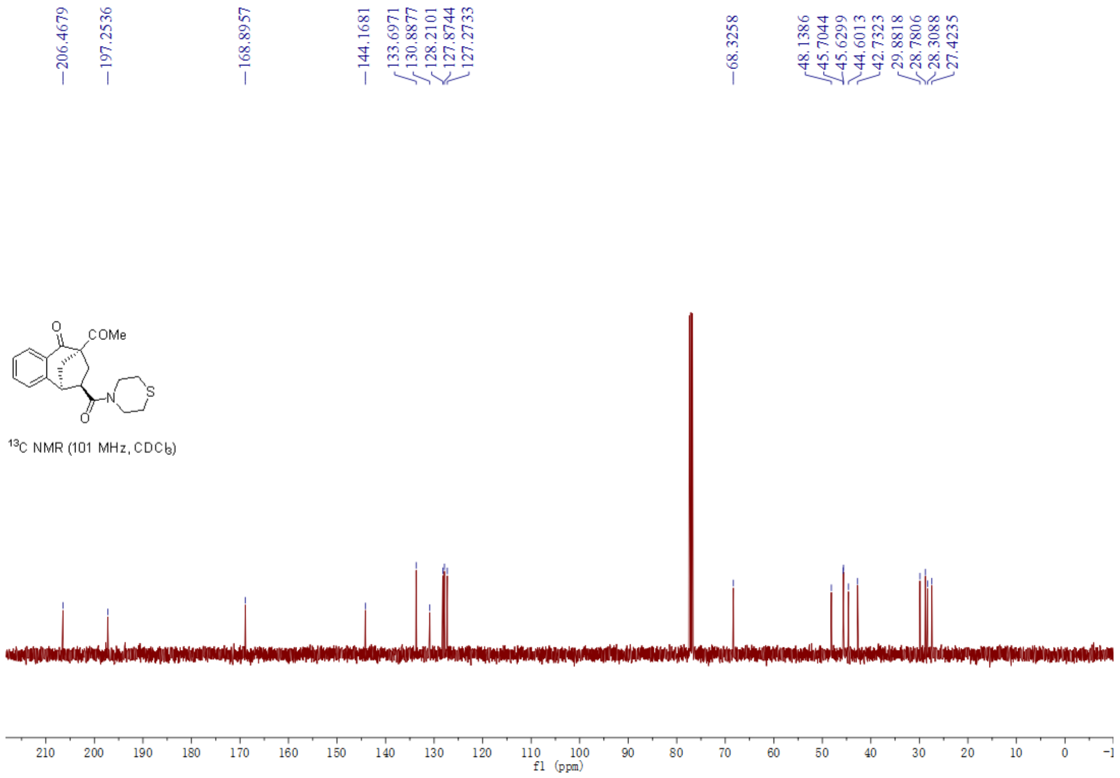

Supplementary Figure 192. ^{13}C NMR of **7ag**

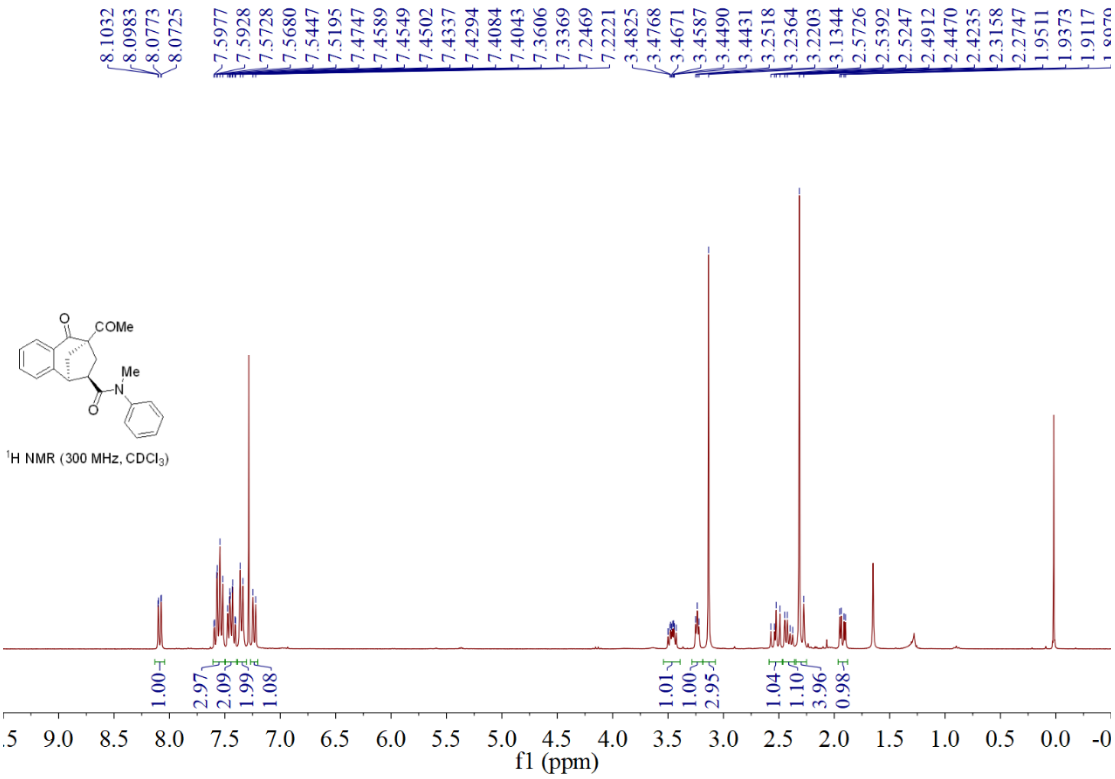

Supplementary Figure 193. ^{1}H NMR of **7ah**

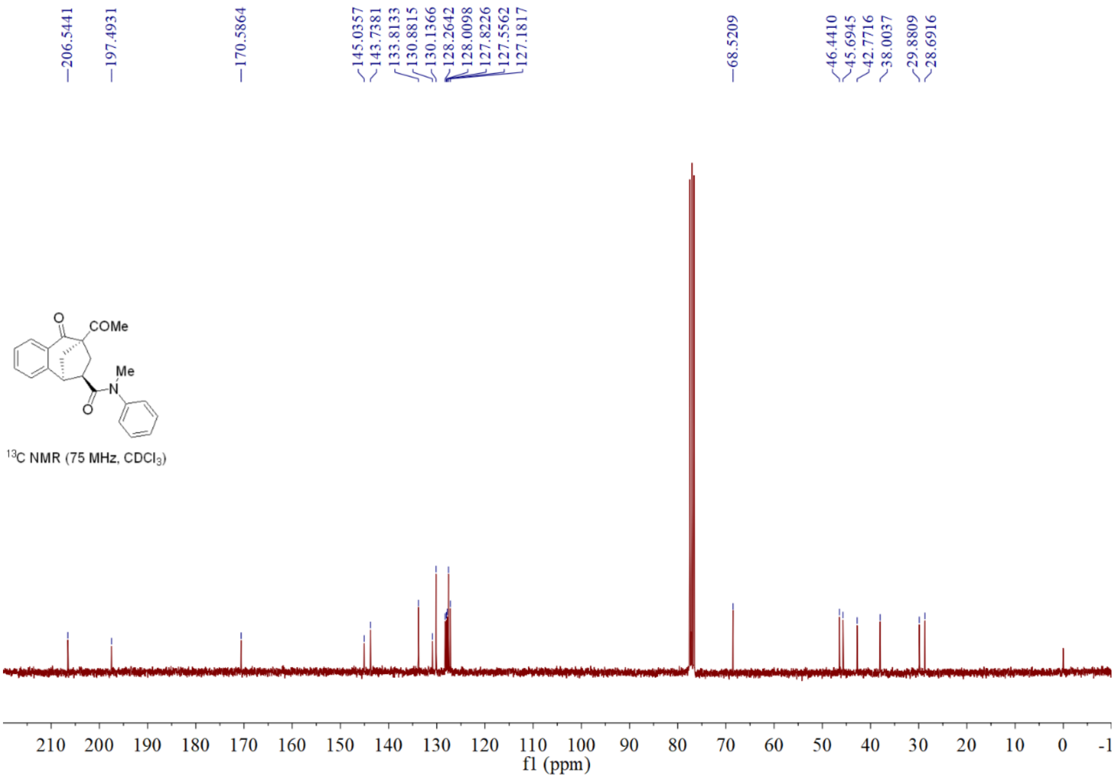

Supplementary Figure 194. ^{13}C NMR of **7ah**

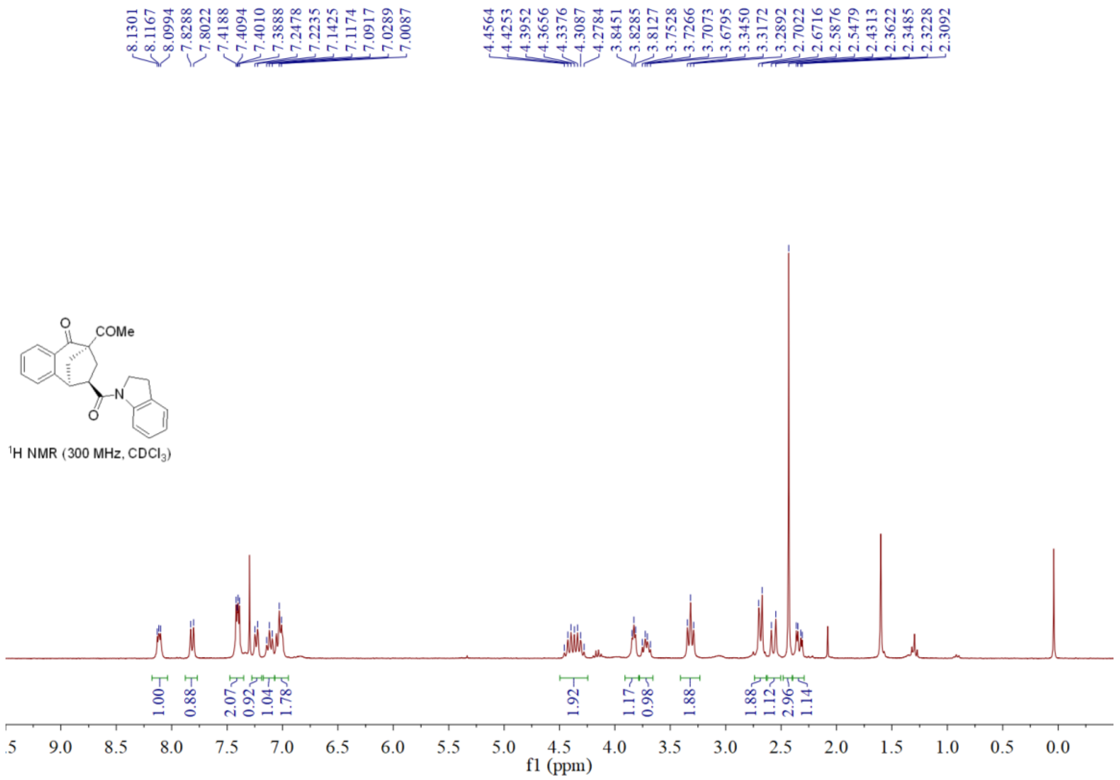

Supplementary Figure 195. ^{1}H NMR of **7ai**

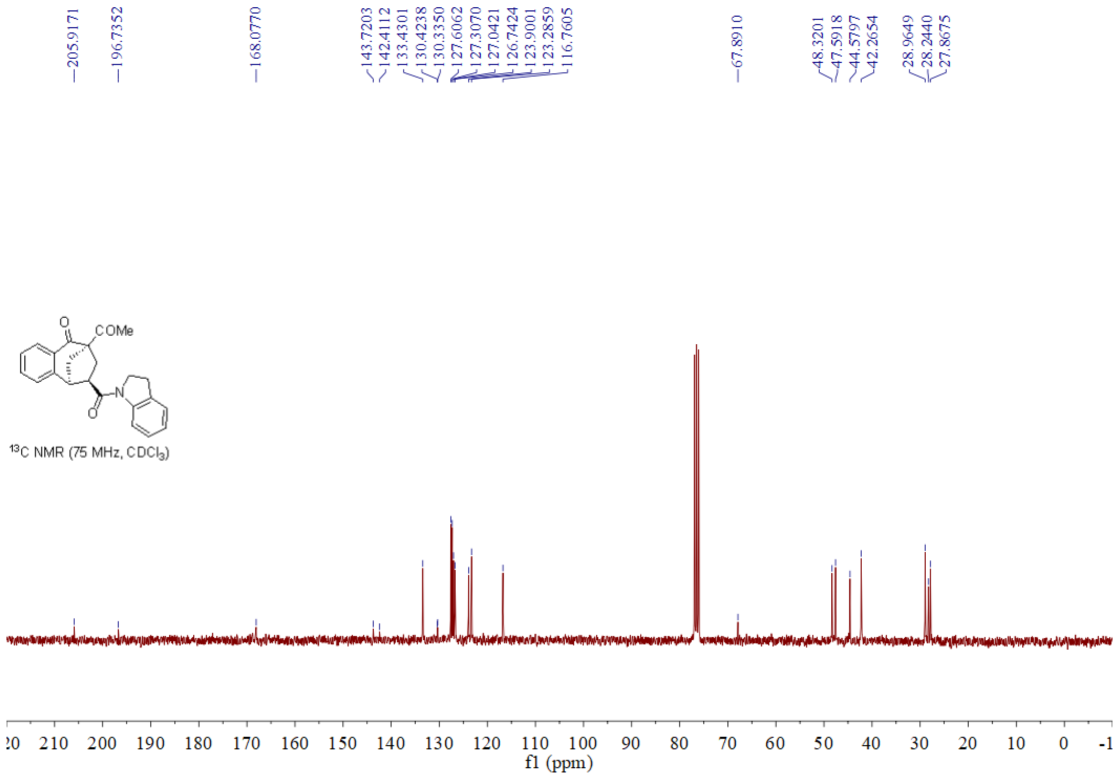

Supplementary Figure 196. ^{13}C NMR of **7ai**

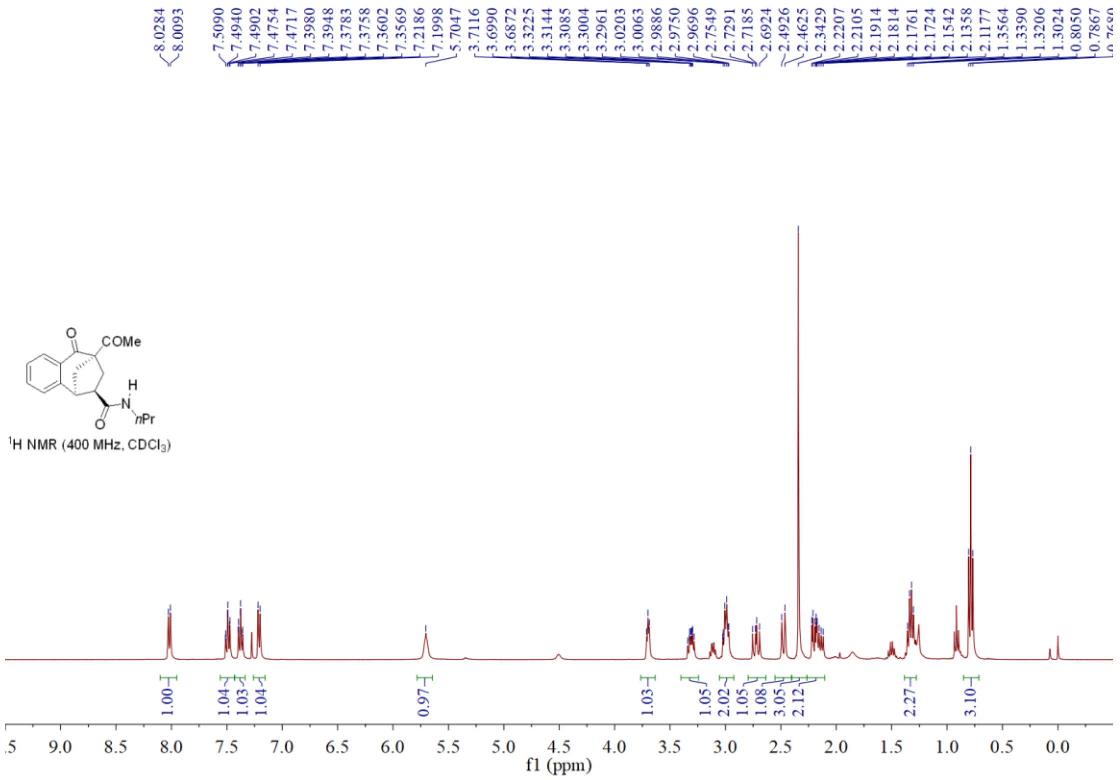

Supplementary Figure 197. ^{1}H NMR of **7aj**

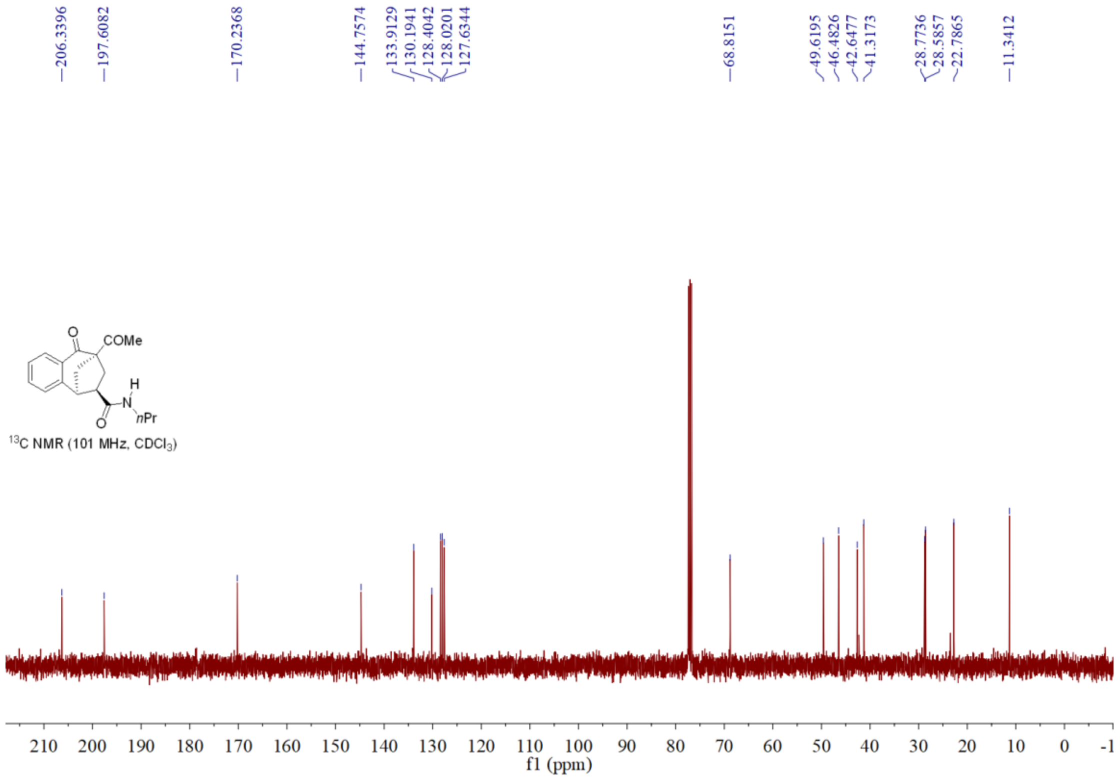

Supplementary Figure 198. ^{13}C NMR of **7aj**

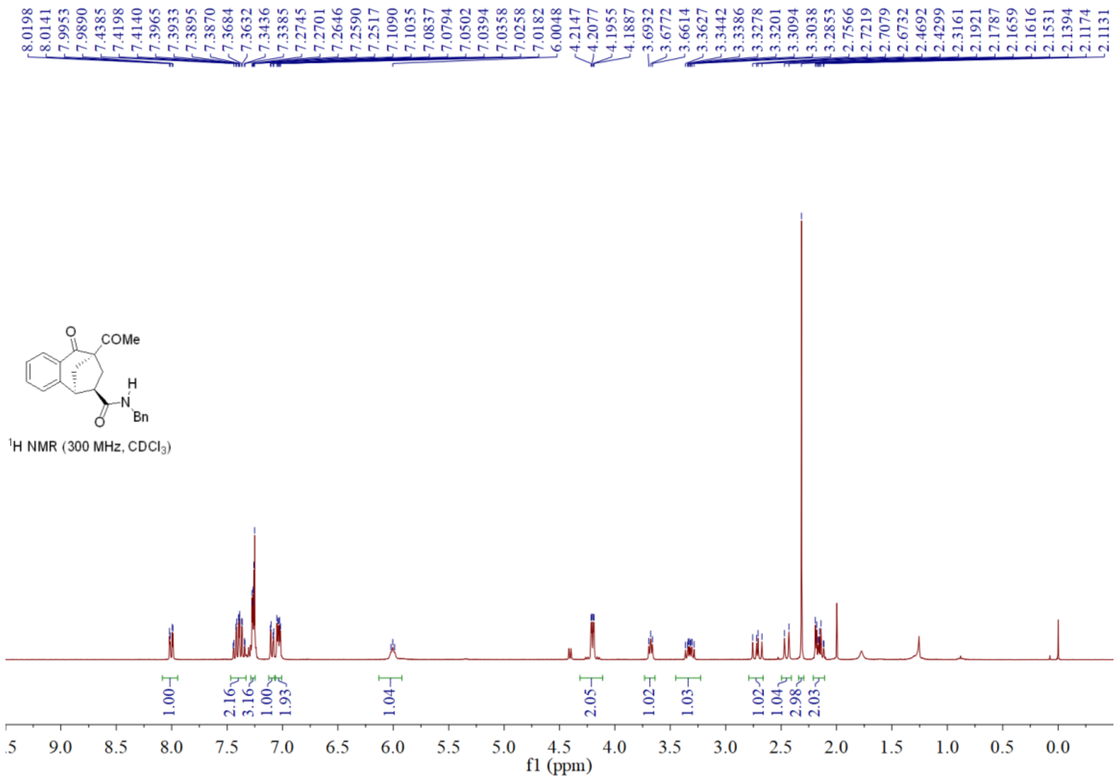

Supplementary Figure 199. ^{1}H NMR of **7ak**

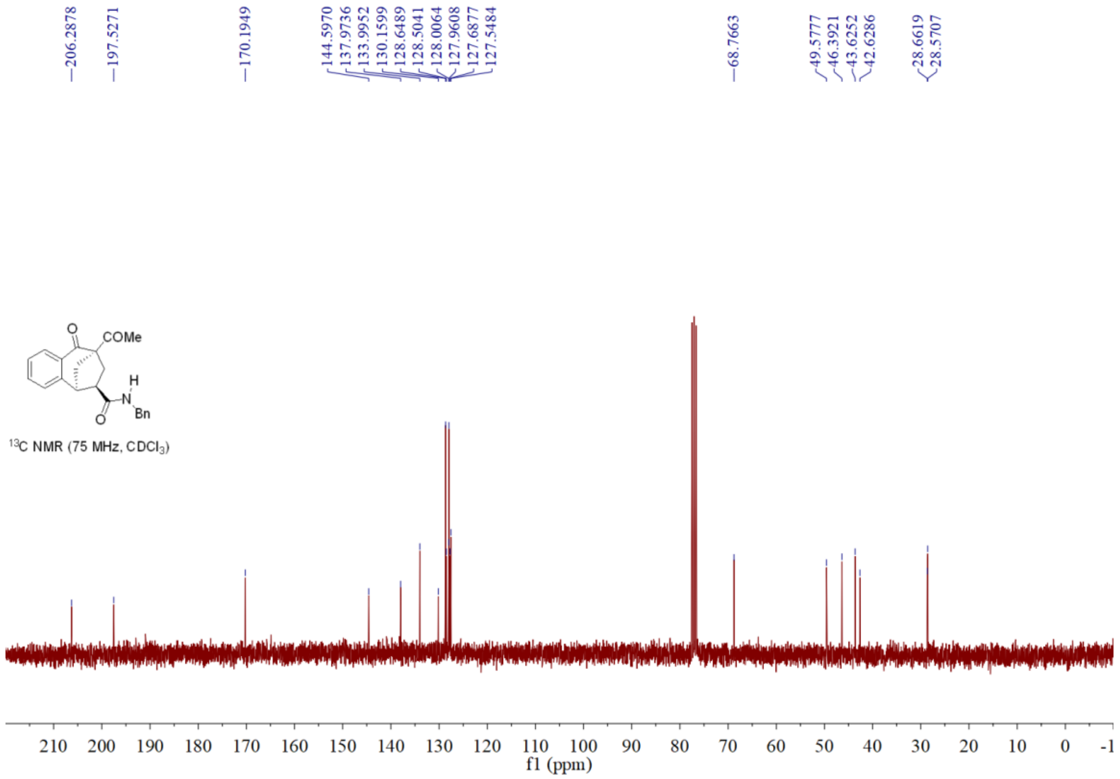

Supplementary Figure 200. ^{13}C NMR of **7ak**

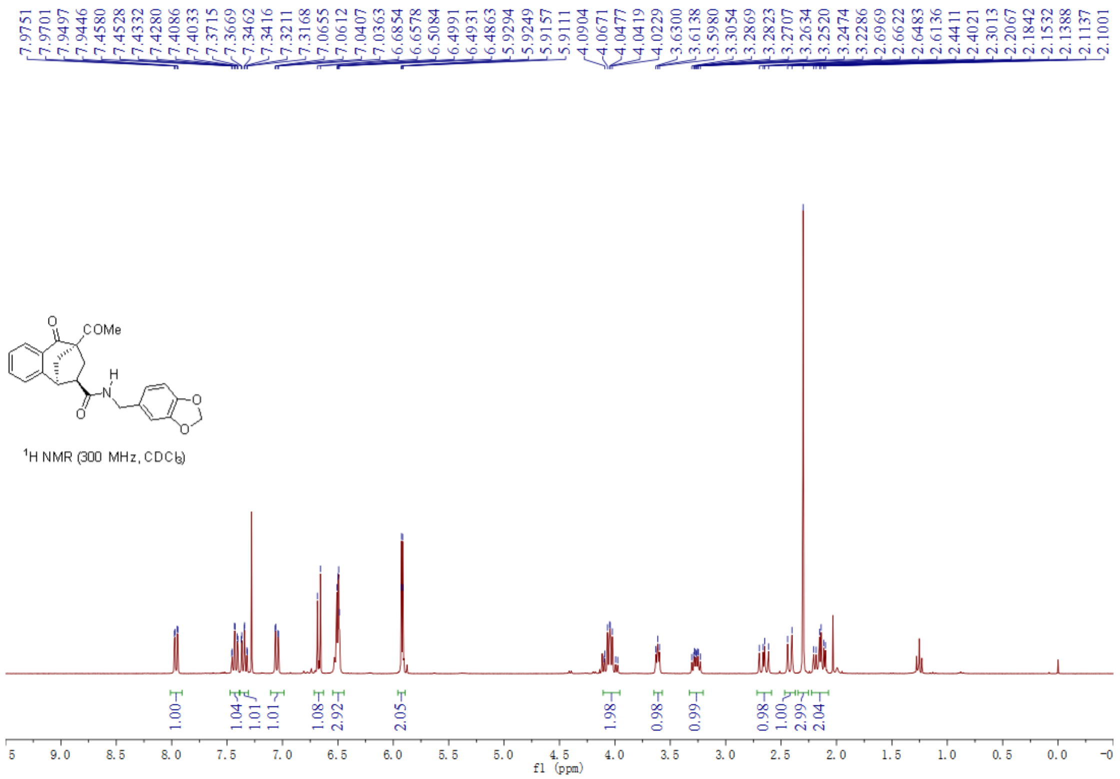

Supplementary Figure 201. ^{1}H NMR of **7al**

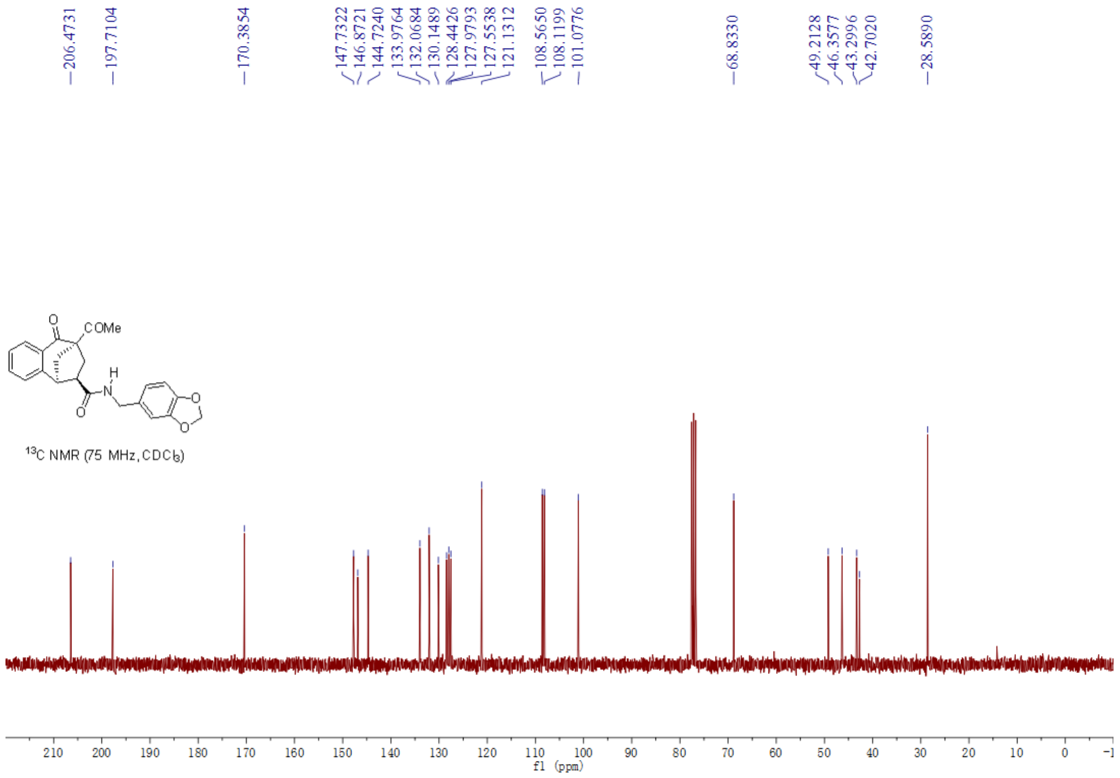

Supplementary Figure 202. ^{13}C NMR of **7al**

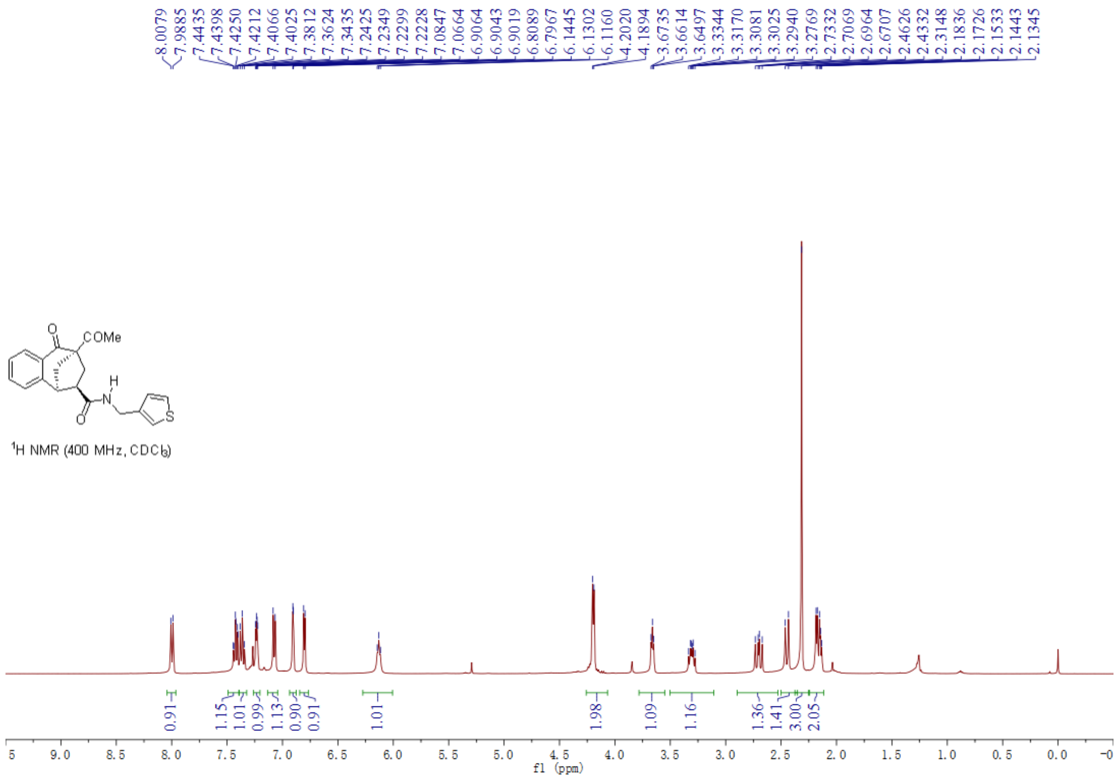

Supplementary Figure 203. ^{1}H NMR of **7am**

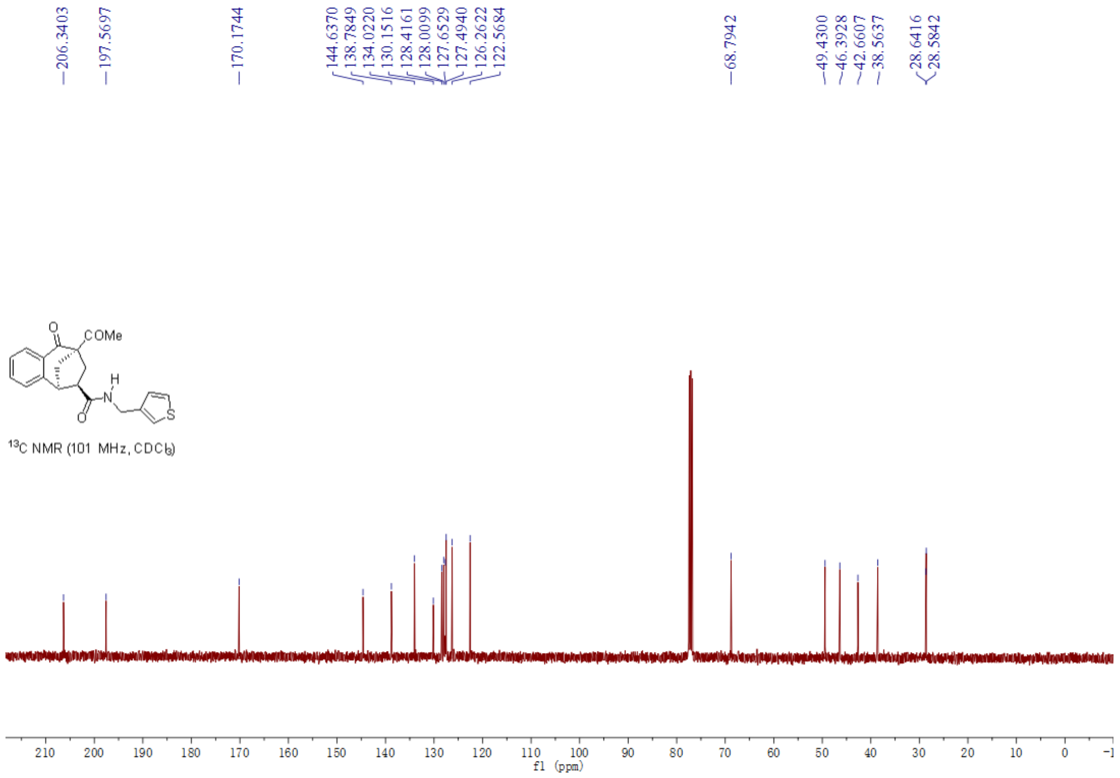

Supplementary Figure 204. ^{13}C NMR of **7am**

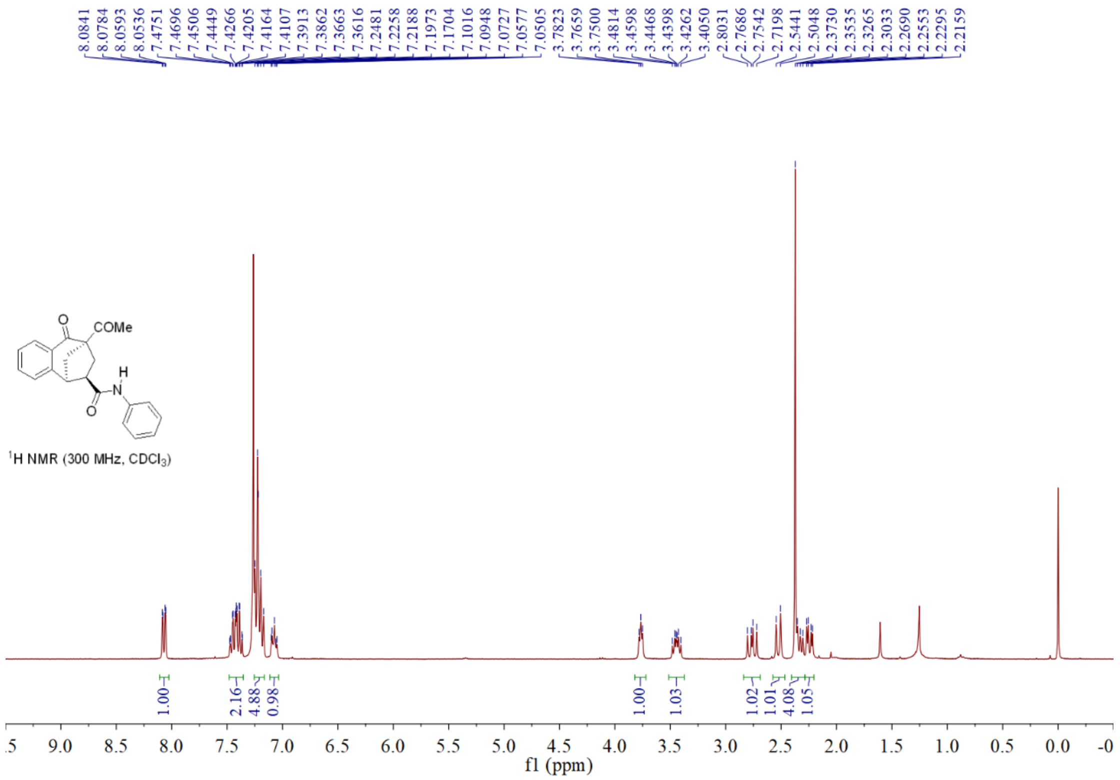

Supplementary Figure 205. ^{1}H NMR of **7an**

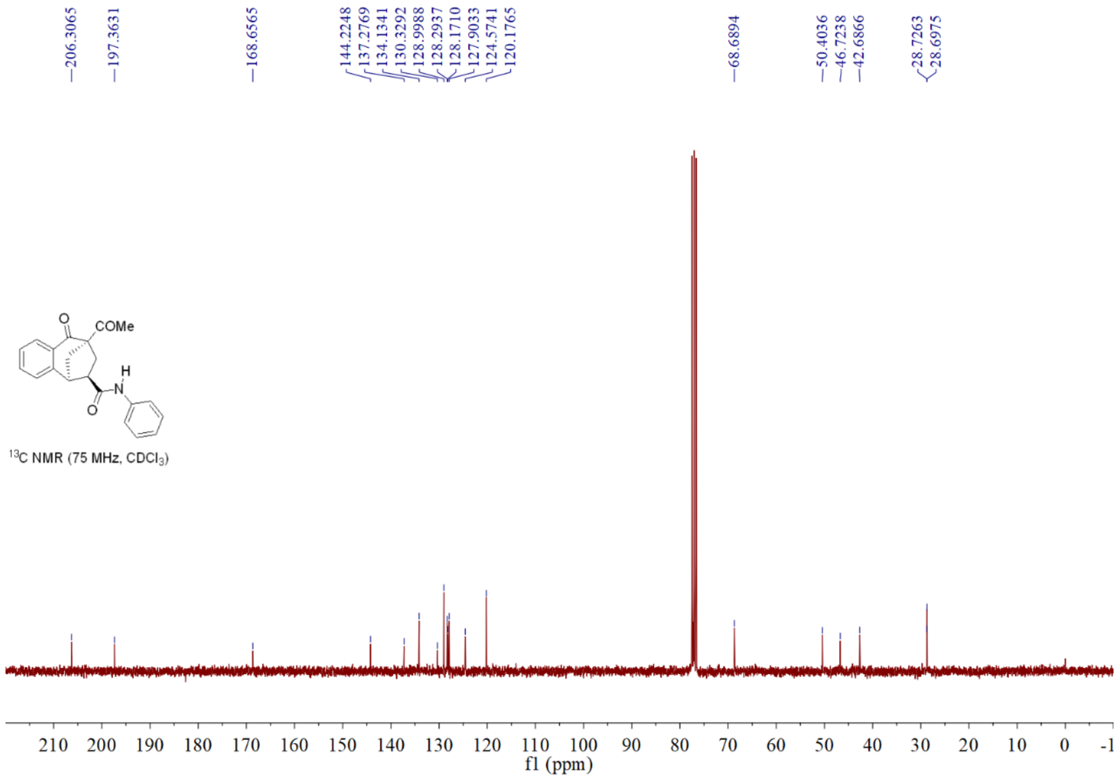

Supplementary Figure 206. ^{13}C NMR of **7an**

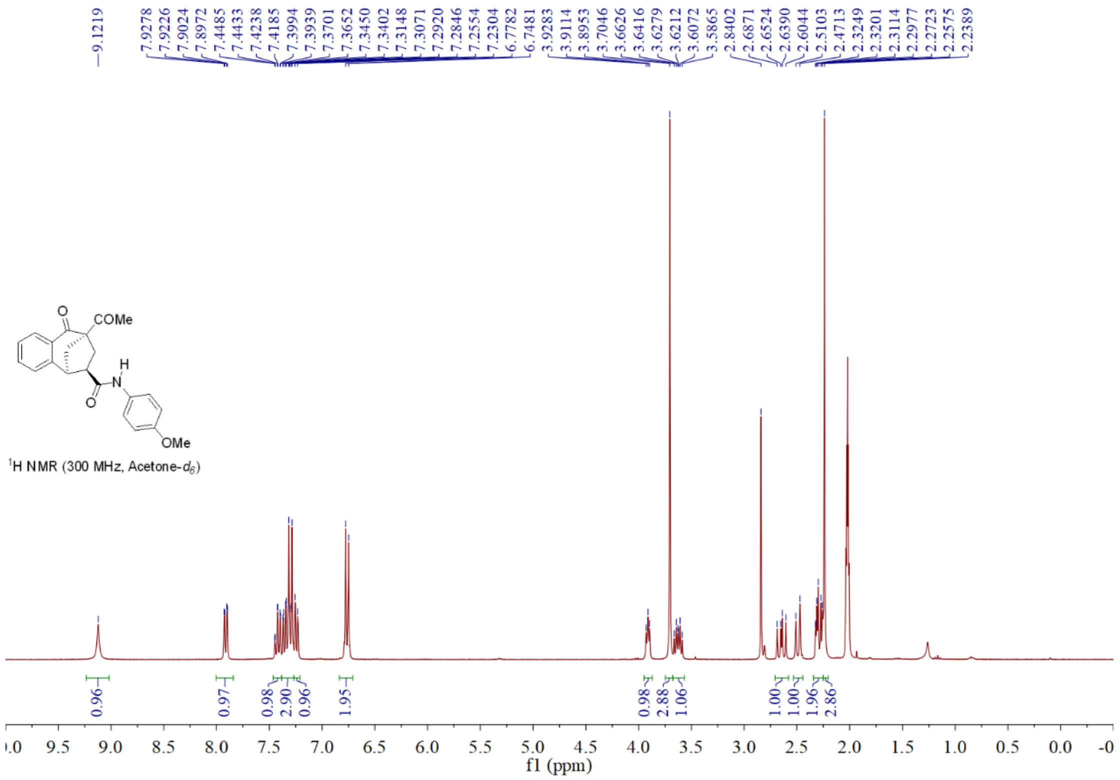

Supplementary Figure 207. ^{1}H NMR of **7ao**

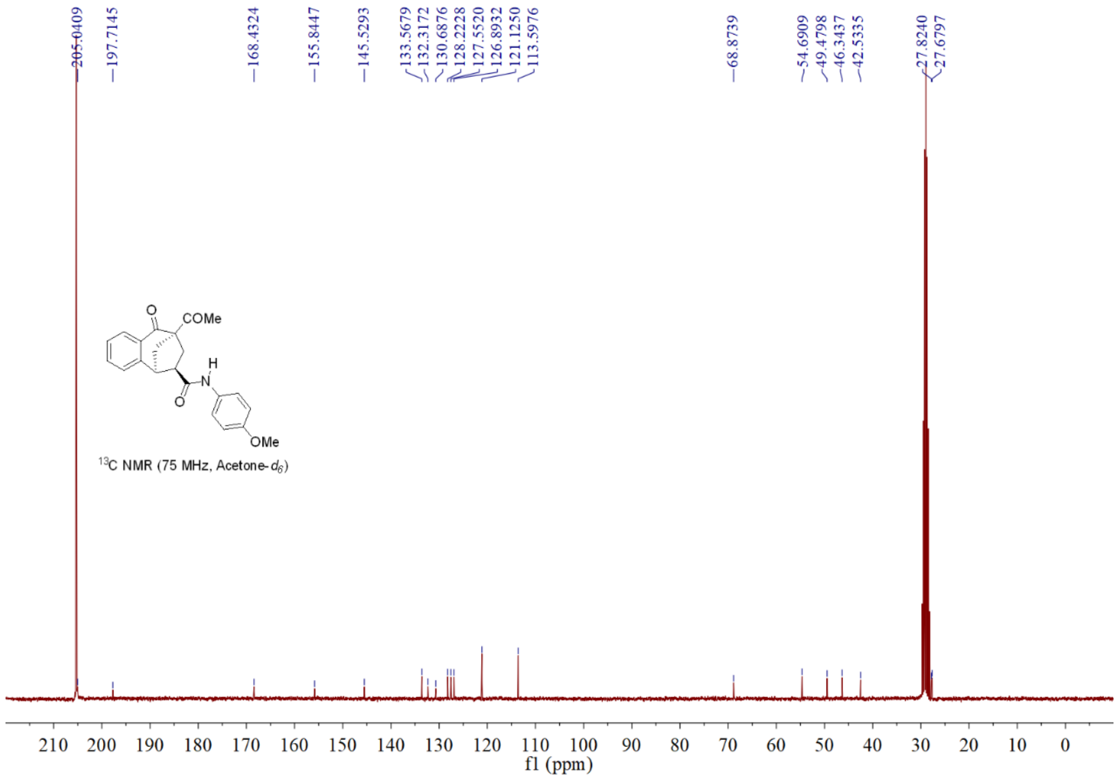

Supplementary Figure 208. ^{13}C NMR of **7ao**

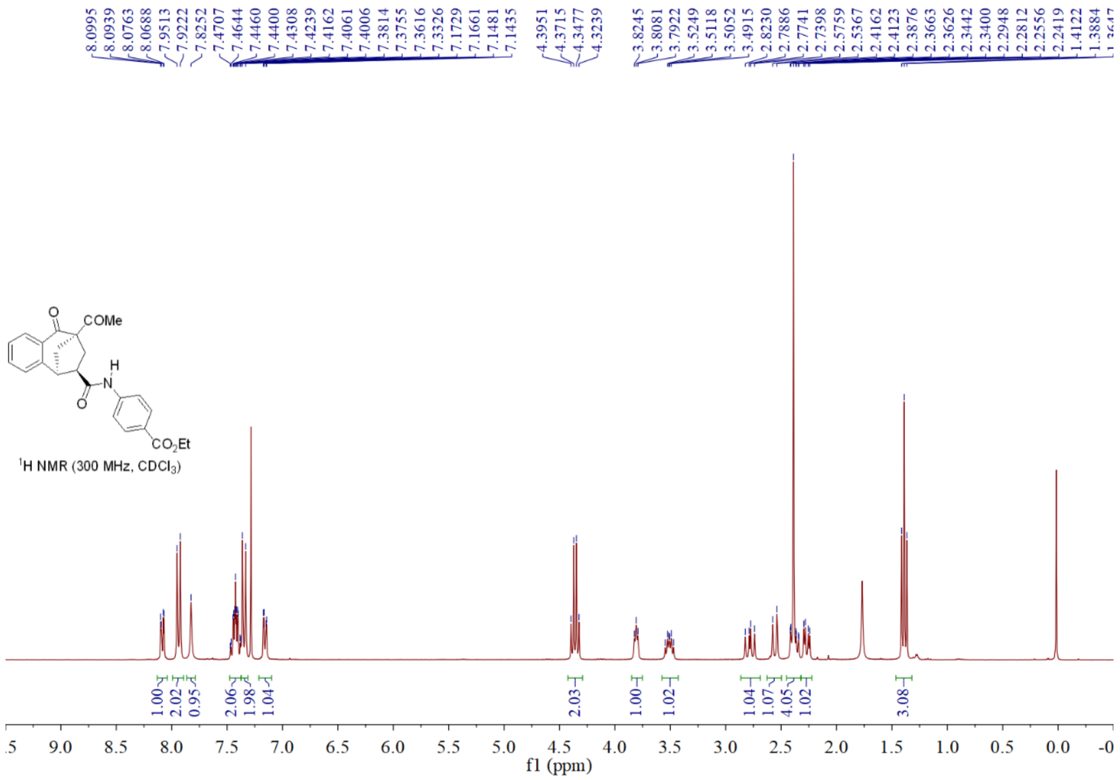

Supplementary Figure 209. ^{1}H NMR of **7ap**

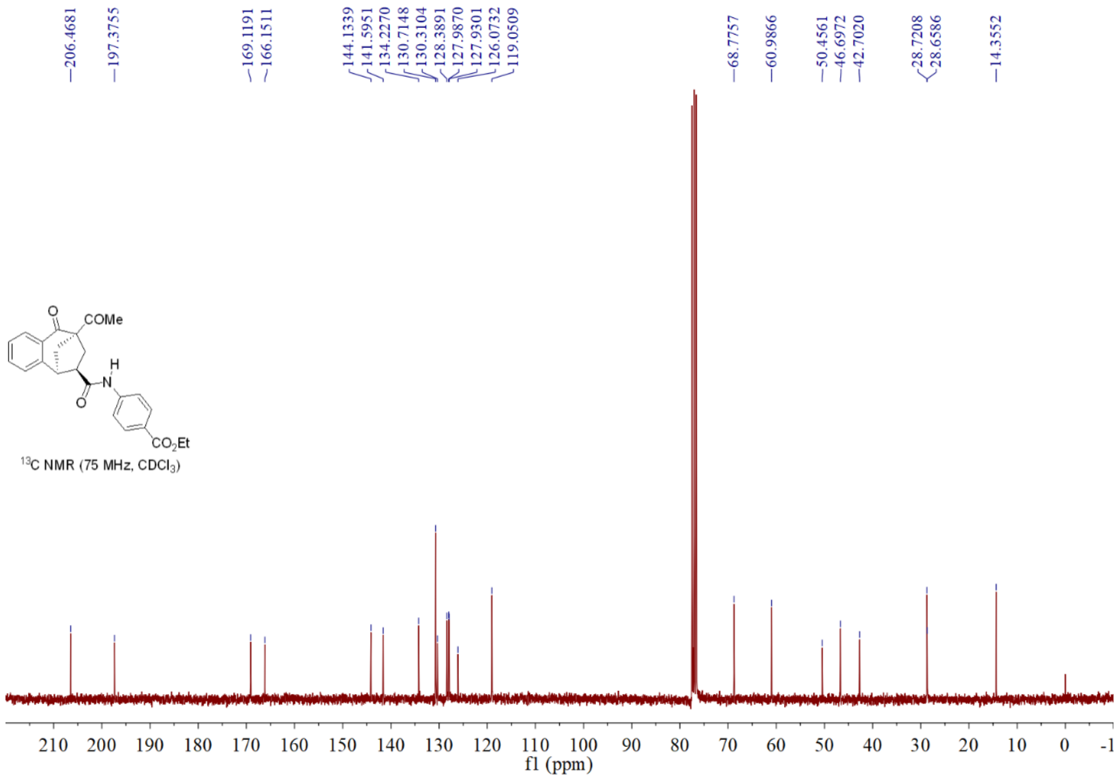

Supplementary Figure 210. ^{13}C NMR of **7ap**

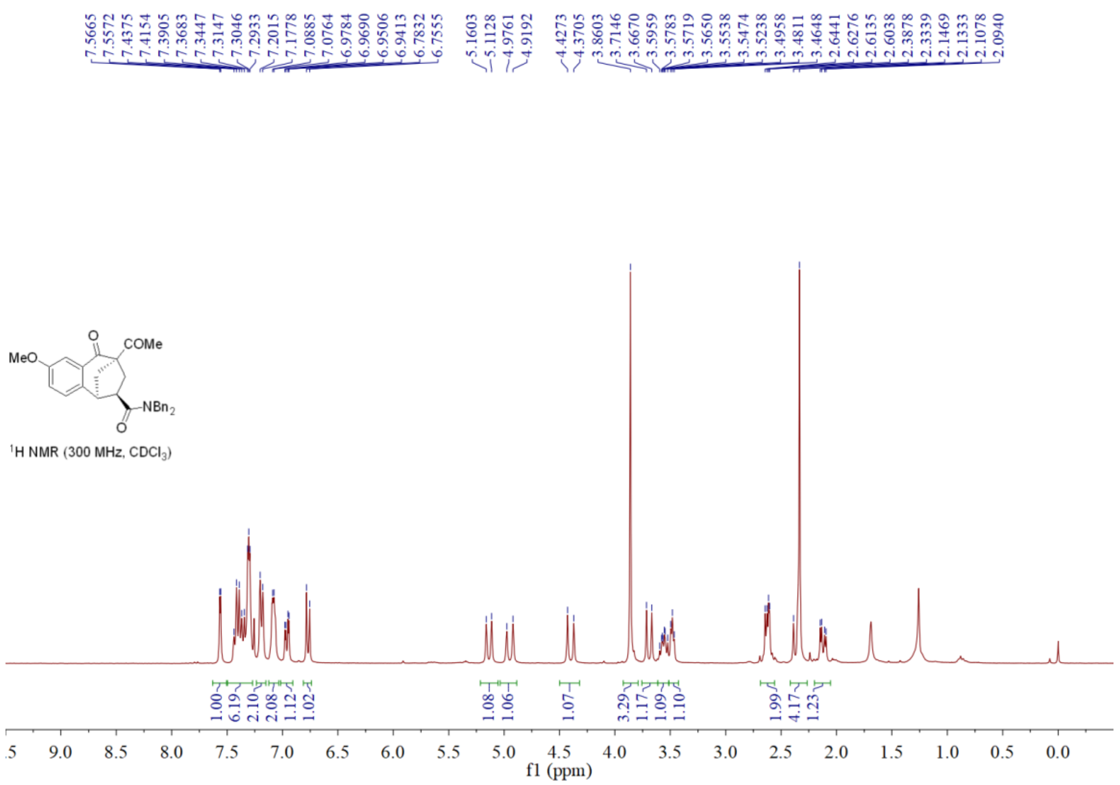

Supplementary Figure 211. ^{1}H NMR of **7fb**

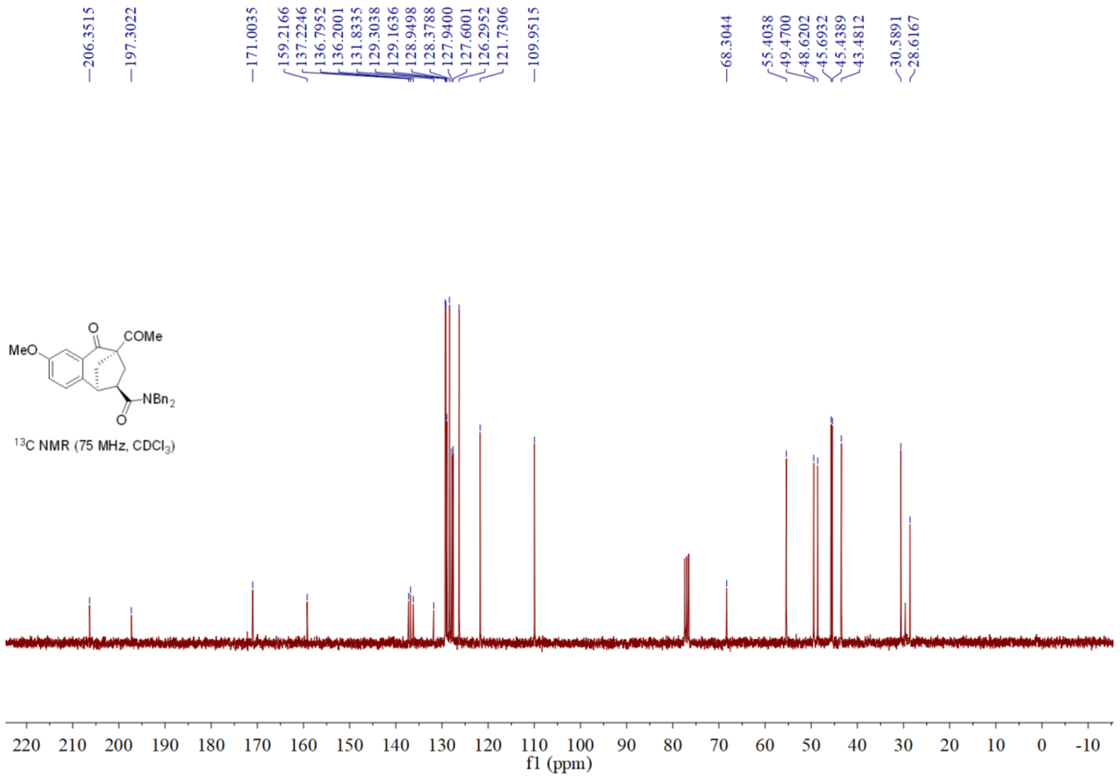

Supplementary Figure 212. ^{13}C NMR of **7fb**

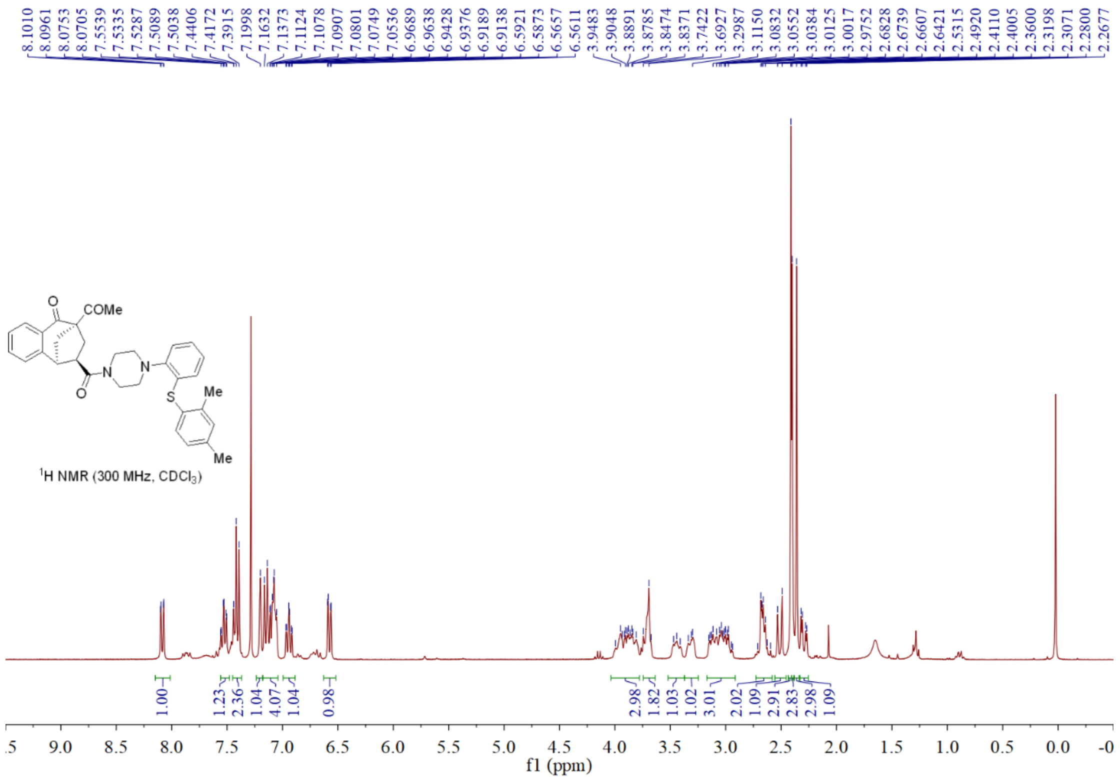

Supplementary Figure 213. ^{1}H NMR of **7aq**

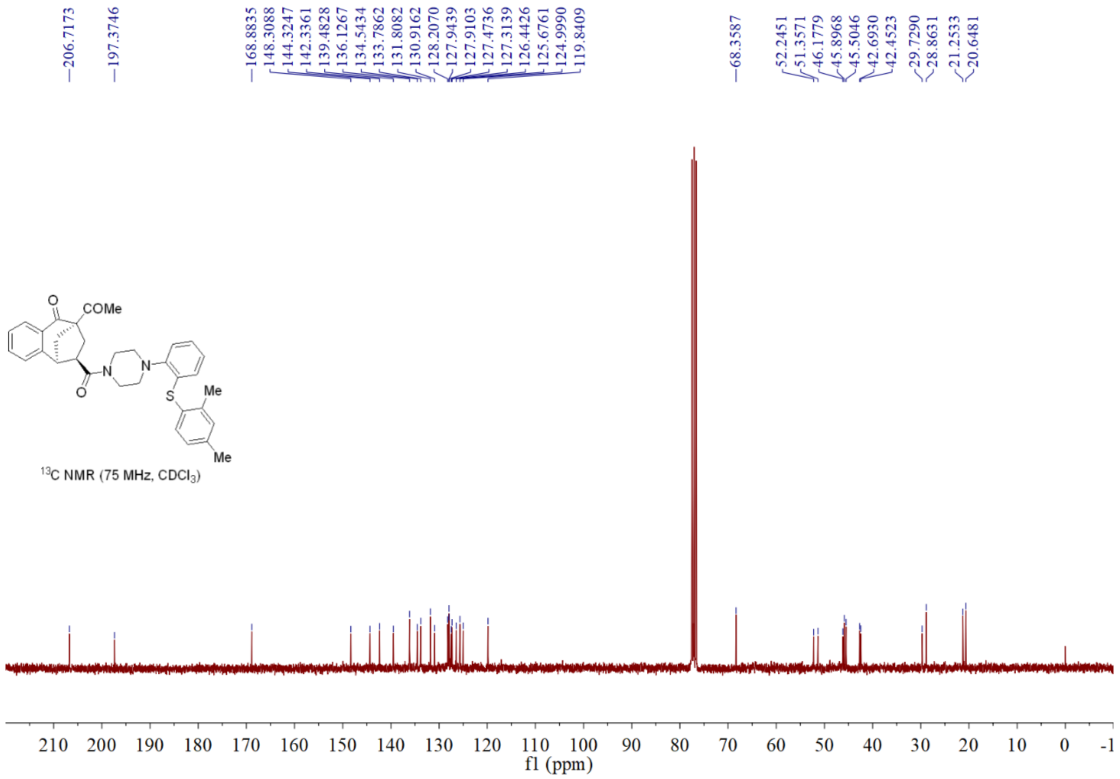

Supplementary Figure 214. ^{13}C NMR of **7aq**

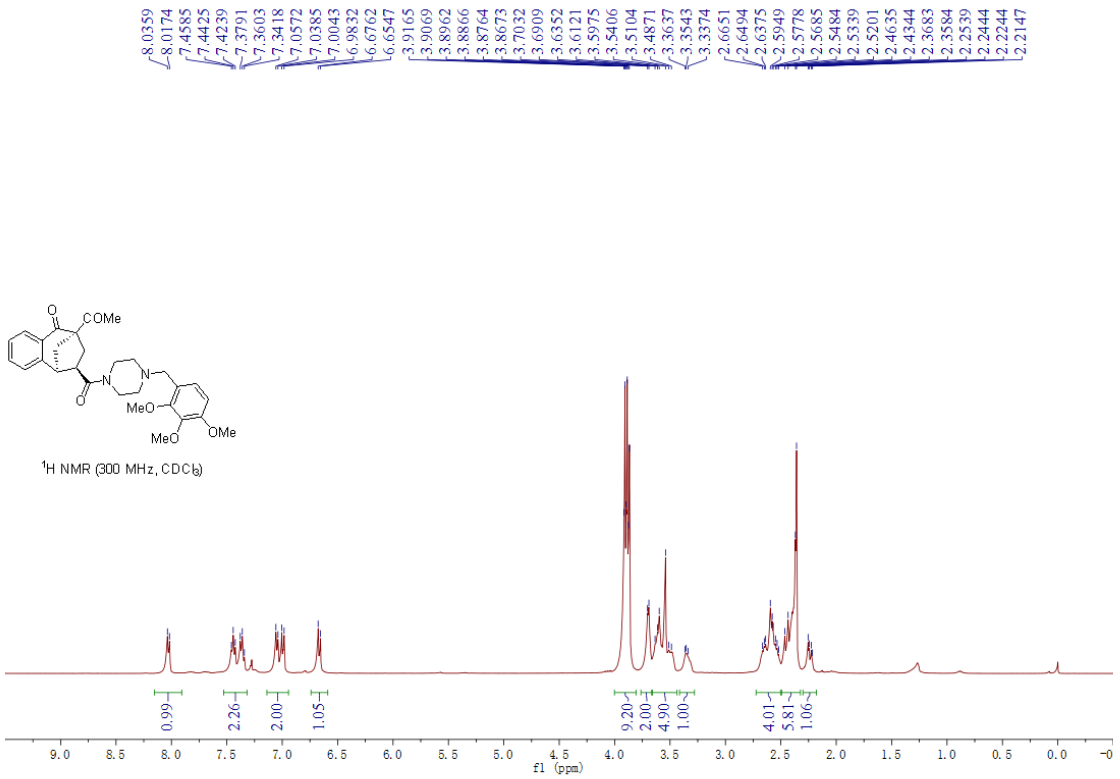

Supplementary Figure 215. ^{1}H NMR of **7ar**

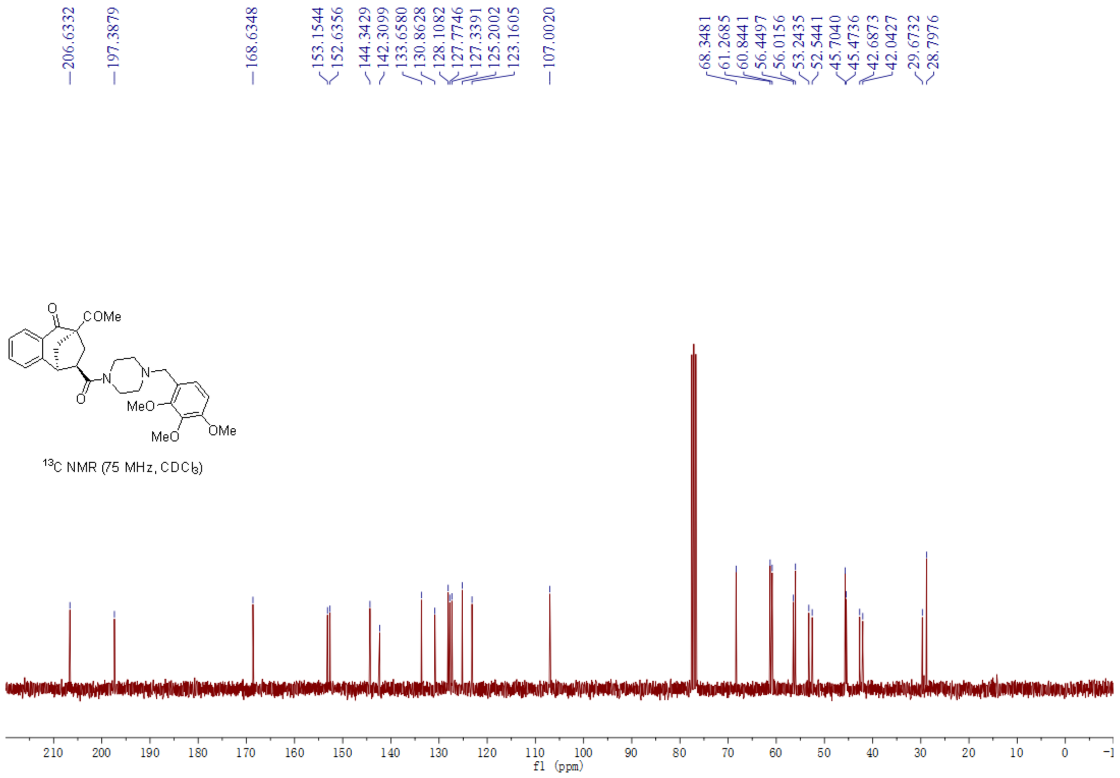

Supplementary Figure 216. ^{13}C NMR of **7ar**

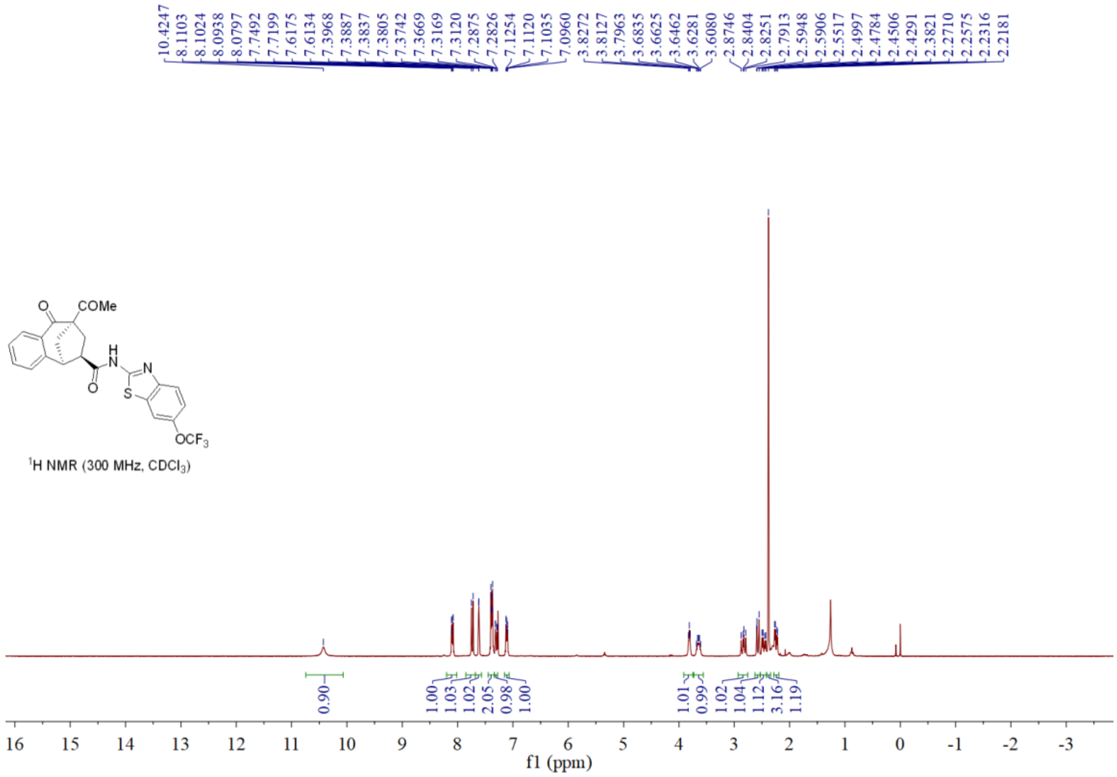

Supplementary Figure 217. ^{1}H NMR of **7as**

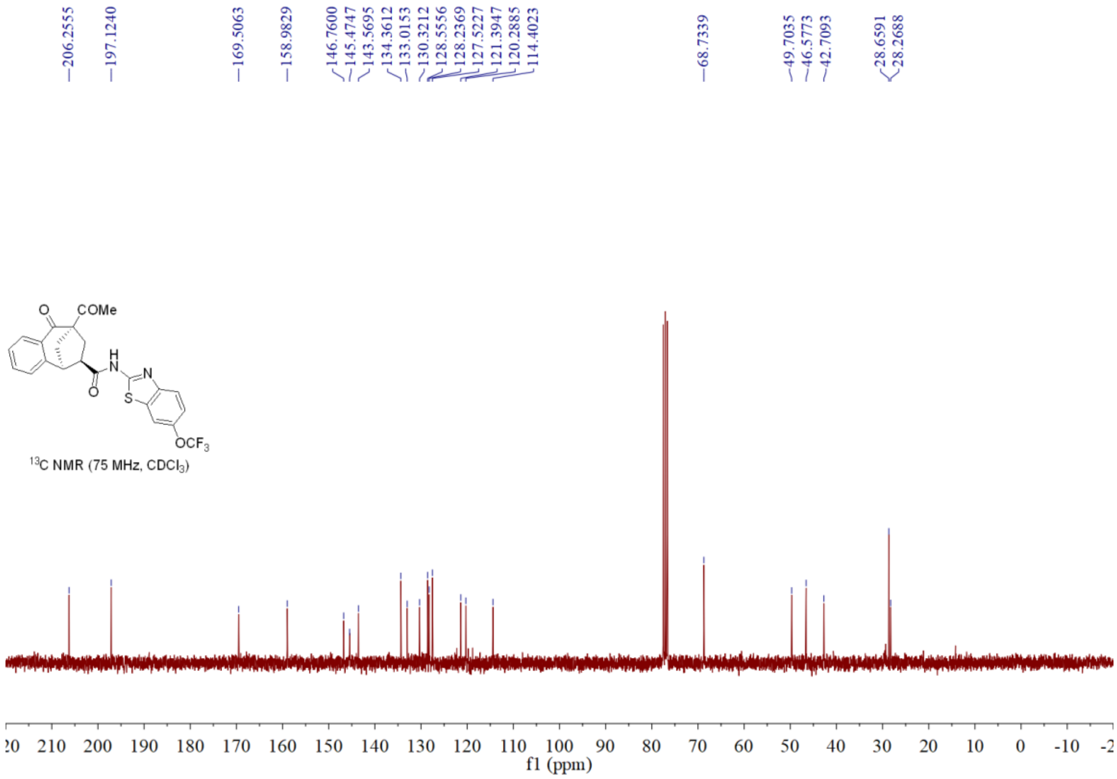

Supplementary Figure 218. ^{13}C NMR of **7as**

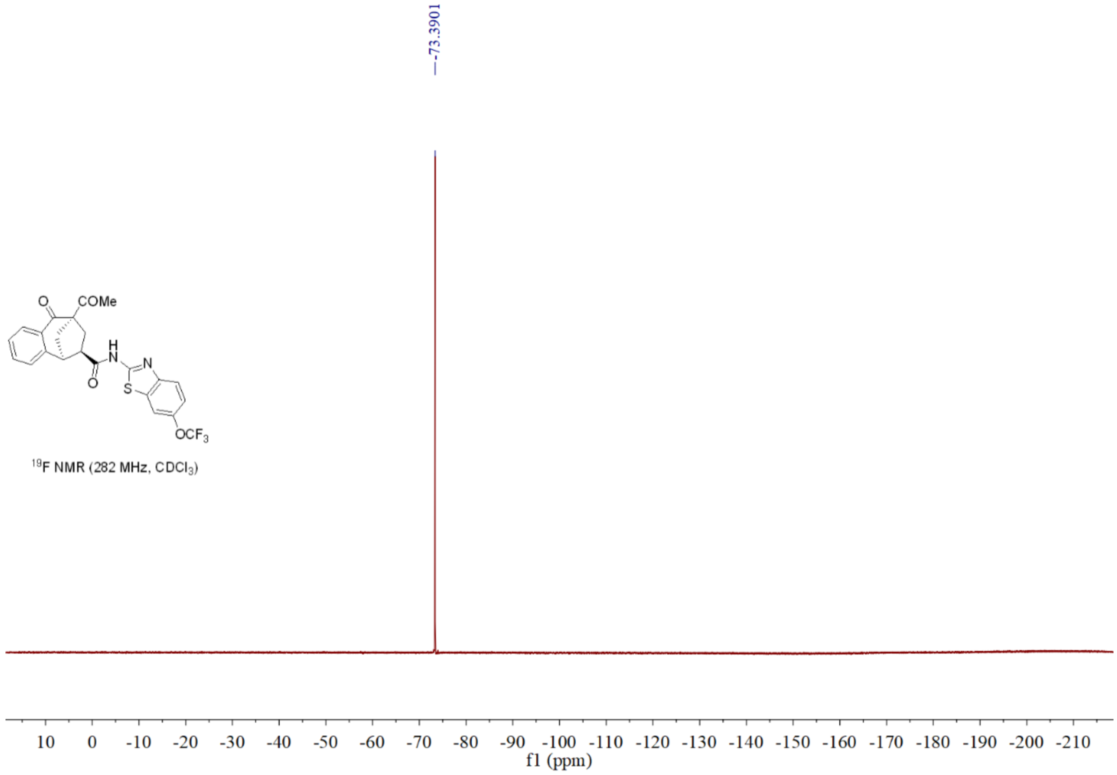

Supplementary Figure 219. ^{19}F NMR of **7as**

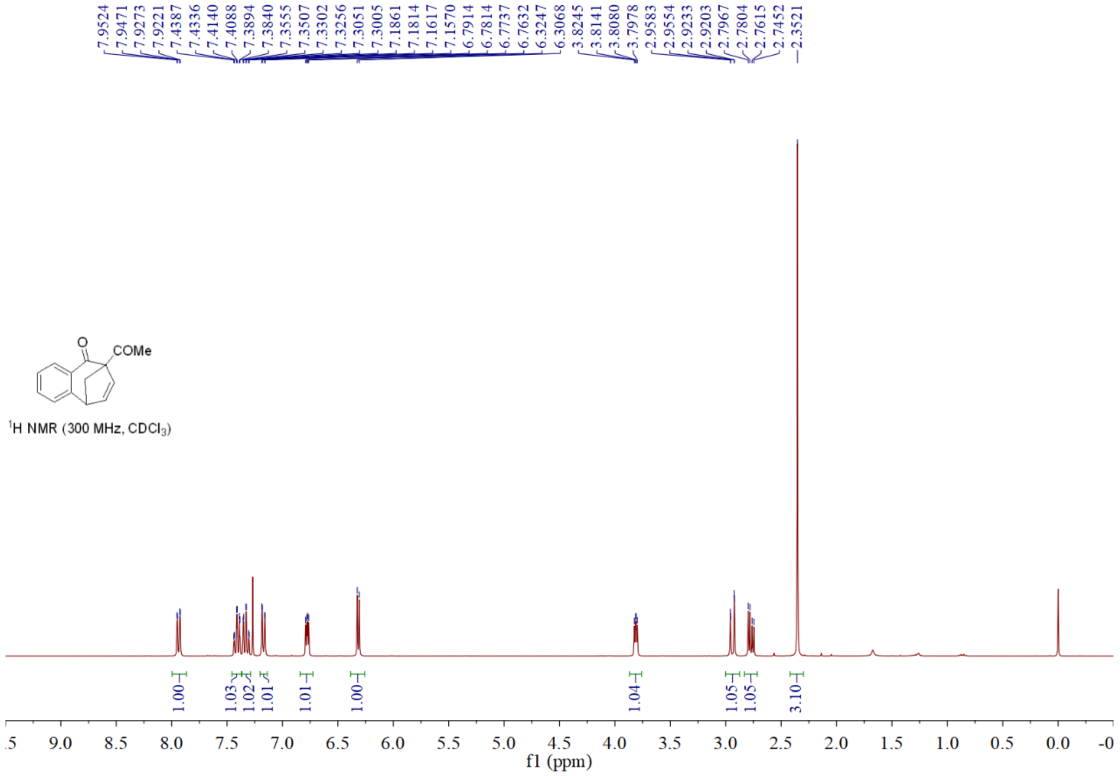

Supplementary Figure 220. ^{1}H NMR of **3a’**

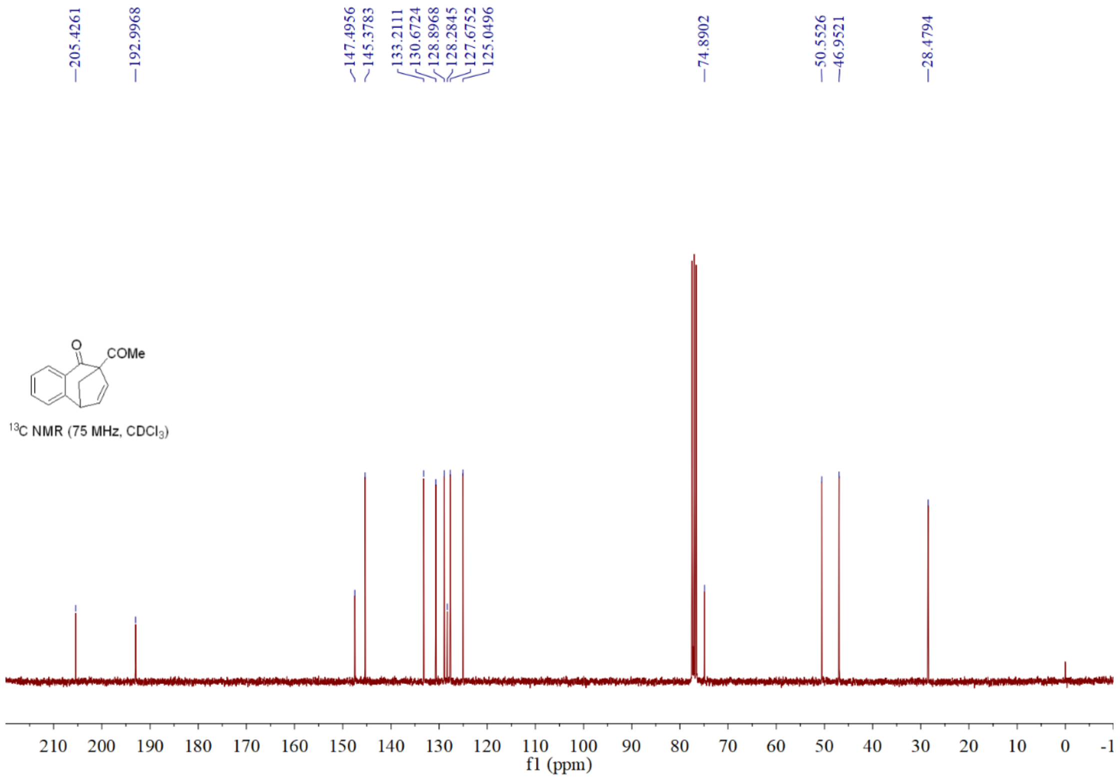

Supplementary Figure 221. ^{13}C NMR of **3a’**

Supplementary References

1. Tietze, L. F., Redert, T., Bell, H.P., Hellkamp, S. & Levy, L. M. Efficient Synthesis of the Structural Core of Tetracyclines by a Palladium-Catalyzed Domino Tsuji-Trost-Heck-Mizoroki Reaction. *Chem. Eur. J.* **14**, 2527-2535 (2008).
2. Okamoto, K., Sasakura, K., Shimbayashi, T. & Ohe, K. Ruthenium-catalyzed Decarboxylative and Dehydrogenative Formation of Highly Substituted Pyridines from Alkene-tethered Isoxazol-5(4H)-ones. *Chem. Lett.* **45**, 988-990 (2016).
3. Zieliński, G. K., Samojłowicz, C., Wdowik, T. & Grela, K. In tandem or alone: a remarkably selective transfer hydrogenation of alkenes catalyzed by ruthenium olefin metathesis catalysts. *Org. Biomol. Chem.* **13**, 2684-2688 (2015).
